# Supplementary material for: Reducing assembly complexity of microbial genomes with single-molecule sequencing
Source: Genome Biol. 2013 Sep 13;14(9):R101. doi: 10.1186/gb-2013-14-9-r101 (PMC4053942; doi:10.1186/gb-2013-14-9-r101)
Supplement: Additional file 2 — Report.log.krona. An interactive Krona [35] chart detailing the expected number of gaps for all strains, species, genera, and so on under various coverage and chemistry scenarios. [file gb-2013-14-9-r101-S2.html]

Javascript must be enabled to view this page.

members
magnitude
count
unassigned
score
gap
taxon
rank

50X\_c1.sizeAndGaps
100X\_c1.sizeAndGaps
150X\_c1.sizeAndGaps
200X\_c1.sizeAndGaps
50X\_c2.sizeAndGaps
100X\_c2.sizeAndGaps
150X\_c2.sizeAndGaps
200X\_c2.sizeAndGaps
50X\_c2xl.sizeAndGaps
100X\_c2xl.sizeAndGaps
150X\_c2xl.sizeAndGaps
200X\_c2xl.sizeAndGaps
50X\_xlxl.sizeAndGaps
100X\_xlxl.sizeAndGaps
150X\_xlxl.sizeAndGaps
200X\_xlxl.sizeAndGaps
50X\_zl.sizeAndGaps
100X\_zl.sizeAndGaps
150X\_zl.sizeAndGaps
200X\_zl.sizeAndGaps


7918.437918.437918.437918.437918.437918.437918.437918.437918.437918.437918.437918.437918.437918.437918.437918.437918.437918.437918.437918.43
22672267226722672267226722672267226722672267226722672267226722672267226722672267

7918.247918.247918.247918.247918.247918.247918.247918.247918.247918.247918.247918.247918.247918.247918.247918.247918.247918.247918.247918.24
22612261226122612261226122612261226122612261226122612261226122612261226122612261
3.615163.413673.325243.267541.116580.7416120.6279040.5920810.8622710.5896990.5193940.4935080.3151520.2623060.2379230.22760.14470.1231660.1133880.11158
141110101111111110000000
131567
no rank

7574.977574.977574.977574.977574.977574.977574.977574.977574.977574.977574.977574.977574.977574.977574.977574.977574.977574.977574.977574.97
21162116211621162116211621162116211621162116211621162116211621162116211621162116
3.67913.478873.389493.333141.152910.764030.6464150.6085960.8887160.6074810.5343130.5082880.322610.2680090.2425280.2325260.1473950.1251330.1151020.113612
151211101111111110000000
2
superkingdom

3.823.823.823.823.823.823.823.823.823.823.823.823.823.823.823.823.823.823.823.82
22222222222222222222
2.583722.583722.098612.238690000000000000000
55330000000000000000
68297
phylum

3.823.823.823.823.823.823.823.823.823.823.823.823.823.823.823.823.823.823.823.82
22222222222222222222
2.583722.583722.098612.238690000000000000000
55330000000000000000
203486
class

3.823.823.823.823.823.823.823.823.823.823.823.823.823.823.823.823.823.823.823.82
22222222222222222222
2.583722.583722.098612.238690000000000000000
55330000000000000000
203487
order

3.823.823.823.823.823.823.823.823.823.823.823.823.823.823.823.823.823.823.823.82
22222222222222222222
2.583722.583722.098612.238690000000000000000
55330000000000000000
203488
family

3.823.823.823.823.823.823.823.823.823.823.823.823.823.823.823.823.823.823.823.82
22222222222222222222
2.583722.583722.098612.238690000000000000000
55330000000000000000
13
genus

1.861.861.861.861.861.861.861.861.861.861.861.861.861.861.861.861.861.861.861.86
11111111111111111111
2.791762.791762.098612.386290000000000000000
66340000000000000000
513050
species

1.861.861.861.861.861.861.861.861.861.861.861.861.861.861.861.861.861.861.861.86
11111111111111111111
2.791762.791762.098612.386290000000000000000
66340000000000000000
515635

Dictyoglomus\_turgidum\_DSM\_6724
Dictyoglomus\_turgidum\_DSM\_6724
Dictyoglomus\_turgidum\_DSM\_6724
Dictyoglomus\_turgidum\_DSM\_6724
Dictyoglomus\_turgidum\_DSM\_6724
Dictyoglomus\_turgidum\_DSM\_6724
Dictyoglomus\_turgidum\_DSM\_6724
Dictyoglomus\_turgidum\_DSM\_6724
Dictyoglomus\_turgidum\_DSM\_6724
Dictyoglomus\_turgidum\_DSM\_6724
Dictyoglomus\_turgidum\_DSM\_6724
Dictyoglomus\_turgidum\_DSM\_6724
Dictyoglomus\_turgidum\_DSM\_6724
Dictyoglomus\_turgidum\_DSM\_6724
Dictyoglomus\_turgidum\_DSM\_6724
Dictyoglomus\_turgidum\_DSM\_6724
Dictyoglomus\_turgidum\_DSM\_6724
Dictyoglomus\_turgidum\_DSM\_6724
Dictyoglomus\_turgidum\_DSM\_6724
Dictyoglomus\_turgidum\_DSM\_6724
no rank

1.961.961.961.961.961.961.961.961.961.961.961.961.961.961.961.961.961.961.961.96
11111111111111111111
2.386292.386292.098612.098610000000000000000
44330000000000000000
14
species

1.961.961.961.961.961.961.961.961.961.961.961.961.961.961.961.961.961.961.961.96
11111111111111111111
2.386292.386292.098612.098610000000000000000
44330000000000000000
309799

Dictyoglomus\_thermophilum\_H-6-12
Dictyoglomus\_thermophilum\_H-6-12
Dictyoglomus\_thermophilum\_H-6-12
Dictyoglomus\_thermophilum\_H-6-12
Dictyoglomus\_thermophilum\_H-6-12
Dictyoglomus\_thermophilum\_H-6-12
Dictyoglomus\_thermophilum\_H-6-12
Dictyoglomus\_thermophilum\_H-6-12
Dictyoglomus\_thermophilum\_H-6-12
Dictyoglomus\_thermophilum\_H-6-12
Dictyoglomus\_thermophilum\_H-6-12
Dictyoglomus\_thermophilum\_H-6-12
Dictyoglomus\_thermophilum\_H-6-12
Dictyoglomus\_thermophilum\_H-6-12
Dictyoglomus\_thermophilum\_H-6-12
Dictyoglomus\_thermophilum\_H-6-12
Dictyoglomus\_thermophilum\_H-6-12
Dictyoglomus\_thermophilum\_H-6-12
Dictyoglomus\_thermophilum\_H-6-12
Dictyoglomus\_thermophilum\_H-6-12
no rank

2.932.932.932.932.932.932.932.932.932.932.932.932.932.932.932.932.932.932.932.93
11111111111111111111
3.639063.484913.302593.302590000000000000000
141210100000000000000000
200938
phylum

2.932.932.932.932.932.932.932.932.932.932.932.932.932.932.932.932.932.932.932.93
11111111111111111111
3.639063.484913.302593.302590000000000000000
141210100000000000000000
118001
class

2.932.932.932.932.932.932.932.932.932.932.932.932.932.932.932.932.932.932.932.93
11111111111111111111
3.639063.484913.302593.302590000000000000000
141210100000000000000000
189769
order

2.932.932.932.932.932.932.932.932.932.932.932.932.932.932.932.932.932.932.932.93
11111111111111111111
3.639063.484913.302593.302590000000000000000
141210100000000000000000
189770
family

2.932.932.932.932.932.932.932.932.932.932.932.932.932.932.932.932.932.932.932.93
11111111111111111111
3.639063.484913.302593.302590000000000000000
141210100000000000000000
393029
genus

2.932.932.932.932.932.932.932.932.932.932.932.932.932.932.932.932.932.932.932.93
11111111111111111111
3.639063.484913.302593.302590000000000000000
141210100000000000000000
936456
species

2.932.932.932.932.932.932.932.932.932.932.932.932.932.932.932.932.932.932.932.93
11111111111111111111
3.639063.484913.302593.302590000000000000000
141210100000000000000000
653733

Desulfurispirillum\_indicum\_S5
Desulfurispirillum\_indicum\_S5
Desulfurispirillum\_indicum\_S5
Desulfurispirillum\_indicum\_S5
Desulfurispirillum\_indicum\_S5
Desulfurispirillum\_indicum\_S5
Desulfurispirillum\_indicum\_S5
Desulfurispirillum\_indicum\_S5
Desulfurispirillum\_indicum\_S5
Desulfurispirillum\_indicum\_S5
Desulfurispirillum\_indicum\_S5
Desulfurispirillum\_indicum\_S5
Desulfurispirillum\_indicum\_S5
Desulfurispirillum\_indicum\_S5
Desulfurispirillum\_indicum\_S5
Desulfurispirillum\_indicum\_S5
Desulfurispirillum\_indicum\_S5
Desulfurispirillum\_indicum\_S5
Desulfurispirillum\_indicum\_S5
Desulfurispirillum\_indicum\_S5
no rank

320.14320.14320.14320.14320.14320.14320.14320.14320.14320.14320.14320.14320.14320.14320.14320.14320.14320.14320.14320.14
7171717171717171717171717171717171717171
3.889223.63023.513093.440951.025770.687520.6889790.5964540.7837370.5828860.5267230.5340730.2747610.227910.154580.1627640.06664230.06664230.06664230.0666423
181412111111111100000000
1117
phylum

52.1152.1152.1152.1152.1152.1152.1152.1152.1152.1152.1152.1152.1152.1152.1152.1152.1152.1152.1152.11
88888888888888888888
4.780314.547824.344864.213351.173220.7628290.7628290.7628290.8522140.8659160.7628290.7628290.4174310.6880740.4174310.4174310000
443528251111111111110000
1150
order

7.757.757.757.757.757.757.757.757.757.757.757.757.757.757.757.757.757.757.757.75
11111111111111111111
5.510865.356715.189655.219511.6931511111.693151111110000
917866682111121111110000
1205
genus

7.757.757.757.757.757.757.757.757.757.757.757.757.757.757.757.757.757.757.757.75
11111111111111111111
5.510865.356715.189655.219511.6931511111.693151111110000
917866682111121111110000
1206
species

7.757.757.757.757.757.757.757.757.757.757.757.757.757.757.757.757.757.757.757.75
11111111111111111111
5.510865.356715.189655.219511.6931511111.693151111110000
917866682111121111110000
203124

Trichodesmium\_erythraeum\_IMS101
Trichodesmium\_erythraeum\_IMS101
Trichodesmium\_erythraeum\_IMS101
Trichodesmium\_erythraeum\_IMS101
Trichodesmium\_erythraeum\_IMS101
Trichodesmium\_erythraeum\_IMS101
Trichodesmium\_erythraeum\_IMS101
Trichodesmium\_erythraeum\_IMS101
Trichodesmium\_erythraeum\_IMS101
Trichodesmium\_erythraeum\_IMS101
Trichodesmium\_erythraeum\_IMS101
Trichodesmium\_erythraeum\_IMS101
Trichodesmium\_erythraeum\_IMS101
Trichodesmium\_erythraeum\_IMS101
Trichodesmium\_erythraeum\_IMS101
Trichodesmium\_erythraeum\_IMS101
Trichodesmium\_erythraeum\_IMS101
Trichodesmium\_erythraeum\_IMS101
Trichodesmium\_erythraeum\_IMS101
Trichodesmium\_erythraeum\_IMS101
no rank

5.625.625.625.625.625.625.625.625.625.625.625.625.625.625.625.625.625.625.625.62
11111111111111111111
4.784194.610924.555354.25812.098611.693151.693151.693152.098611.693151.693151.6931501000000
443735263222322201000000
241421
genus

5.625.625.625.625.625.625.625.625.625.625.625.625.625.625.625.625.625.625.625.62
11111111111111111111
4.784194.610924.555354.25812.098611.693151.693151.693152.098611.693151.693151.6931501000000
443735263222322201000000
241425
species

5.625.625.625.625.625.625.625.625.625.625.625.625.625.625.625.625.625.625.625.62
11111111111111111111
4.784194.610924.555354.25812.098611.693151.693151.693152.098611.693151.693151.6931501000000
443735263222322201000000
1173022

Crinalium\_epipsammum\_PCC\_9333
Crinalium\_epipsammum\_PCC\_9333
Crinalium\_epipsammum\_PCC\_9333
Crinalium\_epipsammum\_PCC\_9333
Crinalium\_epipsammum\_PCC\_9333
Crinalium\_epipsammum\_PCC\_9333
Crinalium\_epipsammum\_PCC\_9333
Crinalium\_epipsammum\_PCC\_9333
Crinalium\_epipsammum\_PCC\_9333
Crinalium\_epipsammum\_PCC\_9333
Crinalium\_epipsammum\_PCC\_9333
Crinalium\_epipsammum\_PCC\_9333
Crinalium\_epipsammum\_PCC\_9333
Crinalium\_epipsammum\_PCC\_9333
Crinalium\_epipsammum\_PCC\_9333
Crinalium\_epipsammum\_PCC\_9333
Crinalium\_epipsammum\_PCC\_9333
Crinalium\_epipsammum\_PCC\_9333
Crinalium\_epipsammum\_PCC\_9333
Crinalium\_epipsammum\_PCC\_9333
no rank

5.135.135.135.135.135.135.135.135.135.135.135.135.135.135.135.135.135.135.135.13
11111111111111111111
4.784194.526364.36734.25811.69315111111101000000
443429262111111101000000
47251
genus

5.135.135.135.135.135.135.135.135.135.135.135.135.135.135.135.135.135.135.135.13
11111111111111111111
4.784194.526364.36734.25811.69315111111101000000
443429262111111101000000
111781

Leptolyngbya\_sp.\_PCC\_7376
Leptolyngbya\_sp.\_PCC\_7376
Leptolyngbya\_sp.\_PCC\_7376
Leptolyngbya\_sp.\_PCC\_7376
Leptolyngbya\_sp.\_PCC\_7376
Leptolyngbya\_sp.\_PCC\_7376
Leptolyngbya\_sp.\_PCC\_7376
Leptolyngbya\_sp.\_PCC\_7376
Leptolyngbya\_sp.\_PCC\_7376
Leptolyngbya\_sp.\_PCC\_7376
Leptolyngbya\_sp.\_PCC\_7376
Leptolyngbya\_sp.\_PCC\_7376
Leptolyngbya\_sp.\_PCC\_7376
Leptolyngbya\_sp.\_PCC\_7376
Leptolyngbya\_sp.\_PCC\_7376
Leptolyngbya\_sp.\_PCC\_7376
Leptolyngbya\_sp.\_PCC\_7376
Leptolyngbya\_sp.\_PCC\_7376
Leptolyngbya\_sp.\_PCC\_7376
Leptolyngbya\_sp.\_PCC\_7376
species

16.0716.0716.0716.0716.0716.0716.0716.0716.0716.0716.0716.0716.0716.0716.0716.0716.0716.0716.0716.07
22222222222222222222
5.293844.971164.830614.788831.713421.081.081.081.228041.081.081.080.8713331.080.8713330.8713330000
735346442111111111110000
1158
genus

8.278.278.278.278.278.278.278.278.278.278.278.278.278.278.278.278.278.278.278.27
11111111111111111111
5.653965.418845.204695.219512.386292.098612.098612.098612.386292.098612.098612.098611.693152.098611.693151.693150000
1058367684333433323220000
482564
species

8.278.278.278.278.278.278.278.278.278.278.278.278.278.278.278.278.278.278.278.27
11111111111111111111
5.653965.418845.204695.219512.386292.098612.098612.098612.386292.098612.098612.098611.693152.098611.693151.693150000
1058367684333433323220000
179408

Oscillatoria\_nigro-viridis\_PCC\_7112
Oscillatoria\_nigro-viridis\_PCC\_7112
Oscillatoria\_nigro-viridis\_PCC\_7112
Oscillatoria\_nigro-viridis\_PCC\_7112
Oscillatoria\_nigro-viridis\_PCC\_7112
Oscillatoria\_nigro-viridis\_PCC\_7112
Oscillatoria\_nigro-viridis\_PCC\_7112
Oscillatoria\_nigro-viridis\_PCC\_7112
Oscillatoria\_nigro-viridis\_PCC\_7112
Oscillatoria\_nigro-viridis\_PCC\_7112
Oscillatoria\_nigro-viridis\_PCC\_7112
Oscillatoria\_nigro-viridis\_PCC\_7112
Oscillatoria\_nigro-viridis\_PCC\_7112
Oscillatoria\_nigro-viridis\_PCC\_7112
Oscillatoria\_nigro-viridis\_PCC\_7112
Oscillatoria\_nigro-viridis\_PCC\_7112
Oscillatoria\_nigro-viridis\_PCC\_7112
Oscillatoria\_nigro-viridis\_PCC\_7112
Oscillatoria\_nigro-viridis\_PCC\_7112
Oscillatoria\_nigro-viridis\_PCC\_7112
no rank

7.807.807.807.807.807.807.807.807.807.807.807.807.807.807.807.807.807.807.807.80
11111111111111111111
4.912024.496514.433994.33221000000000000000
503331281000000000000000
118323
species

7.807.807.807.807.807.807.807.807.807.807.807.807.807.807.807.807.807.807.807.80
11111111111111111111
4.912024.496514.433994.33221000000000000000
503331281000000000000000
56110

Oscillatoria\_acuminata\_PCC\_6304
Oscillatoria\_acuminata\_PCC\_6304
Oscillatoria\_acuminata\_PCC\_6304
Oscillatoria\_acuminata\_PCC\_6304
Oscillatoria\_acuminata\_PCC\_6304
Oscillatoria\_acuminata\_PCC\_6304
Oscillatoria\_acuminata\_PCC\_6304
Oscillatoria\_acuminata\_PCC\_6304
Oscillatoria\_acuminata\_PCC\_6304
Oscillatoria\_acuminata\_PCC\_6304
Oscillatoria\_acuminata\_PCC\_6304
Oscillatoria\_acuminata\_PCC\_6304
Oscillatoria\_acuminata\_PCC\_6304
Oscillatoria\_acuminata\_PCC\_6304
Oscillatoria\_acuminata\_PCC\_6304
Oscillatoria\_acuminata\_PCC\_6304
Oscillatoria\_acuminata\_PCC\_6304
Oscillatoria\_acuminata\_PCC\_6304
Oscillatoria\_acuminata\_PCC\_6304
Oscillatoria\_acuminata\_PCC\_6304
no rank

4.684.684.684.684.684.684.684.684.684.684.684.684.684.684.684.684.684.684.684.68
11111111111111111111
2.945912.791762.609442.386290000000000000000
76540000000000000000
63132
genus

4.684.684.684.684.684.684.684.684.684.684.684.684.684.684.684.684.684.684.684.68
11111111111111111111
2.945912.791762.609442.386290000000000000000
76540000000000000000
1173025

Geitlerinema\_sp.\_PCC\_7407
Geitlerinema\_sp.\_PCC\_7407
Geitlerinema\_sp.\_PCC\_7407
Geitlerinema\_sp.\_PCC\_7407
Geitlerinema\_sp.\_PCC\_7407
Geitlerinema\_sp.\_PCC\_7407
Geitlerinema\_sp.\_PCC\_7407
Geitlerinema\_sp.\_PCC\_7407
Geitlerinema\_sp.\_PCC\_7407
Geitlerinema\_sp.\_PCC\_7407
Geitlerinema\_sp.\_PCC\_7407
Geitlerinema\_sp.\_PCC\_7407
Geitlerinema\_sp.\_PCC\_7407
Geitlerinema\_sp.\_PCC\_7407
Geitlerinema\_sp.\_PCC\_7407
Geitlerinema\_sp.\_PCC\_7407
Geitlerinema\_sp.\_PCC\_7407
Geitlerinema\_sp.\_PCC\_7407
Geitlerinema\_sp.\_PCC\_7407
Geitlerinema\_sp.\_PCC\_7407
species

4.894.894.894.894.894.894.894.894.894.894.894.894.894.894.894.894.894.894.894.89
11111111111111111111
3.639063.639063.639063.564950000000000000000
141414130000000000000000
1152
genus

4.894.894.894.894.894.894.894.894.894.894.894.894.894.894.894.894.894.894.894.89
11111111111111111111
3.639063.639063.639063.564950000000000000000
141414130000000000000000
82654

Pseudanabaena\_sp.\_PCC\_7367
Pseudanabaena\_sp.\_PCC\_7367
Pseudanabaena\_sp.\_PCC\_7367
Pseudanabaena\_sp.\_PCC\_7367
Pseudanabaena\_sp.\_PCC\_7367
Pseudanabaena\_sp.\_PCC\_7367
Pseudanabaena\_sp.\_PCC\_7367
Pseudanabaena\_sp.\_PCC\_7367
Pseudanabaena\_sp.\_PCC\_7367
Pseudanabaena\_sp.\_PCC\_7367
Pseudanabaena\_sp.\_PCC\_7367
Pseudanabaena\_sp.\_PCC\_7367
Pseudanabaena\_sp.\_PCC\_7367
Pseudanabaena\_sp.\_PCC\_7367
Pseudanabaena\_sp.\_PCC\_7367
Pseudanabaena\_sp.\_PCC\_7367
Pseudanabaena\_sp.\_PCC\_7367
Pseudanabaena\_sp.\_PCC\_7367
Pseudanabaena\_sp.\_PCC\_7367
Pseudanabaena\_sp.\_PCC\_7367
species

7.977.977.977.977.977.977.977.977.977.977.977.977.977.977.977.977.977.977.977.97
11111111111111111111
4.806664.465743.833213.484910000000000000000
453217120000000000000000
44471
genus

7.977.977.977.977.977.977.977.977.977.977.977.977.977.977.977.977.977.977.977.97
11111111111111111111
4.806664.465743.833213.484910000000000000000
453217120000000000000000
1173027

Microcoleus\_sp.\_PCC\_7113
Microcoleus\_sp.\_PCC\_7113
Microcoleus\_sp.\_PCC\_7113
Microcoleus\_sp.\_PCC\_7113
Microcoleus\_sp.\_PCC\_7113
Microcoleus\_sp.\_PCC\_7113
Microcoleus\_sp.\_PCC\_7113
Microcoleus\_sp.\_PCC\_7113
Microcoleus\_sp.\_PCC\_7113
Microcoleus\_sp.\_PCC\_7113
Microcoleus\_sp.\_PCC\_7113
Microcoleus\_sp.\_PCC\_7113
Microcoleus\_sp.\_PCC\_7113
Microcoleus\_sp.\_PCC\_7113
Microcoleus\_sp.\_PCC\_7113
Microcoleus\_sp.\_PCC\_7113
Microcoleus\_sp.\_PCC\_7113
Microcoleus\_sp.\_PCC\_7113
Microcoleus\_sp.\_PCC\_7113
Microcoleus\_sp.\_PCC\_7113
species

4.664.664.664.664.664.664.664.664.664.664.664.664.664.664.664.664.664.664.664.66
11111111111111111111
10010000000000000000
10010000000000000000
307596
class

4.664.664.664.664.664.664.664.664.664.664.664.664.664.664.664.664.664.664.664.66
11111111111111111111
10010000000000000000
10010000000000000000
307595
order

4.664.664.664.664.664.664.664.664.664.664.664.664.664.664.664.664.664.664.664.66
11111111111111111111
10010000000000000000
10010000000000000000
33071
genus

4.664.664.664.664.664.664.664.664.664.664.664.664.664.664.664.664.664.664.664.66
11111111111111111111
10010000000000000000
10010000000000000000
33072
species

4.664.664.664.664.664.664.664.664.664.664.664.664.664.664.664.664.664.664.664.66
11111111111111111111
10010000000000000000
10010000000000000000
251221

Gloeobacter\_violaceus\_PCC\_7421
Gloeobacter\_violaceus\_PCC\_7421
Gloeobacter\_violaceus\_PCC\_7421
Gloeobacter\_violaceus\_PCC\_7421
Gloeobacter\_violaceus\_PCC\_7421
Gloeobacter\_violaceus\_PCC\_7421
Gloeobacter\_violaceus\_PCC\_7421
Gloeobacter\_violaceus\_PCC\_7421
Gloeobacter\_violaceus\_PCC\_7421
Gloeobacter\_violaceus\_PCC\_7421
Gloeobacter\_violaceus\_PCC\_7421
Gloeobacter\_violaceus\_PCC\_7421
Gloeobacter\_violaceus\_PCC\_7421
Gloeobacter\_violaceus\_PCC\_7421
Gloeobacter\_violaceus\_PCC\_7421
Gloeobacter\_violaceus\_PCC\_7421
Gloeobacter\_violaceus\_PCC\_7421
Gloeobacter\_violaceus\_PCC\_7421
Gloeobacter\_violaceus\_PCC\_7421
Gloeobacter\_violaceus\_PCC\_7421
no rank

22.3522.3522.3522.3522.3522.3522.3522.3522.3522.3522.3522.3522.3522.3522.3522.3522.3522.3522.3522.35
1212121212121212121212121212121212121212
0.9013170.7425170.6833570.6592950.07561520.07561520.07561520.07561520.1955260.07561520.07561520.07561520.07561520.07561520.07561520.07561520.07561520.07561520.07561520.0756152
11110000000000000000
1212
order

22.3522.3522.3522.3522.3522.3522.3522.3522.3522.3522.3522.3522.3522.3522.3522.3522.3522.3522.3522.35
1212121212121212121212121212121212121212
0.9013170.7425170.6833570.6592950.07561520.07561520.07561520.07561520.1955260.07561520.07561520.07561520.07561520.07561520.07561520.07561520.07561520.07561520.07561520.0756152
11110000000000000000
1217
family

22.3522.3522.3522.3522.3522.3522.3522.3522.3522.3522.3522.3522.3522.3522.3522.3522.3522.3522.3522.35
1212121212121212121212121212121212121212
0.9013170.7425170.6833570.6592950.07561520.07561520.07561520.07561520.1955260.07561520.07561520.07561520.07561520.07561520.07561520.07561520.07561520.07561520.07561520.0756152
11110000000000000000
1218
genus

22.3522.3522.3522.3522.3522.3522.3522.3522.3522.3522.3522.3522.3522.3522.3522.3522.3522.3522.3522.35
1212121212121212121212121212121212121212
0.9013170.7425170.6833570.6592950.07561520.07561520.07561520.07561520.1955260.07561520.07561520.07561520.07561520.07561520.07561520.07561520.07561520.07561520.07561520.0756152
11110000000000000000
1219
species

1.741.741.741.741.741.741.741.741.741.741.741.741.741.741.741.741.741.741.741.74
11111111111111111111
00000000000000000000
00000000000000000000
93060

Prochlorococcus\_marinus\_str.\_MIT\_9215
Prochlorococcus\_marinus\_str.\_MIT\_9215
Prochlorococcus\_marinus\_str.\_MIT\_9215
Prochlorococcus\_marinus\_str.\_MIT\_9215
Prochlorococcus\_marinus\_str.\_MIT\_9215
Prochlorococcus\_marinus\_str.\_MIT\_9215
Prochlorococcus\_marinus\_str.\_MIT\_9215
Prochlorococcus\_marinus\_str.\_MIT\_9215
Prochlorococcus\_marinus\_str.\_MIT\_9215
Prochlorococcus\_marinus\_str.\_MIT\_9215
Prochlorococcus\_marinus\_str.\_MIT\_9215
Prochlorococcus\_marinus\_str.\_MIT\_9215
Prochlorococcus\_marinus\_str.\_MIT\_9215
Prochlorococcus\_marinus\_str.\_MIT\_9215
Prochlorococcus\_marinus\_str.\_MIT\_9215
Prochlorococcus\_marinus\_str.\_MIT\_9215
Prochlorococcus\_marinus\_str.\_MIT\_9215
Prochlorococcus\_marinus\_str.\_MIT\_9215
Prochlorococcus\_marinus\_str.\_MIT\_9215
Prochlorococcus\_marinus\_str.\_MIT\_9215
no rank

1.661.661.661.661.661.661.661.661.661.661.661.661.661.661.661.661.661.661.661.66
11111111111111111111
00000000000000000000
00000000000000000000
142479
subspecies

1.661.661.661.661.661.661.661.661.661.661.661.661.661.661.661.661.661.661.661.66
11111111111111111111
00000000000000000000
00000000000000000000
59919

Prochlorococcus\_marinus\_subsp.\_pastoris\_str.\_CCMP1986
Prochlorococcus\_marinus\_subsp.\_pastoris\_str.\_CCMP1986
Prochlorococcus\_marinus\_subsp.\_pastoris\_str.\_CCMP1986
Prochlorococcus\_marinus\_subsp.\_pastoris\_str.\_CCMP1986
Prochlorococcus\_marinus\_subsp.\_pastoris\_str.\_CCMP1986
Prochlorococcus\_marinus\_subsp.\_pastoris\_str.\_CCMP1986
Prochlorococcus\_marinus\_subsp.\_pastoris\_str.\_CCMP1986
Prochlorococcus\_marinus\_subsp.\_pastoris\_str.\_CCMP1986
Prochlorococcus\_marinus\_subsp.\_pastoris\_str.\_CCMP1986
Prochlorococcus\_marinus\_subsp.\_pastoris\_str.\_CCMP1986
Prochlorococcus\_marinus\_subsp.\_pastoris\_str.\_CCMP1986
Prochlorococcus\_marinus\_subsp.\_pastoris\_str.\_CCMP1986
Prochlorococcus\_marinus\_subsp.\_pastoris\_str.\_CCMP1986
Prochlorococcus\_marinus\_subsp.\_pastoris\_str.\_CCMP1986
Prochlorococcus\_marinus\_subsp.\_pastoris\_str.\_CCMP1986
Prochlorococcus\_marinus\_subsp.\_pastoris\_str.\_CCMP1986
Prochlorococcus\_marinus\_subsp.\_pastoris\_str.\_CCMP1986
Prochlorococcus\_marinus\_subsp.\_pastoris\_str.\_CCMP1986
Prochlorococcus\_marinus\_subsp.\_pastoris\_str.\_CCMP1986
Prochlorococcus\_marinus\_subsp.\_pastoris\_str.\_CCMP1986
no rank

1.861.861.861.861.861.861.861.861.861.861.861.861.861.861.861.861.861.861.861.86
11111111111111111111
11000000000000000000
11000000000000000000
167555

Prochlorococcus\_marinus\_str.\_NATL1A
Prochlorococcus\_marinus\_str.\_NATL1A
Prochlorococcus\_marinus\_str.\_NATL1A
Prochlorococcus\_marinus\_str.\_NATL1A
Prochlorococcus\_marinus\_str.\_NATL1A
Prochlorococcus\_marinus\_str.\_NATL1A
Prochlorococcus\_marinus\_str.\_NATL1A
Prochlorococcus\_marinus\_str.\_NATL1A
Prochlorococcus\_marinus\_str.\_NATL1A
Prochlorococcus\_marinus\_str.\_NATL1A
Prochlorococcus\_marinus\_str.\_NATL1A
Prochlorococcus\_marinus\_str.\_NATL1A
Prochlorococcus\_marinus\_str.\_NATL1A
Prochlorococcus\_marinus\_str.\_NATL1A
Prochlorococcus\_marinus\_str.\_NATL1A
Prochlorococcus\_marinus\_str.\_NATL1A
Prochlorococcus\_marinus\_str.\_NATL1A
Prochlorococcus\_marinus\_str.\_NATL1A
Prochlorococcus\_marinus\_str.\_NATL1A
Prochlorococcus\_marinus\_str.\_NATL1A
no rank

2.412.412.412.412.412.412.412.412.412.412.412.412.412.412.412.412.412.412.412.41
11111111111111111111
2.609442.386292.609442.386290000000000000000
54540000000000000000
74547

Prochlorococcus\_marinus\_str.\_MIT\_9313
Prochlorococcus\_marinus\_str.\_MIT\_9313
Prochlorococcus\_marinus\_str.\_MIT\_9313
Prochlorococcus\_marinus\_str.\_MIT\_9313
Prochlorococcus\_marinus\_str.\_MIT\_9313
Prochlorococcus\_marinus\_str.\_MIT\_9313
Prochlorococcus\_marinus\_str.\_MIT\_9313
Prochlorococcus\_marinus\_str.\_MIT\_9313
Prochlorococcus\_marinus\_str.\_MIT\_9313
Prochlorococcus\_marinus\_str.\_MIT\_9313
Prochlorococcus\_marinus\_str.\_MIT\_9313
Prochlorococcus\_marinus\_str.\_MIT\_9313
Prochlorococcus\_marinus\_str.\_MIT\_9313
Prochlorococcus\_marinus\_str.\_MIT\_9313
Prochlorococcus\_marinus\_str.\_MIT\_9313
Prochlorococcus\_marinus\_str.\_MIT\_9313
Prochlorococcus\_marinus\_str.\_MIT\_9313
Prochlorococcus\_marinus\_str.\_MIT\_9313
Prochlorococcus\_marinus\_str.\_MIT\_9313
Prochlorococcus\_marinus\_str.\_MIT\_9313
no rank

1.641.641.641.641.641.641.641.641.641.641.641.641.641.641.641.641.641.641.641.64
11111111111111111111
00000000000000000000
00000000000000000000
167546

Prochlorococcus\_marinus\_str.\_MIT\_9301
Prochlorococcus\_marinus\_str.\_MIT\_9301
Prochlorococcus\_marinus\_str.\_MIT\_9301
Prochlorococcus\_marinus\_str.\_MIT\_9301
Prochlorococcus\_marinus\_str.\_MIT\_9301
Prochlorococcus\_marinus\_str.\_MIT\_9301
Prochlorococcus\_marinus\_str.\_MIT\_9301
Prochlorococcus\_marinus\_str.\_MIT\_9301
Prochlorococcus\_marinus\_str.\_MIT\_9301
Prochlorococcus\_marinus\_str.\_MIT\_9301
Prochlorococcus\_marinus\_str.\_MIT\_9301
Prochlorococcus\_marinus\_str.\_MIT\_9301
Prochlorococcus\_marinus\_str.\_MIT\_9301
Prochlorococcus\_marinus\_str.\_MIT\_9301
Prochlorococcus\_marinus\_str.\_MIT\_9301
Prochlorococcus\_marinus\_str.\_MIT\_9301
Prochlorococcus\_marinus\_str.\_MIT\_9301
Prochlorococcus\_marinus\_str.\_MIT\_9301
Prochlorococcus\_marinus\_str.\_MIT\_9301
Prochlorococcus\_marinus\_str.\_MIT\_9301
no rank

1.711.711.711.711.711.711.711.711.711.711.711.711.711.711.711.711.711.711.711.71
11111111111111111111
00000000000000000000
00000000000000000000
74546

Prochlorococcus\_marinus\_str.\_MIT\_9312
Prochlorococcus\_marinus\_str.\_MIT\_9312
Prochlorococcus\_marinus\_str.\_MIT\_9312
Prochlorococcus\_marinus\_str.\_MIT\_9312
Prochlorococcus\_marinus\_str.\_MIT\_9312
Prochlorococcus\_marinus\_str.\_MIT\_9312
Prochlorococcus\_marinus\_str.\_MIT\_9312
Prochlorococcus\_marinus\_str.\_MIT\_9312
Prochlorococcus\_marinus\_str.\_MIT\_9312
Prochlorococcus\_marinus\_str.\_MIT\_9312
Prochlorococcus\_marinus\_str.\_MIT\_9312
Prochlorococcus\_marinus\_str.\_MIT\_9312
Prochlorococcus\_marinus\_str.\_MIT\_9312
Prochlorococcus\_marinus\_str.\_MIT\_9312
Prochlorococcus\_marinus\_str.\_MIT\_9312
Prochlorococcus\_marinus\_str.\_MIT\_9312
Prochlorococcus\_marinus\_str.\_MIT\_9312
Prochlorococcus\_marinus\_str.\_MIT\_9312
Prochlorococcus\_marinus\_str.\_MIT\_9312
Prochlorococcus\_marinus\_str.\_MIT\_9312
no rank

2.682.682.682.682.682.682.682.682.682.682.682.682.682.682.682.682.682.682.682.68
11111111111111111111
2.098612.098612.098612.098610000100000000000
33330000100000000000
59922

Prochlorococcus\_marinus\_str.\_MIT\_9303
Prochlorococcus\_marinus\_str.\_MIT\_9303
Prochlorococcus\_marinus\_str.\_MIT\_9303
Prochlorococcus\_marinus\_str.\_MIT\_9303
Prochlorococcus\_marinus\_str.\_MIT\_9303
Prochlorococcus\_marinus\_str.\_MIT\_9303
Prochlorococcus\_marinus\_str.\_MIT\_9303
Prochlorococcus\_marinus\_str.\_MIT\_9303
Prochlorococcus\_marinus\_str.\_MIT\_9303
Prochlorococcus\_marinus\_str.\_MIT\_9303
Prochlorococcus\_marinus\_str.\_MIT\_9303
Prochlorococcus\_marinus\_str.\_MIT\_9303
Prochlorococcus\_marinus\_str.\_MIT\_9303
Prochlorococcus\_marinus\_str.\_MIT\_9303
Prochlorococcus\_marinus\_str.\_MIT\_9303
Prochlorococcus\_marinus\_str.\_MIT\_9303
Prochlorococcus\_marinus\_str.\_MIT\_9303
Prochlorococcus\_marinus\_str.\_MIT\_9303
Prochlorococcus\_marinus\_str.\_MIT\_9303
Prochlorococcus\_marinus\_str.\_MIT\_9303
no rank

1.671.671.671.671.671.671.671.671.671.671.671.671.671.671.671.671.671.671.671.67
11111111111111111111
11110000000000000000
11110000000000000000
146891

Prochlorococcus\_marinus\_str.\_AS9601
Prochlorococcus\_marinus\_str.\_AS9601
Prochlorococcus\_marinus\_str.\_AS9601
Prochlorococcus\_marinus\_str.\_AS9601
Prochlorococcus\_marinus\_str.\_AS9601
Prochlorococcus\_marinus\_str.\_AS9601
Prochlorococcus\_marinus\_str.\_AS9601
Prochlorococcus\_marinus\_str.\_AS9601
Prochlorococcus\_marinus\_str.\_AS9601
Prochlorococcus\_marinus\_str.\_AS9601
Prochlorococcus\_marinus\_str.\_AS9601
Prochlorococcus\_marinus\_str.\_AS9601
Prochlorococcus\_marinus\_str.\_AS9601
Prochlorococcus\_marinus\_str.\_AS9601
Prochlorococcus\_marinus\_str.\_AS9601
Prochlorococcus\_marinus\_str.\_AS9601
Prochlorococcus\_marinus\_str.\_AS9601
Prochlorococcus\_marinus\_str.\_AS9601
Prochlorococcus\_marinus\_str.\_AS9601
Prochlorococcus\_marinus\_str.\_AS9601
no rank

1.691.691.691.691.691.691.691.691.691.691.691.691.691.691.691.691.691.691.691.69
11111111111111111111
1.693151111111111111111111
21111111111111111111
93059

Prochlorococcus\_marinus\_str.\_MIT\_9211
Prochlorococcus\_marinus\_str.\_MIT\_9211
Prochlorococcus\_marinus\_str.\_MIT\_9211
Prochlorococcus\_marinus\_str.\_MIT\_9211
Prochlorococcus\_marinus\_str.\_MIT\_9211
Prochlorococcus\_marinus\_str.\_MIT\_9211
Prochlorococcus\_marinus\_str.\_MIT\_9211
Prochlorococcus\_marinus\_str.\_MIT\_9211
Prochlorococcus\_marinus\_str.\_MIT\_9211
Prochlorococcus\_marinus\_str.\_MIT\_9211
Prochlorococcus\_marinus\_str.\_MIT\_9211
Prochlorococcus\_marinus\_str.\_MIT\_9211
Prochlorococcus\_marinus\_str.\_MIT\_9211
Prochlorococcus\_marinus\_str.\_MIT\_9211
Prochlorococcus\_marinus\_str.\_MIT\_9211
Prochlorococcus\_marinus\_str.\_MIT\_9211
Prochlorococcus\_marinus\_str.\_MIT\_9211
Prochlorococcus\_marinus\_str.\_MIT\_9211
Prochlorococcus\_marinus\_str.\_MIT\_9211
Prochlorococcus\_marinus\_str.\_MIT\_9211
no rank

1.751.751.751.751.751.751.751.751.751.751.751.751.751.751.751.751.751.751.751.75
11111111111111111111
00000000000000000000
00000000000000000000
142554
subspecies

1.751.751.751.751.751.751.751.751.751.751.751.751.751.751.751.751.751.751.751.75
11111111111111111111
00000000000000000000
00000000000000000000
167539

Prochlorococcus\_marinus\_subsp.\_marinus\_str.\_CCMP1375
Prochlorococcus\_marinus\_subsp.\_marinus\_str.\_CCMP1375
Prochlorococcus\_marinus\_subsp.\_marinus\_str.\_CCMP1375
Prochlorococcus\_marinus\_subsp.\_marinus\_str.\_CCMP1375
Prochlorococcus\_marinus\_subsp.\_marinus\_str.\_CCMP1375
Prochlorococcus\_marinus\_subsp.\_marinus\_str.\_CCMP1375
Prochlorococcus\_marinus\_subsp.\_marinus\_str.\_CCMP1375
Prochlorococcus\_marinus\_subsp.\_marinus\_str.\_CCMP1375
Prochlorococcus\_marinus\_subsp.\_marinus\_str.\_CCMP1375
Prochlorococcus\_marinus\_subsp.\_marinus\_str.\_CCMP1375
Prochlorococcus\_marinus\_subsp.\_marinus\_str.\_CCMP1375
Prochlorococcus\_marinus\_subsp.\_marinus\_str.\_CCMP1375
Prochlorococcus\_marinus\_subsp.\_marinus\_str.\_CCMP1375
Prochlorococcus\_marinus\_subsp.\_marinus\_str.\_CCMP1375
Prochlorococcus\_marinus\_subsp.\_marinus\_str.\_CCMP1375
Prochlorococcus\_marinus\_subsp.\_marinus\_str.\_CCMP1375
Prochlorococcus\_marinus\_subsp.\_marinus\_str.\_CCMP1375
Prochlorococcus\_marinus\_subsp.\_marinus\_str.\_CCMP1375
Prochlorococcus\_marinus\_subsp.\_marinus\_str.\_CCMP1375
Prochlorococcus\_marinus\_subsp.\_marinus\_str.\_CCMP1375
no rank

1.841.841.841.841.841.841.841.841.841.841.841.841.841.841.841.841.841.841.841.84
11111111111111111111
10000000000000000000
10000000000000000000
59920

Prochlorococcus\_marinus\_str.\_NATL2A
Prochlorococcus\_marinus\_str.\_NATL2A
Prochlorococcus\_marinus\_str.\_NATL2A
Prochlorococcus\_marinus\_str.\_NATL2A
Prochlorococcus\_marinus\_str.\_NATL2A
Prochlorococcus\_marinus\_str.\_NATL2A
Prochlorococcus\_marinus\_str.\_NATL2A
Prochlorococcus\_marinus\_str.\_NATL2A
Prochlorococcus\_marinus\_str.\_NATL2A
Prochlorococcus\_marinus\_str.\_NATL2A
Prochlorococcus\_marinus\_str.\_NATL2A
Prochlorococcus\_marinus\_str.\_NATL2A
Prochlorococcus\_marinus\_str.\_NATL2A
Prochlorococcus\_marinus\_str.\_NATL2A
Prochlorococcus\_marinus\_str.\_NATL2A
Prochlorococcus\_marinus\_str.\_NATL2A
Prochlorococcus\_marinus\_str.\_NATL2A
Prochlorococcus\_marinus\_str.\_NATL2A
Prochlorococcus\_marinus\_str.\_NATL2A
Prochlorococcus\_marinus\_str.\_NATL2A
no rank

1.701.701.701.701.701.701.701.701.701.701.701.701.701.701.701.701.701.701.701.70
11111111111111111111
00000000000000000000
00000000000000000000
167542

Prochlorococcus\_marinus\_str.\_MIT\_9515
Prochlorococcus\_marinus\_str.\_MIT\_9515
Prochlorococcus\_marinus\_str.\_MIT\_9515
Prochlorococcus\_marinus\_str.\_MIT\_9515
Prochlorococcus\_marinus\_str.\_MIT\_9515
Prochlorococcus\_marinus\_str.\_MIT\_9515
Prochlorococcus\_marinus\_str.\_MIT\_9515
Prochlorococcus\_marinus\_str.\_MIT\_9515
Prochlorococcus\_marinus\_str.\_MIT\_9515
Prochlorococcus\_marinus\_str.\_MIT\_9515
Prochlorococcus\_marinus\_str.\_MIT\_9515
Prochlorococcus\_marinus\_str.\_MIT\_9515
Prochlorococcus\_marinus\_str.\_MIT\_9515
Prochlorococcus\_marinus\_str.\_MIT\_9515
Prochlorococcus\_marinus\_str.\_MIT\_9515
Prochlorococcus\_marinus\_str.\_MIT\_9515
Prochlorococcus\_marinus\_str.\_MIT\_9515
Prochlorococcus\_marinus\_str.\_MIT\_9515
Prochlorococcus\_marinus\_str.\_MIT\_9515
Prochlorococcus\_marinus\_str.\_MIT\_9515
no rank

17.2217.2217.2217.2217.2217.2217.2217.2217.2217.2217.2217.2217.2217.2217.2217.2217.2217.2217.2217.22
33333333333333333333
3.500923.191112.993822.982091.492290.9332190.3885020.3885020.7102210.3885020.3885020.71022100000000
129772111111100000000
52604
order

6.696.696.696.696.696.696.696.696.696.696.696.696.696.696.696.696.696.696.696.69
11111111111111111111
3.302592.945912.945912.945911.69315111111100000000
107772111111100000000
54298
genus

6.696.696.696.696.696.696.696.696.696.696.696.696.696.696.696.696.696.696.696.69
11111111111111111111
3.302592.945912.945912.945911.69315111111100000000
107772111111100000000
54299
species

6.696.696.696.696.696.696.696.696.696.696.696.696.696.696.696.696.696.696.696.69
11111111111111111111
3.302592.945912.945912.945911.69315111111100000000
107772111111100000000
251229

Chroococcidiopsis\_thermalis\_PCC\_7203
Chroococcidiopsis\_thermalis\_PCC\_7203
Chroococcidiopsis\_thermalis\_PCC\_7203
Chroococcidiopsis\_thermalis\_PCC\_7203
Chroococcidiopsis\_thermalis\_PCC\_7203
Chroococcidiopsis\_thermalis\_PCC\_7203
Chroococcidiopsis\_thermalis\_PCC\_7203
Chroococcidiopsis\_thermalis\_PCC\_7203
Chroococcidiopsis\_thermalis\_PCC\_7203
Chroococcidiopsis\_thermalis\_PCC\_7203
Chroococcidiopsis\_thermalis\_PCC\_7203
Chroococcidiopsis\_thermalis\_PCC\_7203
Chroococcidiopsis\_thermalis\_PCC\_7203
Chroococcidiopsis\_thermalis\_PCC\_7203
Chroococcidiopsis\_thermalis\_PCC\_7203
Chroococcidiopsis\_thermalis\_PCC\_7203
Chroococcidiopsis\_thermalis\_PCC\_7203
Chroococcidiopsis\_thermalis\_PCC\_7203
Chroococcidiopsis\_thermalis\_PCC\_7203
Chroococcidiopsis\_thermalis\_PCC\_7203
no rank

5.545.545.545.545.545.545.545.545.545.545.545.545.545.545.545.545.545.545.545.54
11111111111111111111
3.833213.708053.39793.197221.693151.6931500100100000000
17151192200100100000000
102115
genus

5.545.545.545.545.545.545.545.545.545.545.545.545.545.545.545.545.545.545.545.54
11111111111111111111
3.833213.708053.39793.197221.693151.6931500100100000000
17151192200100100000000
102116
species

5.545.545.545.545.545.545.545.545.545.545.545.545.545.545.545.545.545.545.545.54
11111111111111111111
3.833213.708053.39793.197221.693151.6931500100100000000
17151192200100100000000
111780

Stanieria\_cyanosphaera\_PCC\_7437
Stanieria\_cyanosphaera\_PCC\_7437
Stanieria\_cyanosphaera\_PCC\_7437
Stanieria\_cyanosphaera\_PCC\_7437
Stanieria\_cyanosphaera\_PCC\_7437
Stanieria\_cyanosphaera\_PCC\_7437
Stanieria\_cyanosphaera\_PCC\_7437
Stanieria\_cyanosphaera\_PCC\_7437
Stanieria\_cyanosphaera\_PCC\_7437
Stanieria\_cyanosphaera\_PCC\_7437
Stanieria\_cyanosphaera\_PCC\_7437
Stanieria\_cyanosphaera\_PCC\_7437
Stanieria\_cyanosphaera\_PCC\_7437
Stanieria\_cyanosphaera\_PCC\_7437
Stanieria\_cyanosphaera\_PCC\_7437
Stanieria\_cyanosphaera\_PCC\_7437
Stanieria\_cyanosphaera\_PCC\_7437
Stanieria\_cyanosphaera\_PCC\_7437
Stanieria\_cyanosphaera\_PCC\_7437
Stanieria\_cyanosphaera\_PCC\_7437
no rank

4.994.994.994.994.994.994.994.994.994.994.994.994.994.994.994.994.994.994.994.99
11111111111111111111
3.39792.945912.609442.791761000000000000000
117561000000000000000
44474
genus

4.994.994.994.994.994.994.994.994.994.994.994.994.994.994.994.994.994.994.994.99
11111111111111111111
3.39792.945912.609442.791761000000000000000
117561000000000000000
54308
species

4.994.994.994.994.994.994.994.994.994.994.994.994.994.994.994.994.994.994.994.99
11111111111111111111
3.39792.945912.609442.791761000000000000000
117561000000000000000
118163

Pleurocapsa\_sp.\_PCC\_7327
Pleurocapsa\_sp.\_PCC\_7327
Pleurocapsa\_sp.\_PCC\_7327
Pleurocapsa\_sp.\_PCC\_7327
Pleurocapsa\_sp.\_PCC\_7327
Pleurocapsa\_sp.\_PCC\_7327
Pleurocapsa\_sp.\_PCC\_7327
Pleurocapsa\_sp.\_PCC\_7327
Pleurocapsa\_sp.\_PCC\_7327
Pleurocapsa\_sp.\_PCC\_7327
Pleurocapsa\_sp.\_PCC\_7327
Pleurocapsa\_sp.\_PCC\_7327
Pleurocapsa\_sp.\_PCC\_7327
Pleurocapsa\_sp.\_PCC\_7327
Pleurocapsa\_sp.\_PCC\_7327
Pleurocapsa\_sp.\_PCC\_7327
Pleurocapsa\_sp.\_PCC\_7327
Pleurocapsa\_sp.\_PCC\_7327
Pleurocapsa\_sp.\_PCC\_7327
Pleurocapsa\_sp.\_PCC\_7327
no rank

84.4484.4484.4484.4484.4484.4484.4484.4484.4484.4484.4484.4484.4484.4484.4484.4484.4484.4484.4484.44
1212121212121212121212121212121212121212
4.574024.303694.171694.015521.188050.7342390.8039480.6458470.912280.5028320.3994450.4584220.2598870.1525920.1525920.1525920.1525920.1525920.1525920.152592
362724201111111100000000
1161
order

61.7361.7361.7361.7361.7361.7361.7361.7361.7361.7361.7361.7361.7361.7361.7361.7361.7361.7361.7361.73
99999999999999999999
4.614284.314164.228414.060941.320510.5583160.653670.5511270.9432790.3554970.3554970.2947490.3554970.2087290.2087290.2087290.2087290.2087290.2087290.208729
372725211111111010000000
1162
family

19.3119.3119.3119.3119.3119.3119.3119.3119.3119.3119.3119.3119.3119.3119.3119.3119.3119.3119.3119.31
33333333333333333333
4.559414.313664.324794.064341.595120.2749870.4655940.4655940.465594000.27498700000000
352728212011100000000000
1163
genus

5.315.315.315.315.315.315.315.315.315.315.315.315.315.315.315.315.315.315.315.31
11111111111111111111
4.295844.044523.995733.890371.6931511.693151.693151.6931500100000000
272120182122200100000000
46234

Anabaena\_sp.\_90
Anabaena\_sp.\_90
Anabaena\_sp.\_90
Anabaena\_sp.\_90
Anabaena\_sp.\_90
Anabaena\_sp.\_90
Anabaena\_sp.\_90
Anabaena\_sp.\_90
Anabaena\_sp.\_90
Anabaena\_sp.\_90
Anabaena\_sp.\_90
Anabaena\_sp.\_90
Anabaena\_sp.\_90
Anabaena\_sp.\_90
Anabaena\_sp.\_90
Anabaena\_sp.\_90
Anabaena\_sp.\_90
Anabaena\_sp.\_90
Anabaena\_sp.\_90
Anabaena\_sp.\_90
species

6.896.896.896.896.896.896.896.896.896.896.896.896.896.896.896.896.896.896.896.89
11111111111111111111
4.737674.465744.40124.218881000000000000000
423230251000000000000000
1165
species

6.896.896.896.896.896.896.896.896.896.896.896.896.896.896.896.896.896.896.896.89
11111111111111111111
4.737674.465744.40124.218881000000000000000
423230251000000000000000
272123

Anabaena\_cylindrica\_PCC\_7122
Anabaena\_cylindrica\_PCC\_7122
Anabaena\_cylindrica\_PCC\_7122
Anabaena\_cylindrica\_PCC\_7122
Anabaena\_cylindrica\_PCC\_7122
Anabaena\_cylindrica\_PCC\_7122
Anabaena\_cylindrica\_PCC\_7122
Anabaena\_cylindrica\_PCC\_7122
Anabaena\_cylindrica\_PCC\_7122
Anabaena\_cylindrica\_PCC\_7122
Anabaena\_cylindrica\_PCC\_7122
Anabaena\_cylindrica\_PCC\_7122
Anabaena\_cylindrica\_PCC\_7122
Anabaena\_cylindrica\_PCC\_7122
Anabaena\_cylindrica\_PCC\_7122
Anabaena\_cylindrica\_PCC\_7122
Anabaena\_cylindrica\_PCC\_7122
Anabaena\_cylindrica\_PCC\_7122
Anabaena\_cylindrica\_PCC\_7122
Anabaena\_cylindrica\_PCC\_7122
no rank

7.117.117.117.117.117.117.117.117.117.117.117.117.117.117.117.117.117.117.117.11
11111111111111111111
4.583524.36734.496514.044522.09861000000000000000
362933213000000000000000
1172
species

7.117.117.117.117.117.117.117.117.117.117.117.117.117.117.117.117.117.117.117.11
11111111111111111111
4.583524.36734.496514.044522.09861000000000000000
362933213000000000000000
240292

Anabaena\_variabilis\_ATCC\_29413\_(Anabaena\_flos-aquae\_UTEX\_1444)
Anabaena\_variabilis\_ATCC\_29413\_(Anabaena\_flos-aquae\_UTEX\_1444)
Anabaena\_variabilis\_ATCC\_29413\_(Anabaena\_flos-aquae\_UTEX\_1444)
Anabaena\_variabilis\_ATCC\_29413\_(Anabaena\_flos-aquae\_UTEX\_1444)
Anabaena\_variabilis\_ATCC\_29413\_(Anabaena\_flos-aquae\_UTEX\_1444)
Anabaena\_variabilis\_ATCC\_29413\_(Anabaena\_flos-aquae\_UTEX\_1444)
Anabaena\_variabilis\_ATCC\_29413\_(Anabaena\_flos-aquae\_UTEX\_1444)
Anabaena\_variabilis\_ATCC\_29413\_(Anabaena\_flos-aquae\_UTEX\_1444)
Anabaena\_variabilis\_ATCC\_29413\_(Anabaena\_flos-aquae\_UTEX\_1444)
Anabaena\_variabilis\_ATCC\_29413\_(Anabaena\_flos-aquae\_UTEX\_1444)
Anabaena\_variabilis\_ATCC\_29413\_(Anabaena\_flos-aquae\_UTEX\_1444)
Anabaena\_variabilis\_ATCC\_29413\_(Anabaena\_flos-aquae\_UTEX\_1444)
Anabaena\_variabilis\_ATCC\_29413\_(Anabaena\_flos-aquae\_UTEX\_1444)
Anabaena\_variabilis\_ATCC\_29413\_(Anabaena\_flos-aquae\_UTEX\_1444)
Anabaena\_variabilis\_ATCC\_29413\_(Anabaena\_flos-aquae\_UTEX\_1444)
Anabaena\_variabilis\_ATCC\_29413\_(Anabaena\_flos-aquae\_UTEX\_1444)
Anabaena\_variabilis\_ATCC\_29413\_(Anabaena\_flos-aquae\_UTEX\_1444)
Anabaena\_variabilis\_ATCC\_29413\_(Anabaena\_flos-aquae\_UTEX\_1444)
Anabaena\_variabilis\_ATCC\_29413\_(Anabaena\_flos-aquae\_UTEX\_1444)
Anabaena\_variabilis\_ATCC\_29413\_(Anabaena\_flos-aquae\_UTEX\_1444)
no rank

5.495.495.495.495.495.495.495.495.495.495.495.495.495.495.495.495.495.495.495.49
11111111111111111111
5.804025.521795.317495.262680000000000000000
1229275710000000000000000
264688
genus

5.495.495.495.495.495.495.495.495.495.495.495.495.495.495.495.495.495.495.495.49
11111111111111111111
5.804025.521795.317495.262680000000000000000
1229275710000000000000000
1164
species

5.495.495.495.495.495.495.495.495.495.495.495.495.495.495.495.495.495.495.495.49
11111111111111111111
5.804025.521795.317495.262680000000000000000
1229275710000000000000000
551115

'Nostoc\_azollae'\_0708
'Nostoc\_azollae'\_0708
'Nostoc\_azollae'\_0708
'Nostoc\_azollae'\_0708
'Nostoc\_azollae'\_0708
'Nostoc\_azollae'\_0708
'Nostoc\_azollae'\_0708
'Nostoc\_azollae'\_0708
'Nostoc\_azollae'\_0708
'Nostoc\_azollae'\_0708
'Nostoc\_azollae'\_0708
'Nostoc\_azollae'\_0708
'Nostoc\_azollae'\_0708
'Nostoc\_azollae'\_0708
'Nostoc\_azollae'\_0708
'Nostoc\_azollae'\_0708
'Nostoc\_azollae'\_0708
'Nostoc\_azollae'\_0708
'Nostoc\_azollae'\_0708
'Nostoc\_azollae'\_0708
no rank

7.617.617.617.617.617.617.617.617.617.617.617.617.617.617.617.617.617.617.617.61
11111111111111111111
4.663564.465744.36734.433992.386291.693152.098612.098612.098611.693151.693151.693151.693151.693151.693151.693151.693151.693151.693151.69315
393229314233322222222222
56106
genus

7.617.617.617.617.617.617.617.617.617.617.617.617.617.617.617.617.617.617.617.61
11111111111111111111
4.663564.465744.36734.433992.386291.693152.098612.098612.098611.693151.693151.693151.693151.693151.693151.693151.693151.693151.693151.69315
393229314233322222222222
142864
species

7.617.617.617.617.617.617.617.617.617.617.617.617.617.617.617.617.617.617.617.61
11111111111111111111
4.663564.465744.36734.433992.386291.693152.098612.098612.098611.693151.693151.693151.693151.693151.693151.693151.693151.693151.693151.69315
393229314233322222222222
56107

Cylindrospermum\_stagnale\_PCC\_7417
Cylindrospermum\_stagnale\_PCC\_7417
Cylindrospermum\_stagnale\_PCC\_7417
Cylindrospermum\_stagnale\_PCC\_7417
Cylindrospermum\_stagnale\_PCC\_7417
Cylindrospermum\_stagnale\_PCC\_7417
Cylindrospermum\_stagnale\_PCC\_7417
Cylindrospermum\_stagnale\_PCC\_7417
Cylindrospermum\_stagnale\_PCC\_7417
Cylindrospermum\_stagnale\_PCC\_7417
Cylindrospermum\_stagnale\_PCC\_7417
Cylindrospermum\_stagnale\_PCC\_7417
Cylindrospermum\_stagnale\_PCC\_7417
Cylindrospermum\_stagnale\_PCC\_7417
Cylindrospermum\_stagnale\_PCC\_7417
Cylindrospermum\_stagnale\_PCC\_7417
Cylindrospermum\_stagnale\_PCC\_7417
Cylindrospermum\_stagnale\_PCC\_7417
Cylindrospermum\_stagnale\_PCC\_7417
Cylindrospermum\_stagnale\_PCC\_7417
no rank

29.3229.3229.3229.3229.3229.3229.3229.3229.3229.3229.3229.3229.3229.3229.3229.3229.3229.3229.3229.32
44444444444444444444
4.414864.049033.924953.736851.110280.5549110.5248980.3090041.134640.3090040.30900400.3090040000000
302119151111111010000000
1177
genus

6.726.726.726.726.726.726.726.726.726.726.726.726.726.726.726.726.726.726.726.72
11111111111111111111
4.637594.496514.465744.36730000000000000000
383332290000000000000000
28072

Nostoc\_sp.\_PCC\_7524
Nostoc\_sp.\_PCC\_7524
Nostoc\_sp.\_PCC\_7524
Nostoc\_sp.\_PCC\_7524
Nostoc\_sp.\_PCC\_7524
Nostoc\_sp.\_PCC\_7524
Nostoc\_sp.\_PCC\_7524
Nostoc\_sp.\_PCC\_7524
Nostoc\_sp.\_PCC\_7524
Nostoc\_sp.\_PCC\_7524
Nostoc\_sp.\_PCC\_7524
Nostoc\_sp.\_PCC\_7524
Nostoc\_sp.\_PCC\_7524
Nostoc\_sp.\_PCC\_7524
Nostoc\_sp.\_PCC\_7524
Nostoc\_sp.\_PCC\_7524
Nostoc\_sp.\_PCC\_7524
Nostoc\_sp.\_PCC\_7524
Nostoc\_sp.\_PCC\_7524
Nostoc\_sp.\_PCC\_7524
species

6.336.336.336.336.336.336.336.336.336.336.336.336.336.336.336.336.336.336.336.33
11111111111111111111
4.135493.484913.639063.397910101.6931500000000000
231214111010200000000000
317936

Nostoc\_sp.\_PCC\_7107
Nostoc\_sp.\_PCC\_7107
Nostoc\_sp.\_PCC\_7107
Nostoc\_sp.\_PCC\_7107
Nostoc\_sp.\_PCC\_7107
Nostoc\_sp.\_PCC\_7107
Nostoc\_sp.\_PCC\_7107
Nostoc\_sp.\_PCC\_7107
Nostoc\_sp.\_PCC\_7107
Nostoc\_sp.\_PCC\_7107
Nostoc\_sp.\_PCC\_7107
Nostoc\_sp.\_PCC\_7107
Nostoc\_sp.\_PCC\_7107
Nostoc\_sp.\_PCC\_7107
Nostoc\_sp.\_PCC\_7107
Nostoc\_sp.\_PCC\_7107
Nostoc\_sp.\_PCC\_7107
Nostoc\_sp.\_PCC\_7107
Nostoc\_sp.\_PCC\_7107
Nostoc\_sp.\_PCC\_7107
species

9.069.069.069.069.069.069.069.069.069.069.069.069.069.069.069.069.069.069.069.06
11111111111111111111
5.007334.496514.218884.135492.098611111.6931511010000000
553325233111211010000000
272131
species

9.069.069.069.069.069.069.069.069.069.069.069.069.069.069.069.069.069.069.069.06
11111111111111111111
5.007334.496514.218884.135492.098611111.6931511010000000
553325233111211010000000
63737

Nostoc\_punctiforme\_PCC\_73102\_(Nostoc\_punctiforme\_ATCC\_29133)
Nostoc\_punctiforme\_PCC\_73102\_(Nostoc\_punctiforme\_ATCC\_29133)
Nostoc\_punctiforme\_PCC\_73102\_(Nostoc\_punctiforme\_ATCC\_29133)
Nostoc\_punctiforme\_PCC\_73102\_(Nostoc\_punctiforme\_ATCC\_29133)
Nostoc\_punctiforme\_PCC\_73102\_(Nostoc\_punctiforme\_ATCC\_29133)
Nostoc\_punctiforme\_PCC\_73102\_(Nostoc\_punctiforme\_ATCC\_29133)
Nostoc\_punctiforme\_PCC\_73102\_(Nostoc\_punctiforme\_ATCC\_29133)
Nostoc\_punctiforme\_PCC\_73102\_(Nostoc\_punctiforme\_ATCC\_29133)
Nostoc\_punctiforme\_PCC\_73102\_(Nostoc\_punctiforme\_ATCC\_29133)
Nostoc\_punctiforme\_PCC\_73102\_(Nostoc\_punctiforme\_ATCC\_29133)
Nostoc\_punctiforme\_PCC\_73102\_(Nostoc\_punctiforme\_ATCC\_29133)
Nostoc\_punctiforme\_PCC\_73102\_(Nostoc\_punctiforme\_ATCC\_29133)
Nostoc\_punctiforme\_PCC\_73102\_(Nostoc\_punctiforme\_ATCC\_29133)
Nostoc\_punctiforme\_PCC\_73102\_(Nostoc\_punctiforme\_ATCC\_29133)
Nostoc\_punctiforme\_PCC\_73102\_(Nostoc\_punctiforme\_ATCC\_29133)
Nostoc\_punctiforme\_PCC\_73102\_(Nostoc\_punctiforme\_ATCC\_29133)
Nostoc\_punctiforme\_PCC\_73102\_(Nostoc\_punctiforme\_ATCC\_29133)
Nostoc\_punctiforme\_PCC\_73102\_(Nostoc\_punctiforme\_ATCC\_29133)
Nostoc\_punctiforme\_PCC\_73102\_(Nostoc\_punctiforme\_ATCC\_29133)
Nostoc\_punctiforme\_PCC\_73102\_(Nostoc\_punctiforme\_ATCC\_29133)
no rank

7.217.217.217.217.217.217.217.217.217.217.217.217.217.217.217.217.217.217.217.21
11111111111111111111
3.708053.564953.302592.945911100100000000000
15131071100100000000000
103690

Nostoc\_sp.\_PCC\_7120\_(Anabaena\_sp.\_PCC\_7120)
Nostoc\_sp.\_PCC\_7120\_(Anabaena\_sp.\_PCC\_7120)
Nostoc\_sp.\_PCC\_7120\_(Anabaena\_sp.\_PCC\_7120)
Nostoc\_sp.\_PCC\_7120\_(Anabaena\_sp.\_PCC\_7120)
Nostoc\_sp.\_PCC\_7120\_(Anabaena\_sp.\_PCC\_7120)
Nostoc\_sp.\_PCC\_7120\_(Anabaena\_sp.\_PCC\_7120)
Nostoc\_sp.\_PCC\_7120\_(Anabaena\_sp.\_PCC\_7120)
Nostoc\_sp.\_PCC\_7120\_(Anabaena\_sp.\_PCC\_7120)
Nostoc\_sp.\_PCC\_7120\_(Anabaena\_sp.\_PCC\_7120)
Nostoc\_sp.\_PCC\_7120\_(Anabaena\_sp.\_PCC\_7120)
Nostoc\_sp.\_PCC\_7120\_(Anabaena\_sp.\_PCC\_7120)
Nostoc\_sp.\_PCC\_7120\_(Anabaena\_sp.\_PCC\_7120)
Nostoc\_sp.\_PCC\_7120\_(Anabaena\_sp.\_PCC\_7120)
Nostoc\_sp.\_PCC\_7120\_(Anabaena\_sp.\_PCC\_7120)
Nostoc\_sp.\_PCC\_7120\_(Anabaena\_sp.\_PCC\_7120)
Nostoc\_sp.\_PCC\_7120\_(Anabaena\_sp.\_PCC\_7120)
Nostoc\_sp.\_PCC\_7120\_(Anabaena\_sp.\_PCC\_7120)
Nostoc\_sp.\_PCC\_7120\_(Anabaena\_sp.\_PCC\_7120)
Nostoc\_sp.\_PCC\_7120\_(Anabaena\_sp.\_PCC\_7120)
Nostoc\_sp.\_PCC\_7120\_(Anabaena\_sp.\_PCC\_7120)
species

22.7122.7122.7122.7122.7122.7122.7122.7122.7122.7122.7122.7122.7122.7122.7122.7122.7122.7122.7122.71
33333333333333333333
4.464574.275244.017533.892070.8280191.212431.212430.9033160.8280190.9033160.5189040.90331600000000
322620181111111100000000
1185
family

8.738.738.738.738.738.738.738.738.738.738.738.738.738.738.738.738.738.738.738.73
11111111111111111111
4.33224.295844.044523.833210111010100000000
282721170111010100000000
373984
genus

8.738.738.738.738.738.738.738.738.738.738.738.738.738.738.738.738.738.738.738.73
11111111111111111111
4.33224.295844.044523.833210111010100000000
282721170111010100000000
373994

Rivularia\_sp.\_PCC\_7116
Rivularia\_sp.\_PCC\_7116
Rivularia\_sp.\_PCC\_7116
Rivularia\_sp.\_PCC\_7116
Rivularia\_sp.\_PCC\_7116
Rivularia\_sp.\_PCC\_7116
Rivularia\_sp.\_PCC\_7116
Rivularia\_sp.\_PCC\_7116
Rivularia\_sp.\_PCC\_7116
Rivularia\_sp.\_PCC\_7116
Rivularia\_sp.\_PCC\_7116
Rivularia\_sp.\_PCC\_7116
Rivularia\_sp.\_PCC\_7116
Rivularia\_sp.\_PCC\_7116
Rivularia\_sp.\_PCC\_7116
Rivularia\_sp.\_PCC\_7116
Rivularia\_sp.\_PCC\_7116
Rivularia\_sp.\_PCC\_7116
Rivularia\_sp.\_PCC\_7116
Rivularia\_sp.\_PCC\_7116
species

13.9813.9813.9813.9813.9813.9813.9813.9813.9813.9813.9813.9813.9813.9813.9813.9813.9813.9813.9813.98
22222222222222222222
4.547224.262384.000683.928821.345091.345091.345090.842941.345090.842940.842940.8429400000000
352620191111111100000000
1186
genus

7.027.027.027.027.027.027.027.027.027.027.027.027.027.027.027.027.027.027.027.02
11111111111111111111
4.091043.890373.708053.564951110100000000000
221815131110100000000000
99598

Calothrix\_sp.\_PCC\_7507
Calothrix\_sp.\_PCC\_7507
Calothrix\_sp.\_PCC\_7507
Calothrix\_sp.\_PCC\_7507
Calothrix\_sp.\_PCC\_7507
Calothrix\_sp.\_PCC\_7507
Calothrix\_sp.\_PCC\_7507
Calothrix\_sp.\_PCC\_7507
Calothrix\_sp.\_PCC\_7507
Calothrix\_sp.\_PCC\_7507
Calothrix\_sp.\_PCC\_7507
Calothrix\_sp.\_PCC\_7507
Calothrix\_sp.\_PCC\_7507
Calothrix\_sp.\_PCC\_7507
Calothrix\_sp.\_PCC\_7507
Calothrix\_sp.\_PCC\_7507
Calothrix\_sp.\_PCC\_7507
Calothrix\_sp.\_PCC\_7507
Calothrix\_sp.\_PCC\_7507
Calothrix\_sp.\_PCC\_7507
species

6.966.966.966.966.966.966.966.966.966.966.966.966.966.966.966.966.966.966.966.96
11111111111111111111
5.007334.637594.295844.295841.693151.693151.693151.693151.693151.693151.693151.6931500000000
553827272222222200000000
32054
species

6.966.966.966.966.966.966.966.966.966.966.966.966.966.966.966.966.966.966.966.96
11111111111111111111
5.007334.637594.295844.295841.693151.693151.693151.693151.693151.693151.693151.6931500000000
553827272222222200000000
1170562

Calothrix\_sp.\_PCC\_6303
Calothrix\_sp.\_PCC\_6303
Calothrix\_sp.\_PCC\_6303
Calothrix\_sp.\_PCC\_6303
Calothrix\_sp.\_PCC\_6303
Calothrix\_sp.\_PCC\_6303
Calothrix\_sp.\_PCC\_6303
Calothrix\_sp.\_PCC\_6303
Calothrix\_sp.\_PCC\_6303
Calothrix\_sp.\_PCC\_6303
Calothrix\_sp.\_PCC\_6303
Calothrix\_sp.\_PCC\_6303
Calothrix\_sp.\_PCC\_6303
Calothrix\_sp.\_PCC\_6303
Calothrix\_sp.\_PCC\_6303
Calothrix\_sp.\_PCC\_6303
Calothrix\_sp.\_PCC\_6303
Calothrix\_sp.\_PCC\_6303
Calothrix\_sp.\_PCC\_6303
Calothrix\_sp.\_PCC\_6303
no rank

139.36139.36139.36139.36139.36139.36139.36139.36139.36139.36139.36139.36139.36139.36139.36139.36139.36139.36139.36139.36
3535353535353535353535353535353535353535
3.764883.517763.438463.388431.001340.7218170.750240.6334850.8098730.6504240.6225950.5639910.3055030.1616870.09443240.1132330.04850750.04850750.04850750.0485075
161211111111111100000000
1118
order

6.766.766.766.766.766.766.766.766.766.766.766.766.766.766.766.766.766.766.766.76
11111111111111111111
5.077544.583524.218884.218882.386292.386292.386292.098612.098612.098612.098612.3862911111111
593625254443333411111111
217161
genus

6.766.766.766.766.766.766.766.766.766.766.766.766.766.766.766.766.766.766.766.76
11111111111111111111
5.077544.583524.218884.218882.386292.386292.386292.098612.098612.098612.098612.3862911111111
593625254443333411111111
1173032
species

6.766.766.766.766.766.766.766.766.766.766.766.766.766.766.766.766.766.766.766.76
11111111111111111111
5.077544.583524.218884.218882.386292.386292.386292.098612.098612.098612.098612.3862911111111
593625254443333411111111
1173020

Chamaesiphon\_minutus\_PCC\_6605
Chamaesiphon\_minutus\_PCC\_6605
Chamaesiphon\_minutus\_PCC\_6605
Chamaesiphon\_minutus\_PCC\_6605
Chamaesiphon\_minutus\_PCC\_6605
Chamaesiphon\_minutus\_PCC\_6605
Chamaesiphon\_minutus\_PCC\_6605
Chamaesiphon\_minutus\_PCC\_6605
Chamaesiphon\_minutus\_PCC\_6605
Chamaesiphon\_minutus\_PCC\_6605
Chamaesiphon\_minutus\_PCC\_6605
Chamaesiphon\_minutus\_PCC\_6605
Chamaesiphon\_minutus\_PCC\_6605
Chamaesiphon\_minutus\_PCC\_6605
Chamaesiphon\_minutus\_PCC\_6605
Chamaesiphon\_minutus\_PCC\_6605
Chamaesiphon\_minutus\_PCC\_6605
Chamaesiphon\_minutus\_PCC\_6605
Chamaesiphon\_minutus\_PCC\_6605
Chamaesiphon\_minutus\_PCC\_6605
no rank

5.845.845.845.845.845.845.845.845.845.845.845.845.845.845.845.845.845.845.845.84
11111111111111111111
6.08765.744935.700485.605171.69315110000000000000
1621151101002110000000000000
1125
genus

5.845.845.845.845.845.845.845.845.845.845.845.845.845.845.845.845.845.845.845.84
11111111111111111111
6.08765.744935.700485.605171.69315110000000000000
1621151101002110000000000000
1126
species

5.845.845.845.845.845.845.845.845.845.845.845.845.845.845.845.845.845.845.845.84
11111111111111111111
6.08765.744935.700485.605171.69315110000000000000
1621151101002110000000000000
449447

Microcystis\_aeruginosa\_NIES-843
Microcystis\_aeruginosa\_NIES-843
Microcystis\_aeruginosa\_NIES-843
Microcystis\_aeruginosa\_NIES-843
Microcystis\_aeruginosa\_NIES-843
Microcystis\_aeruginosa\_NIES-843
Microcystis\_aeruginosa\_NIES-843
Microcystis\_aeruginosa\_NIES-843
Microcystis\_aeruginosa\_NIES-843
Microcystis\_aeruginosa\_NIES-843
Microcystis\_aeruginosa\_NIES-843
Microcystis\_aeruginosa\_NIES-843
Microcystis\_aeruginosa\_NIES-843
Microcystis\_aeruginosa\_NIES-843
Microcystis\_aeruginosa\_NIES-843
Microcystis\_aeruginosa\_NIES-843
Microcystis\_aeruginosa\_NIES-843
Microcystis\_aeruginosa\_NIES-843
Microcystis\_aeruginosa\_NIES-843
Microcystis\_aeruginosa\_NIES-843
no rank

2.592.592.592.592.592.592.592.592.592.592.592.592.592.592.592.592.592.592.592.59
11111111111111111111
3.995733.484913.639063.484910000000000000000
201214120000000000000000
146785
genus

2.592.592.592.592.592.592.592.592.592.592.592.592.592.592.592.592.592.592.592.59
11111111111111111111
3.995733.484913.639063.484910000000000000000
201214120000000000000000
146786
species

2.592.592.592.592.592.592.592.592.592.592.592.592.592.592.592.592.592.592.592.59
11111111111111111111
3.995733.484913.639063.484910000000000000000
201214120000000000000000
197221

Thermosynechococcus\_elongatus\_BP-1
Thermosynechococcus\_elongatus\_BP-1
Thermosynechococcus\_elongatus\_BP-1
Thermosynechococcus\_elongatus\_BP-1
Thermosynechococcus\_elongatus\_BP-1
Thermosynechococcus\_elongatus\_BP-1
Thermosynechococcus\_elongatus\_BP-1
Thermosynechococcus\_elongatus\_BP-1
Thermosynechococcus\_elongatus\_BP-1
Thermosynechococcus\_elongatus\_BP-1
Thermosynechococcus\_elongatus\_BP-1
Thermosynechococcus\_elongatus\_BP-1
Thermosynechococcus\_elongatus\_BP-1
Thermosynechococcus\_elongatus\_BP-1
Thermosynechococcus\_elongatus\_BP-1
Thermosynechococcus\_elongatus\_BP-1
Thermosynechococcus\_elongatus\_BP-1
Thermosynechococcus\_elongatus\_BP-1
Thermosynechococcus\_elongatus\_BP-1
Thermosynechococcus\_elongatus\_BP-1
no rank

35.2335.2335.2335.2335.2335.2335.2335.2335.2335.2335.2335.2335.2335.2335.2335.2335.2335.2335.2335.23
66666666666666666666
4.465594.272444.122074.10821.570991.051141.246881.087881.483171.287871.141370.9222680.3587850.222538000000
322623222111211110000000
43988
genus

5.795.795.795.795.795.795.795.795.795.795.795.795.795.795.795.795.795.795.795.79
11111111111111111111
4.496514.25813.944443.890370000100000000000
332619180000100000000000
395961

Cyanothece\_sp.\_PCC\_7425
Cyanothece\_sp.\_PCC\_7425
Cyanothece\_sp.\_PCC\_7425
Cyanothece\_sp.\_PCC\_7425
Cyanothece\_sp.\_PCC\_7425
Cyanothece\_sp.\_PCC\_7425
Cyanothece\_sp.\_PCC\_7425
Cyanothece\_sp.\_PCC\_7425
Cyanothece\_sp.\_PCC\_7425
Cyanothece\_sp.\_PCC\_7425
Cyanothece\_sp.\_PCC\_7425
Cyanothece\_sp.\_PCC\_7425
Cyanothece\_sp.\_PCC\_7425
Cyanothece\_sp.\_PCC\_7425
Cyanothece\_sp.\_PCC\_7425
Cyanothece\_sp.\_PCC\_7425
Cyanothece\_sp.\_PCC\_7425
Cyanothece\_sp.\_PCC\_7425
Cyanothece\_sp.\_PCC\_7425
Cyanothece\_sp.\_PCC\_7425
species

4.794.794.794.794.794.794.794.794.794.794.794.794.794.794.794.794.794.794.794.79
11111111111111111111
4.178054.091043.772593.708051000110000000000
242216151000110000000000
41431

Cyanothece\_sp.\_PCC\_8801
Cyanothece\_sp.\_PCC\_8801
Cyanothece\_sp.\_PCC\_8801
Cyanothece\_sp.\_PCC\_8801
Cyanothece\_sp.\_PCC\_8801
Cyanothece\_sp.\_PCC\_8801
Cyanothece\_sp.\_PCC\_8801
Cyanothece\_sp.\_PCC\_8801
Cyanothece\_sp.\_PCC\_8801
Cyanothece\_sp.\_PCC\_8801
Cyanothece\_sp.\_PCC\_8801
Cyanothece\_sp.\_PCC\_8801
Cyanothece\_sp.\_PCC\_8801
Cyanothece\_sp.\_PCC\_8801
Cyanothece\_sp.\_PCC\_8801
Cyanothece\_sp.\_PCC\_8801
Cyanothece\_sp.\_PCC\_8801
Cyanothece\_sp.\_PCC\_8801
Cyanothece\_sp.\_PCC\_8801
Cyanothece\_sp.\_PCC\_8801
species

7.847.847.847.847.847.847.847.847.847.847.847.847.847.847.847.847.847.847.847.84
11111111111111111111
4.76124.76124.713574.637593.079441.693152.386292.098612.098612.386292.098611.6931511000000
434341388243343211000000
497965

Cyanothece\_sp.\_PCC\_7822
Cyanothece\_sp.\_PCC\_7822
Cyanothece\_sp.\_PCC\_7822
Cyanothece\_sp.\_PCC\_7822
Cyanothece\_sp.\_PCC\_7822
Cyanothece\_sp.\_PCC\_7822
Cyanothece\_sp.\_PCC\_7822
Cyanothece\_sp.\_PCC\_7822
Cyanothece\_sp.\_PCC\_7822
Cyanothece\_sp.\_PCC\_7822
Cyanothece\_sp.\_PCC\_7822
Cyanothece\_sp.\_PCC\_7822
Cyanothece\_sp.\_PCC\_7822
Cyanothece\_sp.\_PCC\_7822
Cyanothece\_sp.\_PCC\_7822
Cyanothece\_sp.\_PCC\_7822
Cyanothece\_sp.\_PCC\_7822
Cyanothece\_sp.\_PCC\_7822
Cyanothece\_sp.\_PCC\_7822
Cyanothece\_sp.\_PCC\_7822
species

6.556.556.556.556.556.556.556.556.556.556.556.556.556.556.556.556.556.556.556.55
11111111111111111111
4.951244.713574.637594.637592.791762.386292.609442.098612.609442.098612.386291.6931500000000
524138386453534200000000
65393

Cyanothece\_sp.\_PCC\_7424
Cyanothece\_sp.\_PCC\_7424
Cyanothece\_sp.\_PCC\_7424
Cyanothece\_sp.\_PCC\_7424
Cyanothece\_sp.\_PCC\_7424
Cyanothece\_sp.\_PCC\_7424
Cyanothece\_sp.\_PCC\_7424
Cyanothece\_sp.\_PCC\_7424
Cyanothece\_sp.\_PCC\_7424
Cyanothece\_sp.\_PCC\_7424
Cyanothece\_sp.\_PCC\_7424
Cyanothece\_sp.\_PCC\_7424
Cyanothece\_sp.\_PCC\_7424
Cyanothece\_sp.\_PCC\_7424
Cyanothece\_sp.\_PCC\_7424
Cyanothece\_sp.\_PCC\_7424
Cyanothece\_sp.\_PCC\_7424
Cyanothece\_sp.\_PCC\_7424
Cyanothece\_sp.\_PCC\_7424
Cyanothece\_sp.\_PCC\_7424
species

5.465.465.465.465.465.465.465.465.465.465.465.465.465.465.465.465.465.465.465.46
11111111111111111111
4.091043.772593.639063.772590000000000000000
221614160000000000000000
43989

Cyanothece\_sp.\_ATCC\_51142
Cyanothece\_sp.\_ATCC\_51142
Cyanothece\_sp.\_ATCC\_51142
Cyanothece\_sp.\_ATCC\_51142
Cyanothece\_sp.\_ATCC\_51142
Cyanothece\_sp.\_ATCC\_51142
Cyanothece\_sp.\_ATCC\_51142
Cyanothece\_sp.\_ATCC\_51142
Cyanothece\_sp.\_ATCC\_51142
Cyanothece\_sp.\_ATCC\_51142
Cyanothece\_sp.\_ATCC\_51142
Cyanothece\_sp.\_ATCC\_51142
Cyanothece\_sp.\_ATCC\_51142
Cyanothece\_sp.\_ATCC\_51142
Cyanothece\_sp.\_ATCC\_51142
Cyanothece\_sp.\_ATCC\_51142
Cyanothece\_sp.\_ATCC\_51142
Cyanothece\_sp.\_ATCC\_51142
Cyanothece\_sp.\_ATCC\_51142
Cyanothece\_sp.\_ATCC\_51142
species

4.804.804.804.804.804.804.804.804.804.804.804.804.804.804.804.804.804.804.804.80
11111111111111111111
3.995733.639063.564953.564951.693151.693151.693151.693151.693151.693151.693151.6931510000000
201413132222222210000000
395962

Cyanothece\_sp.\_PCC\_8802
Cyanothece\_sp.\_PCC\_8802
Cyanothece\_sp.\_PCC\_8802
Cyanothece\_sp.\_PCC\_8802
Cyanothece\_sp.\_PCC\_8802
Cyanothece\_sp.\_PCC\_8802
Cyanothece\_sp.\_PCC\_8802
Cyanothece\_sp.\_PCC\_8802
Cyanothece\_sp.\_PCC\_8802
Cyanothece\_sp.\_PCC\_8802
Cyanothece\_sp.\_PCC\_8802
Cyanothece\_sp.\_PCC\_8802
Cyanothece\_sp.\_PCC\_8802
Cyanothece\_sp.\_PCC\_8802
Cyanothece\_sp.\_PCC\_8802
Cyanothece\_sp.\_PCC\_8802
Cyanothece\_sp.\_PCC\_8802
Cyanothece\_sp.\_PCC\_8802
Cyanothece\_sp.\_PCC\_8802
Cyanothece\_sp.\_PCC\_8802
species

4.184.184.184.184.184.184.184.184.184.184.184.184.184.184.184.184.184.184.184.18
11111111111111111111
4.091043.772593.708053.639061111000100000000
221615141111000100000000
92682
no rank

4.184.184.184.184.184.184.184.184.184.184.184.184.184.184.184.184.184.184.184.18
11111111111111111111
4.091043.772593.708053.639061111000100000000
221615141111000100000000
76023
genus

4.184.184.184.184.184.184.184.184.184.184.184.184.184.184.184.184.184.184.184.18
11111111111111111111
4.091043.772593.708053.639061111000100000000
221615141111000100000000
65093

Halothece\_sp.\_PCC\_7418
Halothece\_sp.\_PCC\_7418
Halothece\_sp.\_PCC\_7418
Halothece\_sp.\_PCC\_7418
Halothece\_sp.\_PCC\_7418
Halothece\_sp.\_PCC\_7418
Halothece\_sp.\_PCC\_7418
Halothece\_sp.\_PCC\_7418
Halothece\_sp.\_PCC\_7418
Halothece\_sp.\_PCC\_7418
Halothece\_sp.\_PCC\_7418
Halothece\_sp.\_PCC\_7418
Halothece\_sp.\_PCC\_7418
Halothece\_sp.\_PCC\_7418
Halothece\_sp.\_PCC\_7418
Halothece\_sp.\_PCC\_7418
Halothece\_sp.\_PCC\_7418
Halothece\_sp.\_PCC\_7418
Halothece\_sp.\_PCC\_7418
Halothece\_sp.\_PCC\_7418
species

18.1218.1218.1218.1218.1218.1218.1218.1218.1218.1218.1218.1218.1218.1218.1218.1218.1218.1218.1218.12
55555555555555555555
3.149063.10133.017673.021730.19702000000000000000
98880000000000000000
1142
genus

18.1218.1218.1218.1218.1218.1218.1218.1218.1218.1218.1218.1218.1218.1218.1218.1218.1218.1218.1218.12
55555555555555555555
3.149063.10133.017673.021730.19702000000000000000
98880000000000000000
1148

Synechocystis\_sp.\_PCC\_6803Synechocystis\_sp.\_PCC\_6803
Synechocystis\_sp.\_PCC\_6803Synechocystis\_sp.\_PCC\_6803
Synechocystis\_sp.\_PCC\_6803Synechocystis\_sp.\_PCC\_6803
Synechocystis\_sp.\_PCC\_6803Synechocystis\_sp.\_PCC\_6803
Synechocystis\_sp.\_PCC\_6803Synechocystis\_sp.\_PCC\_6803
Synechocystis\_sp.\_PCC\_6803Synechocystis\_sp.\_PCC\_6803
Synechocystis\_sp.\_PCC\_6803Synechocystis\_sp.\_PCC\_6803
Synechocystis\_sp.\_PCC\_6803Synechocystis\_sp.\_PCC\_6803
Synechocystis\_sp.\_PCC\_6803Synechocystis\_sp.\_PCC\_6803
Synechocystis\_sp.\_PCC\_6803Synechocystis\_sp.\_PCC\_6803
Synechocystis\_sp.\_PCC\_6803Synechocystis\_sp.\_PCC\_6803
Synechocystis\_sp.\_PCC\_6803Synechocystis\_sp.\_PCC\_6803
Synechocystis\_sp.\_PCC\_6803Synechocystis\_sp.\_PCC\_6803
Synechocystis\_sp.\_PCC\_6803Synechocystis\_sp.\_PCC\_6803
Synechocystis\_sp.\_PCC\_6803Synechocystis\_sp.\_PCC\_6803
Synechocystis\_sp.\_PCC\_6803Synechocystis\_sp.\_PCC\_6803
Synechocystis\_sp.\_PCC\_6803Synechocystis\_sp.\_PCC\_6803
Synechocystis\_sp.\_PCC\_6803Synechocystis\_sp.\_PCC\_6803
Synechocystis\_sp.\_PCC\_6803Synechocystis\_sp.\_PCC\_6803
Synechocystis\_sp.\_PCC\_6803Synechocystis\_sp.\_PCC\_6803
22222222222222222222
species

3.573.573.573.573.573.573.573.573.573.573.573.573.573.573.573.573.573.573.573.57
11111111111111111111
3.197223.079443.197223.079440000000000000000
98980000000000000000
1080228

Synechocystis\_sp.\_PCC\_6803\_substr.\_GT-I
Synechocystis\_sp.\_PCC\_6803\_substr.\_GT-I
Synechocystis\_sp.\_PCC\_6803\_substr.\_GT-I
Synechocystis\_sp.\_PCC\_6803\_substr.\_GT-I
Synechocystis\_sp.\_PCC\_6803\_substr.\_GT-I
Synechocystis\_sp.\_PCC\_6803\_substr.\_GT-I
Synechocystis\_sp.\_PCC\_6803\_substr.\_GT-I
Synechocystis\_sp.\_PCC\_6803\_substr.\_GT-I
Synechocystis\_sp.\_PCC\_6803\_substr.\_GT-I
Synechocystis\_sp.\_PCC\_6803\_substr.\_GT-I
Synechocystis\_sp.\_PCC\_6803\_substr.\_GT-I
Synechocystis\_sp.\_PCC\_6803\_substr.\_GT-I
Synechocystis\_sp.\_PCC\_6803\_substr.\_GT-I
Synechocystis\_sp.\_PCC\_6803\_substr.\_GT-I
Synechocystis\_sp.\_PCC\_6803\_substr.\_GT-I
Synechocystis\_sp.\_PCC\_6803\_substr.\_GT-I
Synechocystis\_sp.\_PCC\_6803\_substr.\_GT-I
Synechocystis\_sp.\_PCC\_6803\_substr.\_GT-I
Synechocystis\_sp.\_PCC\_6803\_substr.\_GT-I
Synechocystis\_sp.\_PCC\_6803\_substr.\_GT-I
no rank

3.573.573.573.573.573.573.573.573.573.573.573.573.573.573.573.573.573.573.573.57
11111111111111111111
3.197222.945913.079442.945910000000000000000
97870000000000000000
1080229

Synechocystis\_sp.\_PCC\_6803\_substr.\_PCC-N
Synechocystis\_sp.\_PCC\_6803\_substr.\_PCC-N
Synechocystis\_sp.\_PCC\_6803\_substr.\_PCC-N
Synechocystis\_sp.\_PCC\_6803\_substr.\_PCC-N
Synechocystis\_sp.\_PCC\_6803\_substr.\_PCC-N
Synechocystis\_sp.\_PCC\_6803\_substr.\_PCC-N
Synechocystis\_sp.\_PCC\_6803\_substr.\_PCC-N
Synechocystis\_sp.\_PCC\_6803\_substr.\_PCC-N
Synechocystis\_sp.\_PCC\_6803\_substr.\_PCC-N
Synechocystis\_sp.\_PCC\_6803\_substr.\_PCC-N
Synechocystis\_sp.\_PCC\_6803\_substr.\_PCC-N
Synechocystis\_sp.\_PCC\_6803\_substr.\_PCC-N
Synechocystis\_sp.\_PCC\_6803\_substr.\_PCC-N
Synechocystis\_sp.\_PCC\_6803\_substr.\_PCC-N
Synechocystis\_sp.\_PCC\_6803\_substr.\_PCC-N
Synechocystis\_sp.\_PCC\_6803\_substr.\_PCC-N
Synechocystis\_sp.\_PCC\_6803\_substr.\_PCC-N
Synechocystis\_sp.\_PCC\_6803\_substr.\_PCC-N
Synechocystis\_sp.\_PCC\_6803\_substr.\_PCC-N
Synechocystis\_sp.\_PCC\_6803\_substr.\_PCC-N
no rank

3.573.573.573.573.573.573.573.573.573.573.573.573.573.573.573.573.573.573.573.57
11111111111111111111
3.079443.079443.079443.197221000000000000000
88891000000000000000
1080230

Synechocystis\_sp.\_PCC\_6803\_substr.\_PCC-P
Synechocystis\_sp.\_PCC\_6803\_substr.\_PCC-P
Synechocystis\_sp.\_PCC\_6803\_substr.\_PCC-P
Synechocystis\_sp.\_PCC\_6803\_substr.\_PCC-P
Synechocystis\_sp.\_PCC\_6803\_substr.\_PCC-P
Synechocystis\_sp.\_PCC\_6803\_substr.\_PCC-P
Synechocystis\_sp.\_PCC\_6803\_substr.\_PCC-P
Synechocystis\_sp.\_PCC\_6803\_substr.\_PCC-P
Synechocystis\_sp.\_PCC\_6803\_substr.\_PCC-P
Synechocystis\_sp.\_PCC\_6803\_substr.\_PCC-P
Synechocystis\_sp.\_PCC\_6803\_substr.\_PCC-P
Synechocystis\_sp.\_PCC\_6803\_substr.\_PCC-P
Synechocystis\_sp.\_PCC\_6803\_substr.\_PCC-P
Synechocystis\_sp.\_PCC\_6803\_substr.\_PCC-P
Synechocystis\_sp.\_PCC\_6803\_substr.\_PCC-P
Synechocystis\_sp.\_PCC\_6803\_substr.\_PCC-P
Synechocystis\_sp.\_PCC\_6803\_substr.\_PCC-P
Synechocystis\_sp.\_PCC\_6803\_substr.\_PCC-P
Synechocystis\_sp.\_PCC\_6803\_substr.\_PCC-P
Synechocystis\_sp.\_PCC\_6803\_substr.\_PCC-P
no rank

5.885.885.885.885.885.885.885.885.885.885.885.885.885.885.885.885.885.885.885.88
11111111111111111111
2.098611.693151.693151.693150000000000000000
32220000000000000000
102231
genus

5.885.885.885.885.885.885.885.885.885.885.885.885.885.885.885.885.885.885.885.88
11111111111111111111
2.098611.693151.693151.693150000000000000000
32220000000000000000
1173026

Gloeocapsa\_sp.\_PCC\_7428
Gloeocapsa\_sp.\_PCC\_7428
Gloeocapsa\_sp.\_PCC\_7428
Gloeocapsa\_sp.\_PCC\_7428
Gloeocapsa\_sp.\_PCC\_7428
Gloeocapsa\_sp.\_PCC\_7428
Gloeocapsa\_sp.\_PCC\_7428
Gloeocapsa\_sp.\_PCC\_7428
Gloeocapsa\_sp.\_PCC\_7428
Gloeocapsa\_sp.\_PCC\_7428
Gloeocapsa\_sp.\_PCC\_7428
Gloeocapsa\_sp.\_PCC\_7428
Gloeocapsa\_sp.\_PCC\_7428
Gloeocapsa\_sp.\_PCC\_7428
Gloeocapsa\_sp.\_PCC\_7428
Gloeocapsa\_sp.\_PCC\_7428
Gloeocapsa\_sp.\_PCC\_7428
Gloeocapsa\_sp.\_PCC\_7428
Gloeocapsa\_sp.\_PCC\_7428
Gloeocapsa\_sp.\_PCC\_7428
species

8.368.368.368.368.368.368.368.368.368.368.368.368.368.368.368.368.368.368.368.36
11111111111111111111
5.890355.615125.653965.574712.945912.791762.791762.386292.609442.386292.609441.693151.693150000000
133101105977664545220000000
155977
genus

8.368.368.368.368.368.368.368.368.368.368.368.368.368.368.368.368.368.368.368.36
11111111111111111111
5.890355.615125.653965.574712.945912.791762.791762.386292.609442.386292.609441.693151.693150000000
133101105977664545220000000
155978
species

8.368.368.368.368.368.368.368.368.368.368.368.368.368.368.368.368.368.368.368.36
11111111111111111111
5.890355.615125.653965.574712.945912.791762.791762.386292.609442.386292.609441.693151.693150000000
133101105977664545220000000
329726

Acaryochloris\_marina\_MBIC11017
Acaryochloris\_marina\_MBIC11017
Acaryochloris\_marina\_MBIC11017
Acaryochloris\_marina\_MBIC11017
Acaryochloris\_marina\_MBIC11017
Acaryochloris\_marina\_MBIC11017
Acaryochloris\_marina\_MBIC11017
Acaryochloris\_marina\_MBIC11017
Acaryochloris\_marina\_MBIC11017
Acaryochloris\_marina\_MBIC11017
Acaryochloris\_marina\_MBIC11017
Acaryochloris\_marina\_MBIC11017
Acaryochloris\_marina\_MBIC11017
Acaryochloris\_marina\_MBIC11017
Acaryochloris\_marina\_MBIC11017
Acaryochloris\_marina\_MBIC11017
Acaryochloris\_marina\_MBIC11017
Acaryochloris\_marina\_MBIC11017
Acaryochloris\_marina\_MBIC11017
Acaryochloris\_marina\_MBIC11017
no rank

7.347.347.347.347.347.347.347.347.347.347.347.347.347.347.347.347.347.347.347.34
22222222222222222222
3.350072.609442.386292.222460000000000000000
105430000000000000000
102234
genus

3.163.163.163.163.163.163.163.163.163.163.163.163.163.163.163.163.163.163.163.16
11111111111111111111
3.708052.609442.386292.386290000000000000000
155440000000000000000
102235
species

3.163.163.163.163.163.163.163.163.163.163.163.163.163.163.163.163.163.163.163.16
11111111111111111111
3.708052.609442.386292.386290000000000000000
155440000000000000000
292563

Cyanobacterium\_stanieri\_PCC\_7202
Cyanobacterium\_stanieri\_PCC\_7202
Cyanobacterium\_stanieri\_PCC\_7202
Cyanobacterium\_stanieri\_PCC\_7202
Cyanobacterium\_stanieri\_PCC\_7202
Cyanobacterium\_stanieri\_PCC\_7202
Cyanobacterium\_stanieri\_PCC\_7202
Cyanobacterium\_stanieri\_PCC\_7202
Cyanobacterium\_stanieri\_PCC\_7202
Cyanobacterium\_stanieri\_PCC\_7202
Cyanobacterium\_stanieri\_PCC\_7202
Cyanobacterium\_stanieri\_PCC\_7202
Cyanobacterium\_stanieri\_PCC\_7202
Cyanobacterium\_stanieri\_PCC\_7202
Cyanobacterium\_stanieri\_PCC\_7202
Cyanobacterium\_stanieri\_PCC\_7202
Cyanobacterium\_stanieri\_PCC\_7202
Cyanobacterium\_stanieri\_PCC\_7202
Cyanobacterium\_stanieri\_PCC\_7202
Cyanobacterium\_stanieri\_PCC\_7202
no rank

4.184.184.184.184.184.184.184.184.184.184.184.184.184.184.184.184.184.184.184.18
11111111111111111111
3.079442.609442.386292.098610000000000000000
85430000000000000000
379064
species

4.184.184.184.184.184.184.184.184.184.184.184.184.184.184.184.184.184.184.184.18
11111111111111111111
3.079442.609442.386292.098610000000000000000
85430000000000000000
755178

Cyanobacterium\_aponinum\_PCC\_10605
Cyanobacterium\_aponinum\_PCC\_10605
Cyanobacterium\_aponinum\_PCC\_10605
Cyanobacterium\_aponinum\_PCC\_10605
Cyanobacterium\_aponinum\_PCC\_10605
Cyanobacterium\_aponinum\_PCC\_10605
Cyanobacterium\_aponinum\_PCC\_10605
Cyanobacterium\_aponinum\_PCC\_10605
Cyanobacterium\_aponinum\_PCC\_10605
Cyanobacterium\_aponinum\_PCC\_10605
Cyanobacterium\_aponinum\_PCC\_10605
Cyanobacterium\_aponinum\_PCC\_10605
Cyanobacterium\_aponinum\_PCC\_10605
Cyanobacterium\_aponinum\_PCC\_10605
Cyanobacterium\_aponinum\_PCC\_10605
Cyanobacterium\_aponinum\_PCC\_10605
Cyanobacterium\_aponinum\_PCC\_10605
Cyanobacterium\_aponinum\_PCC\_10605
Cyanobacterium\_aponinum\_PCC\_10605
Cyanobacterium\_aponinum\_PCC\_10605
no rank

36.5036.5036.5036.5036.5036.5036.5036.5036.5036.5036.5036.5036.5036.5036.5036.5036.5036.5036.5036.50
1313131313131313131313131313131313131313
2.596082.393332.387472.311370.2734140.0665753000.35534200000000000
54440000100000000000
1129
genus

2.932.932.932.932.932.932.932.932.932.932.932.932.932.932.932.932.932.932.932.93
11111111111111111111
4.465744.178054.091043.639060000000000000000
322422140000000000000000
321327

Synechococcus\_sp.\_JA-3-3Ab
Synechococcus\_sp.\_JA-3-3Ab
Synechococcus\_sp.\_JA-3-3Ab
Synechococcus\_sp.\_JA-3-3Ab
Synechococcus\_sp.\_JA-3-3Ab
Synechococcus\_sp.\_JA-3-3Ab
Synechococcus\_sp.\_JA-3-3Ab
Synechococcus\_sp.\_JA-3-3Ab
Synechococcus\_sp.\_JA-3-3Ab
Synechococcus\_sp.\_JA-3-3Ab
Synechococcus\_sp.\_JA-3-3Ab
Synechococcus\_sp.\_JA-3-3Ab
Synechococcus\_sp.\_JA-3-3Ab
Synechococcus\_sp.\_JA-3-3Ab
Synechococcus\_sp.\_JA-3-3Ab
Synechococcus\_sp.\_JA-3-3Ab
Synechococcus\_sp.\_JA-3-3Ab
Synechococcus\_sp.\_JA-3-3Ab
Synechococcus\_sp.\_JA-3-3Ab
Synechococcus\_sp.\_JA-3-3Ab
species

2.372.372.372.372.372.372.372.372.372.372.372.372.372.372.372.372.372.372.372.37
11111111111111111111
2.098612.098612.098612.098611000100000000000
33331000100000000000
32051

Synechococcus\_sp.\_WH\_7803
Synechococcus\_sp.\_WH\_7803
Synechococcus\_sp.\_WH\_7803
Synechococcus\_sp.\_WH\_7803
Synechococcus\_sp.\_WH\_7803
Synechococcus\_sp.\_WH\_7803
Synechococcus\_sp.\_WH\_7803
Synechococcus\_sp.\_WH\_7803
Synechococcus\_sp.\_WH\_7803
Synechococcus\_sp.\_WH\_7803
Synechococcus\_sp.\_WH\_7803
Synechococcus\_sp.\_WH\_7803
Synechococcus\_sp.\_WH\_7803
Synechococcus\_sp.\_WH\_7803
Synechococcus\_sp.\_WH\_7803
Synechococcus\_sp.\_WH\_7803
Synechococcus\_sp.\_WH\_7803
Synechococcus\_sp.\_WH\_7803
Synechococcus\_sp.\_WH\_7803
Synechococcus\_sp.\_WH\_7803
species

2.222.222.222.222.222.222.222.222.222.222.222.222.222.222.222.222.222.222.222.22
11111111111111111111
00000000000000000000
00000000000000000000
316278

Synechococcus\_sp.\_RCC307
Synechococcus\_sp.\_RCC307
Synechococcus\_sp.\_RCC307
Synechococcus\_sp.\_RCC307
Synechococcus\_sp.\_RCC307
Synechococcus\_sp.\_RCC307
Synechococcus\_sp.\_RCC307
Synechococcus\_sp.\_RCC307
Synechococcus\_sp.\_RCC307
Synechococcus\_sp.\_RCC307
Synechococcus\_sp.\_RCC307
Synechococcus\_sp.\_RCC307
Synechococcus\_sp.\_RCC307
Synechococcus\_sp.\_RCC307
Synechococcus\_sp.\_RCC307
Synechococcus\_sp.\_RCC307
Synechococcus\_sp.\_RCC307
Synechococcus\_sp.\_RCC307
Synechococcus\_sp.\_RCC307
Synechococcus\_sp.\_RCC307
species

3.583.583.583.583.583.583.583.583.583.583.583.583.583.583.583.583.583.583.583.58
11111111111111111111
3.890373.639063.484913.564950000000000000000
181412130000000000000000
1173263

Synechococcus\_sp.\_PCC\_7502
Synechococcus\_sp.\_PCC\_7502
Synechococcus\_sp.\_PCC\_7502
Synechococcus\_sp.\_PCC\_7502
Synechococcus\_sp.\_PCC\_7502
Synechococcus\_sp.\_PCC\_7502
Synechococcus\_sp.\_PCC\_7502
Synechococcus\_sp.\_PCC\_7502
Synechococcus\_sp.\_PCC\_7502
Synechococcus\_sp.\_PCC\_7502
Synechococcus\_sp.\_PCC\_7502
Synechococcus\_sp.\_PCC\_7502
Synechococcus\_sp.\_PCC\_7502
Synechococcus\_sp.\_PCC\_7502
Synechococcus\_sp.\_PCC\_7502
Synechococcus\_sp.\_PCC\_7502
Synechococcus\_sp.\_PCC\_7502
Synechococcus\_sp.\_PCC\_7502
Synechococcus\_sp.\_PCC\_7502
Synechococcus\_sp.\_PCC\_7502
species

3.413.413.413.413.413.413.413.413.413.413.413.413.413.413.413.413.413.413.413.41
11111111111111111111
3.079442.609442.791762.791760000000000000000
85660000000000000000
32049

Synechococcus\_sp.\_PCC\_7002
Synechococcus\_sp.\_PCC\_7002
Synechococcus\_sp.\_PCC\_7002
Synechococcus\_sp.\_PCC\_7002
Synechococcus\_sp.\_PCC\_7002
Synechococcus\_sp.\_PCC\_7002
Synechococcus\_sp.\_PCC\_7002
Synechococcus\_sp.\_PCC\_7002
Synechococcus\_sp.\_PCC\_7002
Synechococcus\_sp.\_PCC\_7002
Synechococcus\_sp.\_PCC\_7002
Synechococcus\_sp.\_PCC\_7002
Synechococcus\_sp.\_PCC\_7002
Synechococcus\_sp.\_PCC\_7002
Synechococcus\_sp.\_PCC\_7002
Synechococcus\_sp.\_PCC\_7002
Synechococcus\_sp.\_PCC\_7002
Synechococcus\_sp.\_PCC\_7002
Synechococcus\_sp.\_PCC\_7002
Synechococcus\_sp.\_PCC\_7002
species

3.723.723.723.723.723.723.723.723.723.723.723.723.723.723.723.723.723.723.723.72
11111111111111111111
2.609442.386292.386292.386290000000000000000
54440000000000000000
195253

Synechococcus\_sp.\_PCC\_6312
Synechococcus\_sp.\_PCC\_6312
Synechococcus\_sp.\_PCC\_6312
Synechococcus\_sp.\_PCC\_6312
Synechococcus\_sp.\_PCC\_6312
Synechococcus\_sp.\_PCC\_6312
Synechococcus\_sp.\_PCC\_6312
Synechococcus\_sp.\_PCC\_6312
Synechococcus\_sp.\_PCC\_6312
Synechococcus\_sp.\_PCC\_6312
Synechococcus\_sp.\_PCC\_6312
Synechococcus\_sp.\_PCC\_6312
Synechococcus\_sp.\_PCC\_6312
Synechococcus\_sp.\_PCC\_6312
Synechococcus\_sp.\_PCC\_6312
Synechococcus\_sp.\_PCC\_6312
Synechococcus\_sp.\_PCC\_6312
Synechococcus\_sp.\_PCC\_6312
Synechococcus\_sp.\_PCC\_6312
Synechococcus\_sp.\_PCC\_6312
species

2.432.432.432.432.432.432.432.432.432.432.432.432.432.432.432.432.432.432.432.43
11111111111111111111
2.609442.609442.609442.609442.09861100100000000000
55553100100000000000
84588

Synechococcus\_sp.\_WH\_8102
Synechococcus\_sp.\_WH\_8102
Synechococcus\_sp.\_WH\_8102
Synechococcus\_sp.\_WH\_8102
Synechococcus\_sp.\_WH\_8102
Synechococcus\_sp.\_WH\_8102
Synechococcus\_sp.\_WH\_8102
Synechococcus\_sp.\_WH\_8102
Synechococcus\_sp.\_WH\_8102
Synechococcus\_sp.\_WH\_8102
Synechococcus\_sp.\_WH\_8102
Synechococcus\_sp.\_WH\_8102
Synechococcus\_sp.\_WH\_8102
Synechococcus\_sp.\_WH\_8102
Synechococcus\_sp.\_WH\_8102
Synechococcus\_sp.\_WH\_8102
Synechococcus\_sp.\_WH\_8102
Synechococcus\_sp.\_WH\_8102
Synechococcus\_sp.\_WH\_8102
Synechococcus\_sp.\_WH\_8102
species

3.053.053.053.053.053.053.053.053.053.053.053.053.053.053.053.053.053.053.053.05
11111111111111111111
3.484912.791762.791762.791760000100000000000
126660000100000000000
321332

Synechococcus\_sp.\_JA-2-3B'a(2-13)
Synechococcus\_sp.\_JA-2-3B'a(2-13)
Synechococcus\_sp.\_JA-2-3B'a(2-13)
Synechococcus\_sp.\_JA-2-3B'a(2-13)
Synechococcus\_sp.\_JA-2-3B'a(2-13)
Synechococcus\_sp.\_JA-2-3B'a(2-13)
Synechococcus\_sp.\_JA-2-3B'a(2-13)
Synechococcus\_sp.\_JA-2-3B'a(2-13)
Synechococcus\_sp.\_JA-2-3B'a(2-13)
Synechococcus\_sp.\_JA-2-3B'a(2-13)
Synechococcus\_sp.\_JA-2-3B'a(2-13)
Synechococcus\_sp.\_JA-2-3B'a(2-13)
Synechococcus\_sp.\_JA-2-3B'a(2-13)
Synechococcus\_sp.\_JA-2-3B'a(2-13)
Synechococcus\_sp.\_JA-2-3B'a(2-13)
Synechococcus\_sp.\_JA-2-3B'a(2-13)
Synechococcus\_sp.\_JA-2-3B'a(2-13)
Synechococcus\_sp.\_JA-2-3B'a(2-13)
Synechococcus\_sp.\_JA-2-3B'a(2-13)
Synechococcus\_sp.\_JA-2-3B'a(2-13)
species

2.232.232.232.232.232.232.232.232.232.232.232.232.232.232.232.232.232.232.232.23
11111111111111111111
1.693151.693151.693151.693150000000000000000
22220000000000000000
316279

Synechococcus\_sp.\_CC9902
Synechococcus\_sp.\_CC9902
Synechococcus\_sp.\_CC9902
Synechococcus\_sp.\_CC9902
Synechococcus\_sp.\_CC9902
Synechococcus\_sp.\_CC9902
Synechococcus\_sp.\_CC9902
Synechococcus\_sp.\_CC9902
Synechococcus\_sp.\_CC9902
Synechococcus\_sp.\_CC9902
Synechococcus\_sp.\_CC9902
Synechococcus\_sp.\_CC9902
Synechococcus\_sp.\_CC9902
Synechococcus\_sp.\_CC9902
Synechococcus\_sp.\_CC9902
Synechococcus\_sp.\_CC9902
Synechococcus\_sp.\_CC9902
Synechococcus\_sp.\_CC9902
Synechococcus\_sp.\_CC9902
Synechococcus\_sp.\_CC9902
species

2.512.512.512.512.512.512.512.512.512.512.512.512.512.512.512.512.512.512.512.51
11111111111111111111
2.098612.098612.386291.693151000100000000000
33421000100000000000
110662

Synechococcus\_sp.\_CC9605
Synechococcus\_sp.\_CC9605
Synechococcus\_sp.\_CC9605
Synechococcus\_sp.\_CC9605
Synechococcus\_sp.\_CC9605
Synechococcus\_sp.\_CC9605
Synechococcus\_sp.\_CC9605
Synechococcus\_sp.\_CC9605
Synechococcus\_sp.\_CC9605
Synechococcus\_sp.\_CC9605
Synechococcus\_sp.\_CC9605
Synechococcus\_sp.\_CC9605
Synechococcus\_sp.\_CC9605
Synechococcus\_sp.\_CC9605
Synechococcus\_sp.\_CC9605
Synechococcus\_sp.\_CC9605
Synechococcus\_sp.\_CC9605
Synechococcus\_sp.\_CC9605
Synechococcus\_sp.\_CC9605
Synechococcus\_sp.\_CC9605
species

2.612.612.612.612.612.612.612.612.612.612.612.612.612.612.612.612.612.612.612.61
11111111111111111111
2.386292.386292.098612.098610000100000000000
44330000100000000000
64471

Synechococcus\_sp.\_CC9311
Synechococcus\_sp.\_CC9311
Synechococcus\_sp.\_CC9311
Synechococcus\_sp.\_CC9311
Synechococcus\_sp.\_CC9311
Synechococcus\_sp.\_CC9311
Synechococcus\_sp.\_CC9311
Synechococcus\_sp.\_CC9311
Synechococcus\_sp.\_CC9311
Synechococcus\_sp.\_CC9311
Synechococcus\_sp.\_CC9311
Synechococcus\_sp.\_CC9311
Synechococcus\_sp.\_CC9311
Synechococcus\_sp.\_CC9311
Synechococcus\_sp.\_CC9311
Synechococcus\_sp.\_CC9311
Synechococcus\_sp.\_CC9311
Synechococcus\_sp.\_CC9311
Synechococcus\_sp.\_CC9311
Synechococcus\_sp.\_CC9311
species

5.445.445.445.445.445.445.445.445.445.445.445.445.445.445.445.445.445.445.445.44
22222222222222222222
1.897371.693151.693151.693150000000000000000
22220000000000000000
32046
species

2.742.742.742.742.742.742.742.742.742.742.742.742.742.742.742.742.742.742.742.74
11111111111111111111
2.098611.693151.693151.693150000000000000000
32220000000000000000
1140

Synechococcus\_elongatus\_PCC\_7942
Synechococcus\_elongatus\_PCC\_7942
Synechococcus\_elongatus\_PCC\_7942
Synechococcus\_elongatus\_PCC\_7942
Synechococcus\_elongatus\_PCC\_7942
Synechococcus\_elongatus\_PCC\_7942
Synechococcus\_elongatus\_PCC\_7942
Synechococcus\_elongatus\_PCC\_7942
Synechococcus\_elongatus\_PCC\_7942
Synechococcus\_elongatus\_PCC\_7942
Synechococcus\_elongatus\_PCC\_7942
Synechococcus\_elongatus\_PCC\_7942
Synechococcus\_elongatus\_PCC\_7942
Synechococcus\_elongatus\_PCC\_7942
Synechococcus\_elongatus\_PCC\_7942
Synechococcus\_elongatus\_PCC\_7942
Synechococcus\_elongatus\_PCC\_7942
Synechococcus\_elongatus\_PCC\_7942
Synechococcus\_elongatus\_PCC\_7942
Synechococcus\_elongatus\_PCC\_7942
no rank

2.702.702.702.702.702.702.702.702.702.702.702.702.702.702.702.702.702.702.702.70
11111111111111111111
1.693151.693151.693151.693150000000000000000
22220000000000000000
269084

Synechococcus\_elongatus\_PCC\_6301\_(Synechococcus\_leopoliensis\_SAG
Synechococcus\_elongatus\_PCC\_6301\_(Synechococcus\_leopoliensis\_SAG
Synechococcus\_elongatus\_PCC\_6301\_(Synechococcus\_leopoliensis\_SAG
Synechococcus\_elongatus\_PCC\_6301\_(Synechococcus\_leopoliensis\_SAG
Synechococcus\_elongatus\_PCC\_6301\_(Synechococcus\_leopoliensis\_SAG
Synechococcus\_elongatus\_PCC\_6301\_(Synechococcus\_leopoliensis\_SAG
Synechococcus\_elongatus\_PCC\_6301\_(Synechococcus\_leopoliensis\_SAG
Synechococcus\_elongatus\_PCC\_6301\_(Synechococcus\_leopoliensis\_SAG
Synechococcus\_elongatus\_PCC\_6301\_(Synechococcus\_leopoliensis\_SAG
Synechococcus\_elongatus\_PCC\_6301\_(Synechococcus\_leopoliensis\_SAG
Synechococcus\_elongatus\_PCC\_6301\_(Synechococcus\_leopoliensis\_SAG
Synechococcus\_elongatus\_PCC\_6301\_(Synechococcus\_leopoliensis\_SAG
Synechococcus\_elongatus\_PCC\_6301\_(Synechococcus\_leopoliensis\_SAG
Synechococcus\_elongatus\_PCC\_6301\_(Synechococcus\_leopoliensis\_SAG
Synechococcus\_elongatus\_PCC\_6301\_(Synechococcus\_leopoliensis\_SAG
Synechococcus\_elongatus\_PCC\_6301\_(Synechococcus\_leopoliensis\_SAG
Synechococcus\_elongatus\_PCC\_6301\_(Synechococcus\_leopoliensis\_SAG
Synechococcus\_elongatus\_PCC\_6301\_(Synechococcus\_leopoliensis\_SAG
Synechococcus\_elongatus\_PCC\_6301\_(Synechococcus\_leopoliensis\_SAG
Synechococcus\_elongatus\_PCC\_6301\_(Synechococcus\_leopoliensis\_SAG
no rank

1.441.441.441.441.441.441.441.441.441.441.441.441.441.441.441.441.441.441.441.44
11111111111111111111
1.693151.693151.693151.693150000000000000000
22220000000000000000
95881
no rank

1.441.441.441.441.441.441.441.441.441.441.441.441.441.441.441.441.441.441.441.44
11111111111111111111
1.693151.693151.693151.693150000000000000000
22220000000000000000
713887

cyanobacterium\_UCYN-A
cyanobacterium\_UCYN-A
cyanobacterium\_UCYN-A
cyanobacterium\_UCYN-A
cyanobacterium\_UCYN-A
cyanobacterium\_UCYN-A
cyanobacterium\_UCYN-A
cyanobacterium\_UCYN-A
cyanobacterium\_UCYN-A
cyanobacterium\_UCYN-A
cyanobacterium\_UCYN-A
cyanobacterium\_UCYN-A
cyanobacterium\_UCYN-A
cyanobacterium\_UCYN-A
cyanobacterium\_UCYN-A
cyanobacterium\_UCYN-A
cyanobacterium\_UCYN-A
cyanobacterium\_UCYN-A
cyanobacterium\_UCYN-A
cyanobacterium\_UCYN-A
species

3.783.783.783.783.783.783.783.783.783.783.783.783.783.783.783.783.783.783.783.78
11111111111111111111
4.496514.496514.465744.465743.302593.079442.945913.079443.079442.945912.791763.079442.386292.098611.693152.386290000
3333323210878876843240000
13034
genus

3.783.783.783.783.783.783.783.783.783.783.783.783.783.783.783.783.783.783.783.78
11111111111111111111
4.496514.496514.465744.465743.302593.079442.945913.079443.079442.945912.791763.079442.386292.098611.693152.386290000
3333323210878876843240000
292566
species

3.783.783.783.783.783.783.783.783.783.783.783.783.783.783.783.783.783.783.783.78
11111111111111111111
4.496514.496514.465744.465743.302593.079442.945913.079443.079442.945912.791763.079442.386292.098611.693152.386290000
3333323210878876843240000
13035

Dactylococcopsis\_salina\_PCC\_8305
Dactylococcopsis\_salina\_PCC\_8305
Dactylococcopsis\_salina\_PCC\_8305
Dactylococcopsis\_salina\_PCC\_8305
Dactylococcopsis\_salina\_PCC\_8305
Dactylococcopsis\_salina\_PCC\_8305
Dactylococcopsis\_salina\_PCC\_8305
Dactylococcopsis\_salina\_PCC\_8305
Dactylococcopsis\_salina\_PCC\_8305
Dactylococcopsis\_salina\_PCC\_8305
Dactylococcopsis\_salina\_PCC\_8305
Dactylococcopsis\_salina\_PCC\_8305
Dactylococcopsis\_salina\_PCC\_8305
Dactylococcopsis\_salina\_PCC\_8305
Dactylococcopsis\_salina\_PCC\_8305
Dactylococcopsis\_salina\_PCC\_8305
Dactylococcopsis\_salina\_PCC\_8305
Dactylococcopsis\_salina\_PCC\_8305
Dactylococcopsis\_salina\_PCC\_8305
Dactylococcopsis\_salina\_PCC\_8305
no rank

3.343.343.343.343.343.343.343.343.343.343.343.343.343.343.343.343.343.343.343.34
11111111111111111111
3.772593.39793.39793.197221000000000000000
16111191000000000000000
167375
genus

3.343.343.343.343.343.343.343.343.343.343.343.343.343.343.343.343.343.343.343.34
11111111111111111111
3.772593.39793.39793.197221000000000000000
16111191000000000000000
59930
species

3.343.343.343.343.343.343.343.343.343.343.343.343.343.343.343.343.343.343.343.34
11111111111111111111
3.772593.39793.39793.197221000000000000000
16111191000000000000000
292564

Cyanobium\_gracile\_PCC\_6307
Cyanobium\_gracile\_PCC\_6307
Cyanobium\_gracile\_PCC\_6307
Cyanobium\_gracile\_PCC\_6307
Cyanobium\_gracile\_PCC\_6307
Cyanobium\_gracile\_PCC\_6307
Cyanobium\_gracile\_PCC\_6307
Cyanobium\_gracile\_PCC\_6307
Cyanobium\_gracile\_PCC\_6307
Cyanobium\_gracile\_PCC\_6307
Cyanobium\_gracile\_PCC\_6307
Cyanobium\_gracile\_PCC\_6307
Cyanobium\_gracile\_PCC\_6307
Cyanobium\_gracile\_PCC\_6307
Cyanobium\_gracile\_PCC\_6307
Cyanobium\_gracile\_PCC\_6307
Cyanobium\_gracile\_PCC\_6307
Cyanobium\_gracile\_PCC\_6307
Cyanobium\_gracile\_PCC\_6307
Cyanobium\_gracile\_PCC\_6307
no rank

58.1458.1458.1458.1458.1458.1458.1458.1458.1458.1458.1458.1458.1458.1458.1458.1458.1458.1458.1458.14
6565656565656565656565656565656565656565
2.65632.478532.30812.242860.8188890.782930.7562180.736930.7644940.7653090.7439040.7458640.5693810.5494540.4574390.4909960.2087430.1951550.1528640.129966
54431111111111110000
544448
phylum

58.1458.1458.1458.1458.1458.1458.1458.1458.1458.1458.1458.1458.1458.1458.1458.1458.1458.1458.1458.14
6565656565656565656565656565656565656565
2.65632.478532.30812.242860.8188890.782930.7562180.736930.7644940.7653090.7439040.7458640.5693810.5494540.4574390.4909960.2087430.1951550.1528640.129966
54431111111111110000
31969
class

0.790.790.790.790.790.790.790.790.790.790.790.790.790.790.790.790.790.790.790.79
11111111111111111111
2.098612.098612.098612.098610000000000000000
33330000000000000000
186328
order

0.790.790.790.790.790.790.790.790.790.790.790.790.790.790.790.790.790.790.790.79
11111111111111111111
2.098612.098612.098612.098610000000000000000
33330000000000000000
33925
family

0.790.790.790.790.790.790.790.790.790.790.790.790.790.790.790.790.790.790.790.79
11111111111111111111
2.098612.098612.098612.098610000000000000000
33330000000000000000
46239
genus

0.790.790.790.790.790.790.790.790.790.790.790.790.790.790.790.790.790.790.790.79
11111111111111111111
2.098612.098612.098612.098610000000000000000
33330000000000000000
2151
species

0.790.790.790.790.790.790.790.790.790.790.790.790.790.790.790.790.790.790.790.79
11111111111111111111
2.098612.098612.098612.098610000000000000000
33330000000000000000
265311

Mesoplasma\_florum\_L1
Mesoplasma\_florum\_L1
Mesoplasma\_florum\_L1
Mesoplasma\_florum\_L1
Mesoplasma\_florum\_L1
Mesoplasma\_florum\_L1
Mesoplasma\_florum\_L1
Mesoplasma\_florum\_L1
Mesoplasma\_florum\_L1
Mesoplasma\_florum\_L1
Mesoplasma\_florum\_L1
Mesoplasma\_florum\_L1
Mesoplasma\_florum\_L1
Mesoplasma\_florum\_L1
Mesoplasma\_florum\_L1
Mesoplasma\_florum\_L1
Mesoplasma\_florum\_L1
Mesoplasma\_florum\_L1
Mesoplasma\_florum\_L1
Mesoplasma\_florum\_L1
no rank

52.8052.8052.8052.8052.8052.8052.8052.8052.8052.8052.8052.8052.8052.8052.8052.8052.8052.8052.8052.80
5959595959595959595959595959595959595959
2.620392.418552.246192.172970.7531970.7305970.7118850.6951690.7118850.7264190.701260.7067630.5202110.5187540.4106760.4526520.1945150.1956520.1490840.12387
54331111111111110000
2085
order

52.8052.8052.8052.8052.8052.8052.8052.8052.8052.8052.8052.8052.8052.8052.8052.8052.8052.8052.8052.80
5959595959595959595959595959595959595959
2.620392.418552.246192.172970.7531970.7305970.7118850.6951690.7118850.7264190.701260.7067630.5202110.5187540.4106760.4526520.1945150.1956520.1490840.12387
54331111111111110000
2092
family

2.372.372.372.372.372.372.372.372.372.372.372.372.372.372.372.372.372.372.372.37
33333333333333333333
2.029511.947591.947591.947590.3670890.3670890000.3670890000000000
33331100010000000000
2129
genus

0.870.870.870.870.870.870.870.870.870.870.870.870.870.870.870.870.870.870.870.87
11111111111111111111
2.609442.386292.386292.386291100010000000000
54441100010000000000
2130
species

0.870.870.870.870.870.870.870.870.870.870.870.870.870.870.870.870.870.870.870.87
11111111111111111111
2.609442.386292.386292.386291100010000000000
54441100010000000000
95664
no rank

0.870.870.870.870.870.870.870.870.870.870.870.870.870.870.870.870.870.870.870.87
11111111111111111111
2.609442.386292.386292.386291100010000000000
54441100010000000000
565575

Ureaplasma\_urealyticum\_serovar\_10\_str.\_ATCC\_33699
Ureaplasma\_urealyticum\_serovar\_10\_str.\_ATCC\_33699
Ureaplasma\_urealyticum\_serovar\_10\_str.\_ATCC\_33699
Ureaplasma\_urealyticum\_serovar\_10\_str.\_ATCC\_33699
Ureaplasma\_urealyticum\_serovar\_10\_str.\_ATCC\_33699
Ureaplasma\_urealyticum\_serovar\_10\_str.\_ATCC\_33699
Ureaplasma\_urealyticum\_serovar\_10\_str.\_ATCC\_33699
Ureaplasma\_urealyticum\_serovar\_10\_str.\_ATCC\_33699
Ureaplasma\_urealyticum\_serovar\_10\_str.\_ATCC\_33699
Ureaplasma\_urealyticum\_serovar\_10\_str.\_ATCC\_33699
Ureaplasma\_urealyticum\_serovar\_10\_str.\_ATCC\_33699
Ureaplasma\_urealyticum\_serovar\_10\_str.\_ATCC\_33699
Ureaplasma\_urealyticum\_serovar\_10\_str.\_ATCC\_33699
Ureaplasma\_urealyticum\_serovar\_10\_str.\_ATCC\_33699
Ureaplasma\_urealyticum\_serovar\_10\_str.\_ATCC\_33699
Ureaplasma\_urealyticum\_serovar\_10\_str.\_ATCC\_33699
Ureaplasma\_urealyticum\_serovar\_10\_str.\_ATCC\_33699
Ureaplasma\_urealyticum\_serovar\_10\_str.\_ATCC\_33699
Ureaplasma\_urealyticum\_serovar\_10\_str.\_ATCC\_33699
Ureaplasma\_urealyticum\_serovar\_10\_str.\_ATCC\_33699
no rank

1.501.501.501.501.501.501.501.501.501.501.501.501.501.501.501.501.501.501.501.50
22222222222222222222
1.693151.693151.693151.693150000000000000000
22220000000000000000
134821
species

1.501.501.501.501.501.501.501.501.501.501.501.501.501.501.501.501.501.501.501.50
22222222222222222222
1.693151.693151.693151.693150000000000000000
22220000000000000000
38504
no rank

0.750.750.750.750.750.750.750.750.750.750.750.750.750.750.750.750.750.750.750.75
11111111111111111111
1.693151.693151.693151.693150000000000000000
22220000000000000000
273119

Ureaplasma\_parvum\_serovar\_3\_str.\_ATCC\_700970
Ureaplasma\_parvum\_serovar\_3\_str.\_ATCC\_700970
Ureaplasma\_parvum\_serovar\_3\_str.\_ATCC\_700970
Ureaplasma\_parvum\_serovar\_3\_str.\_ATCC\_700970
Ureaplasma\_parvum\_serovar\_3\_str.\_ATCC\_700970
Ureaplasma\_parvum\_serovar\_3\_str.\_ATCC\_700970
Ureaplasma\_parvum\_serovar\_3\_str.\_ATCC\_700970
Ureaplasma\_parvum\_serovar\_3\_str.\_ATCC\_700970
Ureaplasma\_parvum\_serovar\_3\_str.\_ATCC\_700970
Ureaplasma\_parvum\_serovar\_3\_str.\_ATCC\_700970
Ureaplasma\_parvum\_serovar\_3\_str.\_ATCC\_700970
Ureaplasma\_parvum\_serovar\_3\_str.\_ATCC\_700970
Ureaplasma\_parvum\_serovar\_3\_str.\_ATCC\_700970
Ureaplasma\_parvum\_serovar\_3\_str.\_ATCC\_700970
Ureaplasma\_parvum\_serovar\_3\_str.\_ATCC\_700970
Ureaplasma\_parvum\_serovar\_3\_str.\_ATCC\_700970
Ureaplasma\_parvum\_serovar\_3\_str.\_ATCC\_700970
Ureaplasma\_parvum\_serovar\_3\_str.\_ATCC\_700970
Ureaplasma\_parvum\_serovar\_3\_str.\_ATCC\_700970
Ureaplasma\_parvum\_serovar\_3\_str.\_ATCC\_700970
no rank

0.750.750.750.750.750.750.750.750.750.750.750.750.750.750.750.750.750.750.750.75
11111111111111111111
1.693151.693151.693151.693150000000000000000
22220000000000000000
505682

Ureaplasma\_parvum\_serovar\_3\_str.\_ATCC\_27815
Ureaplasma\_parvum\_serovar\_3\_str.\_ATCC\_27815
Ureaplasma\_parvum\_serovar\_3\_str.\_ATCC\_27815
Ureaplasma\_parvum\_serovar\_3\_str.\_ATCC\_27815
Ureaplasma\_parvum\_serovar\_3\_str.\_ATCC\_27815
Ureaplasma\_parvum\_serovar\_3\_str.\_ATCC\_27815
Ureaplasma\_parvum\_serovar\_3\_str.\_ATCC\_27815
Ureaplasma\_parvum\_serovar\_3\_str.\_ATCC\_27815
Ureaplasma\_parvum\_serovar\_3\_str.\_ATCC\_27815
Ureaplasma\_parvum\_serovar\_3\_str.\_ATCC\_27815
Ureaplasma\_parvum\_serovar\_3\_str.\_ATCC\_27815
Ureaplasma\_parvum\_serovar\_3\_str.\_ATCC\_27815
Ureaplasma\_parvum\_serovar\_3\_str.\_ATCC\_27815
Ureaplasma\_parvum\_serovar\_3\_str.\_ATCC\_27815
Ureaplasma\_parvum\_serovar\_3\_str.\_ATCC\_27815
Ureaplasma\_parvum\_serovar\_3\_str.\_ATCC\_27815
Ureaplasma\_parvum\_serovar\_3\_str.\_ATCC\_27815
Ureaplasma\_parvum\_serovar\_3\_str.\_ATCC\_27815
Ureaplasma\_parvum\_serovar\_3\_str.\_ATCC\_27815
Ureaplasma\_parvum\_serovar\_3\_str.\_ATCC\_27815
no rank

50.4350.4350.4350.4350.4350.4350.4350.4350.4350.4350.4350.4350.4350.4350.4350.4350.4350.4350.4350.43
5656565656565656565656565656565656565656
2.648162.440692.260222.183560.7713430.747680.745340.7278390.745340.7433060.7342160.7399780.5446590.5431340.4299760.4739250.2036570.2048470.156090.129691
54431111111111110000
2093
genus

0.930.930.930.930.930.930.930.930.930.930.930.930.930.930.930.930.930.930.930.93
11111111111111111111
3.079443.079442.791762.609440000000000000000
88650000000000000000
50052
species

0.930.930.930.930.930.930.930.930.930.930.930.930.930.930.930.930.930.930.930.93
11111111111111111111
3.079443.079442.791762.609440000000000000000
88650000000000000000
512564

Mycoplasma\_crocodyli\_MP145
Mycoplasma\_crocodyli\_MP145
Mycoplasma\_crocodyli\_MP145
Mycoplasma\_crocodyli\_MP145
Mycoplasma\_crocodyli\_MP145
Mycoplasma\_crocodyli\_MP145
Mycoplasma\_crocodyli\_MP145
Mycoplasma\_crocodyli\_MP145
Mycoplasma\_crocodyli\_MP145
Mycoplasma\_crocodyli\_MP145
Mycoplasma\_crocodyli\_MP145
Mycoplasma\_crocodyli\_MP145
Mycoplasma\_crocodyli\_MP145
Mycoplasma\_crocodyli\_MP145
Mycoplasma\_crocodyli\_MP145
Mycoplasma\_crocodyli\_MP145
Mycoplasma\_crocodyli\_MP145
Mycoplasma\_crocodyli\_MP145
Mycoplasma\_crocodyli\_MP145
Mycoplasma\_crocodyli\_MP145
no rank

0.780.780.780.780.780.780.780.780.780.780.780.780.780.780.780.780.780.780.780.78
11111111111111111111
1.693151.693151.693151.693151110111100000000
22221110111100000000
2118
species

0.780.780.780.780.780.780.780.780.780.780.780.780.780.780.780.780.780.780.780.78
11111111111111111111
1.693151.693151.693151.693151110111100000000
22221110111100000000
267748

Mycoplasma\_mobile\_163K
Mycoplasma\_mobile\_163K
Mycoplasma\_mobile\_163K
Mycoplasma\_mobile\_163K
Mycoplasma\_mobile\_163K
Mycoplasma\_mobile\_163K
Mycoplasma\_mobile\_163K
Mycoplasma\_mobile\_163K
Mycoplasma\_mobile\_163K
Mycoplasma\_mobile\_163K
Mycoplasma\_mobile\_163K
Mycoplasma\_mobile\_163K
Mycoplasma\_mobile\_163K
Mycoplasma\_mobile\_163K
Mycoplasma\_mobile\_163K
Mycoplasma\_mobile\_163K
Mycoplasma\_mobile\_163K
Mycoplasma\_mobile\_163K
Mycoplasma\_mobile\_163K
Mycoplasma\_mobile\_163K
no rank

3.353.353.353.353.353.353.353.353.353.353.353.353.353.353.353.353.353.353.353.35
44444444444444444444
3.244112.251591.02291.094790000000000000000
93110000000000000000
2100
species

0.840.840.840.840.840.840.840.840.840.840.840.840.840.840.840.840.840.840.840.84
11111111111111111111
3.197222.60944000000000000000000
95000000000000000000
1118964

Mycoplasma\_hyorhinis\_SK76
Mycoplasma\_hyorhinis\_SK76
Mycoplasma\_hyorhinis\_SK76
Mycoplasma\_hyorhinis\_SK76
Mycoplasma\_hyorhinis\_SK76
Mycoplasma\_hyorhinis\_SK76
Mycoplasma\_hyorhinis\_SK76
Mycoplasma\_hyorhinis\_SK76
Mycoplasma\_hyorhinis\_SK76
Mycoplasma\_hyorhinis\_SK76
Mycoplasma\_hyorhinis\_SK76
Mycoplasma\_hyorhinis\_SK76
Mycoplasma\_hyorhinis\_SK76
Mycoplasma\_hyorhinis\_SK76
Mycoplasma\_hyorhinis\_SK76
Mycoplasma\_hyorhinis\_SK76
Mycoplasma\_hyorhinis\_SK76
Mycoplasma\_hyorhinis\_SK76
Mycoplasma\_hyorhinis\_SK76
Mycoplasma\_hyorhinis\_SK76
no rank

0.840.840.840.840.840.840.840.840.840.840.840.840.840.840.840.840.840.840.840.84
11111111111111111111
3.079441.693151.693151.693150000000000000000
82220000000000000000
1129369

Mycoplasma\_hyorhinis\_GDL-1
Mycoplasma\_hyorhinis\_GDL-1
Mycoplasma\_hyorhinis\_GDL-1
Mycoplasma\_hyorhinis\_GDL-1
Mycoplasma\_hyorhinis\_GDL-1
Mycoplasma\_hyorhinis\_GDL-1
Mycoplasma\_hyorhinis\_GDL-1
Mycoplasma\_hyorhinis\_GDL-1
Mycoplasma\_hyorhinis\_GDL-1
Mycoplasma\_hyorhinis\_GDL-1
Mycoplasma\_hyorhinis\_GDL-1
Mycoplasma\_hyorhinis\_GDL-1
Mycoplasma\_hyorhinis\_GDL-1
Mycoplasma\_hyorhinis\_GDL-1
Mycoplasma\_hyorhinis\_GDL-1
Mycoplasma\_hyorhinis\_GDL-1
Mycoplasma\_hyorhinis\_GDL-1
Mycoplasma\_hyorhinis\_GDL-1
Mycoplasma\_hyorhinis\_GDL-1
Mycoplasma\_hyorhinis\_GDL-1
no rank

0.840.840.840.840.840.840.840.840.840.840.840.840.840.840.840.840.840.840.840.84
11111111111111111111
3.39792.098612.3862910000000000000000
113410000000000000000
872331

Mycoplasma\_hyorhinis\_HUB-1
Mycoplasma\_hyorhinis\_HUB-1
Mycoplasma\_hyorhinis\_HUB-1
Mycoplasma\_hyorhinis\_HUB-1
Mycoplasma\_hyorhinis\_HUB-1
Mycoplasma\_hyorhinis\_HUB-1
Mycoplasma\_hyorhinis\_HUB-1
Mycoplasma\_hyorhinis\_HUB-1
Mycoplasma\_hyorhinis\_HUB-1
Mycoplasma\_hyorhinis\_HUB-1
Mycoplasma\_hyorhinis\_HUB-1
Mycoplasma\_hyorhinis\_HUB-1
Mycoplasma\_hyorhinis\_HUB-1
Mycoplasma\_hyorhinis\_HUB-1
Mycoplasma\_hyorhinis\_HUB-1
Mycoplasma\_hyorhinis\_HUB-1
Mycoplasma\_hyorhinis\_HUB-1
Mycoplasma\_hyorhinis\_HUB-1
Mycoplasma\_hyorhinis\_HUB-1
Mycoplasma\_hyorhinis\_HUB-1
no rank

0.830.830.830.830.830.830.830.830.830.830.830.830.830.830.830.830.830.830.830.83
11111111111111111111
3.302592.6094401.693150000000000000000
105020000000000000000
936139

Mycoplasma\_hyorhinis\_MCLD
Mycoplasma\_hyorhinis\_MCLD
Mycoplasma\_hyorhinis\_MCLD
Mycoplasma\_hyorhinis\_MCLD
Mycoplasma\_hyorhinis\_MCLD
Mycoplasma\_hyorhinis\_MCLD
Mycoplasma\_hyorhinis\_MCLD
Mycoplasma\_hyorhinis\_MCLD
Mycoplasma\_hyorhinis\_MCLD
Mycoplasma\_hyorhinis\_MCLD
Mycoplasma\_hyorhinis\_MCLD
Mycoplasma\_hyorhinis\_MCLD
Mycoplasma\_hyorhinis\_MCLD
Mycoplasma\_hyorhinis\_MCLD
Mycoplasma\_hyorhinis\_MCLD
Mycoplasma\_hyorhinis\_MCLD
Mycoplasma\_hyorhinis\_MCLD
Mycoplasma\_hyorhinis\_MCLD
Mycoplasma\_hyorhinis\_MCLD
Mycoplasma\_hyorhinis\_MCLD
no rank

0.820.820.820.820.820.820.820.820.820.820.820.820.820.820.820.820.820.820.820.82
11111111111111111111
1.693151.693151.693151.693150000000000000000
22220000000000000000
2111
species

0.820.820.820.820.820.820.820.820.820.820.820.820.820.820.820.820.820.820.820.82
11111111111111111111
1.693151.693151.693151.693150000000000000000
22220000000000000000
243272

Mycoplasma\_arthritidis\_158L3-1
Mycoplasma\_arthritidis\_158L3-1
Mycoplasma\_arthritidis\_158L3-1
Mycoplasma\_arthritidis\_158L3-1
Mycoplasma\_arthritidis\_158L3-1
Mycoplasma\_arthritidis\_158L3-1
Mycoplasma\_arthritidis\_158L3-1
Mycoplasma\_arthritidis\_158L3-1
Mycoplasma\_arthritidis\_158L3-1
Mycoplasma\_arthritidis\_158L3-1
Mycoplasma\_arthritidis\_158L3-1
Mycoplasma\_arthritidis\_158L3-1
Mycoplasma\_arthritidis\_158L3-1
Mycoplasma\_arthritidis\_158L3-1
Mycoplasma\_arthritidis\_158L3-1
Mycoplasma\_arthritidis\_158L3-1
Mycoplasma\_arthritidis\_158L3-1
Mycoplasma\_arthritidis\_158L3-1
Mycoplasma\_arthritidis\_158L3-1
Mycoplasma\_arthritidis\_158L3-1
no rank

1.361.361.361.361.361.361.361.361.361.361.361.361.361.361.361.361.361.361.361.36
11111111111111111111
3.39793.079442.945912.945910000000000000000
118770000000000000000
28227
species

1.361.361.361.361.361.361.361.361.361.361.361.361.361.361.361.361.361.361.361.36
11111111111111111111
3.39793.079442.945912.945910000000000000000
118770000000000000000
272633

Mycoplasma\_penetrans\_HF-2
Mycoplasma\_penetrans\_HF-2
Mycoplasma\_penetrans\_HF-2
Mycoplasma\_penetrans\_HF-2
Mycoplasma\_penetrans\_HF-2
Mycoplasma\_penetrans\_HF-2
Mycoplasma\_penetrans\_HF-2
Mycoplasma\_penetrans\_HF-2
Mycoplasma\_penetrans\_HF-2
Mycoplasma\_penetrans\_HF-2
Mycoplasma\_penetrans\_HF-2
Mycoplasma\_penetrans\_HF-2
Mycoplasma\_penetrans\_HF-2
Mycoplasma\_penetrans\_HF-2
Mycoplasma\_penetrans\_HF-2
Mycoplasma\_penetrans\_HF-2
Mycoplasma\_penetrans\_HF-2
Mycoplasma\_penetrans\_HF-2
Mycoplasma\_penetrans\_HF-2
Mycoplasma\_penetrans\_HF-2
no rank

0.650.650.650.650.650.650.650.650.650.650.650.650.650.650.650.650.650.650.650.65
11111111111111111111
11110000000000000000
11110000000000000000
65123
species

0.650.650.650.650.650.650.650.650.650.650.650.650.650.650.650.650.650.650.650.65
11111111111111111111
11110000000000000000
11110000000000000000
1197325

Mycoplasma\_wenyonii\_str.\_Massachusetts
Mycoplasma\_wenyonii\_str.\_Massachusetts
Mycoplasma\_wenyonii\_str.\_Massachusetts
Mycoplasma\_wenyonii\_str.\_Massachusetts
Mycoplasma\_wenyonii\_str.\_Massachusetts
Mycoplasma\_wenyonii\_str.\_Massachusetts
Mycoplasma\_wenyonii\_str.\_Massachusetts
Mycoplasma\_wenyonii\_str.\_Massachusetts
Mycoplasma\_wenyonii\_str.\_Massachusetts
Mycoplasma\_wenyonii\_str.\_Massachusetts
Mycoplasma\_wenyonii\_str.\_Massachusetts
Mycoplasma\_wenyonii\_str.\_Massachusetts
Mycoplasma\_wenyonii\_str.\_Massachusetts
Mycoplasma\_wenyonii\_str.\_Massachusetts
Mycoplasma\_wenyonii\_str.\_Massachusetts
Mycoplasma\_wenyonii\_str.\_Massachusetts
Mycoplasma\_wenyonii\_str.\_Massachusetts
Mycoplasma\_wenyonii\_str.\_Massachusetts
Mycoplasma\_wenyonii\_str.\_Massachusetts
Mycoplasma\_wenyonii\_str.\_Massachusetts
no rank

2.452.452.452.452.452.452.452.452.452.452.452.452.452.452.452.452.452.452.452.45
33333333333333333333
1.599691.6931510.6693880000000000000000
22110000000000000000
2104
species

0.820.820.820.820.820.820.820.820.820.820.820.820.820.820.820.820.820.820.820.82
11111111111111111111
1.693151.69315110000000000000000
22110000000000000000
272634

Mycoplasma\_pneumoniae\_M129
Mycoplasma\_pneumoniae\_M129
Mycoplasma\_pneumoniae\_M129
Mycoplasma\_pneumoniae\_M129
Mycoplasma\_pneumoniae\_M129
Mycoplasma\_pneumoniae\_M129
Mycoplasma\_pneumoniae\_M129
Mycoplasma\_pneumoniae\_M129
Mycoplasma\_pneumoniae\_M129
Mycoplasma\_pneumoniae\_M129
Mycoplasma\_pneumoniae\_M129
Mycoplasma\_pneumoniae\_M129
Mycoplasma\_pneumoniae\_M129
Mycoplasma\_pneumoniae\_M129
Mycoplasma\_pneumoniae\_M129
Mycoplasma\_pneumoniae\_M129
Mycoplasma\_pneumoniae\_M129
Mycoplasma\_pneumoniae\_M129
Mycoplasma\_pneumoniae\_M129
Mycoplasma\_pneumoniae\_M129
no rank

0.820.820.820.820.820.820.820.820.820.820.820.820.820.820.820.820.820.820.820.82
11111111111111111111
2.098611.69315110000000000000000
32110000000000000000
1112856

Mycoplasma\_pneumoniae\_309
Mycoplasma\_pneumoniae\_309
Mycoplasma\_pneumoniae\_309
Mycoplasma\_pneumoniae\_309
Mycoplasma\_pneumoniae\_309
Mycoplasma\_pneumoniae\_309
Mycoplasma\_pneumoniae\_309
Mycoplasma\_pneumoniae\_309
Mycoplasma\_pneumoniae\_309
Mycoplasma\_pneumoniae\_309
Mycoplasma\_pneumoniae\_309
Mycoplasma\_pneumoniae\_309
Mycoplasma\_pneumoniae\_309
Mycoplasma\_pneumoniae\_309
Mycoplasma\_pneumoniae\_309
Mycoplasma\_pneumoniae\_309
Mycoplasma\_pneumoniae\_309
Mycoplasma\_pneumoniae\_309
Mycoplasma\_pneumoniae\_309
Mycoplasma\_pneumoniae\_309
no rank

0.810.810.810.810.810.810.810.810.810.810.810.810.810.810.810.810.810.810.810.81
11111111111111111111
11.69315100000000000000000
12100000000000000000
722438

Mycoplasma\_pneumoniae\_FH
Mycoplasma\_pneumoniae\_FH
Mycoplasma\_pneumoniae\_FH
Mycoplasma\_pneumoniae\_FH
Mycoplasma\_pneumoniae\_FH
Mycoplasma\_pneumoniae\_FH
Mycoplasma\_pneumoniae\_FH
Mycoplasma\_pneumoniae\_FH
Mycoplasma\_pneumoniae\_FH
Mycoplasma\_pneumoniae\_FH
Mycoplasma\_pneumoniae\_FH
Mycoplasma\_pneumoniae\_FH
Mycoplasma\_pneumoniae\_FH
Mycoplasma\_pneumoniae\_FH
Mycoplasma\_pneumoniae\_FH
Mycoplasma\_pneumoniae\_FH
Mycoplasma\_pneumoniae\_FH
Mycoplasma\_pneumoniae\_FH
Mycoplasma\_pneumoniae\_FH
Mycoplasma\_pneumoniae\_FH
no rank

0.760.760.760.760.760.760.760.760.760.760.760.760.760.760.760.760.760.760.760.76
11111111111111111111
1.693151.693151.693151.693150000000000000000
22220000000000000000
141391
species

0.760.760.760.760.760.760.760.760.760.760.760.760.760.760.760.760.760.760.760.76
11111111111111111111
1.693151.693151.693151.693150000000000000000
22220000000000000000
1212765

Candidatus\_Mycoplasma\_haemolamae\_str.\_Purdue
Candidatus\_Mycoplasma\_haemolamae\_str.\_Purdue
Candidatus\_Mycoplasma\_haemolamae\_str.\_Purdue
Candidatus\_Mycoplasma\_haemolamae\_str.\_Purdue
Candidatus\_Mycoplasma\_haemolamae\_str.\_Purdue
Candidatus\_Mycoplasma\_haemolamae\_str.\_Purdue
Candidatus\_Mycoplasma\_haemolamae\_str.\_Purdue
Candidatus\_Mycoplasma\_haemolamae\_str.\_Purdue
Candidatus\_Mycoplasma\_haemolamae\_str.\_Purdue
Candidatus\_Mycoplasma\_haemolamae\_str.\_Purdue
Candidatus\_Mycoplasma\_haemolamae\_str.\_Purdue
Candidatus\_Mycoplasma\_haemolamae\_str.\_Purdue
Candidatus\_Mycoplasma\_haemolamae\_str.\_Purdue
Candidatus\_Mycoplasma\_haemolamae\_str.\_Purdue
Candidatus\_Mycoplasma\_haemolamae\_str.\_Purdue
Candidatus\_Mycoplasma\_haemolamae\_str.\_Purdue
Candidatus\_Mycoplasma\_haemolamae\_str.\_Purdue
Candidatus\_Mycoplasma\_haemolamae\_str.\_Purdue
Candidatus\_Mycoplasma\_haemolamae\_str.\_Purdue
Candidatus\_Mycoplasma\_haemolamae\_str.\_Purdue
no rank

5.415.415.415.415.415.415.415.415.415.415.415.415.415.415.415.415.415.415.415.41
55555555555555555555
3.256063.058813.030612.991821.479431.479431.479431.438651.479431.438651.479431.479431.388741.32441.32441.388740.8706720.8706720.930060.870672
108872222222211111111
656088
species group

1.011.011.011.011.011.011.011.011.011.011.011.011.011.011.011.011.011.011.011.01
11111111111111111111
1.693151.693151.693151.693150000000000000000
22220000000000000000
2095
species

1.011.011.011.011.011.011.011.011.011.011.011.011.011.011.011.011.011.011.011.01
11111111111111111111
1.693151.693151.693151.693150000000000000000
22220000000000000000
40479
subspecies

1.011.011.011.011.011.011.011.011.011.011.011.011.011.011.011.011.011.011.011.01
11111111111111111111
1.693151.693151.693151.693150000000000000000
22220000000000000000
340047

Mycoplasma\_capricolum\_subsp.\_capricolum\_ATCC\_27343
Mycoplasma\_capricolum\_subsp.\_capricolum\_ATCC\_27343
Mycoplasma\_capricolum\_subsp.\_capricolum\_ATCC\_27343
Mycoplasma\_capricolum\_subsp.\_capricolum\_ATCC\_27343
Mycoplasma\_capricolum\_subsp.\_capricolum\_ATCC\_27343
Mycoplasma\_capricolum\_subsp.\_capricolum\_ATCC\_27343
Mycoplasma\_capricolum\_subsp.\_capricolum\_ATCC\_27343
Mycoplasma\_capricolum\_subsp.\_capricolum\_ATCC\_27343
Mycoplasma\_capricolum\_subsp.\_capricolum\_ATCC\_27343
Mycoplasma\_capricolum\_subsp.\_capricolum\_ATCC\_27343
Mycoplasma\_capricolum\_subsp.\_capricolum\_ATCC\_27343
Mycoplasma\_capricolum\_subsp.\_capricolum\_ATCC\_27343
Mycoplasma\_capricolum\_subsp.\_capricolum\_ATCC\_27343
Mycoplasma\_capricolum\_subsp.\_capricolum\_ATCC\_27343
Mycoplasma\_capricolum\_subsp.\_capricolum\_ATCC\_27343
Mycoplasma\_capricolum\_subsp.\_capricolum\_ATCC\_27343
Mycoplasma\_capricolum\_subsp.\_capricolum\_ATCC\_27343
Mycoplasma\_capricolum\_subsp.\_capricolum\_ATCC\_27343
Mycoplasma\_capricolum\_subsp.\_capricolum\_ATCC\_27343
Mycoplasma\_capricolum\_subsp.\_capricolum\_ATCC\_27343
no rank

2.032.032.032.032.032.032.032.032.032.032.032.032.032.032.032.032.032.032.032.03
22222222222222222222
2.751452.591442.650622.591441.311151.311151.311151.311151.311151.311151.311151.311151.311151.311151.311151.311151.311151.311151.311151.31115
65551111111111111111
2105
species

1.021.021.021.021.021.021.021.021.021.021.021.021.021.021.021.021.021.021.021.02
11111111111111111111
3.39793.079443.197223.079442.609442.609442.609442.609442.609442.609442.609442.609442.609442.609442.609442.609442.609442.609442.609442.60944
118985555555555555555
866629

Mycoplasma\_leachii\_99\_014\_6
Mycoplasma\_leachii\_99\_014\_6
Mycoplasma\_leachii\_99\_014\_6
Mycoplasma\_leachii\_99\_014\_6
Mycoplasma\_leachii\_99\_014\_6
Mycoplasma\_leachii\_99\_014\_6
Mycoplasma\_leachii\_99\_014\_6
Mycoplasma\_leachii\_99\_014\_6
Mycoplasma\_leachii\_99\_014\_6
Mycoplasma\_leachii\_99\_014\_6
Mycoplasma\_leachii\_99\_014\_6
Mycoplasma\_leachii\_99\_014\_6
Mycoplasma\_leachii\_99\_014\_6
Mycoplasma\_leachii\_99\_014\_6
Mycoplasma\_leachii\_99\_014\_6
Mycoplasma\_leachii\_99\_014\_6
Mycoplasma\_leachii\_99\_014\_6
Mycoplasma\_leachii\_99\_014\_6
Mycoplasma\_leachii\_99\_014\_6
Mycoplasma\_leachii\_99\_014\_6
no rank

1.011.011.011.011.011.011.011.011.011.011.011.011.011.011.011.011.011.011.011.01
11111111111111111111
2.098612.098612.098612.098610000000000000000
33330000000000000000
880447

Mycoplasma\_leachii\_PG50
Mycoplasma\_leachii\_PG50
Mycoplasma\_leachii\_PG50
Mycoplasma\_leachii\_PG50
Mycoplasma\_leachii\_PG50
Mycoplasma\_leachii\_PG50
Mycoplasma\_leachii\_PG50
Mycoplasma\_leachii\_PG50
Mycoplasma\_leachii\_PG50
Mycoplasma\_leachii\_PG50
Mycoplasma\_leachii\_PG50
Mycoplasma\_leachii\_PG50
Mycoplasma\_leachii\_PG50
Mycoplasma\_leachii\_PG50
Mycoplasma\_leachii\_PG50
Mycoplasma\_leachii\_PG50
Mycoplasma\_leachii\_PG50
Mycoplasma\_leachii\_PG50
Mycoplasma\_leachii\_PG50
Mycoplasma\_leachii\_PG50
no rank

2.372.372.372.372.372.372.372.372.372.372.372.372.372.372.372.372.372.372.372.37
22222222222222222222
4.354334.041123.926053.888212.254042.254042.254042.160962.254042.160962.254042.254042.047031.900161.900162.047030.8644340.86443410.864434
292119184443434432231111
2102
species

1.161.161.161.161.161.161.161.161.161.161.161.161.161.161.161.161.161.161.161.16
11111111111111111111
3.772593.197223.079443.079441.693151.693151.693151.693151.693151.693151.693151.693151.693151.693151.693151.693150010
169882222222222220010
40477
subspecies

1.161.161.161.161.161.161.161.161.161.161.161.161.161.161.161.161.161.161.161.16
11111111111111111111
3.772593.197223.079443.079441.693151.693151.693151.693151.693151.693151.693151.693151.693151.693151.693151.693150010
169882222222222220010
44100
no rank

1.161.161.161.161.161.161.161.161.161.161.161.161.161.161.161.161.161.161.161.16
11111111111111111111
3.772593.197223.079443.079441.693151.693151.693151.693151.693151.693151.693151.693151.693151.693151.693151.693150010
169882222222222220010
862259

Mycoplasma\_mycoides\_subsp.\_capri\_LC\_str.\_95010
Mycoplasma\_mycoides\_subsp.\_capri\_LC\_str.\_95010
Mycoplasma\_mycoides\_subsp.\_capri\_LC\_str.\_95010
Mycoplasma\_mycoides\_subsp.\_capri\_LC\_str.\_95010
Mycoplasma\_mycoides\_subsp.\_capri\_LC\_str.\_95010
Mycoplasma\_mycoides\_subsp.\_capri\_LC\_str.\_95010
Mycoplasma\_mycoides\_subsp.\_capri\_LC\_str.\_95010
Mycoplasma\_mycoides\_subsp.\_capri\_LC\_str.\_95010
Mycoplasma\_mycoides\_subsp.\_capri\_LC\_str.\_95010
Mycoplasma\_mycoides\_subsp.\_capri\_LC\_str.\_95010
Mycoplasma\_mycoides\_subsp.\_capri\_LC\_str.\_95010
Mycoplasma\_mycoides\_subsp.\_capri\_LC\_str.\_95010
Mycoplasma\_mycoides\_subsp.\_capri\_LC\_str.\_95010
Mycoplasma\_mycoides\_subsp.\_capri\_LC\_str.\_95010
Mycoplasma\_mycoides\_subsp.\_capri\_LC\_str.\_95010
Mycoplasma\_mycoides\_subsp.\_capri\_LC\_str.\_95010
Mycoplasma\_mycoides\_subsp.\_capri\_LC\_str.\_95010
Mycoplasma\_mycoides\_subsp.\_capri\_LC\_str.\_95010
Mycoplasma\_mycoides\_subsp.\_capri\_LC\_str.\_95010
Mycoplasma\_mycoides\_subsp.\_capri\_LC\_str.\_95010
no rank

1.211.211.211.211.211.211.211.211.211.211.211.211.211.211.211.211.211.211.211.21
11111111111111111111
4.912024.850154.737674.663562.791762.791762.791762.609442.791762.609442.791762.791762.386292.098612.098612.386291.693151.6931511.69315
504742396665656643342212
2103
subspecies

1.211.211.211.211.211.211.211.211.211.211.211.211.211.211.211.211.211.211.211.21
11111111111111111111
4.912024.850154.737674.663562.791762.791762.791762.609442.791762.609442.791762.791762.386292.098612.098612.386291.693151.6931511.69315
504742396665656643342212
44101
no rank

1.211.211.211.211.211.211.211.211.211.211.211.211.211.211.211.211.211.211.211.21
11111111111111111111
4.912024.850154.737674.663562.791762.791762.791762.609442.791762.609442.791762.791762.386292.098612.098612.386291.693151.6931511.69315
504742396665656643342212
272632

Mycoplasma\_mycoides\_subsp.\_mycoides\_SC\_str.\_PG1
Mycoplasma\_mycoides\_subsp.\_mycoides\_SC\_str.\_PG1
Mycoplasma\_mycoides\_subsp.\_mycoides\_SC\_str.\_PG1
Mycoplasma\_mycoides\_subsp.\_mycoides\_SC\_str.\_PG1
Mycoplasma\_mycoides\_subsp.\_mycoides\_SC\_str.\_PG1
Mycoplasma\_mycoides\_subsp.\_mycoides\_SC\_str.\_PG1
Mycoplasma\_mycoides\_subsp.\_mycoides\_SC\_str.\_PG1
Mycoplasma\_mycoides\_subsp.\_mycoides\_SC\_str.\_PG1
Mycoplasma\_mycoides\_subsp.\_mycoides\_SC\_str.\_PG1
Mycoplasma\_mycoides\_subsp.\_mycoides\_SC\_str.\_PG1
Mycoplasma\_mycoides\_subsp.\_mycoides\_SC\_str.\_PG1
Mycoplasma\_mycoides\_subsp.\_mycoides\_SC\_str.\_PG1
Mycoplasma\_mycoides\_subsp.\_mycoides\_SC\_str.\_PG1
Mycoplasma\_mycoides\_subsp.\_mycoides\_SC\_str.\_PG1
Mycoplasma\_mycoides\_subsp.\_mycoides\_SC\_str.\_PG1
Mycoplasma\_mycoides\_subsp.\_mycoides\_SC\_str.\_PG1
Mycoplasma\_mycoides\_subsp.\_mycoides\_SC\_str.\_PG1
Mycoplasma\_mycoides\_subsp.\_mycoides\_SC\_str.\_PG1
Mycoplasma\_mycoides\_subsp.\_mycoides\_SC\_str.\_PG1
Mycoplasma\_mycoides\_subsp.\_mycoides\_SC\_str.\_PG1
no rank

0.960.960.960.960.960.960.960.960.960.960.960.960.960.960.960.960.960.960.960.96
11111111111111111111
2.609442.098612.098611.693150000000000000000
53320000000000000000
2107
species

0.960.960.960.960.960.960.960.960.960.960.960.960.960.960.960.960.960.960.960.96
11111111111111111111
2.609442.098612.098611.693150000000000000000
53320000000000000000
272635

Mycoplasma\_pulmonis\_UAB\_CTIP
Mycoplasma\_pulmonis\_UAB\_CTIP
Mycoplasma\_pulmonis\_UAB\_CTIP
Mycoplasma\_pulmonis\_UAB\_CTIP
Mycoplasma\_pulmonis\_UAB\_CTIP
Mycoplasma\_pulmonis\_UAB\_CTIP
Mycoplasma\_pulmonis\_UAB\_CTIP
Mycoplasma\_pulmonis\_UAB\_CTIP
Mycoplasma\_pulmonis\_UAB\_CTIP
Mycoplasma\_pulmonis\_UAB\_CTIP
Mycoplasma\_pulmonis\_UAB\_CTIP
Mycoplasma\_pulmonis\_UAB\_CTIP
Mycoplasma\_pulmonis\_UAB\_CTIP
Mycoplasma\_pulmonis\_UAB\_CTIP
Mycoplasma\_pulmonis\_UAB\_CTIP
Mycoplasma\_pulmonis\_UAB\_CTIP
Mycoplasma\_pulmonis\_UAB\_CTIP
Mycoplasma\_pulmonis\_UAB\_CTIP
Mycoplasma\_pulmonis\_UAB\_CTIP
Mycoplasma\_pulmonis\_UAB\_CTIP
no rank

0.920.920.920.920.920.920.920.920.920.920.920.920.920.920.920.920.920.920.920.92
11111111111111111111
11000000000000000000
11000000000000000000
136241
species

0.920.920.920.920.920.920.920.920.920.920.920.920.920.920.920.920.920.920.920.92
11111111111111111111
11000000000000000000
11000000000000000000
1111676

Mycoplasma\_haemocanis\_str.\_Illinois
Mycoplasma\_haemocanis\_str.\_Illinois
Mycoplasma\_haemocanis\_str.\_Illinois
Mycoplasma\_haemocanis\_str.\_Illinois
Mycoplasma\_haemocanis\_str.\_Illinois
Mycoplasma\_haemocanis\_str.\_Illinois
Mycoplasma\_haemocanis\_str.\_Illinois
Mycoplasma\_haemocanis\_str.\_Illinois
Mycoplasma\_haemocanis\_str.\_Illinois
Mycoplasma\_haemocanis\_str.\_Illinois
Mycoplasma\_haemocanis\_str.\_Illinois
Mycoplasma\_haemocanis\_str.\_Illinois
Mycoplasma\_haemocanis\_str.\_Illinois
Mycoplasma\_haemocanis\_str.\_Illinois
Mycoplasma\_haemocanis\_str.\_Illinois
Mycoplasma\_haemocanis\_str.\_Illinois
Mycoplasma\_haemocanis\_str.\_Illinois
Mycoplasma\_haemocanis\_str.\_Illinois
Mycoplasma\_haemocanis\_str.\_Illinois
Mycoplasma\_haemocanis\_str.\_Illinois
no rank

0.830.830.830.830.830.830.830.830.830.830.830.830.830.830.830.830.830.830.830.83
11111111111111111111
2.098611.693151.693151.693150000000000000000
32220000000000000000
2123
species

0.830.830.830.830.830.830.830.830.830.830.830.830.830.830.830.830.830.830.830.83
11111111111111111111
2.098611.693151.693151.693150000000000000000
32220000000000000000
743965

Mycoplasma\_putrefaciens\_KS1
Mycoplasma\_putrefaciens\_KS1
Mycoplasma\_putrefaciens\_KS1
Mycoplasma\_putrefaciens\_KS1
Mycoplasma\_putrefaciens\_KS1
Mycoplasma\_putrefaciens\_KS1
Mycoplasma\_putrefaciens\_KS1
Mycoplasma\_putrefaciens\_KS1
Mycoplasma\_putrefaciens\_KS1
Mycoplasma\_putrefaciens\_KS1
Mycoplasma\_putrefaciens\_KS1
Mycoplasma\_putrefaciens\_KS1
Mycoplasma\_putrefaciens\_KS1
Mycoplasma\_putrefaciens\_KS1
Mycoplasma\_putrefaciens\_KS1
Mycoplasma\_putrefaciens\_KS1
Mycoplasma\_putrefaciens\_KS1
Mycoplasma\_putrefaciens\_KS1
Mycoplasma\_putrefaciens\_KS1
Mycoplasma\_putrefaciens\_KS1
no rank

10.6610.6610.6610.6610.6610.6610.6610.6610.6610.6610.6610.6610.6610.6610.6610.6610.6610.6610.6610.66
1111111111111111111111111111111111111111
3.433253.29193.232233.139021.68321.646311.646311.646311.646311.646311.620941.620940.9868571.039550.6105530.7970180.2814260.1819890.1866790.0919325
1110982222222211110000
2096
species

0.970.970.970.970.970.970.970.970.970.970.970.970.970.970.970.970.970.970.970.97
11111111111111111111
3.39793.302593.197223.197222.098611.693151.693151.693151.693151.693151.693151.6931511010000
1110993222222211010000
1159200

Mycoplasma\_gallisepticum\_NY01\_2001.047-5-1P
Mycoplasma\_gallisepticum\_NY01\_2001.047-5-1P
Mycoplasma\_gallisepticum\_NY01\_2001.047-5-1P
Mycoplasma\_gallisepticum\_NY01\_2001.047-5-1P
Mycoplasma\_gallisepticum\_NY01\_2001.047-5-1P
Mycoplasma\_gallisepticum\_NY01\_2001.047-5-1P
Mycoplasma\_gallisepticum\_NY01\_2001.047-5-1P
Mycoplasma\_gallisepticum\_NY01\_2001.047-5-1P
Mycoplasma\_gallisepticum\_NY01\_2001.047-5-1P
Mycoplasma\_gallisepticum\_NY01\_2001.047-5-1P
Mycoplasma\_gallisepticum\_NY01\_2001.047-5-1P
Mycoplasma\_gallisepticum\_NY01\_2001.047-5-1P
Mycoplasma\_gallisepticum\_NY01\_2001.047-5-1P
Mycoplasma\_gallisepticum\_NY01\_2001.047-5-1P
Mycoplasma\_gallisepticum\_NY01\_2001.047-5-1P
Mycoplasma\_gallisepticum\_NY01\_2001.047-5-1P
Mycoplasma\_gallisepticum\_NY01\_2001.047-5-1P
Mycoplasma\_gallisepticum\_NY01\_2001.047-5-1P
Mycoplasma\_gallisepticum\_NY01\_2001.047-5-1P
Mycoplasma\_gallisepticum\_NY01\_2001.047-5-1P
no rank

0.940.940.940.940.940.940.940.940.940.940.940.940.940.940.940.940.940.940.940.94
11111111111111111111
3.564953.079443.079443.079442.386292.386292.386292.386292.386292.386292.098612.0986111110000
138884444443311110000
1159202

Mycoplasma\_gallisepticum\_NC06\_2006.080-5-2P
Mycoplasma\_gallisepticum\_NC06\_2006.080-5-2P
Mycoplasma\_gallisepticum\_NC06\_2006.080-5-2P
Mycoplasma\_gallisepticum\_NC06\_2006.080-5-2P
Mycoplasma\_gallisepticum\_NC06\_2006.080-5-2P
Mycoplasma\_gallisepticum\_NC06\_2006.080-5-2P
Mycoplasma\_gallisepticum\_NC06\_2006.080-5-2P
Mycoplasma\_gallisepticum\_NC06\_2006.080-5-2P
Mycoplasma\_gallisepticum\_NC06\_2006.080-5-2P
Mycoplasma\_gallisepticum\_NC06\_2006.080-5-2P
Mycoplasma\_gallisepticum\_NC06\_2006.080-5-2P
Mycoplasma\_gallisepticum\_NC06\_2006.080-5-2P
Mycoplasma\_gallisepticum\_NC06\_2006.080-5-2P
Mycoplasma\_gallisepticum\_NC06\_2006.080-5-2P
Mycoplasma\_gallisepticum\_NC06\_2006.080-5-2P
Mycoplasma\_gallisepticum\_NC06\_2006.080-5-2P
Mycoplasma\_gallisepticum\_NC06\_2006.080-5-2P
Mycoplasma\_gallisepticum\_NC06\_2006.080-5-2P
Mycoplasma\_gallisepticum\_NC06\_2006.080-5-2P
Mycoplasma\_gallisepticum\_NC06\_2006.080-5-2P
no rank

0.990.990.990.990.990.990.990.990.990.990.990.990.990.990.990.990.990.990.990.99
11111111111111111111
3.708053.39793.39793.197222.098612.098612.098612.098612.098612.098612.098612.098611.693151.6931501.693150000
15111193333333322020000
1159199

Mycoplasma\_gallisepticum\_NC96\_1596-4-2P
Mycoplasma\_gallisepticum\_NC96\_1596-4-2P
Mycoplasma\_gallisepticum\_NC96\_1596-4-2P
Mycoplasma\_gallisepticum\_NC96\_1596-4-2P
Mycoplasma\_gallisepticum\_NC96\_1596-4-2P
Mycoplasma\_gallisepticum\_NC96\_1596-4-2P
Mycoplasma\_gallisepticum\_NC96\_1596-4-2P
Mycoplasma\_gallisepticum\_NC96\_1596-4-2P
Mycoplasma\_gallisepticum\_NC96\_1596-4-2P
Mycoplasma\_gallisepticum\_NC96\_1596-4-2P
Mycoplasma\_gallisepticum\_NC96\_1596-4-2P
Mycoplasma\_gallisepticum\_NC96\_1596-4-2P
Mycoplasma\_gallisepticum\_NC96\_1596-4-2P
Mycoplasma\_gallisepticum\_NC96\_1596-4-2P
Mycoplasma\_gallisepticum\_NC96\_1596-4-2P
Mycoplasma\_gallisepticum\_NC96\_1596-4-2P
Mycoplasma\_gallisepticum\_NC96\_1596-4-2P
Mycoplasma\_gallisepticum\_NC96\_1596-4-2P
Mycoplasma\_gallisepticum\_NC96\_1596-4-2P
Mycoplasma\_gallisepticum\_NC96\_1596-4-2P
no rank

2.022.022.022.022.022.022.022.022.022.022.022.022.022.022.022.022.022.022.022.02
22222222222222222222
3.596483.433773.524933.29756111111111111100.50
131112101111111111111010
233150
no rank

1.011.011.011.011.011.011.011.011.011.011.011.011.011.011.011.011.011.011.011.01
11111111111111111111
3.708053.302593.564953.197221111111111111000
15101391111111111111000
710127

Mycoplasma\_gallisepticum\_str.\_R(low)
Mycoplasma\_gallisepticum\_str.\_R(low)
Mycoplasma\_gallisepticum\_str.\_R(low)
Mycoplasma\_gallisepticum\_str.\_R(low)
Mycoplasma\_gallisepticum\_str.\_R(low)
Mycoplasma\_gallisepticum\_str.\_R(low)
Mycoplasma\_gallisepticum\_str.\_R(low)
Mycoplasma\_gallisepticum\_str.\_R(low)
Mycoplasma\_gallisepticum\_str.\_R(low)
Mycoplasma\_gallisepticum\_str.\_R(low)
Mycoplasma\_gallisepticum\_str.\_R(low)
Mycoplasma\_gallisepticum\_str.\_R(low)
Mycoplasma\_gallisepticum\_str.\_R(low)
Mycoplasma\_gallisepticum\_str.\_R(low)
Mycoplasma\_gallisepticum\_str.\_R(low)
Mycoplasma\_gallisepticum\_str.\_R(low)
Mycoplasma\_gallisepticum\_str.\_R(low)
Mycoplasma\_gallisepticum\_str.\_R(low)
Mycoplasma\_gallisepticum\_str.\_R(low)
Mycoplasma\_gallisepticum\_str.\_R(low)
no rank

1.011.011.011.011.011.011.011.011.011.011.011.011.011.011.011.011.011.011.011.01
11111111111111111111
3.484913.564953.484913.39791111111111111010
121312111111111111111010
710128

Mycoplasma\_gallisepticum\_str.\_R(high)
Mycoplasma\_gallisepticum\_str.\_R(high)
Mycoplasma\_gallisepticum\_str.\_R(high)
Mycoplasma\_gallisepticum\_str.\_R(high)
Mycoplasma\_gallisepticum\_str.\_R(high)
Mycoplasma\_gallisepticum\_str.\_R(high)
Mycoplasma\_gallisepticum\_str.\_R(high)
Mycoplasma\_gallisepticum\_str.\_R(high)
Mycoplasma\_gallisepticum\_str.\_R(high)
Mycoplasma\_gallisepticum\_str.\_R(high)
Mycoplasma\_gallisepticum\_str.\_R(high)
Mycoplasma\_gallisepticum\_str.\_R(high)
Mycoplasma\_gallisepticum\_str.\_R(high)
Mycoplasma\_gallisepticum\_str.\_R(high)
Mycoplasma\_gallisepticum\_str.\_R(high)
Mycoplasma\_gallisepticum\_str.\_R(high)
Mycoplasma\_gallisepticum\_str.\_R(high)
Mycoplasma\_gallisepticum\_str.\_R(high)
Mycoplasma\_gallisepticum\_str.\_R(high)
Mycoplasma\_gallisepticum\_str.\_R(high)
no rank

0.950.950.950.950.950.950.950.950.950.950.950.950.950.950.950.950.950.950.950.95
11111111111111111111
3.302593.079442.945912.945912.098612.098612.098612.098612.098612.098612.098612.098612.0986111.6931510000
108773333333331210000
1159198

Mycoplasma\_gallisepticum\_NC95\_13295-2-2P
Mycoplasma\_gallisepticum\_NC95\_13295-2-2P
Mycoplasma\_gallisepticum\_NC95\_13295-2-2P
Mycoplasma\_gallisepticum\_NC95\_13295-2-2P
Mycoplasma\_gallisepticum\_NC95\_13295-2-2P
Mycoplasma\_gallisepticum\_NC95\_13295-2-2P
Mycoplasma\_gallisepticum\_NC95\_13295-2-2P
Mycoplasma\_gallisepticum\_NC95\_13295-2-2P
Mycoplasma\_gallisepticum\_NC95\_13295-2-2P
Mycoplasma\_gallisepticum\_NC95\_13295-2-2P
Mycoplasma\_gallisepticum\_NC95\_13295-2-2P
Mycoplasma\_gallisepticum\_NC95\_13295-2-2P
Mycoplasma\_gallisepticum\_NC95\_13295-2-2P
Mycoplasma\_gallisepticum\_NC95\_13295-2-2P
Mycoplasma\_gallisepticum\_NC95\_13295-2-2P
Mycoplasma\_gallisepticum\_NC95\_13295-2-2P
Mycoplasma\_gallisepticum\_NC95\_13295-2-2P
Mycoplasma\_gallisepticum\_NC95\_13295-2-2P
Mycoplasma\_gallisepticum\_NC95\_13295-2-2P
Mycoplasma\_gallisepticum\_NC95\_13295-2-2P
no rank

0.960.960.960.960.960.960.960.960.960.960.960.960.960.960.960.960.960.960.960.96
11111111111111111111
3.39793.197223.079442.945912.098612.098612.098612.098612.098612.098612.098612.0986111.69315110100
119873333333312110100
1159197

Mycoplasma\_gallisepticum\_VA94\_7994-1-7P
Mycoplasma\_gallisepticum\_VA94\_7994-1-7P
Mycoplasma\_gallisepticum\_VA94\_7994-1-7P
Mycoplasma\_gallisepticum\_VA94\_7994-1-7P
Mycoplasma\_gallisepticum\_VA94\_7994-1-7P
Mycoplasma\_gallisepticum\_VA94\_7994-1-7P
Mycoplasma\_gallisepticum\_VA94\_7994-1-7P
Mycoplasma\_gallisepticum\_VA94\_7994-1-7P
Mycoplasma\_gallisepticum\_VA94\_7994-1-7P
Mycoplasma\_gallisepticum\_VA94\_7994-1-7P
Mycoplasma\_gallisepticum\_VA94\_7994-1-7P
Mycoplasma\_gallisepticum\_VA94\_7994-1-7P
Mycoplasma\_gallisepticum\_VA94\_7994-1-7P
Mycoplasma\_gallisepticum\_VA94\_7994-1-7P
Mycoplasma\_gallisepticum\_VA94\_7994-1-7P
Mycoplasma\_gallisepticum\_VA94\_7994-1-7P
Mycoplasma\_gallisepticum\_VA94\_7994-1-7P
Mycoplasma\_gallisepticum\_VA94\_7994-1-7P
Mycoplasma\_gallisepticum\_VA94\_7994-1-7P
Mycoplasma\_gallisepticum\_VA94\_7994-1-7P
no rank

0.930.930.930.930.930.930.930.930.930.930.930.930.930.930.930.930.930.930.930.93
11111111111111111111
3.484913.197223.302593.079441111111100000000
1291081111111100000000
1159204

Mycoplasma\_gallisepticum\_NC08\_2008.031-4-3P
Mycoplasma\_gallisepticum\_NC08\_2008.031-4-3P
Mycoplasma\_gallisepticum\_NC08\_2008.031-4-3P
Mycoplasma\_gallisepticum\_NC08\_2008.031-4-3P
Mycoplasma\_gallisepticum\_NC08\_2008.031-4-3P
Mycoplasma\_gallisepticum\_NC08\_2008.031-4-3P
Mycoplasma\_gallisepticum\_NC08\_2008.031-4-3P
Mycoplasma\_gallisepticum\_NC08\_2008.031-4-3P
Mycoplasma\_gallisepticum\_NC08\_2008.031-4-3P
Mycoplasma\_gallisepticum\_NC08\_2008.031-4-3P
Mycoplasma\_gallisepticum\_NC08\_2008.031-4-3P
Mycoplasma\_gallisepticum\_NC08\_2008.031-4-3P
Mycoplasma\_gallisepticum\_NC08\_2008.031-4-3P
Mycoplasma\_gallisepticum\_NC08\_2008.031-4-3P
Mycoplasma\_gallisepticum\_NC08\_2008.031-4-3P
Mycoplasma\_gallisepticum\_NC08\_2008.031-4-3P
Mycoplasma\_gallisepticum\_NC08\_2008.031-4-3P
Mycoplasma\_gallisepticum\_NC08\_2008.031-4-3P
Mycoplasma\_gallisepticum\_NC08\_2008.031-4-3P
Mycoplasma\_gallisepticum\_NC08\_2008.031-4-3P
no rank

0.980.980.980.980.980.980.980.980.980.980.980.980.980.980.980.980.980.980.980.98
11111111111111111111
3.302593.484913.302593.302592.098612.098612.098612.098612.098612.098612.098612.0986111000000
101210103333333311000000
1159203

Mycoplasma\_gallisepticum\_CA06\_2006.052-5-2P
Mycoplasma\_gallisepticum\_CA06\_2006.052-5-2P
Mycoplasma\_gallisepticum\_CA06\_2006.052-5-2P
Mycoplasma\_gallisepticum\_CA06\_2006.052-5-2P
Mycoplasma\_gallisepticum\_CA06\_2006.052-5-2P
Mycoplasma\_gallisepticum\_CA06\_2006.052-5-2P
Mycoplasma\_gallisepticum\_CA06\_2006.052-5-2P
Mycoplasma\_gallisepticum\_CA06\_2006.052-5-2P
Mycoplasma\_gallisepticum\_CA06\_2006.052-5-2P
Mycoplasma\_gallisepticum\_CA06\_2006.052-5-2P
Mycoplasma\_gallisepticum\_CA06\_2006.052-5-2P
Mycoplasma\_gallisepticum\_CA06\_2006.052-5-2P
Mycoplasma\_gallisepticum\_CA06\_2006.052-5-2P
Mycoplasma\_gallisepticum\_CA06\_2006.052-5-2P
Mycoplasma\_gallisepticum\_CA06\_2006.052-5-2P
Mycoplasma\_gallisepticum\_CA06\_2006.052-5-2P
Mycoplasma\_gallisepticum\_CA06\_2006.052-5-2P
Mycoplasma\_gallisepticum\_CA06\_2006.052-5-2P
Mycoplasma\_gallisepticum\_CA06\_2006.052-5-2P
Mycoplasma\_gallisepticum\_CA06\_2006.052-5-2P
no rank

0.940.940.940.940.940.940.940.940.940.940.940.940.940.940.940.940.940.940.940.94
11111111111111111111
3.197223.079443.079443.079441.693151.693151.693151.693151.693151.693151.693151.6931501000000
98882222222201000000
1159201

Mycoplasma\_gallisepticum\_WI01\_2001.043-13-2P
Mycoplasma\_gallisepticum\_WI01\_2001.043-13-2P
Mycoplasma\_gallisepticum\_WI01\_2001.043-13-2P
Mycoplasma\_gallisepticum\_WI01\_2001.043-13-2P
Mycoplasma\_gallisepticum\_WI01\_2001.043-13-2P
Mycoplasma\_gallisepticum\_WI01\_2001.043-13-2P
Mycoplasma\_gallisepticum\_WI01\_2001.043-13-2P
Mycoplasma\_gallisepticum\_WI01\_2001.043-13-2P
Mycoplasma\_gallisepticum\_WI01\_2001.043-13-2P
Mycoplasma\_gallisepticum\_WI01\_2001.043-13-2P
Mycoplasma\_gallisepticum\_WI01\_2001.043-13-2P
Mycoplasma\_gallisepticum\_WI01\_2001.043-13-2P
Mycoplasma\_gallisepticum\_WI01\_2001.043-13-2P
Mycoplasma\_gallisepticum\_WI01\_2001.043-13-2P
Mycoplasma\_gallisepticum\_WI01\_2001.043-13-2P
Mycoplasma\_gallisepticum\_WI01\_2001.043-13-2P
Mycoplasma\_gallisepticum\_WI01\_2001.043-13-2P
Mycoplasma\_gallisepticum\_WI01\_2001.043-13-2P
Mycoplasma\_gallisepticum\_WI01\_2001.043-13-2P
Mycoplasma\_gallisepticum\_WI01\_2001.043-13-2P
no rank

0.980.980.980.980.980.980.980.980.980.980.980.980.980.980.980.980.980.980.980.98
11111111111111111111
3.197223.484913.079443.079441111111111111111
912881111111111111111
708616

Mycoplasma\_gallisepticum\_str.\_F
Mycoplasma\_gallisepticum\_str.\_F
Mycoplasma\_gallisepticum\_str.\_F
Mycoplasma\_gallisepticum\_str.\_F
Mycoplasma\_gallisepticum\_str.\_F
Mycoplasma\_gallisepticum\_str.\_F
Mycoplasma\_gallisepticum\_str.\_F
Mycoplasma\_gallisepticum\_str.\_F
Mycoplasma\_gallisepticum\_str.\_F
Mycoplasma\_gallisepticum\_str.\_F
Mycoplasma\_gallisepticum\_str.\_F
Mycoplasma\_gallisepticum\_str.\_F
Mycoplasma\_gallisepticum\_str.\_F
Mycoplasma\_gallisepticum\_str.\_F
Mycoplasma\_gallisepticum\_str.\_F
Mycoplasma\_gallisepticum\_str.\_F
Mycoplasma\_gallisepticum\_str.\_F
Mycoplasma\_gallisepticum\_str.\_F
Mycoplasma\_gallisepticum\_str.\_F
Mycoplasma\_gallisepticum\_str.\_F
no rank

2.902.902.902.902.902.902.902.902.902.902.902.902.902.902.902.902.902.902.902.90
55555555555555555555
00000000000000000000
00000000000000000000
2097
species

0.580.580.580.580.580.580.580.580.580.580.580.580.580.580.580.580.580.580.580.58
11111111111111111111
00000000000000000000
00000000000000000000
243273

Mycoplasma\_genitalium\_G37
Mycoplasma\_genitalium\_G37
Mycoplasma\_genitalium\_G37
Mycoplasma\_genitalium\_G37
Mycoplasma\_genitalium\_G37
Mycoplasma\_genitalium\_G37
Mycoplasma\_genitalium\_G37
Mycoplasma\_genitalium\_G37
Mycoplasma\_genitalium\_G37
Mycoplasma\_genitalium\_G37
Mycoplasma\_genitalium\_G37
Mycoplasma\_genitalium\_G37
Mycoplasma\_genitalium\_G37
Mycoplasma\_genitalium\_G37
Mycoplasma\_genitalium\_G37
Mycoplasma\_genitalium\_G37
Mycoplasma\_genitalium\_G37
Mycoplasma\_genitalium\_G37
Mycoplasma\_genitalium\_G37
Mycoplasma\_genitalium\_G37
no rank

0.580.580.580.580.580.580.580.580.580.580.580.580.580.580.580.580.580.580.580.58
11111111111111111111
00000000000000000000
00000000000000000000
662946

Mycoplasma\_genitalium\_M6282
Mycoplasma\_genitalium\_M6282
Mycoplasma\_genitalium\_M6282
Mycoplasma\_genitalium\_M6282
Mycoplasma\_genitalium\_M6282
Mycoplasma\_genitalium\_M6282
Mycoplasma\_genitalium\_M6282
Mycoplasma\_genitalium\_M6282
Mycoplasma\_genitalium\_M6282
Mycoplasma\_genitalium\_M6282
Mycoplasma\_genitalium\_M6282
Mycoplasma\_genitalium\_M6282
Mycoplasma\_genitalium\_M6282
Mycoplasma\_genitalium\_M6282
Mycoplasma\_genitalium\_M6282
Mycoplasma\_genitalium\_M6282
Mycoplasma\_genitalium\_M6282
Mycoplasma\_genitalium\_M6282
Mycoplasma\_genitalium\_M6282
Mycoplasma\_genitalium\_M6282
no rank

0.580.580.580.580.580.580.580.580.580.580.580.580.580.580.580.580.580.580.580.58
11111111111111111111
00000000000000000000
00000000000000000000
662945

Mycoplasma\_genitalium\_M6320
Mycoplasma\_genitalium\_M6320
Mycoplasma\_genitalium\_M6320
Mycoplasma\_genitalium\_M6320
Mycoplasma\_genitalium\_M6320
Mycoplasma\_genitalium\_M6320
Mycoplasma\_genitalium\_M6320
Mycoplasma\_genitalium\_M6320
Mycoplasma\_genitalium\_M6320
Mycoplasma\_genitalium\_M6320
Mycoplasma\_genitalium\_M6320
Mycoplasma\_genitalium\_M6320
Mycoplasma\_genitalium\_M6320
Mycoplasma\_genitalium\_M6320
Mycoplasma\_genitalium\_M6320
Mycoplasma\_genitalium\_M6320
Mycoplasma\_genitalium\_M6320
Mycoplasma\_genitalium\_M6320
Mycoplasma\_genitalium\_M6320
Mycoplasma\_genitalium\_M6320
no rank

0.580.580.580.580.580.580.580.580.580.580.580.580.580.580.580.580.580.580.580.58
11111111111111111111
00000000000000000000
00000000000000000000
662947

Mycoplasma\_genitalium\_M2288
Mycoplasma\_genitalium\_M2288
Mycoplasma\_genitalium\_M2288
Mycoplasma\_genitalium\_M2288
Mycoplasma\_genitalium\_M2288
Mycoplasma\_genitalium\_M2288
Mycoplasma\_genitalium\_M2288
Mycoplasma\_genitalium\_M2288
Mycoplasma\_genitalium\_M2288
Mycoplasma\_genitalium\_M2288
Mycoplasma\_genitalium\_M2288
Mycoplasma\_genitalium\_M2288
Mycoplasma\_genitalium\_M2288
Mycoplasma\_genitalium\_M2288
Mycoplasma\_genitalium\_M2288
Mycoplasma\_genitalium\_M2288
Mycoplasma\_genitalium\_M2288
Mycoplasma\_genitalium\_M2288
Mycoplasma\_genitalium\_M2288
Mycoplasma\_genitalium\_M2288
no rank

0.580.580.580.580.580.580.580.580.580.580.580.580.580.580.580.580.580.580.580.58
11111111111111111111
00000000000000000000
00000000000000000000
663918

Mycoplasma\_genitalium\_M2321
Mycoplasma\_genitalium\_M2321
Mycoplasma\_genitalium\_M2321
Mycoplasma\_genitalium\_M2321
Mycoplasma\_genitalium\_M2321
Mycoplasma\_genitalium\_M2321
Mycoplasma\_genitalium\_M2321
Mycoplasma\_genitalium\_M2321
Mycoplasma\_genitalium\_M2321
Mycoplasma\_genitalium\_M2321
Mycoplasma\_genitalium\_M2321
Mycoplasma\_genitalium\_M2321
Mycoplasma\_genitalium\_M2321
Mycoplasma\_genitalium\_M2321
Mycoplasma\_genitalium\_M2321
Mycoplasma\_genitalium\_M2321
Mycoplasma\_genitalium\_M2321
Mycoplasma\_genitalium\_M2321
Mycoplasma\_genitalium\_M2321
Mycoplasma\_genitalium\_M2321
no rank

0.800.800.800.800.800.800.800.800.800.800.800.800.800.800.800.800.800.800.800.80
11111111111111111111
3.197222.791762.791762.609441000000000000000
96651000000000000000
2109
species

0.800.800.800.800.800.800.800.800.800.800.800.800.800.800.800.800.800.800.800.80
11111111111111111111
3.197222.791762.791762.609441000000000000000
96651000000000000000
262723

Mycoplasma\_synoviae\_53
Mycoplasma\_synoviae\_53
Mycoplasma\_synoviae\_53
Mycoplasma\_synoviae\_53
Mycoplasma\_synoviae\_53
Mycoplasma\_synoviae\_53
Mycoplasma\_synoviae\_53
Mycoplasma\_synoviae\_53
Mycoplasma\_synoviae\_53
Mycoplasma\_synoviae\_53
Mycoplasma\_synoviae\_53
Mycoplasma\_synoviae\_53
Mycoplasma\_synoviae\_53
Mycoplasma\_synoviae\_53
Mycoplasma\_synoviae\_53
Mycoplasma\_synoviae\_53
Mycoplasma\_synoviae\_53
Mycoplasma\_synoviae\_53
Mycoplasma\_synoviae\_53
Mycoplasma\_synoviae\_53
no rank

2.102.102.102.102.102.102.102.102.102.102.102.102.102.102.102.102.102.102.102.10
22222222222222222222
3.716743.630063.536423.536422.551512.551512.495322.551512.495322.551512.495322.495322.495322.495321.955612.0628300.53333300
151413135545454444330100
2115
species

0.980.980.980.980.980.980.980.980.980.980.980.980.980.980.980.980.980.980.980.98
11111111111111111111
3.39793.39793.197223.197221.693151.693151.693151.693151.693151.693151.693151.693151.693151.6931511.693150000
1111992222222222120000
637387

Mycoplasma\_fermentans\_JER
Mycoplasma\_fermentans\_JER
Mycoplasma\_fermentans\_JER
Mycoplasma\_fermentans\_JER
Mycoplasma\_fermentans\_JER
Mycoplasma\_fermentans\_JER
Mycoplasma\_fermentans\_JER
Mycoplasma\_fermentans\_JER
Mycoplasma\_fermentans\_JER
Mycoplasma\_fermentans\_JER
Mycoplasma\_fermentans\_JER
Mycoplasma\_fermentans\_JER
Mycoplasma\_fermentans\_JER
Mycoplasma\_fermentans\_JER
Mycoplasma\_fermentans\_JER
Mycoplasma\_fermentans\_JER
Mycoplasma\_fermentans\_JER
Mycoplasma\_fermentans\_JER
Mycoplasma\_fermentans\_JER
Mycoplasma\_fermentans\_JER
no rank

1.121.121.121.121.121.121.121.121.121.121.121.121.121.121.121.121.121.121.121.12
11111111111111111111
3.995733.833213.833213.833213.302593.302593.197223.302593.197223.302593.197223.197223.197223.197222.791762.386290100
2017171710109109109999640100
943945

Mycoplasma\_fermentans\_M64
Mycoplasma\_fermentans\_M64
Mycoplasma\_fermentans\_M64
Mycoplasma\_fermentans\_M64
Mycoplasma\_fermentans\_M64
Mycoplasma\_fermentans\_M64
Mycoplasma\_fermentans\_M64
Mycoplasma\_fermentans\_M64
Mycoplasma\_fermentans\_M64
Mycoplasma\_fermentans\_M64
Mycoplasma\_fermentans\_M64
Mycoplasma\_fermentans\_M64
Mycoplasma\_fermentans\_M64
Mycoplasma\_fermentans\_M64
Mycoplasma\_fermentans\_M64
Mycoplasma\_fermentans\_M64
Mycoplasma\_fermentans\_M64
Mycoplasma\_fermentans\_M64
Mycoplasma\_fermentans\_M64
Mycoplasma\_fermentans\_M64
no rank

1.891.891.891.891.891.891.891.891.891.891.891.891.891.891.891.891.891.891.891.89
22222222222222222222
2.769892.553222.553222.496911.275221.275221.275221.275221.275221.275221.121481.275221.275221.121481.121481.121480.9048040.90480400
65541111111111111100
2110

Mycoplasma\_agalactiae
Mycoplasma\_agalactiae
Mycoplasma\_agalactiae
Mycoplasma\_agalactiae
Mycoplasma\_agalactiae
Mycoplasma\_agalactiae
Mycoplasma\_agalactiae
Mycoplasma\_agalactiae
Mycoplasma\_agalactiae
Mycoplasma\_agalactiae
Mycoplasma\_agalactiae
Mycoplasma\_agalactiae
Mycoplasma\_agalactiae
Mycoplasma\_agalactiae
Mycoplasma\_agalactiae
Mycoplasma\_agalactiae
Mycoplasma\_agalactiae
Mycoplasma\_agalactiae
Mycoplasma\_agalactiae
Mycoplasma\_agalactiae
11111111111111111111
species

0.880.880.880.880.880.880.880.880.880.880.880.880.880.880.880.880.880.880.880.88
11111111111111111111
1.693151.693151.693151.693150000000000000000
22220000000000000000
347257

Mycoplasma\_agalactiae\_PG2
Mycoplasma\_agalactiae\_PG2
Mycoplasma\_agalactiae\_PG2
Mycoplasma\_agalactiae\_PG2
Mycoplasma\_agalactiae\_PG2
Mycoplasma\_agalactiae\_PG2
Mycoplasma\_agalactiae\_PG2
Mycoplasma\_agalactiae\_PG2
Mycoplasma\_agalactiae\_PG2
Mycoplasma\_agalactiae\_PG2
Mycoplasma\_agalactiae\_PG2
Mycoplasma\_agalactiae\_PG2
Mycoplasma\_agalactiae\_PG2
Mycoplasma\_agalactiae\_PG2
Mycoplasma\_agalactiae\_PG2
Mycoplasma\_agalactiae\_PG2
Mycoplasma\_agalactiae\_PG2
Mycoplasma\_agalactiae\_PG2
Mycoplasma\_agalactiae\_PG2
Mycoplasma\_agalactiae\_PG2
no rank

0.850.850.850.850.850.850.850.850.850.850.850.850.850.850.850.850.850.850.850.85
11111111111111111111
2.098612.098612.098612.098612.098612.098612.098612.098612.098612.098612.098612.098612.098612.098612.098611.693151111
33333333333333321111
45361
species

0.850.850.850.850.850.850.850.850.850.850.850.850.850.850.850.850.850.850.850.85
11111111111111111111
2.098612.098612.098612.098612.098612.098612.098612.098612.098612.098612.098612.098612.098612.098612.098611.693151111
33333333333333321111
572263

Mycoplasma\_conjunctivae\_HRC\_581
Mycoplasma\_conjunctivae\_HRC\_581
Mycoplasma\_conjunctivae\_HRC\_581
Mycoplasma\_conjunctivae\_HRC\_581
Mycoplasma\_conjunctivae\_HRC\_581
Mycoplasma\_conjunctivae\_HRC\_581
Mycoplasma\_conjunctivae\_HRC\_581
Mycoplasma\_conjunctivae\_HRC\_581
Mycoplasma\_conjunctivae\_HRC\_581
Mycoplasma\_conjunctivae\_HRC\_581
Mycoplasma\_conjunctivae\_HRC\_581
Mycoplasma\_conjunctivae\_HRC\_581
Mycoplasma\_conjunctivae\_HRC\_581
Mycoplasma\_conjunctivae\_HRC\_581
Mycoplasma\_conjunctivae\_HRC\_581
Mycoplasma\_conjunctivae\_HRC\_581
Mycoplasma\_conjunctivae\_HRC\_581
Mycoplasma\_conjunctivae\_HRC\_581
Mycoplasma\_conjunctivae\_HRC\_581
Mycoplasma\_conjunctivae\_HRC\_581
no rank

1.451.451.451.451.451.451.451.451.451.451.451.451.451.451.451.451.451.451.451.45
22222222222222222222
00000000000000000000
00000000000000000000
57372
species

0.710.710.710.710.710.710.710.710.710.710.710.710.710.710.710.710.710.710.710.71
11111111111111111111
00000000000000000000
00000000000000000000
708248

Mycoplasma\_suis\_KI3806
Mycoplasma\_suis\_KI3806
Mycoplasma\_suis\_KI3806
Mycoplasma\_suis\_KI3806
Mycoplasma\_suis\_KI3806
Mycoplasma\_suis\_KI3806
Mycoplasma\_suis\_KI3806
Mycoplasma\_suis\_KI3806
Mycoplasma\_suis\_KI3806
Mycoplasma\_suis\_KI3806
Mycoplasma\_suis\_KI3806
Mycoplasma\_suis\_KI3806
Mycoplasma\_suis\_KI3806
Mycoplasma\_suis\_KI3806
Mycoplasma\_suis\_KI3806
Mycoplasma\_suis\_KI3806
Mycoplasma\_suis\_KI3806
Mycoplasma\_suis\_KI3806
Mycoplasma\_suis\_KI3806
Mycoplasma\_suis\_KI3806
no rank

0.740.740.740.740.740.740.740.740.740.740.740.740.740.740.740.740.740.740.740.74
11111111111111111111
00000000000000000000
00000000000000000000
768700

Mycoplasma\_suis\_str.\_Illinois
Mycoplasma\_suis\_str.\_Illinois
Mycoplasma\_suis\_str.\_Illinois
Mycoplasma\_suis\_str.\_Illinois
Mycoplasma\_suis\_str.\_Illinois
Mycoplasma\_suis\_str.\_Illinois
Mycoplasma\_suis\_str.\_Illinois
Mycoplasma\_suis\_str.\_Illinois
Mycoplasma\_suis\_str.\_Illinois
Mycoplasma\_suis\_str.\_Illinois
Mycoplasma\_suis\_str.\_Illinois
Mycoplasma\_suis\_str.\_Illinois
Mycoplasma\_suis\_str.\_Illinois
Mycoplasma\_suis\_str.\_Illinois
Mycoplasma\_suis\_str.\_Illinois
Mycoplasma\_suis\_str.\_Illinois
Mycoplasma\_suis\_str.\_Illinois
Mycoplasma\_suis\_str.\_Illinois
Mycoplasma\_suis\_str.\_Illinois
Mycoplasma\_suis\_str.\_Illinois
no rank

2.312.312.312.312.312.312.312.312.312.312.312.312.312.312.312.312.312.312.312.31
22222222222222222222
00000000000000000000
00000000000000000000
29501
species

1.161.161.161.161.161.161.161.161.161.161.161.161.161.161.161.161.161.161.161.16
11111111111111111111
00000000000000000000
00000000000000000000
859194

Mycoplasma\_haemofelis\_Ohio2
Mycoplasma\_haemofelis\_Ohio2
Mycoplasma\_haemofelis\_Ohio2
Mycoplasma\_haemofelis\_Ohio2
Mycoplasma\_haemofelis\_Ohio2
Mycoplasma\_haemofelis\_Ohio2
Mycoplasma\_haemofelis\_Ohio2
Mycoplasma\_haemofelis\_Ohio2
Mycoplasma\_haemofelis\_Ohio2
Mycoplasma\_haemofelis\_Ohio2
Mycoplasma\_haemofelis\_Ohio2
Mycoplasma\_haemofelis\_Ohio2
Mycoplasma\_haemofelis\_Ohio2
Mycoplasma\_haemofelis\_Ohio2
Mycoplasma\_haemofelis\_Ohio2
Mycoplasma\_haemofelis\_Ohio2
Mycoplasma\_haemofelis\_Ohio2
Mycoplasma\_haemofelis\_Ohio2
Mycoplasma\_haemofelis\_Ohio2
Mycoplasma\_haemofelis\_Ohio2
no rank

1.151.151.151.151.151.151.151.151.151.151.151.151.151.151.151.151.151.151.151.15
11111111111111111111
00000000000000000000
00000000000000000000
941640

Mycoplasma\_haemofelis\_str.\_Langford\_1
Mycoplasma\_haemofelis\_str.\_Langford\_1
Mycoplasma\_haemofelis\_str.\_Langford\_1
Mycoplasma\_haemofelis\_str.\_Langford\_1
Mycoplasma\_haemofelis\_str.\_Langford\_1
Mycoplasma\_haemofelis\_str.\_Langford\_1
Mycoplasma\_haemofelis\_str.\_Langford\_1
Mycoplasma\_haemofelis\_str.\_Langford\_1
Mycoplasma\_haemofelis\_str.\_Langford\_1
Mycoplasma\_haemofelis\_str.\_Langford\_1
Mycoplasma\_haemofelis\_str.\_Langford\_1
Mycoplasma\_haemofelis\_str.\_Langford\_1
Mycoplasma\_haemofelis\_str.\_Langford\_1
Mycoplasma\_haemofelis\_str.\_Langford\_1
Mycoplasma\_haemofelis\_str.\_Langford\_1
Mycoplasma\_haemofelis\_str.\_Langford\_1
Mycoplasma\_haemofelis\_str.\_Langford\_1
Mycoplasma\_haemofelis\_str.\_Langford\_1
Mycoplasma\_haemofelis\_str.\_Langford\_1
Mycoplasma\_haemofelis\_str.\_Langford\_1
no rank

1.001.001.001.001.001.001.001.001.001.001.001.001.001.001.001.001.001.001.001.00
11111111111111111111
3.708053.484913.079443.197220000000000000000
1512890000000000000000
171284
species

1.001.001.001.001.001.001.001.001.001.001.001.001.001.001.001.001.001.001.001.00
11111111111111111111
3.708053.484913.079443.197220000000000000000
1512890000000000000000
1246955

Mycoplasma\_cynos\_C142
Mycoplasma\_cynos\_C142
Mycoplasma\_cynos\_C142
Mycoplasma\_cynos\_C142
Mycoplasma\_cynos\_C142
Mycoplasma\_cynos\_C142
Mycoplasma\_cynos\_C142
Mycoplasma\_cynos\_C142
Mycoplasma\_cynos\_C142
Mycoplasma\_cynos\_C142
Mycoplasma\_cynos\_C142
Mycoplasma\_cynos\_C142
Mycoplasma\_cynos\_C142
Mycoplasma\_cynos\_C142
Mycoplasma\_cynos\_C142
Mycoplasma\_cynos\_C142
Mycoplasma\_cynos\_C142
Mycoplasma\_cynos\_C142
Mycoplasma\_cynos\_C142
Mycoplasma\_cynos\_C142
no rank

2.942.942.942.942.942.942.942.942.942.942.942.942.942.942.942.942.942.942.942.94
33333333333333333333
4.053143.682993.609093.374790000000000000000
211514110000000000000000
28903
species

0.990.990.990.990.990.990.990.990.990.990.990.990.990.990.990.990.990.990.990.99
11111111111111111111
4.25813.833213.833213.772590000000000000000
261717160000000000000000
767465

Mycoplasma\_bovis\_HB0801
Mycoplasma\_bovis\_HB0801
Mycoplasma\_bovis\_HB0801
Mycoplasma\_bovis\_HB0801
Mycoplasma\_bovis\_HB0801
Mycoplasma\_bovis\_HB0801
Mycoplasma\_bovis\_HB0801
Mycoplasma\_bovis\_HB0801
Mycoplasma\_bovis\_HB0801
Mycoplasma\_bovis\_HB0801
Mycoplasma\_bovis\_HB0801
Mycoplasma\_bovis\_HB0801
Mycoplasma\_bovis\_HB0801
Mycoplasma\_bovis\_HB0801
Mycoplasma\_bovis\_HB0801
Mycoplasma\_bovis\_HB0801
Mycoplasma\_bovis\_HB0801
Mycoplasma\_bovis\_HB0801
Mycoplasma\_bovis\_HB0801
Mycoplasma\_bovis\_HB0801
no rank

0.950.950.950.950.950.950.950.950.950.950.950.950.950.950.950.950.950.950.950.95
11111111111111111111
3.708053.197223.079442.609440000000000000000
159850000000000000000
956483

Mycoplasma\_bovis\_Hubei-1
Mycoplasma\_bovis\_Hubei-1
Mycoplasma\_bovis\_Hubei-1
Mycoplasma\_bovis\_Hubei-1
Mycoplasma\_bovis\_Hubei-1
Mycoplasma\_bovis\_Hubei-1
Mycoplasma\_bovis\_Hubei-1
Mycoplasma\_bovis\_Hubei-1
Mycoplasma\_bovis\_Hubei-1
Mycoplasma\_bovis\_Hubei-1
Mycoplasma\_bovis\_Hubei-1
Mycoplasma\_bovis\_Hubei-1
Mycoplasma\_bovis\_Hubei-1
Mycoplasma\_bovis\_Hubei-1
Mycoplasma\_bovis\_Hubei-1
Mycoplasma\_bovis\_Hubei-1
Mycoplasma\_bovis\_Hubei-1
Mycoplasma\_bovis\_Hubei-1
Mycoplasma\_bovis\_Hubei-1
Mycoplasma\_bovis\_Hubei-1
no rank

1.001.001.001.001.001.001.001.001.001.001.001.001.001.001.001.001.001.001.001.00
11111111111111111111
4.178053.995733.890373.708050000000000000000
242018150000000000000000
289397

Mycoplasma\_bovis\_PG45
Mycoplasma\_bovis\_PG45
Mycoplasma\_bovis\_PG45
Mycoplasma\_bovis\_PG45
Mycoplasma\_bovis\_PG45
Mycoplasma\_bovis\_PG45
Mycoplasma\_bovis\_PG45
Mycoplasma\_bovis\_PG45
Mycoplasma\_bovis\_PG45
Mycoplasma\_bovis\_PG45
Mycoplasma\_bovis\_PG45
Mycoplasma\_bovis\_PG45
Mycoplasma\_bovis\_PG45
Mycoplasma\_bovis\_PG45
Mycoplasma\_bovis\_PG45
Mycoplasma\_bovis\_PG45
Mycoplasma\_bovis\_PG45
Mycoplasma\_bovis\_PG45
Mycoplasma\_bovis\_PG45
Mycoplasma\_bovis\_PG45
no rank

0.670.670.670.670.670.670.670.670.670.670.670.670.670.670.670.670.670.670.670.67
11111111111111111111
2.386292.609442.386292.386290000000000000000
45440000000000000000
2098
species

0.670.670.670.670.670.670.670.670.670.670.670.670.670.670.670.670.670.670.670.67
11111111111111111111
2.386292.609442.386292.386290000000000000000
45440000000000000000
347256

Mycoplasma\_hominis\_ATCC\_23114
Mycoplasma\_hominis\_ATCC\_23114
Mycoplasma\_hominis\_ATCC\_23114
Mycoplasma\_hominis\_ATCC\_23114
Mycoplasma\_hominis\_ATCC\_23114
Mycoplasma\_hominis\_ATCC\_23114
Mycoplasma\_hominis\_ATCC\_23114
Mycoplasma\_hominis\_ATCC\_23114
Mycoplasma\_hominis\_ATCC\_23114
Mycoplasma\_hominis\_ATCC\_23114
Mycoplasma\_hominis\_ATCC\_23114
Mycoplasma\_hominis\_ATCC\_23114
Mycoplasma\_hominis\_ATCC\_23114
Mycoplasma\_hominis\_ATCC\_23114
Mycoplasma\_hominis\_ATCC\_23114
Mycoplasma\_hominis\_ATCC\_23114
Mycoplasma\_hominis\_ATCC\_23114
Mycoplasma\_hominis\_ATCC\_23114
Mycoplasma\_hominis\_ATCC\_23114
Mycoplasma\_hominis\_ATCC\_23114
no rank

3.643.643.643.643.643.643.643.643.643.643.643.643.643.643.643.643.643.643.643.64
44444444444444444444
3.473483.073643.029692.834330.50.50.50.50.50.50.50.500000000
128861111111100000000
2099
species

0.900.900.900.900.900.900.900.900.900.900.900.900.900.900.900.900.900.900.900.90
11111111111111111111
3.39792.945913.079442.945911111111100000000
117871111111100000000
262719

Mycoplasma\_hyopneumoniae\_J
Mycoplasma\_hyopneumoniae\_J
Mycoplasma\_hyopneumoniae\_J
Mycoplasma\_hyopneumoniae\_J
Mycoplasma\_hyopneumoniae\_J
Mycoplasma\_hyopneumoniae\_J
Mycoplasma\_hyopneumoniae\_J
Mycoplasma\_hyopneumoniae\_J
Mycoplasma\_hyopneumoniae\_J
Mycoplasma\_hyopneumoniae\_J
Mycoplasma\_hyopneumoniae\_J
Mycoplasma\_hyopneumoniae\_J
Mycoplasma\_hyopneumoniae\_J
Mycoplasma\_hyopneumoniae\_J
Mycoplasma\_hyopneumoniae\_J
Mycoplasma\_hyopneumoniae\_J
Mycoplasma\_hyopneumoniae\_J
Mycoplasma\_hyopneumoniae\_J
Mycoplasma\_hyopneumoniae\_J
Mycoplasma\_hyopneumoniae\_J
no rank

0.920.920.920.920.920.920.920.920.920.920.920.920.920.920.920.920.920.920.920.92
11111111111111111111
3.564953.197223.302592.791761111111100000000
1391061111111100000000
262722

Mycoplasma\_hyopneumoniae\_7448
Mycoplasma\_hyopneumoniae\_7448
Mycoplasma\_hyopneumoniae\_7448
Mycoplasma\_hyopneumoniae\_7448
Mycoplasma\_hyopneumoniae\_7448
Mycoplasma\_hyopneumoniae\_7448
Mycoplasma\_hyopneumoniae\_7448
Mycoplasma\_hyopneumoniae\_7448
Mycoplasma\_hyopneumoniae\_7448
Mycoplasma\_hyopneumoniae\_7448
Mycoplasma\_hyopneumoniae\_7448
Mycoplasma\_hyopneumoniae\_7448
Mycoplasma\_hyopneumoniae\_7448
Mycoplasma\_hyopneumoniae\_7448
Mycoplasma\_hyopneumoniae\_7448
Mycoplasma\_hyopneumoniae\_7448
Mycoplasma\_hyopneumoniae\_7448
Mycoplasma\_hyopneumoniae\_7448
Mycoplasma\_hyopneumoniae\_7448
Mycoplasma\_hyopneumoniae\_7448
no rank

0.890.890.890.890.890.890.890.890.890.890.890.890.890.890.890.890.890.890.890.89
11111111111111111111
3.079442.945912.945912.386290000000000000000
87740000000000000000
295358

Mycoplasma\_hyopneumoniae\_232
Mycoplasma\_hyopneumoniae\_232
Mycoplasma\_hyopneumoniae\_232
Mycoplasma\_hyopneumoniae\_232
Mycoplasma\_hyopneumoniae\_232
Mycoplasma\_hyopneumoniae\_232
Mycoplasma\_hyopneumoniae\_232
Mycoplasma\_hyopneumoniae\_232
Mycoplasma\_hyopneumoniae\_232
Mycoplasma\_hyopneumoniae\_232
Mycoplasma\_hyopneumoniae\_232
Mycoplasma\_hyopneumoniae\_232
Mycoplasma\_hyopneumoniae\_232
Mycoplasma\_hyopneumoniae\_232
Mycoplasma\_hyopneumoniae\_232
Mycoplasma\_hyopneumoniae\_232
Mycoplasma\_hyopneumoniae\_232
Mycoplasma\_hyopneumoniae\_232
Mycoplasma\_hyopneumoniae\_232
Mycoplasma\_hyopneumoniae\_232
no rank

0.930.930.930.930.930.930.930.930.930.930.930.930.930.930.930.930.930.930.930.93
11111111111111111111
3.833213.197222.791763.197220000000000000000
179690000000000000000
907287

Mycoplasma\_hyopneumoniae\_168
Mycoplasma\_hyopneumoniae\_168
Mycoplasma\_hyopneumoniae\_168
Mycoplasma\_hyopneumoniae\_168
Mycoplasma\_hyopneumoniae\_168
Mycoplasma\_hyopneumoniae\_168
Mycoplasma\_hyopneumoniae\_168
Mycoplasma\_hyopneumoniae\_168
Mycoplasma\_hyopneumoniae\_168
Mycoplasma\_hyopneumoniae\_168
Mycoplasma\_hyopneumoniae\_168
Mycoplasma\_hyopneumoniae\_168
Mycoplasma\_hyopneumoniae\_168
Mycoplasma\_hyopneumoniae\_168
Mycoplasma\_hyopneumoniae\_168
Mycoplasma\_hyopneumoniae\_168
Mycoplasma\_hyopneumoniae\_168
Mycoplasma\_hyopneumoniae\_168
Mycoplasma\_hyopneumoniae\_168
Mycoplasma\_hyopneumoniae\_168
no rank

4.554.554.554.554.554.554.554.554.554.554.554.554.554.554.554.554.554.554.554.55
55555555555555555555
3.169753.240443.0633.078921.723381.526171.401981.349491.507731.349491.367921.329111.238821.00111.079521.021210.4100850.2232720.2232720.223272
99882211211111111000
186329
order

4.554.554.554.554.554.554.554.554.554.554.554.554.554.554.554.554.554.554.554.55
55555555555555555555
3.169753.240443.0633.078921.723381.526171.401981.349491.507731.349491.367921.329111.238821.00111.079521.021210.4100850.2232720.2232720.223272
99882211211111111000
2146
family

3.053.053.053.053.053.053.053.053.053.053.053.053.053.053.053.053.053.053.053.05
44444444444444444444
3.895943.8023.73673.760452.570942.276742.091492.013182.249242.013182.040671.982781.848081.493441.610431.523440.6117670.3330780.3330780.333078
181615165433333322221111
33926
genus

1.571.571.571.571.571.571.571.571.571.571.571.571.571.571.571.571.571.571.571.57
22222222222222222222
4.068223.903513.918993.860182.287941.871351.511461.412751.871351.412751.412751.412751.291940.9166720.9166721.136190.541401000
221819174222222211111000
85620
species group

0.720.720.720.720.720.720.720.720.720.720.720.720.720.720.720.720.720.720.720.72
11111111111111111111
3.890373.484913.564953.484911.69315100100000000000
181213122100100000000000
229545
species

0.720.720.720.720.720.720.720.720.720.720.720.720.720.720.720.720.720.720.720.72
11111111111111111111
3.890373.484913.564953.484911.69315100100000000000
181213122100100000000000
322098

Aster\_yellows\_witches'-broom\_phytoplasma\_AYWB
Aster\_yellows\_witches'-broom\_phytoplasma\_AYWB
Aster\_yellows\_witches'-broom\_phytoplasma\_AYWB
Aster\_yellows\_witches'-broom\_phytoplasma\_AYWB
Aster\_yellows\_witches'-broom\_phytoplasma\_AYWB
Aster\_yellows\_witches'-broom\_phytoplasma\_AYWB
Aster\_yellows\_witches'-broom\_phytoplasma\_AYWB
Aster\_yellows\_witches'-broom\_phytoplasma\_AYWB
Aster\_yellows\_witches'-broom\_phytoplasma\_AYWB
Aster\_yellows\_witches'-broom\_phytoplasma\_AYWB
Aster\_yellows\_witches'-broom\_phytoplasma\_AYWB
Aster\_yellows\_witches'-broom\_phytoplasma\_AYWB
Aster\_yellows\_witches'-broom\_phytoplasma\_AYWB
Aster\_yellows\_witches'-broom\_phytoplasma\_AYWB
Aster\_yellows\_witches'-broom\_phytoplasma\_AYWB
Aster\_yellows\_witches'-broom\_phytoplasma\_AYWB
Aster\_yellows\_witches'-broom\_phytoplasma\_AYWB
Aster\_yellows\_witches'-broom\_phytoplasma\_AYWB
Aster\_yellows\_witches'-broom\_phytoplasma\_AYWB
Aster\_yellows\_witches'-broom\_phytoplasma\_AYWB
no rank

0.850.850.850.850.850.850.850.850.850.850.850.850.850.850.850.850.850.850.850.85
11111111111111111111
4.218884.25814.218884.178052.791762.609442.791762.609442.609442.609442.609442.609442.386291.693151.693152.098611000
252625246565555542231000
100379
species

0.850.850.850.850.850.850.850.850.850.850.850.850.850.850.850.850.850.850.850.85
11111111111111111111
4.218884.25814.218884.178052.791762.609442.791762.609442.609442.609442.609442.609442.386291.693151.693152.098611000
252625246565555542231000
262768

Onion\_yellows\_phytoplasma\_OY-M
Onion\_yellows\_phytoplasma\_OY-M
Onion\_yellows\_phytoplasma\_OY-M
Onion\_yellows\_phytoplasma\_OY-M
Onion\_yellows\_phytoplasma\_OY-M
Onion\_yellows\_phytoplasma\_OY-M
Onion\_yellows\_phytoplasma\_OY-M
Onion\_yellows\_phytoplasma\_OY-M
Onion\_yellows\_phytoplasma\_OY-M
Onion\_yellows\_phytoplasma\_OY-M
Onion\_yellows\_phytoplasma\_OY-M
Onion\_yellows\_phytoplasma\_OY-M
Onion\_yellows\_phytoplasma\_OY-M
Onion\_yellows\_phytoplasma\_OY-M
Onion\_yellows\_phytoplasma\_OY-M
Onion\_yellows\_phytoplasma\_OY-M
Onion\_yellows\_phytoplasma\_OY-M
Onion\_yellows\_phytoplasma\_OY-M
Onion\_yellows\_phytoplasma\_OY-M
Onion\_yellows\_phytoplasma\_OY-M
no rank

0.600.600.600.600.600.600.600.600.600.600.600.600.600.600.600.600.600.600.600.60
11111111111111111111
2.609442.609442.386292.609442.098611.693151.693151.693151.693151.693151.693151.693151.693151.693151.693151.693151.693151.693151.693151.69315
55453222222222222222
85630
species group

0.600.600.600.600.600.600.600.600.600.600.600.600.600.600.600.600.600.600.600.60
11111111111111111111
2.609442.609442.386292.609442.098611.693151.693151.693151.693151.693151.693151.693151.693151.693151.693151.693151.693151.693151.693151.69315
55453222222222222222
37692

Candidatus\_Phytoplasma\_mali
Candidatus\_Phytoplasma\_mali
Candidatus\_Phytoplasma\_mali
Candidatus\_Phytoplasma\_mali
Candidatus\_Phytoplasma\_mali
Candidatus\_Phytoplasma\_mali
Candidatus\_Phytoplasma\_mali
Candidatus\_Phytoplasma\_mali
Candidatus\_Phytoplasma\_mali
Candidatus\_Phytoplasma\_mali
Candidatus\_Phytoplasma\_mali
Candidatus\_Phytoplasma\_mali
Candidatus\_Phytoplasma\_mali
Candidatus\_Phytoplasma\_mali
Candidatus\_Phytoplasma\_mali
Candidatus\_Phytoplasma\_mali
Candidatus\_Phytoplasma\_mali
Candidatus\_Phytoplasma\_mali
Candidatus\_Phytoplasma\_mali
Candidatus\_Phytoplasma\_mali
species

0.880.880.880.880.880.880.880.880.880.880.880.880.880.880.880.880.880.880.880.88
11111111111111111111
4.465744.433994.33224.36733.39793.39793.39793.302593.302593.302593.39793.197222.945912.386292.791762.098610000
3231282911111110101011974630000
85632
species group

0.880.880.880.880.880.880.880.880.880.880.880.880.880.880.880.880.880.880.880.88
11111111111111111111
4.465744.433994.33224.36733.39793.39793.39793.302593.302593.302593.39793.197222.945912.386292.791762.098610000
3231282911111110101011974630000
59748

Candidatus\_Phytoplasma\_australiense
Candidatus\_Phytoplasma\_australiense
Candidatus\_Phytoplasma\_australiense
Candidatus\_Phytoplasma\_australiense
Candidatus\_Phytoplasma\_australiense
Candidatus\_Phytoplasma\_australiense
Candidatus\_Phytoplasma\_australiense
Candidatus\_Phytoplasma\_australiense
Candidatus\_Phytoplasma\_australiense
Candidatus\_Phytoplasma\_australiense
Candidatus\_Phytoplasma\_australiense
Candidatus\_Phytoplasma\_australiense
Candidatus\_Phytoplasma\_australiense
Candidatus\_Phytoplasma\_australiense
Candidatus\_Phytoplasma\_australiense
Candidatus\_Phytoplasma\_australiense
Candidatus\_Phytoplasma\_australiense
Candidatus\_Phytoplasma\_australiense
Candidatus\_Phytoplasma\_australiense
Candidatus\_Phytoplasma\_australiense
species

1.501.501.501.501.501.501.501.501.501.501.501.501.501.501.501.501.501.501.501.50
11111111111111111111
1.693152.098611.693151.693150000000000000000
23220000000000000000
2147
genus

1.501.501.501.501.501.501.501.501.501.501.501.501.501.501.501.501.501.501.501.50
11111111111111111111
1.693152.098611.693151.693150000000000000000
23220000000000000000
2148
species

1.501.501.501.501.501.501.501.501.501.501.501.501.501.501.501.501.501.501.501.50
11111111111111111111
1.693152.098611.693151.693150000000000000000
23220000000000000000
441768

Acholeplasma\_laidlawii\_PG-8A
Acholeplasma\_laidlawii\_PG-8A
Acholeplasma\_laidlawii\_PG-8A
Acholeplasma\_laidlawii\_PG-8A
Acholeplasma\_laidlawii\_PG-8A
Acholeplasma\_laidlawii\_PG-8A
Acholeplasma\_laidlawii\_PG-8A
Acholeplasma\_laidlawii\_PG-8A
Acholeplasma\_laidlawii\_PG-8A
Acholeplasma\_laidlawii\_PG-8A
Acholeplasma\_laidlawii\_PG-8A
Acholeplasma\_laidlawii\_PG-8A
Acholeplasma\_laidlawii\_PG-8A
Acholeplasma\_laidlawii\_PG-8A
Acholeplasma\_laidlawii\_PG-8A
Acholeplasma\_laidlawii\_PG-8A
Acholeplasma\_laidlawii\_PG-8A
Acholeplasma\_laidlawii\_PG-8A
Acholeplasma\_laidlawii\_PG-8A
Acholeplasma\_laidlawii\_PG-8A
no rank

31.1831.1831.1831.1831.1831.1831.1831.1831.1831.1831.1831.1831.1831.1831.1831.1831.1831.1831.1831.18
1515151515151515151515151515151515151515
2.791932.627752.415562.196690.2233990.2859390.2233990.2233990.2859390.1618210.2233990.2233990.1618210.1618210.1618210.1618210.09557410.1618210.1618210.0955741
65430000000000000000
200918
phylum

31.1831.1831.1831.1831.1831.1831.1831.1831.1831.1831.1831.1831.1831.1831.1831.1831.1831.1831.1831.18
1515151515151515151515151515151515151515
2.791932.627752.415562.196690.2233990.2859390.2233990.2233990.2859390.1618210.2233990.2233990.1618210.1618210.1618210.1618210.09557410.1618210.1618210.0955741
65430000000000000000
188708
class

31.1831.1831.1831.1831.1831.1831.1831.1831.1831.1831.1831.1831.1831.1831.1831.1831.1831.1831.1831.18
1515151515151515151515151515151515151515
2.791932.627752.415562.196690.2233990.2859390.2233990.2233990.2859390.1618210.2233990.2233990.1618210.1618210.1618210.1618210.09557410.1618210.1618210.0955741
65430000000000000000
2419
order

31.1831.1831.1831.1831.1831.1831.1831.1831.1831.1831.1831.1831.1831.1831.1831.1831.1831.1831.1831.18
1515151515151515151515151515151515151515
2.791932.627752.415562.196690.2233990.2859390.2233990.2233990.2859390.1618210.2233990.2233990.1618210.1618210.1618210.1618210.09557410.1618210.1618210.0955741
65430000000000000000
188709
family

2.302.302.302.302.302.302.302.302.302.302.302.302.302.302.302.302.302.302.302.30
11111111111111111111
4.465744.135494.044524.091040000000000000000
322321220000000000000000
651456
genus

2.302.302.302.302.302.302.302.302.302.302.302.302.302.302.302.302.302.302.302.30
11111111111111111111
4.465744.135494.044524.091040000000000000000
322321220000000000000000
651457
species

2.302.302.302.302.302.302.302.302.302.302.302.302.302.302.302.302.302.302.302.30
11111111111111111111
4.465744.135494.044524.091040000000000000000
322321220000000000000000
521045

Kosmotoga\_olearia\_TBF\_19.5.1
Kosmotoga\_olearia\_TBF\_19.5.1
Kosmotoga\_olearia\_TBF\_19.5.1
Kosmotoga\_olearia\_TBF\_19.5.1
Kosmotoga\_olearia\_TBF\_19.5.1
Kosmotoga\_olearia\_TBF\_19.5.1
Kosmotoga\_olearia\_TBF\_19.5.1
Kosmotoga\_olearia\_TBF\_19.5.1
Kosmotoga\_olearia\_TBF\_19.5.1
Kosmotoga\_olearia\_TBF\_19.5.1
Kosmotoga\_olearia\_TBF\_19.5.1
Kosmotoga\_olearia\_TBF\_19.5.1
Kosmotoga\_olearia\_TBF\_19.5.1
Kosmotoga\_olearia\_TBF\_19.5.1
Kosmotoga\_olearia\_TBF\_19.5.1
Kosmotoga\_olearia\_TBF\_19.5.1
Kosmotoga\_olearia\_TBF\_19.5.1
Kosmotoga\_olearia\_TBF\_19.5.1
Kosmotoga\_olearia\_TBF\_19.5.1
Kosmotoga\_olearia\_TBF\_19.5.1
no rank

4.124.124.124.124.124.124.124.124.124.124.124.124.124.124.124.124.124.124.124.12
22222222222222222222
3.133772.732742.86892.7513700.473301000.47330100000000000
86660100100000000000
2422
genus

2.172.172.172.172.172.172.172.172.172.172.172.172.172.172.172.172.172.172.172.17
11111111111111111111
3.302593.302593.302593.079440000000000000000
10101080000000000000000
93466
species

2.172.172.172.172.172.172.172.172.172.172.172.172.172.172.172.172.172.172.172.17
11111111111111111111
3.302593.302593.302593.079440000000000000000
10101080000000000000000
771875

Fervidobacterium\_pennivorans\_DSM\_9078
Fervidobacterium\_pennivorans\_DSM\_9078
Fervidobacterium\_pennivorans\_DSM\_9078
Fervidobacterium\_pennivorans\_DSM\_9078
Fervidobacterium\_pennivorans\_DSM\_9078
Fervidobacterium\_pennivorans\_DSM\_9078
Fervidobacterium\_pennivorans\_DSM\_9078
Fervidobacterium\_pennivorans\_DSM\_9078
Fervidobacterium\_pennivorans\_DSM\_9078
Fervidobacterium\_pennivorans\_DSM\_9078
Fervidobacterium\_pennivorans\_DSM\_9078
Fervidobacterium\_pennivorans\_DSM\_9078
Fervidobacterium\_pennivorans\_DSM\_9078
Fervidobacterium\_pennivorans\_DSM\_9078
Fervidobacterium\_pennivorans\_DSM\_9078
Fervidobacterium\_pennivorans\_DSM\_9078
Fervidobacterium\_pennivorans\_DSM\_9078
Fervidobacterium\_pennivorans\_DSM\_9078
Fervidobacterium\_pennivorans\_DSM\_9078
Fervidobacterium\_pennivorans\_DSM\_9078
no rank

1.951.951.951.951.951.951.951.951.951.951.951.951.951.951.951.951.951.951.951.95
11111111111111111111
2.945912.098612.386292.386290100100000000000
73440100100000000000
2424
species

1.951.951.951.951.951.951.951.951.951.951.951.951.951.951.951.951.951.951.951.95
11111111111111111111
2.945912.098612.386292.386290100100000000000
73440100100000000000
381764

Fervidobacterium\_nodosum\_Rt17-B1
Fervidobacterium\_nodosum\_Rt17-B1
Fervidobacterium\_nodosum\_Rt17-B1
Fervidobacterium\_nodosum\_Rt17-B1
Fervidobacterium\_nodosum\_Rt17-B1
Fervidobacterium\_nodosum\_Rt17-B1
Fervidobacterium\_nodosum\_Rt17-B1
Fervidobacterium\_nodosum\_Rt17-B1
Fervidobacterium\_nodosum\_Rt17-B1
Fervidobacterium\_nodosum\_Rt17-B1
Fervidobacterium\_nodosum\_Rt17-B1
Fervidobacterium\_nodosum\_Rt17-B1
Fervidobacterium\_nodosum\_Rt17-B1
Fervidobacterium\_nodosum\_Rt17-B1
Fervidobacterium\_nodosum\_Rt17-B1
Fervidobacterium\_nodosum\_Rt17-B1
Fervidobacterium\_nodosum\_Rt17-B1
Fervidobacterium\_nodosum\_Rt17-B1
Fervidobacterium\_nodosum\_Rt17-B1
Fervidobacterium\_nodosum\_Rt17-B1
no rank

3.943.943.943.943.943.943.943.943.943.943.943.943.943.943.943.943.943.943.943.94
22222222222222222222
3.205772.904933.058282.594170.487310.487310.487310.487310.4873100.487310.4873100000000
97851111101100000000
2420
genus

2.022.022.022.022.022.022.022.022.022.022.022.022.022.022.022.022.022.022.022.02
11111111111111111111
3.772593.39793.484912.791760000000000000000
16111260000000000000000
2421
species

2.022.022.022.022.022.022.022.022.022.022.022.022.022.022.022.022.022.022.022.02
11111111111111111111
3.772593.39793.484912.791760000000000000000
16111260000000000000000
484019

Thermosipho\_africanus\_TCF52B
Thermosipho\_africanus\_TCF52B
Thermosipho\_africanus\_TCF52B
Thermosipho\_africanus\_TCF52B
Thermosipho\_africanus\_TCF52B
Thermosipho\_africanus\_TCF52B
Thermosipho\_africanus\_TCF52B
Thermosipho\_africanus\_TCF52B
Thermosipho\_africanus\_TCF52B
Thermosipho\_africanus\_TCF52B
Thermosipho\_africanus\_TCF52B
Thermosipho\_africanus\_TCF52B
Thermosipho\_africanus\_TCF52B
Thermosipho\_africanus\_TCF52B
Thermosipho\_africanus\_TCF52B
Thermosipho\_africanus\_TCF52B
Thermosipho\_africanus\_TCF52B
Thermosipho\_africanus\_TCF52B
Thermosipho\_africanus\_TCF52B
Thermosipho\_africanus\_TCF52B
no rank

1.921.921.921.921.921.921.921.921.921.921.921.921.921.921.921.921.921.921.921.92
11111111111111111111
2.609442.386292.609442.386291111101100000000
54541111101100000000
46541
species

1.921.921.921.921.921.921.921.921.921.921.921.921.921.921.921.921.921.921.921.92
11111111111111111111
2.609442.386292.609442.386291111101100000000
54541111101100000000
391009

Thermosipho\_melanesiensis\_BI429
Thermosipho\_melanesiensis\_BI429
Thermosipho\_melanesiensis\_BI429
Thermosipho\_melanesiensis\_BI429
Thermosipho\_melanesiensis\_BI429
Thermosipho\_melanesiensis\_BI429
Thermosipho\_melanesiensis\_BI429
Thermosipho\_melanesiensis\_BI429
Thermosipho\_melanesiensis\_BI429
Thermosipho\_melanesiensis\_BI429
Thermosipho\_melanesiensis\_BI429
Thermosipho\_melanesiensis\_BI429
Thermosipho\_melanesiensis\_BI429
Thermosipho\_melanesiensis\_BI429
Thermosipho\_melanesiensis\_BI429
Thermosipho\_melanesiensis\_BI429
Thermosipho\_melanesiensis\_BI429
Thermosipho\_melanesiensis\_BI429
Thermosipho\_melanesiensis\_BI429
Thermosipho\_melanesiensis\_BI429
no rank

13.4313.4313.4313.4313.4313.4313.4313.4313.4313.4313.4313.4313.4313.4313.4313.4313.4313.4313.4313.43
77777777777777777777
1.629681.682381.159140.9478770000000000000000
22110000000000000000
2335
genus

2.042.042.042.042.042.042.042.042.042.042.042.042.042.042.042.042.042.042.042.04
11111111111111111111
1.693152.38629110000000000000000
24110000000000000000
119394
species

2.042.042.042.042.042.042.042.042.042.042.042.042.042.042.042.042.042.042.042.04
11111111111111111111
1.693152.38629110000000000000000
24110000000000000000
688269

Thermotoga\_thermarum\_DSM\_5069
Thermotoga\_thermarum\_DSM\_5069
Thermotoga\_thermarum\_DSM\_5069
Thermotoga\_thermarum\_DSM\_5069
Thermotoga\_thermarum\_DSM\_5069
Thermotoga\_thermarum\_DSM\_5069
Thermotoga\_thermarum\_DSM\_5069
Thermotoga\_thermarum\_DSM\_5069
Thermotoga\_thermarum\_DSM\_5069
Thermotoga\_thermarum\_DSM\_5069
Thermotoga\_thermarum\_DSM\_5069
Thermotoga\_thermarum\_DSM\_5069
Thermotoga\_thermarum\_DSM\_5069
Thermotoga\_thermarum\_DSM\_5069
Thermotoga\_thermarum\_DSM\_5069
Thermotoga\_thermarum\_DSM\_5069
Thermotoga\_thermarum\_DSM\_5069
Thermotoga\_thermarum\_DSM\_5069
Thermotoga\_thermarum\_DSM\_5069
Thermotoga\_thermarum\_DSM\_5069
no rank

1.881.881.881.881.881.881.881.881.881.881.881.881.881.881.881.881.881.881.881.88
11111111111111111111
2.098612.386292.098612.098610000000000000000
34330000000000000000
126740

Thermotoga\_sp.\_RQ2
Thermotoga\_sp.\_RQ2
Thermotoga\_sp.\_RQ2
Thermotoga\_sp.\_RQ2
Thermotoga\_sp.\_RQ2
Thermotoga\_sp.\_RQ2
Thermotoga\_sp.\_RQ2
Thermotoga\_sp.\_RQ2
Thermotoga\_sp.\_RQ2
Thermotoga\_sp.\_RQ2
Thermotoga\_sp.\_RQ2
Thermotoga\_sp.\_RQ2
Thermotoga\_sp.\_RQ2
Thermotoga\_sp.\_RQ2
Thermotoga\_sp.\_RQ2
Thermotoga\_sp.\_RQ2
Thermotoga\_sp.\_RQ2
Thermotoga\_sp.\_RQ2
Thermotoga\_sp.\_RQ2
Thermotoga\_sp.\_RQ2
species

1.821.821.821.821.821.821.821.821.821.821.821.821.821.821.821.821.821.821.821.82
11111111111111111111
1.693151010000000000000000
21010000000000000000
93929
species

1.821.821.821.821.821.821.821.821.821.821.821.821.821.821.821.821.821.821.821.82
11111111111111111111
1.693151010000000000000000
21010000000000000000
390874

Thermotoga\_petrophila\_RKU-1
Thermotoga\_petrophila\_RKU-1
Thermotoga\_petrophila\_RKU-1
Thermotoga\_petrophila\_RKU-1
Thermotoga\_petrophila\_RKU-1
Thermotoga\_petrophila\_RKU-1
Thermotoga\_petrophila\_RKU-1
Thermotoga\_petrophila\_RKU-1
Thermotoga\_petrophila\_RKU-1
Thermotoga\_petrophila\_RKU-1
Thermotoga\_petrophila\_RKU-1
Thermotoga\_petrophila\_RKU-1
Thermotoga\_petrophila\_RKU-1
Thermotoga\_petrophila\_RKU-1
Thermotoga\_petrophila\_RKU-1
Thermotoga\_petrophila\_RKU-1
Thermotoga\_petrophila\_RKU-1
Thermotoga\_petrophila\_RKU-1
Thermotoga\_petrophila\_RKU-1
Thermotoga\_petrophila\_RKU-1
no rank

1.811.811.811.811.811.811.811.811.811.811.811.811.811.811.811.811.811.811.811.81
11111111111111111111
2.386292.098612.098611.693150000000000000000
43320000000000000000
93930
species

1.811.811.811.811.811.811.811.811.811.811.811.811.811.811.811.811.811.811.811.81
11111111111111111111
2.386292.098612.098611.693150000000000000000
43320000000000000000
590168

Thermotoga\_naphthophila\_RKU-10
Thermotoga\_naphthophila\_RKU-10
Thermotoga\_naphthophila\_RKU-10
Thermotoga\_naphthophila\_RKU-10
Thermotoga\_naphthophila\_RKU-10
Thermotoga\_naphthophila\_RKU-10
Thermotoga\_naphthophila\_RKU-10
Thermotoga\_naphthophila\_RKU-10
Thermotoga\_naphthophila\_RKU-10
Thermotoga\_naphthophila\_RKU-10
Thermotoga\_naphthophila\_RKU-10
Thermotoga\_naphthophila\_RKU-10
Thermotoga\_naphthophila\_RKU-10
Thermotoga\_naphthophila\_RKU-10
Thermotoga\_naphthophila\_RKU-10
Thermotoga\_naphthophila\_RKU-10
Thermotoga\_naphthophila\_RKU-10
Thermotoga\_naphthophila\_RKU-10
Thermotoga\_naphthophila\_RKU-10
Thermotoga\_naphthophila\_RKU-10
no rank

2.142.142.142.142.142.142.142.142.142.142.142.142.142.142.142.142.142.142.142.14
11111111111111111111
00000000000000000000
00000000000000000000
177758
species

2.142.142.142.142.142.142.142.142.142.142.142.142.142.142.142.142.142.142.142.14
11111111111111111111
00000000000000000000
00000000000000000000
416591

Thermotoga\_lettingae\_TMO
Thermotoga\_lettingae\_TMO
Thermotoga\_lettingae\_TMO
Thermotoga\_lettingae\_TMO
Thermotoga\_lettingae\_TMO
Thermotoga\_lettingae\_TMO
Thermotoga\_lettingae\_TMO
Thermotoga\_lettingae\_TMO
Thermotoga\_lettingae\_TMO
Thermotoga\_lettingae\_TMO
Thermotoga\_lettingae\_TMO
Thermotoga\_lettingae\_TMO
Thermotoga\_lettingae\_TMO
Thermotoga\_lettingae\_TMO
Thermotoga\_lettingae\_TMO
Thermotoga\_lettingae\_TMO
Thermotoga\_lettingae\_TMO
Thermotoga\_lettingae\_TMO
Thermotoga\_lettingae\_TMO
Thermotoga\_lettingae\_TMO
no rank

1.861.861.861.861.861.861.861.861.861.861.861.861.861.861.861.861.861.861.861.86
11111111111111111111
2.098612.386292.0986110000000000000000
34310000000000000000
2336
species

1.861.861.861.861.861.861.861.861.861.861.861.861.861.861.861.861.861.861.861.86
11111111111111111111
2.098612.386292.0986110000000000000000
34310000000000000000
243274

Thermotoga\_maritima\_MSB8
Thermotoga\_maritima\_MSB8
Thermotoga\_maritima\_MSB8
Thermotoga\_maritima\_MSB8
Thermotoga\_maritima\_MSB8
Thermotoga\_maritima\_MSB8
Thermotoga\_maritima\_MSB8
Thermotoga\_maritima\_MSB8
Thermotoga\_maritima\_MSB8
Thermotoga\_maritima\_MSB8
Thermotoga\_maritima\_MSB8
Thermotoga\_maritima\_MSB8
Thermotoga\_maritima\_MSB8
Thermotoga\_maritima\_MSB8
Thermotoga\_maritima\_MSB8
Thermotoga\_maritima\_MSB8
Thermotoga\_maritima\_MSB8
Thermotoga\_maritima\_MSB8
Thermotoga\_maritima\_MSB8
Thermotoga\_maritima\_MSB8
no rank

1.881.881.881.881.881.881.881.881.881.881.881.881.881.881.881.881.881.881.881.88
11111111111111111111
1.693151.69315100000000000000000
22100000000000000000
2337
species

1.881.881.881.881.881.881.881.881.881.881.881.881.881.881.881.881.881.881.881.88
11111111111111111111
1.693151.69315100000000000000000
22100000000000000000
309803

Thermotoga\_neapolitana\_DSM\_4359
Thermotoga\_neapolitana\_DSM\_4359
Thermotoga\_neapolitana\_DSM\_4359
Thermotoga\_neapolitana\_DSM\_4359
Thermotoga\_neapolitana\_DSM\_4359
Thermotoga\_neapolitana\_DSM\_4359
Thermotoga\_neapolitana\_DSM\_4359
Thermotoga\_neapolitana\_DSM\_4359
Thermotoga\_neapolitana\_DSM\_4359
Thermotoga\_neapolitana\_DSM\_4359
Thermotoga\_neapolitana\_DSM\_4359
Thermotoga\_neapolitana\_DSM\_4359
Thermotoga\_neapolitana\_DSM\_4359
Thermotoga\_neapolitana\_DSM\_4359
Thermotoga\_neapolitana\_DSM\_4359
Thermotoga\_neapolitana\_DSM\_4359
Thermotoga\_neapolitana\_DSM\_4359
Thermotoga\_neapolitana\_DSM\_4359
Thermotoga\_neapolitana\_DSM\_4359
Thermotoga\_neapolitana\_DSM\_4359
no rank

2.172.172.172.172.172.172.172.172.172.172.172.172.172.172.172.172.172.172.172.17
11111111111111111111
3.772593.484913.39792.945910000000000000000
16121170000000000000000
28236
genus

2.172.172.172.172.172.172.172.172.172.172.172.172.172.172.172.172.172.172.172.17
11111111111111111111
3.772593.484913.39792.945910000000000000000
16121170000000000000000
69499
species

2.172.172.172.172.172.172.172.172.172.172.172.172.172.172.172.172.172.172.172.17
11111111111111111111
3.772593.484913.39792.945910000000000000000
16121170000000000000000
403833

Petrotoga\_mobilis\_SJ95
Petrotoga\_mobilis\_SJ95
Petrotoga\_mobilis\_SJ95
Petrotoga\_mobilis\_SJ95
Petrotoga\_mobilis\_SJ95
Petrotoga\_mobilis\_SJ95
Petrotoga\_mobilis\_SJ95
Petrotoga\_mobilis\_SJ95
Petrotoga\_mobilis\_SJ95
Petrotoga\_mobilis\_SJ95
Petrotoga\_mobilis\_SJ95
Petrotoga\_mobilis\_SJ95
Petrotoga\_mobilis\_SJ95
Petrotoga\_mobilis\_SJ95
Petrotoga\_mobilis\_SJ95
Petrotoga\_mobilis\_SJ95
Petrotoga\_mobilis\_SJ95
Petrotoga\_mobilis\_SJ95
Petrotoga\_mobilis\_SJ95
Petrotoga\_mobilis\_SJ95
no rank

2.242.242.242.242.242.242.242.242.242.242.242.242.242.242.242.242.242.242.242.24
11111111111111111111
3.639063.484913.39793.197220000000000000000
14121190000000000000000
160798
genus

2.242.242.242.242.242.242.242.242.242.242.242.242.242.242.242.242.242.242.242.24
11111111111111111111
3.639063.484913.39793.197220000000000000000
14121190000000000000000
149715
species

2.242.242.242.242.242.242.242.242.242.242.242.242.242.242.242.242.242.242.242.24
11111111111111111111
3.639063.484913.39793.197220000000000000000
14121190000000000000000
443254

Marinitoga\_piezophila\_KA3
Marinitoga\_piezophila\_KA3
Marinitoga\_piezophila\_KA3
Marinitoga\_piezophila\_KA3
Marinitoga\_piezophila\_KA3
Marinitoga\_piezophila\_KA3
Marinitoga\_piezophila\_KA3
Marinitoga\_piezophila\_KA3
Marinitoga\_piezophila\_KA3
Marinitoga\_piezophila\_KA3
Marinitoga\_piezophila\_KA3
Marinitoga\_piezophila\_KA3
Marinitoga\_piezophila\_KA3
Marinitoga\_piezophila\_KA3
Marinitoga\_piezophila\_KA3
Marinitoga\_piezophila\_KA3
Marinitoga\_piezophila\_KA3
Marinitoga\_piezophila\_KA3
Marinitoga\_piezophila\_KA3
Marinitoga\_piezophila\_KA3
no rank

2.982.982.982.982.982.982.982.982.982.982.982.982.982.982.982.982.982.982.982.98
11111111111111111111
4.36733.944443.890373.772591.693151.693151.693151.693151.693151.693151.693151.693151.693151.693151.693151.6931511.693151.693151
291918162222222222221221
1184396
genus

2.982.982.982.982.982.982.982.982.982.982.982.982.982.982.982.982.982.982.982.98
11111111111111111111
4.36733.944443.890373.772591.693151.693151.693151.693151.693151.693151.693151.693151.693151.693151.693151.6931511.693151.693151
291918162222222222221221
1184387
species

2.982.982.982.982.982.982.982.982.982.982.982.982.982.982.982.982.982.982.982.98
11111111111111111111
4.36733.944443.890373.772591.693151.693151.693151.693151.693151.693151.693151.693151.693151.693151.693151.6931511.693151.693151
291918162222222222221221
660470

Mesotoga\_prima\_MesG1.Ag.4.2
Mesotoga\_prima\_MesG1.Ag.4.2
Mesotoga\_prima\_MesG1.Ag.4.2
Mesotoga\_prima\_MesG1.Ag.4.2
Mesotoga\_prima\_MesG1.Ag.4.2
Mesotoga\_prima\_MesG1.Ag.4.2
Mesotoga\_prima\_MesG1.Ag.4.2
Mesotoga\_prima\_MesG1.Ag.4.2
Mesotoga\_prima\_MesG1.Ag.4.2
Mesotoga\_prima\_MesG1.Ag.4.2
Mesotoga\_prima\_MesG1.Ag.4.2
Mesotoga\_prima\_MesG1.Ag.4.2
Mesotoga\_prima\_MesG1.Ag.4.2
Mesotoga\_prima\_MesG1.Ag.4.2
Mesotoga\_prima\_MesG1.Ag.4.2
Mesotoga\_prima\_MesG1.Ag.4.2
Mesotoga\_prima\_MesG1.Ag.4.2
Mesotoga\_prima\_MesG1.Ag.4.2
Mesotoga\_prima\_MesG1.Ag.4.2
Mesotoga\_prima\_MesG1.Ag.4.2
no rank

13.9313.9313.9313.9313.9313.9313.9313.9313.9313.9313.9313.9313.9313.9313.9313.9313.9313.9313.9313.93
55555555555555555555
3.960473.82033.709033.661190.2637570.47810500.1557790.2637570.4781050000000000
191715140100010000000000
32066
phylum

13.9313.9313.9313.9313.9313.9313.9313.9313.9313.9313.9313.9313.9313.9313.9313.9313.9313.9313.9313.93
55555555555555555555
3.960473.82033.709033.661190.2637570.47810500.1557790.2637570.4781050000000000
191715140100010000000000
203490
class

13.9313.9313.9313.9313.9313.9313.9313.9313.9313.9313.9313.9313.9313.9313.9313.9313.9313.9313.9313.93
55555555555555555555
3.960473.82033.709033.661190.2637570.47810500.1557790.2637570.4781050000000000
191715140100010000000000
203491
order

5.305.305.305.305.305.305.305.305.305.305.305.305.305.305.305.305.305.305.305.30
22222222222222222222
3.974733.808393.65483.649460.6932320.40943400.4094340.6932320.4094340000000000
201714141101110000000000
203492
family

3.133.133.133.133.133.133.133.133.133.133.133.133.133.133.133.133.133.133.133.13
11111111111111111111
3.995733.833213.772593.708050000000000000000
201716150000000000000000
167639
genus

3.133.133.133.133.133.133.133.133.133.133.133.133.133.133.133.133.133.133.133.13
11111111111111111111
3.995733.833213.772593.708050000000000000000
201716150000000000000000
167642
species

3.133.133.133.133.133.133.133.133.133.133.133.133.133.133.133.133.133.133.133.13
11111111111111111111
3.995733.833213.772593.708050000000000000000
201716150000000000000000
572544

Ilyobacter\_polytropus\_DSM\_2926
Ilyobacter\_polytropus\_DSM\_2926
Ilyobacter\_polytropus\_DSM\_2926
Ilyobacter\_polytropus\_DSM\_2926
Ilyobacter\_polytropus\_DSM\_2926
Ilyobacter\_polytropus\_DSM\_2926
Ilyobacter\_polytropus\_DSM\_2926
Ilyobacter\_polytropus\_DSM\_2926
Ilyobacter\_polytropus\_DSM\_2926
Ilyobacter\_polytropus\_DSM\_2926
Ilyobacter\_polytropus\_DSM\_2926
Ilyobacter\_polytropus\_DSM\_2926
Ilyobacter\_polytropus\_DSM\_2926
Ilyobacter\_polytropus\_DSM\_2926
Ilyobacter\_polytropus\_DSM\_2926
Ilyobacter\_polytropus\_DSM\_2926
Ilyobacter\_polytropus\_DSM\_2926
Ilyobacter\_polytropus\_DSM\_2926
Ilyobacter\_polytropus\_DSM\_2926
Ilyobacter\_polytropus\_DSM\_2926
no rank

2.172.172.172.172.172.172.172.172.172.172.172.172.172.172.172.172.172.172.172.17
11111111111111111111
3.944443.772593.484913.564951.693151011.6931510000000000
191612132101210000000000
848
genus

2.172.172.172.172.172.172.172.172.172.172.172.172.172.172.172.172.172.172.172.17
11111111111111111111
3.944443.772593.484913.564951.693151011.6931510000000000
191612132101210000000000
851
species

2.172.172.172.172.172.172.172.172.172.172.172.172.172.172.172.172.172.172.172.17
11111111111111111111
3.944443.772593.484913.564951.693151011.6931510000000000
191612132101210000000000
76856
subspecies

2.172.172.172.172.172.172.172.172.172.172.172.172.172.172.172.172.172.172.172.17
11111111111111111111
3.944443.772593.484913.564951.693151011.6931510000000000
191612132101210000000000
190304

Fusobacterium\_nucleatum\_subsp.\_nucleatum\_ATCC\_25586
Fusobacterium\_nucleatum\_subsp.\_nucleatum\_ATCC\_25586
Fusobacterium\_nucleatum\_subsp.\_nucleatum\_ATCC\_25586
Fusobacterium\_nucleatum\_subsp.\_nucleatum\_ATCC\_25586
Fusobacterium\_nucleatum\_subsp.\_nucleatum\_ATCC\_25586
Fusobacterium\_nucleatum\_subsp.\_nucleatum\_ATCC\_25586
Fusobacterium\_nucleatum\_subsp.\_nucleatum\_ATCC\_25586
Fusobacterium\_nucleatum\_subsp.\_nucleatum\_ATCC\_25586
Fusobacterium\_nucleatum\_subsp.\_nucleatum\_ATCC\_25586
Fusobacterium\_nucleatum\_subsp.\_nucleatum\_ATCC\_25586
Fusobacterium\_nucleatum\_subsp.\_nucleatum\_ATCC\_25586
Fusobacterium\_nucleatum\_subsp.\_nucleatum\_ATCC\_25586
Fusobacterium\_nucleatum\_subsp.\_nucleatum\_ATCC\_25586
Fusobacterium\_nucleatum\_subsp.\_nucleatum\_ATCC\_25586
Fusobacterium\_nucleatum\_subsp.\_nucleatum\_ATCC\_25586
Fusobacterium\_nucleatum\_subsp.\_nucleatum\_ATCC\_25586
Fusobacterium\_nucleatum\_subsp.\_nucleatum\_ATCC\_25586
Fusobacterium\_nucleatum\_subsp.\_nucleatum\_ATCC\_25586
Fusobacterium\_nucleatum\_subsp.\_nucleatum\_ATCC\_25586
Fusobacterium\_nucleatum\_subsp.\_nucleatum\_ATCC\_25586
no rank

8.638.638.638.638.638.638.638.638.638.638.638.638.638.638.638.638.638.638.638.63
33333333333333333333
3.951723.827623.742343.6683900.5202780000.5202780000000000
191716140100010000000000
1129771
family

1.671.671.671.671.671.671.671.671.671.671.671.671.671.671.671.671.671.671.671.67
11111111111111111111
4.218883.708053.639063.484910000000000000000
251514120000000000000000
34104
genus

1.671.671.671.671.671.671.671.671.671.671.671.671.671.671.671.671.671.671.671.67
11111111111111111111
4.218883.708053.639063.484910000000000000000
251514120000000000000000
34105
species

1.671.671.671.671.671.671.671.671.671.671.671.671.671.671.671.671.671.671.671.67
11111111111111111111
4.218883.708053.639063.484910000000000000000
251514120000000000000000
519441

Streptobacillus\_moniliformis\_DSM\_12112
Streptobacillus\_moniliformis\_DSM\_12112
Streptobacillus\_moniliformis\_DSM\_12112
Streptobacillus\_moniliformis\_DSM\_12112
Streptobacillus\_moniliformis\_DSM\_12112
Streptobacillus\_moniliformis\_DSM\_12112
Streptobacillus\_moniliformis\_DSM\_12112
Streptobacillus\_moniliformis\_DSM\_12112
Streptobacillus\_moniliformis\_DSM\_12112
Streptobacillus\_moniliformis\_DSM\_12112
Streptobacillus\_moniliformis\_DSM\_12112
Streptobacillus\_moniliformis\_DSM\_12112
Streptobacillus\_moniliformis\_DSM\_12112
Streptobacillus\_moniliformis\_DSM\_12112
Streptobacillus\_moniliformis\_DSM\_12112
Streptobacillus\_moniliformis\_DSM\_12112
Streptobacillus\_moniliformis\_DSM\_12112
Streptobacillus\_moniliformis\_DSM\_12112
Streptobacillus\_moniliformis\_DSM\_12112
Streptobacillus\_moniliformis\_DSM\_12112
no rank

2.472.472.472.472.472.472.472.472.472.472.472.472.472.472.472.472.472.472.472.47
11111111111111111111
3.079443.197222.945912.791760000000000000000
89760000000000000000
32067
genus

2.472.472.472.472.472.472.472.472.472.472.472.472.472.472.472.472.472.472.472.47
11111111111111111111
3.079443.197222.945912.791760000000000000000
89760000000000000000
40542
species

2.472.472.472.472.472.472.472.472.472.472.472.472.472.472.472.472.472.472.472.47
11111111111111111111
3.079443.197222.945912.791760000000000000000
89760000000000000000
523794

Leptotrichia\_buccalis\_C-1013-b
Leptotrichia\_buccalis\_C-1013-b
Leptotrichia\_buccalis\_C-1013-b
Leptotrichia\_buccalis\_C-1013-b
Leptotrichia\_buccalis\_C-1013-b
Leptotrichia\_buccalis\_C-1013-b
Leptotrichia\_buccalis\_C-1013-b
Leptotrichia\_buccalis\_C-1013-b
Leptotrichia\_buccalis\_C-1013-b
Leptotrichia\_buccalis\_C-1013-b
Leptotrichia\_buccalis\_C-1013-b
Leptotrichia\_buccalis\_C-1013-b
Leptotrichia\_buccalis\_C-1013-b
Leptotrichia\_buccalis\_C-1013-b
Leptotrichia\_buccalis\_C-1013-b
Leptotrichia\_buccalis\_C-1013-b
Leptotrichia\_buccalis\_C-1013-b
Leptotrichia\_buccalis\_C-1013-b
Leptotrichia\_buccalis\_C-1013-b
Leptotrichia\_buccalis\_C-1013-b
no rank

4.494.494.494.494.494.494.494.494.494.494.494.494.494.494.494.494.494.494.494.49
11111111111111111111
4.33224.218884.218884.218880100010000000000
282525250100010000000000
32068
genus

4.494.494.494.494.494.494.494.494.494.494.494.494.494.494.494.494.494.494.494.49
11111111111111111111
4.33224.218884.218884.218880100010000000000
282525250100010000000000
826
species

4.494.494.494.494.494.494.494.494.494.494.494.494.494.494.494.494.494.494.494.49
11111111111111111111
4.33224.218884.218884.218880100010000000000
282525250100010000000000
526218

Sebaldella\_termitidis\_ATCC\_33386
Sebaldella\_termitidis\_ATCC\_33386
Sebaldella\_termitidis\_ATCC\_33386
Sebaldella\_termitidis\_ATCC\_33386
Sebaldella\_termitidis\_ATCC\_33386
Sebaldella\_termitidis\_ATCC\_33386
Sebaldella\_termitidis\_ATCC\_33386
Sebaldella\_termitidis\_ATCC\_33386
Sebaldella\_termitidis\_ATCC\_33386
Sebaldella\_termitidis\_ATCC\_33386
Sebaldella\_termitidis\_ATCC\_33386
Sebaldella\_termitidis\_ATCC\_33386
Sebaldella\_termitidis\_ATCC\_33386
Sebaldella\_termitidis\_ATCC\_33386
Sebaldella\_termitidis\_ATCC\_33386
Sebaldella\_termitidis\_ATCC\_33386
Sebaldella\_termitidis\_ATCC\_33386
Sebaldella\_termitidis\_ATCC\_33386
Sebaldella\_termitidis\_ATCC\_33386
Sebaldella\_termitidis\_ATCC\_33386
no rank

2.792.792.792.792.792.792.792.792.792.792.792.792.792.792.792.792.792.792.792.79
22222222222222222222
2.309172.309172.217192.217190000000000000000
44330000000000000000
74152
phylum

1.151.151.151.151.151.151.151.151.151.151.151.151.151.151.151.151.151.151.151.15
11111111111111111111
2.609442.609442.386292.386290000000000000000
55440000000000000000
99260
no rank

1.151.151.151.151.151.151.151.151.151.151.151.151.151.151.151.151.151.151.151.15
11111111111111111111
2.609442.609442.386292.386290000000000000000
55440000000000000000
167965
species

1.151.151.151.151.151.151.151.151.151.151.151.151.151.151.151.151.151.151.151.15
11111111111111111111
2.609442.609442.386292.386290000000000000000
55440000000000000000
471821

uncultured\_Termite\_group\_1\_bacterium\_phylotype\_Rs-D17
uncultured\_Termite\_group\_1\_bacterium\_phylotype\_Rs-D17
uncultured\_Termite\_group\_1\_bacterium\_phylotype\_Rs-D17
uncultured\_Termite\_group\_1\_bacterium\_phylotype\_Rs-D17
uncultured\_Termite\_group\_1\_bacterium\_phylotype\_Rs-D17
uncultured\_Termite\_group\_1\_bacterium\_phylotype\_Rs-D17
uncultured\_Termite\_group\_1\_bacterium\_phylotype\_Rs-D17
uncultured\_Termite\_group\_1\_bacterium\_phylotype\_Rs-D17
uncultured\_Termite\_group\_1\_bacterium\_phylotype\_Rs-D17
uncultured\_Termite\_group\_1\_bacterium\_phylotype\_Rs-D17
uncultured\_Termite\_group\_1\_bacterium\_phylotype\_Rs-D17
uncultured\_Termite\_group\_1\_bacterium\_phylotype\_Rs-D17
uncultured\_Termite\_group\_1\_bacterium\_phylotype\_Rs-D17
uncultured\_Termite\_group\_1\_bacterium\_phylotype\_Rs-D17
uncultured\_Termite\_group\_1\_bacterium\_phylotype\_Rs-D17
uncultured\_Termite\_group\_1\_bacterium\_phylotype\_Rs-D17
uncultured\_Termite\_group\_1\_bacterium\_phylotype\_Rs-D17
uncultured\_Termite\_group\_1\_bacterium\_phylotype\_Rs-D17
uncultured\_Termite\_group\_1\_bacterium\_phylotype\_Rs-D17
uncultured\_Termite\_group\_1\_bacterium\_phylotype\_Rs-D17
no rank

1.641.641.641.641.641.641.641.641.641.641.641.641.641.641.641.641.641.641.641.64
11111111111111111111
2.098612.098612.098612.098610000000000000000
33330000000000000000
641853
class

1.641.641.641.641.641.641.641.641.641.641.641.641.641.641.641.641.641.641.641.64
11111111111111111111
2.098612.098612.098612.098610000000000000000
33330000000000000000
641854
order

1.641.641.641.641.641.641.641.641.641.641.641.641.641.641.641.641.641.641.641.64
11111111111111111111
2.098612.098612.098612.098610000000000000000
33330000000000000000
641876
family

1.641.641.641.641.641.641.641.641.641.641.641.641.641.641.641.641.641.641.641.64
11111111111111111111
2.098612.098612.098612.098610000000000000000
33330000000000000000
423604
genus

1.641.641.641.641.641.641.641.641.641.641.641.641.641.641.641.641.641.641.641.64
11111111111111111111
2.098612.098612.098612.098610000000000000000
33330000000000000000
423605
species

1.641.641.641.641.641.641.641.641.641.641.641.641.641.641.641.641.641.641.641.64
11111111111111111111
2.098612.098612.098612.098610000000000000000
33330000000000000000
445932

Elusimicrobium\_minutum\_Pei191
Elusimicrobium\_minutum\_Pei191
Elusimicrobium\_minutum\_Pei191
Elusimicrobium\_minutum\_Pei191
Elusimicrobium\_minutum\_Pei191
Elusimicrobium\_minutum\_Pei191
Elusimicrobium\_minutum\_Pei191
Elusimicrobium\_minutum\_Pei191
Elusimicrobium\_minutum\_Pei191
Elusimicrobium\_minutum\_Pei191
Elusimicrobium\_minutum\_Pei191
Elusimicrobium\_minutum\_Pei191
Elusimicrobium\_minutum\_Pei191
Elusimicrobium\_minutum\_Pei191
Elusimicrobium\_minutum\_Pei191
Elusimicrobium\_minutum\_Pei191
Elusimicrobium\_minutum\_Pei191
Elusimicrobium\_minutum\_Pei191
Elusimicrobium\_minutum\_Pei191
Elusimicrobium\_minutum\_Pei191
no rank

4.644.644.644.644.644.644.644.644.644.644.644.644.644.644.644.644.644.644.644.64
11111111111111111111
00000000000000000000
00000000000000000000
142182
phylum

4.644.644.644.644.644.644.644.644.644.644.644.644.644.644.644.644.644.644.644.64
11111111111111111111
00000000000000000000
00000000000000000000
219685
class

4.644.644.644.644.644.644.644.644.644.644.644.644.644.644.644.644.644.644.644.64
11111111111111111111
00000000000000000000
00000000000000000000
219686
order

4.644.644.644.644.644.644.644.644.644.644.644.644.644.644.644.644.644.644.644.64
11111111111111111111
00000000000000000000
00000000000000000000
219687
family

4.644.644.644.644.644.644.644.644.644.644.644.644.644.644.644.644.644.644.644.64
11111111111111111111
00000000000000000000
00000000000000000000
173479
genus

4.644.644.644.644.644.644.644.644.644.644.644.644.644.644.644.644.644.644.644.64
11111111111111111111
00000000000000000000
00000000000000000000
173480
species

4.644.644.644.644.644.644.644.644.644.644.644.644.644.644.644.644.644.644.644.64
11111111111111111111
00000000000000000000
00000000000000000000
379066

Gemmatimonas\_aurantiaca\_T-27
Gemmatimonas\_aurantiaca\_T-27
Gemmatimonas\_aurantiaca\_T-27
Gemmatimonas\_aurantiaca\_T-27
Gemmatimonas\_aurantiaca\_T-27
Gemmatimonas\_aurantiaca\_T-27
Gemmatimonas\_aurantiaca\_T-27
Gemmatimonas\_aurantiaca\_T-27
Gemmatimonas\_aurantiaca\_T-27
Gemmatimonas\_aurantiaca\_T-27
Gemmatimonas\_aurantiaca\_T-27
Gemmatimonas\_aurantiaca\_T-27
Gemmatimonas\_aurantiaca\_T-27
Gemmatimonas\_aurantiaca\_T-27
Gemmatimonas\_aurantiaca\_T-27
Gemmatimonas\_aurantiaca\_T-27
Gemmatimonas\_aurantiaca\_T-27
Gemmatimonas\_aurantiaca\_T-27
Gemmatimonas\_aurantiaca\_T-27
Gemmatimonas\_aurantiaca\_T-27
no rank

10.5110.5110.5110.5110.5110.5110.5110.5110.5110.5110.5110.5110.5110.5110.5110.5110.5110.5110.5110.51
44444444444444444444
4.296573.942973.728073.599431.525630.5187380.5187380.5187380.5187380.5187380.5187380.51873800000000
271915132111111100000000
200930
phylum

10.5110.5110.5110.5110.5110.5110.5110.5110.5110.5110.5110.5110.5110.5110.5110.5110.5110.5110.5110.51
44444444444444444444
4.296573.942973.728073.599431.525630.5187380.5187380.5187380.5187380.5187380.5187380.51873800000000
271915132111111100000000
68337
class

10.5110.5110.5110.5110.5110.5110.5110.5110.5110.5110.5110.5110.5110.5110.5110.5110.5110.5110.5110.51
44444444444444444444
4.296573.942973.728073.599431.525630.5187380.5187380.5187380.5187380.5187380.5187380.51873800000000
271915132111111100000000
191393
order

10.5110.5110.5110.5110.5110.5110.5110.5110.5110.5110.5110.5110.5110.5110.5110.5110.5110.5110.5110.51
44444444444444444444
4.296573.942973.728073.599431.525630.5187380.5187380.5187380.5187380.5187380.5187380.51873800000000
271915132111111100000000
191394
family

3.223.223.223.223.223.223.223.223.223.223.223.223.223.223.223.223.223.223.223.22
11111111111111111111
4.496514.295844.091044.218881.693151.693151.693151.693151.693151.693151.693151.6931500000000
332722252222222200000000
117999
genus

3.223.223.223.223.223.223.223.223.223.223.223.223.223.223.223.223.223.223.223.22
11111111111111111111
4.496514.295844.091044.218881.693151.693151.693151.693151.693151.693151.693151.6931500000000
332722252222222200000000
118000
species

3.223.223.223.223.223.223.223.223.223.223.223.223.223.223.223.223.223.223.223.22
11111111111111111111
4.496514.295844.091044.218881.693151.693151.693151.693151.693151.693151.693151.6931500000000
332722252222222200000000
522772

Denitrovibrio\_acetiphilus\_DSM\_12809
Denitrovibrio\_acetiphilus\_DSM\_12809
Denitrovibrio\_acetiphilus\_DSM\_12809
Denitrovibrio\_acetiphilus\_DSM\_12809
Denitrovibrio\_acetiphilus\_DSM\_12809
Denitrovibrio\_acetiphilus\_DSM\_12809
Denitrovibrio\_acetiphilus\_DSM\_12809
Denitrovibrio\_acetiphilus\_DSM\_12809
Denitrovibrio\_acetiphilus\_DSM\_12809
Denitrovibrio\_acetiphilus\_DSM\_12809
Denitrovibrio\_acetiphilus\_DSM\_12809
Denitrovibrio\_acetiphilus\_DSM\_12809
Denitrovibrio\_acetiphilus\_DSM\_12809
Denitrovibrio\_acetiphilus\_DSM\_12809
Denitrovibrio\_acetiphilus\_DSM\_12809
Denitrovibrio\_acetiphilus\_DSM\_12809
Denitrovibrio\_acetiphilus\_DSM\_12809
Denitrovibrio\_acetiphilus\_DSM\_12809
Denitrovibrio\_acetiphilus\_DSM\_12809
Denitrovibrio\_acetiphilus\_DSM\_12809
no rank

2.532.532.532.532.532.532.532.532.532.532.532.532.532.532.532.532.532.532.532.53
11111111111111111111
5.219514.828644.737674.737671.69315000000000000000
684642422000000000000000
2351
genus

2.532.532.532.532.532.532.532.532.532.532.532.532.532.532.532.532.532.532.532.53
11111111111111111111
5.219514.828644.737674.737671.69315000000000000000
684642422000000000000000
2352
species

2.532.532.532.532.532.532.532.532.532.532.532.532.532.532.532.532.532.532.532.53
11111111111111111111
5.219514.828644.737674.737671.69315000000000000000
684642422000000000000000
717231

Flexistipes\_sinusarabici\_DSM\_4947
Flexistipes\_sinusarabici\_DSM\_4947
Flexistipes\_sinusarabici\_DSM\_4947
Flexistipes\_sinusarabici\_DSM\_4947
Flexistipes\_sinusarabici\_DSM\_4947
Flexistipes\_sinusarabici\_DSM\_4947
Flexistipes\_sinusarabici\_DSM\_4947
Flexistipes\_sinusarabici\_DSM\_4947
Flexistipes\_sinusarabici\_DSM\_4947
Flexistipes\_sinusarabici\_DSM\_4947
Flexistipes\_sinusarabici\_DSM\_4947
Flexistipes\_sinusarabici\_DSM\_4947
Flexistipes\_sinusarabici\_DSM\_4947
Flexistipes\_sinusarabici\_DSM\_4947
Flexistipes\_sinusarabici\_DSM\_4947
Flexistipes\_sinusarabici\_DSM\_4947
Flexistipes\_sinusarabici\_DSM\_4947
Flexistipes\_sinusarabici\_DSM\_4947
Flexistipes\_sinusarabici\_DSM\_4947
Flexistipes\_sinusarabici\_DSM\_4947
no rank

2.542.542.542.542.542.542.542.542.542.542.542.542.542.542.542.542.542.542.542.54
11111111111111111111
3.833213.484912.945912.386291000000000000000
1712741000000000000000
53572
genus

2.542.542.542.542.542.542.542.542.542.542.542.542.542.542.542.542.542.542.542.54
11111111111111111111
3.833213.484912.945912.386291000000000000000
1712741000000000000000
197162
species

2.542.542.542.542.542.542.542.542.542.542.542.542.542.542.542.542.542.542.542.54
11111111111111111111
3.833213.484912.945912.386291000000000000000
1712741000000000000000
639282

Deferribacter\_desulfuricans\_SSM1
Deferribacter\_desulfuricans\_SSM1
Deferribacter\_desulfuricans\_SSM1
Deferribacter\_desulfuricans\_SSM1
Deferribacter\_desulfuricans\_SSM1
Deferribacter\_desulfuricans\_SSM1
Deferribacter\_desulfuricans\_SSM1
Deferribacter\_desulfuricans\_SSM1
Deferribacter\_desulfuricans\_SSM1
Deferribacter\_desulfuricans\_SSM1
Deferribacter\_desulfuricans\_SSM1
Deferribacter\_desulfuricans\_SSM1
Deferribacter\_desulfuricans\_SSM1
Deferribacter\_desulfuricans\_SSM1
Deferribacter\_desulfuricans\_SSM1
Deferribacter\_desulfuricans\_SSM1
Deferribacter\_desulfuricans\_SSM1
Deferribacter\_desulfuricans\_SSM1
Deferribacter\_desulfuricans\_SSM1
Deferribacter\_desulfuricans\_SSM1
no rank

2.222.222.222.222.222.222.222.222.222.222.222.222.222.222.222.222.222.222.222.22
11111111111111111111
3.484912.945912.945912.791761.69315000000000000000
127762000000000000000
545865
genus

2.222.222.222.222.222.222.222.222.222.222.222.222.222.222.222.222.222.222.222.22
11111111111111111111
3.484912.945912.945912.791761.69315000000000000000
127762000000000000000
477976
species

2.222.222.222.222.222.222.222.222.222.222.222.222.222.222.222.222.222.222.222.22
11111111111111111111
3.484912.945912.945912.791761.69315000000000000000
127762000000000000000
768670

Calditerrivibrio\_nitroreducens\_DSM\_19672
Calditerrivibrio\_nitroreducens\_DSM\_19672
Calditerrivibrio\_nitroreducens\_DSM\_19672
Calditerrivibrio\_nitroreducens\_DSM\_19672
Calditerrivibrio\_nitroreducens\_DSM\_19672
Calditerrivibrio\_nitroreducens\_DSM\_19672
Calditerrivibrio\_nitroreducens\_DSM\_19672
Calditerrivibrio\_nitroreducens\_DSM\_19672
Calditerrivibrio\_nitroreducens\_DSM\_19672
Calditerrivibrio\_nitroreducens\_DSM\_19672
Calditerrivibrio\_nitroreducens\_DSM\_19672
Calditerrivibrio\_nitroreducens\_DSM\_19672
Calditerrivibrio\_nitroreducens\_DSM\_19672
Calditerrivibrio\_nitroreducens\_DSM\_19672
Calditerrivibrio\_nitroreducens\_DSM\_19672
Calditerrivibrio\_nitroreducens\_DSM\_19672
Calditerrivibrio\_nitroreducens\_DSM\_19672
Calditerrivibrio\_nitroreducens\_DSM\_19672
Calditerrivibrio\_nitroreducens\_DSM\_19672
Calditerrivibrio\_nitroreducens\_DSM\_19672
no rank

11.2911.2911.2911.2911.2911.2911.2911.2911.2911.2911.2911.2911.2911.2911.2911.2911.2911.2911.2911.29
44444444444444444444
2.863362.603572.618352.38340.3906110.1771480.1771480.1771480.1771480.1771480.1771480.1771480.1771480.1771480.1771480.1771480.1771480.1771480.1771480.177148
65541000000000000000
40117
phylum

11.2911.2911.2911.2911.2911.2911.2911.2911.2911.2911.2911.2911.2911.2911.2911.2911.2911.2911.2911.29
44444444444444444444
2.863362.603572.618352.38340.3906110.1771480.1771480.1771480.1771480.1771480.1771480.1771480.1771480.1771480.1771480.1771480.1771480.1771480.1771480.177148
65541000000000000000
203693
class

11.2911.2911.2911.2911.2911.2911.2911.2911.2911.2911.2911.2911.2911.2911.2911.2911.2911.2911.2911.29
44444444444444444444
2.863362.603572.618352.38340.3906110.1771480.1771480.1771480.1771480.1771480.1771480.1771480.1771480.1771480.1771480.1771480.1771480.1771480.1771480.177148
65541000000000000000
189778
order

11.2911.2911.2911.2911.2911.2911.2911.2911.2911.2911.2911.2911.2911.2911.2911.2911.2911.2911.2911.29
44444444444444444444
2.863362.603572.618352.38340.3906110.1771480.1771480.1771480.1771480.1771480.1771480.1771480.1771480.1771480.1771480.1771480.1771480.1771480.1771480.177148
65541000000000000000
189779
family

2.002.002.002.002.002.002.002.002.002.002.002.002.002.002.002.002.002.002.002.00
11111111111111111111
1.693151111111111111111111
21111111111111111111
28261
genus

2.002.002.002.002.002.002.002.002.002.002.002.002.002.002.002.002.002.002.002.00
11111111111111111111
1.693151111111111111111111
21111111111111111111
28262
species

2.002.002.002.002.002.002.002.002.002.002.002.002.002.002.002.002.002.002.002.00
11111111111111111111
1.693151111111111111111111
21111111111111111111
289376

Thermodesulfovibrio\_yellowstonii\_DSM\_11347
Thermodesulfovibrio\_yellowstonii\_DSM\_11347
Thermodesulfovibrio\_yellowstonii\_DSM\_11347
Thermodesulfovibrio\_yellowstonii\_DSM\_11347
Thermodesulfovibrio\_yellowstonii\_DSM\_11347
Thermodesulfovibrio\_yellowstonii\_DSM\_11347
Thermodesulfovibrio\_yellowstonii\_DSM\_11347
Thermodesulfovibrio\_yellowstonii\_DSM\_11347
Thermodesulfovibrio\_yellowstonii\_DSM\_11347
Thermodesulfovibrio\_yellowstonii\_DSM\_11347
Thermodesulfovibrio\_yellowstonii\_DSM\_11347
Thermodesulfovibrio\_yellowstonii\_DSM\_11347
Thermodesulfovibrio\_yellowstonii\_DSM\_11347
Thermodesulfovibrio\_yellowstonii\_DSM\_11347
Thermodesulfovibrio\_yellowstonii\_DSM\_11347
Thermodesulfovibrio\_yellowstonii\_DSM\_11347
Thermodesulfovibrio\_yellowstonii\_DSM\_11347
Thermodesulfovibrio\_yellowstonii\_DSM\_11347
Thermodesulfovibrio\_yellowstonii\_DSM\_11347
Thermodesulfovibrio\_yellowstonii\_DSM\_11347
no rank

4.324.324.324.324.324.324.324.324.324.324.324.324.324.324.324.324.324.324.324.32
11111111111111111111
2.791762.386292.791762.098610000000000000000
64630000000000000000
1234
genus

4.324.324.324.324.324.324.324.324.324.324.324.324.324.324.324.324.324.324.324.32
11111111111111111111
2.791762.386292.791762.098610000000000000000
64630000000000000000
330214

Candidatus\_Nitrospira\_defluvii
Candidatus\_Nitrospira\_defluvii
Candidatus\_Nitrospira\_defluvii
Candidatus\_Nitrospira\_defluvii
Candidatus\_Nitrospira\_defluvii
Candidatus\_Nitrospira\_defluvii
Candidatus\_Nitrospira\_defluvii
Candidatus\_Nitrospira\_defluvii
Candidatus\_Nitrospira\_defluvii
Candidatus\_Nitrospira\_defluvii
Candidatus\_Nitrospira\_defluvii
Candidatus\_Nitrospira\_defluvii
Candidatus\_Nitrospira\_defluvii
Candidatus\_Nitrospira\_defluvii
Candidatus\_Nitrospira\_defluvii
Candidatus\_Nitrospira\_defluvii
Candidatus\_Nitrospira\_defluvii
Candidatus\_Nitrospira\_defluvii
Candidatus\_Nitrospira\_defluvii
Candidatus\_Nitrospira\_defluvii
species

4.974.974.974.974.974.974.974.974.974.974.974.974.974.974.974.974.974.974.974.97
22222222222222222222
3.39653.437733.118873.187650.484909000000000000000
1111891000000000000000
179
genus

2.562.562.562.562.562.562.562.562.562.562.562.562.562.562.562.562.562.562.562.56
11111111111111111111
3.484913.564952.945913.079440000000000000000
1213780000000000000000
655606
species group

2.562.562.562.562.562.562.562.562.562.562.562.562.562.562.562.562.562.562.562.56
11111111111111111111
3.484913.564952.945913.079440000000000000000
1213780000000000000000
180
species

2.562.562.562.562.562.562.562.562.562.562.562.562.562.562.562.562.562.562.562.56
11111111111111111111
3.484913.564952.945913.079440000000000000000
1213780000000000000000
1162668

Leptospirillum\_ferrooxidans\_C2-3
Leptospirillum\_ferrooxidans\_C2-3
Leptospirillum\_ferrooxidans\_C2-3
Leptospirillum\_ferrooxidans\_C2-3
Leptospirillum\_ferrooxidans\_C2-3
Leptospirillum\_ferrooxidans\_C2-3
Leptospirillum\_ferrooxidans\_C2-3
Leptospirillum\_ferrooxidans\_C2-3
Leptospirillum\_ferrooxidans\_C2-3
Leptospirillum\_ferrooxidans\_C2-3
Leptospirillum\_ferrooxidans\_C2-3
Leptospirillum\_ferrooxidans\_C2-3
Leptospirillum\_ferrooxidans\_C2-3
Leptospirillum\_ferrooxidans\_C2-3
Leptospirillum\_ferrooxidans\_C2-3
Leptospirillum\_ferrooxidans\_C2-3
Leptospirillum\_ferrooxidans\_C2-3
Leptospirillum\_ferrooxidans\_C2-3
Leptospirillum\_ferrooxidans\_C2-3
Leptospirillum\_ferrooxidans\_C2-3
no rank

2.412.412.412.412.412.412.412.412.412.412.412.412.412.412.412.412.412.412.412.41
11111111111111111111
3.302593.302593.302593.302591000000000000000
101010101000000000000000
261386
species group

2.412.412.412.412.412.412.412.412.412.412.412.412.412.412.412.412.412.412.412.41
11111111111111111111
3.302593.302593.302593.302591000000000000000
101010101000000000000000
178606
species

2.412.412.412.412.412.412.412.412.412.412.412.412.412.412.412.412.412.412.412.41
11111111111111111111
3.302593.302593.302593.302591000000000000000
101010101000000000000000
1048260

Leptospirillum\_ferriphilum\_ML-04
Leptospirillum\_ferriphilum\_ML-04
Leptospirillum\_ferriphilum\_ML-04
Leptospirillum\_ferriphilum\_ML-04
Leptospirillum\_ferriphilum\_ML-04
Leptospirillum\_ferriphilum\_ML-04
Leptospirillum\_ferriphilum\_ML-04
Leptospirillum\_ferriphilum\_ML-04
Leptospirillum\_ferriphilum\_ML-04
Leptospirillum\_ferriphilum\_ML-04
Leptospirillum\_ferriphilum\_ML-04
Leptospirillum\_ferriphilum\_ML-04
Leptospirillum\_ferriphilum\_ML-04
Leptospirillum\_ferriphilum\_ML-04
Leptospirillum\_ferriphilum\_ML-04
Leptospirillum\_ferriphilum\_ML-04
Leptospirillum\_ferriphilum\_ML-04
Leptospirillum\_ferriphilum\_ML-04
Leptospirillum\_ferriphilum\_ML-04
Leptospirillum\_ferriphilum\_ML-04
no rank

1034.181034.181034.181034.181034.181034.181034.181034.181034.181034.181034.181034.181034.181034.181034.181034.181034.181034.181034.181034.18
234234234234234234234234234234234234234234234234234234234234
3.439423.176223.055732.962130.9661290.6516480.5594560.5679410.7549050.5708430.5070590.5177380.3836930.3468140.3295070.3166180.2573820.2124250.2154150.222013
119871111111111110000
201174
phylum

1034.181034.181034.181034.181034.181034.181034.181034.181034.181034.181034.181034.181034.181034.181034.181034.181034.181034.181034.181034.18
234234234234234234234234234234234234234234234234234234234234
3.439423.176223.055732.962130.9661290.6516480.5594560.5679410.7549050.5708430.5070590.5177380.3836930.3468140.3295070.3166180.2573820.2124250.2154150.222013
119871111111111110000
1760
class

2.162.162.162.162.162.162.162.162.162.162.162.162.162.162.162.162.162.162.162.16
11111111111111111111
3.39792.945912.609442.945910000000000000000
117570000000000000000
84992
subclass

2.162.162.162.162.162.162.162.162.162.162.162.162.162.162.162.162.162.162.162.16
11111111111111111111
3.39792.945912.609442.945910000000000000000
117570000000000000000
84993
order

2.162.162.162.162.162.162.162.162.162.162.162.162.162.162.162.162.162.162.162.16
11111111111111111111
3.39792.945912.609442.945910000000000000000
117570000000000000000
255726
suborder

2.162.162.162.162.162.162.162.162.162.162.162.162.162.162.162.162.162.162.162.16
11111111111111111111
3.39792.945912.609442.945910000000000000000
117570000000000000000
84994
family

2.162.162.162.162.162.162.162.162.162.162.162.162.162.162.162.162.162.162.162.16
11111111111111111111
3.39792.945912.609442.945910000000000000000
117570000000000000000
53634
genus

2.162.162.162.162.162.162.162.162.162.162.162.162.162.162.162.162.162.162.162.16
11111111111111111111
3.39792.945912.609442.945910000000000000000
117570000000000000000
53635
species

2.162.162.162.162.162.162.162.162.162.162.162.162.162.162.162.162.162.162.162.16
11111111111111111111
3.39792.945912.609442.945910000000000000000
117570000000000000000
525909

Acidimicrobium\_ferrooxidans\_DSM\_10331
Acidimicrobium\_ferrooxidans\_DSM\_10331
Acidimicrobium\_ferrooxidans\_DSM\_10331
Acidimicrobium\_ferrooxidans\_DSM\_10331
Acidimicrobium\_ferrooxidans\_DSM\_10331
Acidimicrobium\_ferrooxidans\_DSM\_10331
Acidimicrobium\_ferrooxidans\_DSM\_10331
Acidimicrobium\_ferrooxidans\_DSM\_10331
Acidimicrobium\_ferrooxidans\_DSM\_10331
Acidimicrobium\_ferrooxidans\_DSM\_10331
Acidimicrobium\_ferrooxidans\_DSM\_10331
Acidimicrobium\_ferrooxidans\_DSM\_10331
Acidimicrobium\_ferrooxidans\_DSM\_10331
Acidimicrobium\_ferrooxidans\_DSM\_10331
Acidimicrobium\_ferrooxidans\_DSM\_10331
Acidimicrobium\_ferrooxidans\_DSM\_10331
Acidimicrobium\_ferrooxidans\_DSM\_10331
Acidimicrobium\_ferrooxidans\_DSM\_10331
Acidimicrobium\_ferrooxidans\_DSM\_10331
Acidimicrobium\_ferrooxidans\_DSM\_10331
no rank

17.2517.2517.2517.2517.2517.2517.2517.2517.2517.2517.2517.2517.2517.2517.2517.2517.2517.2517.2517.25
77777777777777777777
2.116531.809781.650091.589550.3062390.3062390.3062390.3062390.5024740.3062390.3062390.30623900000000
32220000100000000000
84998
subclass

17.2517.2517.2517.2517.2517.2517.2517.2517.2517.2517.2517.2517.2517.2517.2517.2517.2517.2517.2517.25
77777777777777777777
2.116531.809781.650091.589550.3062390.3062390.3062390.3062390.5024740.3062390.3062390.30623900000000
32220000100000000000
84999
order

17.2517.2517.2517.2517.2517.2517.2517.2517.2517.2517.2517.2517.2517.2517.2517.2517.2517.2517.2517.25
77777777777777777777
2.116531.809781.650091.589550.3062390.3062390.3062390.3062390.5024740.3062390.3062390.30623900000000
32220000100000000000
255727
suborder

17.2517.2517.2517.2517.2517.2517.2517.2517.2517.2517.2517.2517.2517.2517.2517.2517.2517.2517.2517.25
77777777777777777777
2.116531.809781.650091.589550.3062390.3062390.3062390.3062390.5024740.3062390.3062390.30623900000000
32220000100000000000
84107
family

3.173.173.173.173.173.173.173.173.173.173.173.173.173.173.173.173.173.173.173.17
11111111111111111111
2.609442.098611.693151.693150000000000000000
53220000000000000000
84108
genus

3.173.173.173.173.173.173.173.173.173.173.173.173.173.173.173.173.173.173.173.17
11111111111111111111
2.609442.098611.693151.693150000000000000000
53220000000000000000
84110
species

3.173.173.173.173.173.173.173.173.173.173.173.173.173.173.173.173.173.173.173.17
11111111111111111111
2.609442.098611.693151.693150000000000000000
53220000000000000000
471855

Slackia\_heliotrinireducens\_DSM\_20476
Slackia\_heliotrinireducens\_DSM\_20476
Slackia\_heliotrinireducens\_DSM\_20476
Slackia\_heliotrinireducens\_DSM\_20476
Slackia\_heliotrinireducens\_DSM\_20476
Slackia\_heliotrinireducens\_DSM\_20476
Slackia\_heliotrinireducens\_DSM\_20476
Slackia\_heliotrinireducens\_DSM\_20476
Slackia\_heliotrinireducens\_DSM\_20476
Slackia\_heliotrinireducens\_DSM\_20476
Slackia\_heliotrinireducens\_DSM\_20476
Slackia\_heliotrinireducens\_DSM\_20476
Slackia\_heliotrinireducens\_DSM\_20476
Slackia\_heliotrinireducens\_DSM\_20476
Slackia\_heliotrinireducens\_DSM\_20476
Slackia\_heliotrinireducens\_DSM\_20476
Slackia\_heliotrinireducens\_DSM\_20476
Slackia\_heliotrinireducens\_DSM\_20476
Slackia\_heliotrinireducens\_DSM\_20476
Slackia\_heliotrinireducens\_DSM\_20476
no rank

1.541.541.541.541.541.541.541.541.541.541.541.541.541.541.541.541.541.541.541.54
11111111111111111111
00000000000000000000
00000000000000000000
1380
genus

1.541.541.541.541.541.541.541.541.541.541.541.541.541.541.541.541.541.541.541.54
11111111111111111111
00000000000000000000
00000000000000000000
1382
species

1.541.541.541.541.541.541.541.541.541.541.541.541.541.541.541.541.541.541.541.54
11111111111111111111
00000000000000000000
00000000000000000000
521095

Atopobium\_parvulum\_DSM\_20469
Atopobium\_parvulum\_DSM\_20469
Atopobium\_parvulum\_DSM\_20469
Atopobium\_parvulum\_DSM\_20469
Atopobium\_parvulum\_DSM\_20469
Atopobium\_parvulum\_DSM\_20469
Atopobium\_parvulum\_DSM\_20469
Atopobium\_parvulum\_DSM\_20469
Atopobium\_parvulum\_DSM\_20469
Atopobium\_parvulum\_DSM\_20469
Atopobium\_parvulum\_DSM\_20469
Atopobium\_parvulum\_DSM\_20469
Atopobium\_parvulum\_DSM\_20469
Atopobium\_parvulum\_DSM\_20469
Atopobium\_parvulum\_DSM\_20469
Atopobium\_parvulum\_DSM\_20469
Atopobium\_parvulum\_DSM\_20469
Atopobium\_parvulum\_DSM\_20469
Atopobium\_parvulum\_DSM\_20469
Atopobium\_parvulum\_DSM\_20469
no rank

1.621.621.621.621.621.621.621.621.621.621.621.621.621.621.621.621.621.621.621.62
11111111111111111111
2.098612.098612.098612.098610000000000000000
33330000000000000000
84162
genus

1.621.621.621.621.621.621.621.621.621.621.621.621.621.621.621.621.621.621.621.62
11111111111111111111
2.098612.098612.098612.098610000000000000000
33330000000000000000
84163
species

1.621.621.621.621.621.621.621.621.621.621.621.621.621.621.621.621.621.621.621.62
11111111111111111111
2.098612.098612.098612.098610000000000000000
33330000000000000000
469378

Cryptobacterium\_curtum\_DSM\_15641
Cryptobacterium\_curtum\_DSM\_15641
Cryptobacterium\_curtum\_DSM\_15641
Cryptobacterium\_curtum\_DSM\_15641
Cryptobacterium\_curtum\_DSM\_15641
Cryptobacterium\_curtum\_DSM\_15641
Cryptobacterium\_curtum\_DSM\_15641
Cryptobacterium\_curtum\_DSM\_15641
Cryptobacterium\_curtum\_DSM\_15641
Cryptobacterium\_curtum\_DSM\_15641
Cryptobacterium\_curtum\_DSM\_15641
Cryptobacterium\_curtum\_DSM\_15641
Cryptobacterium\_curtum\_DSM\_15641
Cryptobacterium\_curtum\_DSM\_15641
Cryptobacterium\_curtum\_DSM\_15641
Cryptobacterium\_curtum\_DSM\_15641
Cryptobacterium\_curtum\_DSM\_15641
Cryptobacterium\_curtum\_DSM\_15641
Cryptobacterium\_curtum\_DSM\_15641
Cryptobacterium\_curtum\_DSM\_15641
no rank

6.756.756.756.756.756.756.756.756.756.756.756.756.756.756.756.756.756.756.756.75
22222222222222222222
2.930312.386292.386292.231590.782610.782610.782610.782610.9700250.782610.782610.7826100000000
74431111111100000000
84111
genus

3.633.633.633.633.633.633.633.633.633.633.633.633.633.633.633.633.633.633.633.63
11111111111111111111
3.39792.386292.386292.098610000000000000000
114430000000000000000
84112
species

3.633.633.633.633.633.633.633.633.633.633.633.633.633.633.633.633.633.633.633.63
11111111111111111111
3.39792.386292.386292.098610000000000000000
114430000000000000000
479437

Eggerthella\_lenta\_DSM\_2243
Eggerthella\_lenta\_DSM\_2243
Eggerthella\_lenta\_DSM\_2243
Eggerthella\_lenta\_DSM\_2243
Eggerthella\_lenta\_DSM\_2243
Eggerthella\_lenta\_DSM\_2243
Eggerthella\_lenta\_DSM\_2243
Eggerthella\_lenta\_DSM\_2243
Eggerthella\_lenta\_DSM\_2243
Eggerthella\_lenta\_DSM\_2243
Eggerthella\_lenta\_DSM\_2243
Eggerthella\_lenta\_DSM\_2243
Eggerthella\_lenta\_DSM\_2243
Eggerthella\_lenta\_DSM\_2243
Eggerthella\_lenta\_DSM\_2243
Eggerthella\_lenta\_DSM\_2243
Eggerthella\_lenta\_DSM\_2243
Eggerthella\_lenta\_DSM\_2243
Eggerthella\_lenta\_DSM\_2243
Eggerthella\_lenta\_DSM\_2243
no rank

3.123.123.123.123.123.123.123.123.123.123.123.123.123.123.123.123.123.123.123.12
11111111111111111111
2.386292.386292.386292.386291.693151.693151.693151.693152.098611.693151.693151.6931500000000
44442222322200000000
502558

Eggerthella\_sp.\_YY7918
Eggerthella\_sp.\_YY7918
Eggerthella\_sp.\_YY7918
Eggerthella\_sp.\_YY7918
Eggerthella\_sp.\_YY7918
Eggerthella\_sp.\_YY7918
Eggerthella\_sp.\_YY7918
Eggerthella\_sp.\_YY7918
Eggerthella\_sp.\_YY7918
Eggerthella\_sp.\_YY7918
Eggerthella\_sp.\_YY7918
Eggerthella\_sp.\_YY7918
Eggerthella\_sp.\_YY7918
Eggerthella\_sp.\_YY7918
Eggerthella\_sp.\_YY7918
Eggerthella\_sp.\_YY7918
Eggerthella\_sp.\_YY7918
Eggerthella\_sp.\_YY7918
Eggerthella\_sp.\_YY7918
Eggerthella\_sp.\_YY7918
species

2.122.122.122.122.122.122.122.122.122.122.122.122.122.122.122.122.122.122.122.12
11111111111111111111
2.386292.386291.693151.693150000100000000000
44220000100000000000
33870
genus

2.122.122.122.122.122.122.122.122.122.122.122.122.122.122.122.122.122.122.122.12
11111111111111111111
2.386292.386291.693151.693150000100000000000
44220000100000000000
33871
species

2.122.122.122.122.122.122.122.122.122.122.122.122.122.122.122.122.122.122.122.12
11111111111111111111
2.386292.386291.693151.693150000100000000000
44220000100000000000
700015

Coriobacterium\_glomerans\_PW2
Coriobacterium\_glomerans\_PW2
Coriobacterium\_glomerans\_PW2
Coriobacterium\_glomerans\_PW2
Coriobacterium\_glomerans\_PW2
Coriobacterium\_glomerans\_PW2
Coriobacterium\_glomerans\_PW2
Coriobacterium\_glomerans\_PW2
Coriobacterium\_glomerans\_PW2
Coriobacterium\_glomerans\_PW2
Coriobacterium\_glomerans\_PW2
Coriobacterium\_glomerans\_PW2
Coriobacterium\_glomerans\_PW2
Coriobacterium\_glomerans\_PW2
Coriobacterium\_glomerans\_PW2
Coriobacterium\_glomerans\_PW2
Coriobacterium\_glomerans\_PW2
Coriobacterium\_glomerans\_PW2
Coriobacterium\_glomerans\_PW2
Coriobacterium\_glomerans\_PW2
no rank

2.052.052.052.052.052.052.052.052.052.052.052.052.052.052.052.052.052.052.052.05
11111111111111111111
00000000000000000000
00000000000000000000
133925
genus

2.052.052.052.052.052.052.052.052.052.052.052.052.052.052.052.052.052.052.052.05
11111111111111111111
00000000000000000000
00000000000000000000
133926
species

2.052.052.052.052.052.052.052.052.052.052.052.052.052.052.052.052.052.052.052.05
11111111111111111111
00000000000000000000
00000000000000000000
633147

Olsenella\_uli\_DSM\_7084
Olsenella\_uli\_DSM\_7084
Olsenella\_uli\_DSM\_7084
Olsenella\_uli\_DSM\_7084
Olsenella\_uli\_DSM\_7084
Olsenella\_uli\_DSM\_7084
Olsenella\_uli\_DSM\_7084
Olsenella\_uli\_DSM\_7084
Olsenella\_uli\_DSM\_7084
Olsenella\_uli\_DSM\_7084
Olsenella\_uli\_DSM\_7084
Olsenella\_uli\_DSM\_7084
Olsenella\_uli\_DSM\_7084
Olsenella\_uli\_DSM\_7084
Olsenella\_uli\_DSM\_7084
Olsenella\_uli\_DSM\_7084
Olsenella\_uli\_DSM\_7084
Olsenella\_uli\_DSM\_7084
Olsenella\_uli\_DSM\_7084
Olsenella\_uli\_DSM\_7084
no rank

9.599.599.599.599.599.599.599.599.599.599.599.599.599.599.599.599.599.599.599.59
22222222222222222222
1.144440.992210.992210.940290000000000000000
11110000000000000000
84995
subclass

3.233.233.233.233.233.233.233.233.233.233.233.233.233.233.233.233.233.233.233.23
11111111111111111111
3.39792.945912.945912.791760000000000000000
117760000000000000000
84996
order

3.233.233.233.233.233.233.233.233.233.233.233.233.233.233.233.233.233.233.233.23
11111111111111111111
3.39792.945912.945912.791760000000000000000
117760000000000000000
255725
suborder

3.233.233.233.233.233.233.233.233.233.233.233.233.233.233.233.233.233.233.233.23
11111111111111111111
3.39792.945912.945912.791760000000000000000
117760000000000000000
84997
family

3.233.233.233.233.233.233.233.233.233.233.233.233.233.233.233.233.233.233.233.23
11111111111111111111
3.39792.945912.945912.791760000000000000000
117760000000000000000
42255
genus

3.233.233.233.233.233.233.233.233.233.233.233.233.233.233.233.233.233.233.233.23
11111111111111111111
3.39792.945912.945912.791760000000000000000
117760000000000000000
49319
species

3.233.233.233.233.233.233.233.233.233.233.233.233.233.233.233.233.233.233.233.23
11111111111111111111
3.39792.945912.945912.791760000000000000000
117760000000000000000
266117

Rubrobacter\_xylanophilus\_DSM\_9941
Rubrobacter\_xylanophilus\_DSM\_9941
Rubrobacter\_xylanophilus\_DSM\_9941
Rubrobacter\_xylanophilus\_DSM\_9941
Rubrobacter\_xylanophilus\_DSM\_9941
Rubrobacter\_xylanophilus\_DSM\_9941
Rubrobacter\_xylanophilus\_DSM\_9941
Rubrobacter\_xylanophilus\_DSM\_9941
Rubrobacter\_xylanophilus\_DSM\_9941
Rubrobacter\_xylanophilus\_DSM\_9941
Rubrobacter\_xylanophilus\_DSM\_9941
Rubrobacter\_xylanophilus\_DSM\_9941
Rubrobacter\_xylanophilus\_DSM\_9941
Rubrobacter\_xylanophilus\_DSM\_9941
Rubrobacter\_xylanophilus\_DSM\_9941
Rubrobacter\_xylanophilus\_DSM\_9941
Rubrobacter\_xylanophilus\_DSM\_9941
Rubrobacter\_xylanophilus\_DSM\_9941
Rubrobacter\_xylanophilus\_DSM\_9941
Rubrobacter\_xylanophilus\_DSM\_9941
no rank

6.366.366.366.366.366.366.366.366.366.366.366.366.366.366.366.366.366.366.366.36
11111111111111111111
00000000000000000000
00000000000000000000
588673
order

6.366.366.366.366.366.366.366.366.366.366.366.366.366.366.366.366.366.366.366.36
11111111111111111111
00000000000000000000
00000000000000000000
320583
family

6.366.366.366.366.366.366.366.366.366.366.366.366.366.366.366.366.366.366.366.36
11111111111111111111
00000000000000000000
00000000000000000000
191494
genus

6.366.366.366.366.366.366.366.366.366.366.366.366.366.366.366.366.366.366.366.36
11111111111111111111
00000000000000000000
00000000000000000000
191495
species

6.366.366.366.366.366.366.366.366.366.366.366.366.366.366.366.366.366.366.366.36
11111111111111111111
00000000000000000000
00000000000000000000
469383

Conexibacter\_woesei\_DSM\_14684
Conexibacter\_woesei\_DSM\_14684
Conexibacter\_woesei\_DSM\_14684
Conexibacter\_woesei\_DSM\_14684
Conexibacter\_woesei\_DSM\_14684
Conexibacter\_woesei\_DSM\_14684
Conexibacter\_woesei\_DSM\_14684
Conexibacter\_woesei\_DSM\_14684
Conexibacter\_woesei\_DSM\_14684
Conexibacter\_woesei\_DSM\_14684
Conexibacter\_woesei\_DSM\_14684
Conexibacter\_woesei\_DSM\_14684
Conexibacter\_woesei\_DSM\_14684
Conexibacter\_woesei\_DSM\_14684
Conexibacter\_woesei\_DSM\_14684
Conexibacter\_woesei\_DSM\_14684
Conexibacter\_woesei\_DSM\_14684
Conexibacter\_woesei\_DSM\_14684
Conexibacter\_woesei\_DSM\_14684
Conexibacter\_woesei\_DSM\_14684
no rank

1005.181005.181005.181005.181005.181005.181005.181005.181005.181005.181005.181005.181005.181005.181005.181005.181005.181005.181005.181005.18
224224224224224224224224224224224224224224224224224224224224
3.484113.2213.10053.005010.9887470.6651930.5703410.5790710.7680620.5820570.5164330.527420.3947620.356820.3390130.3257520.2648070.2185540.221630.228418
129871111111111110000
85003
subclass

942.67942.67942.67942.67942.67942.67942.67942.67942.67942.67942.67942.67942.67942.67942.67942.67942.67942.67942.67942.67
195195195195195195195195195195195195195195195195195195195195
3.513453.244063.118563.017591.011570.7007580.6081610.6154230.8015530.6176520.5478770.5623940.420940.3804810.3614940.3473530.2823670.2330470.2363260.243565
129881111111111110000
2037
order

118.60118.60118.60118.60118.60118.60118.60118.60118.60118.60118.60118.60118.60118.60118.60118.60118.60118.60118.60118.60
1313131313131313131313131313131313131313
3.945593.807293.718523.639821.675341.220211.167091.167091.356141.1291.06391.1290.9699670.9699670.9699670.8250640.7115070.6239860.6239860.623986
191715142111111111111111
85011
suborder

118.60118.60118.60118.60118.60118.60118.60118.60118.60118.60118.60118.60118.60118.60118.60118.60118.60118.60118.60118.60
1313131313131313131313131313131313131313
3.945593.807293.718523.639821.675341.220211.167091.167091.356141.1291.06391.1290.9699670.9699670.9699670.8250640.7115070.6239860.6239860.623986
191715142111111111111111
2062
family

8.788.788.788.788.788.788.788.788.788.788.788.788.788.788.788.788.788.788.788.78
11111111111111111111
3.995733.995733.890373.944442.386291.693151.693151.693152.098611.693151.693151.693151.693151.693151.693151.693151.693151.693151.693151.69315
202018194222322222222222
2063
genus

8.788.788.788.788.788.788.788.788.788.788.788.788.788.788.788.788.788.788.788.78
11111111111111111111
3.995733.995733.890373.944442.386291.693151.693151.693152.098611.693151.693151.693151.693151.693151.693151.693151.693151.693151.693151.69315
202018194222322222222222
2066
species

8.788.788.788.788.788.788.788.788.788.788.788.788.788.788.788.788.788.788.788.78
11111111111111111111
3.995733.995733.890373.944442.386291.693151.693151.693152.098611.693151.693151.693151.693151.693151.693151.693151.693151.693151.693151.69315
202018194222322222222222
452652

Kitasatospora\_setae\_KM-6054
Kitasatospora\_setae\_KM-6054
Kitasatospora\_setae\_KM-6054
Kitasatospora\_setae\_KM-6054
Kitasatospora\_setae\_KM-6054
Kitasatospora\_setae\_KM-6054
Kitasatospora\_setae\_KM-6054
Kitasatospora\_setae\_KM-6054
Kitasatospora\_setae\_KM-6054
Kitasatospora\_setae\_KM-6054
Kitasatospora\_setae\_KM-6054
Kitasatospora\_setae\_KM-6054
Kitasatospora\_setae\_KM-6054
Kitasatospora\_setae\_KM-6054
Kitasatospora\_setae\_KM-6054
Kitasatospora\_setae\_KM-6054
Kitasatospora\_setae\_KM-6054
Kitasatospora\_setae\_KM-6054
Kitasatospora\_setae\_KM-6054
Kitasatospora\_setae\_KM-6054
no rank

109.82109.82109.82109.82109.82109.82109.82109.82109.82109.82109.82109.82109.82109.82109.82109.82109.82109.82109.82109.82
1212121212121212121212121212121212121212
3.941583.792223.704783.615471.618491.18241.125031.125031.296781.08391.013591.08390.9121490.9121490.9121490.7556620.6330260.5385080.5385080.538508
191615142111111111111111
1883
genus

7.417.417.417.417.417.417.417.417.417.417.417.417.417.417.417.417.417.417.417.41
11111111111111111111
3.197222.945912.791762.791761.69315000000000000000
97662000000000000000
862751

Streptomyces\_sp.\_SirexAA-E
Streptomyces\_sp.\_SirexAA-E
Streptomyces\_sp.\_SirexAA-E
Streptomyces\_sp.\_SirexAA-E
Streptomyces\_sp.\_SirexAA-E
Streptomyces\_sp.\_SirexAA-E
Streptomyces\_sp.\_SirexAA-E
Streptomyces\_sp.\_SirexAA-E
Streptomyces\_sp.\_SirexAA-E
Streptomyces\_sp.\_SirexAA-E
Streptomyces\_sp.\_SirexAA-E
Streptomyces\_sp.\_SirexAA-E
Streptomyces\_sp.\_SirexAA-E
Streptomyces\_sp.\_SirexAA-E
Streptomyces\_sp.\_SirexAA-E
Streptomyces\_sp.\_SirexAA-E
Streptomyces\_sp.\_SirexAA-E
Streptomyces\_sp.\_SirexAA-E
Streptomyces\_sp.\_SirexAA-E
Streptomyces\_sp.\_SirexAA-E
species

16.1916.1916.1916.1916.1916.1916.1916.1916.1916.1916.1916.1916.1916.1916.1916.1916.1916.1916.1916.19
22222222222222222222
3.917423.917423.74033.524950000000000000000
181815120000000000000000
29303
species

16.1916.1916.1916.1916.1916.1916.1916.1916.1916.1916.1916.1916.1916.1916.1916.1916.1916.1916.1916.19
22222222222222222222
3.917423.917423.74033.524950000000000000000
181815120000000000000000
1003195

Streptomyces\_cattleya\_NRRL\_8057\_=\_DSM\_46488Streptomyces\_cattleya\_NRRL\_8057\_=\_DSM\_46488
Streptomyces\_cattleya\_NRRL\_8057\_=\_DSM\_46488Streptomyces\_cattleya\_NRRL\_8057\_=\_DSM\_46488
Streptomyces\_cattleya\_NRRL\_8057\_=\_DSM\_46488Streptomyces\_cattleya\_NRRL\_8057\_=\_DSM\_46488
Streptomyces\_cattleya\_NRRL\_8057\_=\_DSM\_46488Streptomyces\_cattleya\_NRRL\_8057\_=\_DSM\_46488
Streptomyces\_cattleya\_NRRL\_8057\_=\_DSM\_46488Streptomyces\_cattleya\_NRRL\_8057\_=\_DSM\_46488
Streptomyces\_cattleya\_NRRL\_8057\_=\_DSM\_46488Streptomyces\_cattleya\_NRRL\_8057\_=\_DSM\_46488
Streptomyces\_cattleya\_NRRL\_8057\_=\_DSM\_46488Streptomyces\_cattleya\_NRRL\_8057\_=\_DSM\_46488
Streptomyces\_cattleya\_NRRL\_8057\_=\_DSM\_46488Streptomyces\_cattleya\_NRRL\_8057\_=\_DSM\_46488
Streptomyces\_cattleya\_NRRL\_8057\_=\_DSM\_46488Streptomyces\_cattleya\_NRRL\_8057\_=\_DSM\_46488
Streptomyces\_cattleya\_NRRL\_8057\_=\_DSM\_46488Streptomyces\_cattleya\_NRRL\_8057\_=\_DSM\_46488
Streptomyces\_cattleya\_NRRL\_8057\_=\_DSM\_46488Streptomyces\_cattleya\_NRRL\_8057\_=\_DSM\_46488
Streptomyces\_cattleya\_NRRL\_8057\_=\_DSM\_46488Streptomyces\_cattleya\_NRRL\_8057\_=\_DSM\_46488
Streptomyces\_cattleya\_NRRL\_8057\_=\_DSM\_46488Streptomyces\_cattleya\_NRRL\_8057\_=\_DSM\_46488
Streptomyces\_cattleya\_NRRL\_8057\_=\_DSM\_46488Streptomyces\_cattleya\_NRRL\_8057\_=\_DSM\_46488
Streptomyces\_cattleya\_NRRL\_8057\_=\_DSM\_46488Streptomyces\_cattleya\_NRRL\_8057\_=\_DSM\_46488
Streptomyces\_cattleya\_NRRL\_8057\_=\_DSM\_46488Streptomyces\_cattleya\_NRRL\_8057\_=\_DSM\_46488
Streptomyces\_cattleya\_NRRL\_8057\_=\_DSM\_46488Streptomyces\_cattleya\_NRRL\_8057\_=\_DSM\_46488
Streptomyces\_cattleya\_NRRL\_8057\_=\_DSM\_46488Streptomyces\_cattleya\_NRRL\_8057\_=\_DSM\_46488
Streptomyces\_cattleya\_NRRL\_8057\_=\_DSM\_46488Streptomyces\_cattleya\_NRRL\_8057\_=\_DSM\_46488
Streptomyces\_cattleya\_NRRL\_8057\_=\_DSM\_46488Streptomyces\_cattleya\_NRRL\_8057\_=\_DSM\_46488
no rank

10.3810.3810.3810.3810.3810.3810.3810.3810.3810.3810.3810.3810.3810.3810.3810.3810.3810.3810.3810.38
11111111111111111111
4.465744.295844.135494.135492.386291.693151.693151.693151.693151.693151.693151.693151.693151.693151.693151.693151000
322723234222222222221000
1912
species

10.3810.3810.3810.3810.3810.3810.3810.3810.3810.3810.3810.3810.3810.3810.3810.3810.3810.3810.3810.38
11111111111111111111
4.465744.295844.135494.135492.386291.693151.693151.693151.693151.693151.693151.693151.693151.693151.693151.693151000
322723234222222222221000
311982
subspecies

10.3810.3810.3810.3810.3810.3810.3810.3810.3810.3810.3810.3810.3810.3810.3810.3810.3810.3810.3810.38
11111111111111111111
4.465744.295844.135494.135492.386291.693151.693151.693151.693151.693151.693151.693151.693151.693151.693151.693151000
322723234222222222221000
1133850

Streptomyces\_hygroscopicus\_subsp.\_jinggangensis\_5008
Streptomyces\_hygroscopicus\_subsp.\_jinggangensis\_5008
Streptomyces\_hygroscopicus\_subsp.\_jinggangensis\_5008
Streptomyces\_hygroscopicus\_subsp.\_jinggangensis\_5008
Streptomyces\_hygroscopicus\_subsp.\_jinggangensis\_5008
Streptomyces\_hygroscopicus\_subsp.\_jinggangensis\_5008
Streptomyces\_hygroscopicus\_subsp.\_jinggangensis\_5008
Streptomyces\_hygroscopicus\_subsp.\_jinggangensis\_5008
Streptomyces\_hygroscopicus\_subsp.\_jinggangensis\_5008
Streptomyces\_hygroscopicus\_subsp.\_jinggangensis\_5008
Streptomyces\_hygroscopicus\_subsp.\_jinggangensis\_5008
Streptomyces\_hygroscopicus\_subsp.\_jinggangensis\_5008
Streptomyces\_hygroscopicus\_subsp.\_jinggangensis\_5008
Streptomyces\_hygroscopicus\_subsp.\_jinggangensis\_5008
Streptomyces\_hygroscopicus\_subsp.\_jinggangensis\_5008
Streptomyces\_hygroscopicus\_subsp.\_jinggangensis\_5008
Streptomyces\_hygroscopicus\_subsp.\_jinggangensis\_5008
Streptomyces\_hygroscopicus\_subsp.\_jinggangensis\_5008
Streptomyces\_hygroscopicus\_subsp.\_jinggangensis\_5008
Streptomyces\_hygroscopicus\_subsp.\_jinggangensis\_5008
no rank

11.9411.9411.9411.9411.9411.9411.9411.9411.9411.9411.9411.9411.9411.9411.9411.9411.9411.9411.9411.94
11111111111111111111
3.302593.197223.197222.945910000000000000000
109970000000000000000
379067
species

11.9411.9411.9411.9411.9411.9411.9411.9411.9411.9411.9411.9411.9411.9411.9411.9411.9411.9411.9411.94
11111111111111111111
3.302593.197223.197222.945910000000000000000
109970000000000000000
749414

Streptomyces\_bingchenggensis\_BCW-1
Streptomyces\_bingchenggensis\_BCW-1
Streptomyces\_bingchenggensis\_BCW-1
Streptomyces\_bingchenggensis\_BCW-1
Streptomyces\_bingchenggensis\_BCW-1
Streptomyces\_bingchenggensis\_BCW-1
Streptomyces\_bingchenggensis\_BCW-1
Streptomyces\_bingchenggensis\_BCW-1
Streptomyces\_bingchenggensis\_BCW-1
Streptomyces\_bingchenggensis\_BCW-1
Streptomyces\_bingchenggensis\_BCW-1
Streptomyces\_bingchenggensis\_BCW-1
Streptomyces\_bingchenggensis\_BCW-1
Streptomyces\_bingchenggensis\_BCW-1
Streptomyces\_bingchenggensis\_BCW-1
Streptomyces\_bingchenggensis\_BCW-1
Streptomyces\_bingchenggensis\_BCW-1
Streptomyces\_bingchenggensis\_BCW-1
Streptomyces\_bingchenggensis\_BCW-1
Streptomyces\_bingchenggensis\_BCW-1
no rank

9.059.059.059.059.059.059.059.059.059.059.059.059.059.059.059.059.059.059.059.05
11111111111111111111
4.178054.091043.995733.890372.609442.386292.386292.386292.609442.386292.386292.386292.386292.386292.386292.386291.693151.693151.693151.69315
242220185444544444442222
1902
species

9.059.059.059.059.059.059.059.059.059.059.059.059.059.059.059.059.059.059.059.05
11111111111111111111
4.178054.091043.995733.890372.609442.386292.386292.386292.609442.386292.386292.386292.386292.386292.386292.386291.693151.693151.693151.69315
242220185444544444442222
100226

Streptomyces\_coelicolor\_A3(2)
Streptomyces\_coelicolor\_A3(2)
Streptomyces\_coelicolor\_A3(2)
Streptomyces\_coelicolor\_A3(2)
Streptomyces\_coelicolor\_A3(2)
Streptomyces\_coelicolor\_A3(2)
Streptomyces\_coelicolor\_A3(2)
Streptomyces\_coelicolor\_A3(2)
Streptomyces\_coelicolor\_A3(2)
Streptomyces\_coelicolor\_A3(2)
Streptomyces\_coelicolor\_A3(2)
Streptomyces\_coelicolor\_A3(2)
Streptomyces\_coelicolor\_A3(2)
Streptomyces\_coelicolor\_A3(2)
Streptomyces\_coelicolor\_A3(2)
Streptomyces\_coelicolor\_A3(2)
Streptomyces\_coelicolor\_A3(2)
Streptomyces\_coelicolor\_A3(2)
Streptomyces\_coelicolor\_A3(2)
Streptomyces\_coelicolor\_A3(2)
no rank

7.667.667.667.667.667.667.667.667.667.667.667.667.667.667.667.667.667.667.667.66
11111111111111111111
3.639063.39793.39793.484911000000000000000
141111121000000000000000
67299
species

7.667.667.667.667.667.667.667.667.667.667.667.667.667.667.667.667.667.667.667.66
11111111111111111111
3.639063.39793.39793.484911000000000000000
141111121000000000000000
591167

Streptomyces\_flavogriseus\_ATCC\_33331
Streptomyces\_flavogriseus\_ATCC\_33331
Streptomyces\_flavogriseus\_ATCC\_33331
Streptomyces\_flavogriseus\_ATCC\_33331
Streptomyces\_flavogriseus\_ATCC\_33331
Streptomyces\_flavogriseus\_ATCC\_33331
Streptomyces\_flavogriseus\_ATCC\_33331
Streptomyces\_flavogriseus\_ATCC\_33331
Streptomyces\_flavogriseus\_ATCC\_33331
Streptomyces\_flavogriseus\_ATCC\_33331
Streptomyces\_flavogriseus\_ATCC\_33331
Streptomyces\_flavogriseus\_ATCC\_33331
Streptomyces\_flavogriseus\_ATCC\_33331
Streptomyces\_flavogriseus\_ATCC\_33331
Streptomyces\_flavogriseus\_ATCC\_33331
Streptomyces\_flavogriseus\_ATCC\_33331
Streptomyces\_flavogriseus\_ATCC\_33331
Streptomyces\_flavogriseus\_ATCC\_33331
Streptomyces\_flavogriseus\_ATCC\_33331
Streptomyces\_flavogriseus\_ATCC\_33331
no rank

11.1411.1411.1411.1411.1411.1411.1411.1411.1411.1411.1411.1411.1411.1411.1411.1411.1411.1411.1411.14
11111111111111111111
4.555354.178054.218884.135493.079442.609442.098612.098612.791761.6931511.6931500000000
352425238533621200000000
68280
species

11.1411.1411.1411.1411.1411.1411.1411.1411.1411.1411.1411.1411.1411.1411.1411.1411.1411.1411.1411.14
11111111111111111111
4.555354.178054.218884.135493.079442.609442.098612.098612.791761.6931511.6931500000000
352425238533621200000000
653045

Streptomyces\_violaceusniger\_Tu\_4113
Streptomyces\_violaceusniger\_Tu\_4113
Streptomyces\_violaceusniger\_Tu\_4113
Streptomyces\_violaceusniger\_Tu\_4113
Streptomyces\_violaceusniger\_Tu\_4113
Streptomyces\_violaceusniger\_Tu\_4113
Streptomyces\_violaceusniger\_Tu\_4113
Streptomyces\_violaceusniger\_Tu\_4113
Streptomyces\_violaceusniger\_Tu\_4113
Streptomyces\_violaceusniger\_Tu\_4113
Streptomyces\_violaceusniger\_Tu\_4113
Streptomyces\_violaceusniger\_Tu\_4113
Streptomyces\_violaceusniger\_Tu\_4113
Streptomyces\_violaceusniger\_Tu\_4113
Streptomyces\_violaceusniger\_Tu\_4113
Streptomyces\_violaceusniger\_Tu\_4113
Streptomyces\_violaceusniger\_Tu\_4113
Streptomyces\_violaceusniger\_Tu\_4113
Streptomyces\_violaceusniger\_Tu\_4113
Streptomyces\_violaceusniger\_Tu\_4113
no rank

8.558.558.558.558.558.558.558.558.558.558.558.558.558.558.558.558.558.558.558.55
11111111111111111111
3.39793.39793.302593.39791.693151.693151.693151.693151.693151.693151.693151.693151.693151.693151.693151.693151.693151.693151.693151.69315
111110112222222222222222
629295
species subgroup

8.558.558.558.558.558.558.558.558.558.558.558.558.558.558.558.558.558.558.558.55
11111111111111111111
3.39793.39793.302593.39791.693151.693151.693151.693151.693151.693151.693151.693151.693151.693151.693151.693151.693151.693151.693151.69315
111110112222222222222222
1911
species

8.558.558.558.558.558.558.558.558.558.558.558.558.558.558.558.558.558.558.558.55
11111111111111111111
3.39793.39793.302593.39791.693151.693151.693151.693151.693151.693151.693151.693151.693151.693151.693151.693151.693151.693151.693151.69315
111110112222222222222222
67263
subspecies

8.558.558.558.558.558.558.558.558.558.558.558.558.558.558.558.558.558.558.558.55
11111111111111111111
3.39793.39793.302593.39791.693151.693151.693151.693151.693151.693151.693151.693151.693151.693151.693151.693151.693151.693151.693151.69315
111110112222222222222222
455632

Streptomyces\_griseus\_subsp.\_griseus\_NBRC\_13350\_(Streptomyces
Streptomyces\_griseus\_subsp.\_griseus\_NBRC\_13350\_(Streptomyces
Streptomyces\_griseus\_subsp.\_griseus\_NBRC\_13350\_(Streptomyces
Streptomyces\_griseus\_subsp.\_griseus\_NBRC\_13350\_(Streptomyces
Streptomyces\_griseus\_subsp.\_griseus\_NBRC\_13350\_(Streptomyces
Streptomyces\_griseus\_subsp.\_griseus\_NBRC\_13350\_(Streptomyces
Streptomyces\_griseus\_subsp.\_griseus\_NBRC\_13350\_(Streptomyces
Streptomyces\_griseus\_subsp.\_griseus\_NBRC\_13350\_(Streptomyces
Streptomyces\_griseus\_subsp.\_griseus\_NBRC\_13350\_(Streptomyces
Streptomyces\_griseus\_subsp.\_griseus\_NBRC\_13350\_(Streptomyces
Streptomyces\_griseus\_subsp.\_griseus\_NBRC\_13350\_(Streptomyces
Streptomyces\_griseus\_subsp.\_griseus\_NBRC\_13350\_(Streptomyces
Streptomyces\_griseus\_subsp.\_griseus\_NBRC\_13350\_(Streptomyces
Streptomyces\_griseus\_subsp.\_griseus\_NBRC\_13350\_(Streptomyces
Streptomyces\_griseus\_subsp.\_griseus\_NBRC\_13350\_(Streptomyces
Streptomyces\_griseus\_subsp.\_griseus\_NBRC\_13350\_(Streptomyces
Streptomyces\_griseus\_subsp.\_griseus\_NBRC\_13350\_(Streptomyces
Streptomyces\_griseus\_subsp.\_griseus\_NBRC\_13350\_(Streptomyces
Streptomyces\_griseus\_subsp.\_griseus\_NBRC\_13350\_(Streptomyces
Streptomyces\_griseus\_subsp.\_griseus\_NBRC\_13350\_(Streptomyces
no rank

8.238.238.238.238.238.238.238.238.238.238.238.238.238.238.238.238.238.238.238.23
11111111111111111111
4.044523.995733.995733.995733.639063.639063.564953.564953.564953.564953.564953.564953.564953.564953.564953.564953.564953.564953.564953.56495
2120202014141313131313131313131313131313
54571
species

8.238.238.238.238.238.238.238.238.238.238.238.238.238.238.238.238.238.238.238.23
11111111111111111111
4.044523.995733.995733.995733.639063.639063.564953.564953.564953.564953.564953.564953.564953.564953.564953.564953.564953.564953.564953.56495
2120202014141313131313131313131313131313
953739

Streptomyces\_venezuelae\_ATCC\_10712
Streptomyces\_venezuelae\_ATCC\_10712
Streptomyces\_venezuelae\_ATCC\_10712
Streptomyces\_venezuelae\_ATCC\_10712
Streptomyces\_venezuelae\_ATCC\_10712
Streptomyces\_venezuelae\_ATCC\_10712
Streptomyces\_venezuelae\_ATCC\_10712
Streptomyces\_venezuelae\_ATCC\_10712
Streptomyces\_venezuelae\_ATCC\_10712
Streptomyces\_venezuelae\_ATCC\_10712
Streptomyces\_venezuelae\_ATCC\_10712
Streptomyces\_venezuelae\_ATCC\_10712
Streptomyces\_venezuelae\_ATCC\_10712
Streptomyces\_venezuelae\_ATCC\_10712
Streptomyces\_venezuelae\_ATCC\_10712
Streptomyces\_venezuelae\_ATCC\_10712
Streptomyces\_venezuelae\_ATCC\_10712
Streptomyces\_venezuelae\_ATCC\_10712
Streptomyces\_venezuelae\_ATCC\_10712
Streptomyces\_venezuelae\_ATCC\_10712
no rank

9.129.129.129.129.129.129.129.129.129.129.129.129.129.129.129.129.129.129.129.12
11111111111111111111
4.40124.465744.25814.25811000100000000000
303226261000100000000000
33903
species

9.129.129.129.129.129.129.129.129.129.129.129.129.129.129.129.129.129.129.129.12
11111111111111111111
4.40124.465744.25814.25811000100000000000
303226261000100000000000
227882

Streptomyces\_avermitilis\_MA-4680
Streptomyces\_avermitilis\_MA-4680
Streptomyces\_avermitilis\_MA-4680
Streptomyces\_avermitilis\_MA-4680
Streptomyces\_avermitilis\_MA-4680
Streptomyces\_avermitilis\_MA-4680
Streptomyces\_avermitilis\_MA-4680
Streptomyces\_avermitilis\_MA-4680
Streptomyces\_avermitilis\_MA-4680
Streptomyces\_avermitilis\_MA-4680
Streptomyces\_avermitilis\_MA-4680
Streptomyces\_avermitilis\_MA-4680
Streptomyces\_avermitilis\_MA-4680
Streptomyces\_avermitilis\_MA-4680
Streptomyces\_avermitilis\_MA-4680
Streptomyces\_avermitilis\_MA-4680
Streptomyces\_avermitilis\_MA-4680
Streptomyces\_avermitilis\_MA-4680
Streptomyces\_avermitilis\_MA-4680
Streptomyces\_avermitilis\_MA-4680
no rank

10.1510.1510.1510.1510.1510.1510.1510.1510.1510.1510.1510.1510.1510.1510.1510.1510.1510.1510.1510.15
11111111111111111111
4.044523.564953.484913.197222.098611.693151.693151.693151.693151.693151.693151.693151.693151.693151.6931500000
21131293222222222200000
1930
species

10.1510.1510.1510.1510.1510.1510.1510.1510.1510.1510.1510.1510.1510.1510.1510.1510.1510.1510.1510.15
11111111111111111111
4.044523.564953.484913.197222.098611.693151.693151.693151.693151.693151.693151.693151.693151.693151.6931500000
21131293222222222200000
680198

Streptomyces\_scabiei\_87.22
Streptomyces\_scabiei\_87.22
Streptomyces\_scabiei\_87.22
Streptomyces\_scabiei\_87.22
Streptomyces\_scabiei\_87.22
Streptomyces\_scabiei\_87.22
Streptomyces\_scabiei\_87.22
Streptomyces\_scabiei\_87.22
Streptomyces\_scabiei\_87.22
Streptomyces\_scabiei\_87.22
Streptomyces\_scabiei\_87.22
Streptomyces\_scabiei\_87.22
Streptomyces\_scabiei\_87.22
Streptomyces\_scabiei\_87.22
Streptomyces\_scabiei\_87.22
Streptomyces\_scabiei\_87.22
Streptomyces\_scabiei\_87.22
Streptomyces\_scabiei\_87.22
Streptomyces\_scabiei\_87.22
Streptomyces\_scabiei\_87.22
no rank

433.85433.85433.85433.85433.85433.85433.85433.85433.85433.85433.85433.85433.85433.85433.85433.85433.85433.85433.85433.85
103103103103103103103103103103103103103103103103103103103103
3.565693.216583.054632.946361.10380.8470080.787730.7440620.9164360.7975560.7349560.7235190.6115920.5236830.4824280.4913150.3858940.3224420.3295670.33361
139871111111111111111
85007
suborder

103.99103.99103.99103.99103.99103.99103.99103.99103.99103.99103.99103.99103.99103.99103.99103.99103.99103.99103.99103.99
4141414141414141414141414141414141414141
3.171822.989992.948262.897210.9200060.364550.2997590.1752180.5638740.2382790.1586870.1586870.1224510.1224510.08946690.08946690.06504150.06504150.04817770.0650415
97771100100000000000
1653
family

103.99103.99103.99103.99103.99103.99103.99103.99103.99103.99103.99103.99103.99103.99103.99103.99103.99103.99103.99103.99
4141414141414141414141414141414141414141
3.171822.989992.948262.897210.9200060.364550.2997590.1752180.5638740.2382790.1586870.1586870.1224510.1224510.08946690.08946690.06504150.06504150.04817770.0650415
97771100100000000000
1716
genus

2.822.822.822.822.822.822.822.822.822.822.822.822.822.822.822.822.822.822.822.82
11111111111111111111
3.995733.833213.772593.772591000000000000000
201716161000000000000000
169292
species

2.822.822.822.822.822.822.822.822.822.822.822.822.822.822.822.822.822.822.822.82
11111111111111111111
3.995733.833213.772593.772591000000000000000
201716161000000000000000
548476

Corynebacterium\_aurimucosum\_ATCC\_700975
Corynebacterium\_aurimucosum\_ATCC\_700975
Corynebacterium\_aurimucosum\_ATCC\_700975
Corynebacterium\_aurimucosum\_ATCC\_700975
Corynebacterium\_aurimucosum\_ATCC\_700975
Corynebacterium\_aurimucosum\_ATCC\_700975
Corynebacterium\_aurimucosum\_ATCC\_700975
Corynebacterium\_aurimucosum\_ATCC\_700975
Corynebacterium\_aurimucosum\_ATCC\_700975
Corynebacterium\_aurimucosum\_ATCC\_700975
Corynebacterium\_aurimucosum\_ATCC\_700975
Corynebacterium\_aurimucosum\_ATCC\_700975
Corynebacterium\_aurimucosum\_ATCC\_700975
Corynebacterium\_aurimucosum\_ATCC\_700975
Corynebacterium\_aurimucosum\_ATCC\_700975
Corynebacterium\_aurimucosum\_ATCC\_700975
Corynebacterium\_aurimucosum\_ATCC\_700975
Corynebacterium\_aurimucosum\_ATCC\_700975
Corynebacterium\_aurimucosum\_ATCC\_700975
Corynebacterium\_aurimucosum\_ATCC\_700975
no rank

32.1232.1232.1232.1232.1232.1232.1232.1232.1232.1232.1232.1232.1232.1232.1232.1232.1232.1232.1232.12
1313131313131313131313131313131313131313
3.586113.203253.135023.047330.9392770.7565130.5203510.3431710.8554980.3665520.2896530.2896530.2896530.2896530.2896530.2896530.2105750.2105750.1559780.210575
139881111110000000000
1717
species

2.492.492.492.492.492.492.492.492.492.492.492.492.492.492.492.492.492.492.492.49
11111111111111111111
3.708053.197223.197223.0794401.69315101.6931500000000000
159980210200000000000
698973

Corynebacterium\_diphtheriae\_BH8
Corynebacterium\_diphtheriae\_BH8
Corynebacterium\_diphtheriae\_BH8
Corynebacterium\_diphtheriae\_BH8
Corynebacterium\_diphtheriae\_BH8
Corynebacterium\_diphtheriae\_BH8
Corynebacterium\_diphtheriae\_BH8
Corynebacterium\_diphtheriae\_BH8
Corynebacterium\_diphtheriae\_BH8
Corynebacterium\_diphtheriae\_BH8
Corynebacterium\_diphtheriae\_BH8
Corynebacterium\_diphtheriae\_BH8
Corynebacterium\_diphtheriae\_BH8
Corynebacterium\_diphtheriae\_BH8
Corynebacterium\_diphtheriae\_BH8
Corynebacterium\_diphtheriae\_BH8
Corynebacterium\_diphtheriae\_BH8
Corynebacterium\_diphtheriae\_BH8
Corynebacterium\_diphtheriae\_BH8
Corynebacterium\_diphtheriae\_BH8
no rank

2.402.402.402.402.402.402.402.402.402.402.402.402.402.402.402.402.402.402.402.40
11111111111111111111
3.39793.302593.197223.197220100100000000000
1110990100100000000000
698971

Corynebacterium\_diphtheriae\_VA01
Corynebacterium\_diphtheriae\_VA01
Corynebacterium\_diphtheriae\_VA01
Corynebacterium\_diphtheriae\_VA01
Corynebacterium\_diphtheriae\_VA01
Corynebacterium\_diphtheriae\_VA01
Corynebacterium\_diphtheriae\_VA01
Corynebacterium\_diphtheriae\_VA01
Corynebacterium\_diphtheriae\_VA01
Corynebacterium\_diphtheriae\_VA01
Corynebacterium\_diphtheriae\_VA01
Corynebacterium\_diphtheriae\_VA01
Corynebacterium\_diphtheriae\_VA01
Corynebacterium\_diphtheriae\_VA01
Corynebacterium\_diphtheriae\_VA01
Corynebacterium\_diphtheriae\_VA01
Corynebacterium\_diphtheriae\_VA01
Corynebacterium\_diphtheriae\_VA01
Corynebacterium\_diphtheriae\_VA01
Corynebacterium\_diphtheriae\_VA01
no rank

2.432.432.432.432.432.432.432.432.432.432.432.432.432.432.432.432.432.432.432.43
11111111111111111111
3.079442.945912.791762.609441000100000000000
87651000100000000000
698965

Corynebacterium\_diphtheriae\_CDCE\_8392
Corynebacterium\_diphtheriae\_CDCE\_8392
Corynebacterium\_diphtheriae\_CDCE\_8392
Corynebacterium\_diphtheriae\_CDCE\_8392
Corynebacterium\_diphtheriae\_CDCE\_8392
Corynebacterium\_diphtheriae\_CDCE\_8392
Corynebacterium\_diphtheriae\_CDCE\_8392
Corynebacterium\_diphtheriae\_CDCE\_8392
Corynebacterium\_diphtheriae\_CDCE\_8392
Corynebacterium\_diphtheriae\_CDCE\_8392
Corynebacterium\_diphtheriae\_CDCE\_8392
Corynebacterium\_diphtheriae\_CDCE\_8392
Corynebacterium\_diphtheriae\_CDCE\_8392
Corynebacterium\_diphtheriae\_CDCE\_8392
Corynebacterium\_diphtheriae\_CDCE\_8392
Corynebacterium\_diphtheriae\_CDCE\_8392
Corynebacterium\_diphtheriae\_CDCE\_8392
Corynebacterium\_diphtheriae\_CDCE\_8392
Corynebacterium\_diphtheriae\_CDCE\_8392
Corynebacterium\_diphtheriae\_CDCE\_8392
no rank

2.532.532.532.532.532.532.532.532.532.532.532.532.532.532.532.532.532.532.532.53
11111111111111111111
3.944443.197223.197222.945912.098611.693151.693151.693151.693151.693151.693151.693151.693151.693151.693151.693151.693151.6931511.69315
199973222222222222212
698964

Corynebacterium\_diphtheriae\_PW8
Corynebacterium\_diphtheriae\_PW8
Corynebacterium\_diphtheriae\_PW8
Corynebacterium\_diphtheriae\_PW8
Corynebacterium\_diphtheriae\_PW8
Corynebacterium\_diphtheriae\_PW8
Corynebacterium\_diphtheriae\_PW8
Corynebacterium\_diphtheriae\_PW8
Corynebacterium\_diphtheriae\_PW8
Corynebacterium\_diphtheriae\_PW8
Corynebacterium\_diphtheriae\_PW8
Corynebacterium\_diphtheriae\_PW8
Corynebacterium\_diphtheriae\_PW8
Corynebacterium\_diphtheriae\_PW8
Corynebacterium\_diphtheriae\_PW8
Corynebacterium\_diphtheriae\_PW8
Corynebacterium\_diphtheriae\_PW8
Corynebacterium\_diphtheriae\_PW8
Corynebacterium\_diphtheriae\_PW8
Corynebacterium\_diphtheriae\_PW8
no rank

2.472.472.472.472.472.472.472.472.472.472.472.472.472.472.472.472.472.472.472.47
11111111111111111111
3.772593.484913.302593.39791.69315100110000000000
161210112100110000000000
698968

Corynebacterium\_diphtheriae\_HC02
Corynebacterium\_diphtheriae\_HC02
Corynebacterium\_diphtheriae\_HC02
Corynebacterium\_diphtheriae\_HC02
Corynebacterium\_diphtheriae\_HC02
Corynebacterium\_diphtheriae\_HC02
Corynebacterium\_diphtheriae\_HC02
Corynebacterium\_diphtheriae\_HC02
Corynebacterium\_diphtheriae\_HC02
Corynebacterium\_diphtheriae\_HC02
Corynebacterium\_diphtheriae\_HC02
Corynebacterium\_diphtheriae\_HC02
Corynebacterium\_diphtheriae\_HC02
Corynebacterium\_diphtheriae\_HC02
Corynebacterium\_diphtheriae\_HC02
Corynebacterium\_diphtheriae\_HC02
Corynebacterium\_diphtheriae\_HC02
Corynebacterium\_diphtheriae\_HC02
Corynebacterium\_diphtheriae\_HC02
Corynebacterium\_diphtheriae\_HC02
no rank

2.492.492.492.492.492.492.492.492.492.492.492.492.492.492.492.492.492.492.492.49
11111111111111111111
3.39792.609442.609442.609440010100000000000
115550010100000000000
257309

Corynebacterium\_diphtheriae\_NCTC\_13129
Corynebacterium\_diphtheriae\_NCTC\_13129
Corynebacterium\_diphtheriae\_NCTC\_13129
Corynebacterium\_diphtheriae\_NCTC\_13129
Corynebacterium\_diphtheriae\_NCTC\_13129
Corynebacterium\_diphtheriae\_NCTC\_13129
Corynebacterium\_diphtheriae\_NCTC\_13129
Corynebacterium\_diphtheriae\_NCTC\_13129
Corynebacterium\_diphtheriae\_NCTC\_13129
Corynebacterium\_diphtheriae\_NCTC\_13129
Corynebacterium\_diphtheriae\_NCTC\_13129
Corynebacterium\_diphtheriae\_NCTC\_13129
Corynebacterium\_diphtheriae\_NCTC\_13129
Corynebacterium\_diphtheriae\_NCTC\_13129
Corynebacterium\_diphtheriae\_NCTC\_13129
Corynebacterium\_diphtheriae\_NCTC\_13129
Corynebacterium\_diphtheriae\_NCTC\_13129
Corynebacterium\_diphtheriae\_NCTC\_13129
Corynebacterium\_diphtheriae\_NCTC\_13129
Corynebacterium\_diphtheriae\_NCTC\_13129
no rank

2.542.542.542.542.542.542.542.542.542.542.542.542.542.542.542.542.542.542.542.54
11111111111111111111
3.708053.302593.302593.1972211.6931511111111110000
15101091211111111110000
698962

Corynebacterium\_diphtheriae\_31A
Corynebacterium\_diphtheriae\_31A
Corynebacterium\_diphtheriae\_31A
Corynebacterium\_diphtheriae\_31A
Corynebacterium\_diphtheriae\_31A
Corynebacterium\_diphtheriae\_31A
Corynebacterium\_diphtheriae\_31A
Corynebacterium\_diphtheriae\_31A
Corynebacterium\_diphtheriae\_31A
Corynebacterium\_diphtheriae\_31A
Corynebacterium\_diphtheriae\_31A
Corynebacterium\_diphtheriae\_31A
Corynebacterium\_diphtheriae\_31A
Corynebacterium\_diphtheriae\_31A
Corynebacterium\_diphtheriae\_31A
Corynebacterium\_diphtheriae\_31A
Corynebacterium\_diphtheriae\_31A
Corynebacterium\_diphtheriae\_31A
Corynebacterium\_diphtheriae\_31A
Corynebacterium\_diphtheriae\_31A
no rank

2.432.432.432.432.432.432.432.432.432.432.432.432.432.432.432.432.432.432.432.43
11111111111111111111
3.484913.302593.079443.079441010000000000000
1210881010000000000000
698967

Corynebacterium\_diphtheriae\_HC01
Corynebacterium\_diphtheriae\_HC01
Corynebacterium\_diphtheriae\_HC01
Corynebacterium\_diphtheriae\_HC01
Corynebacterium\_diphtheriae\_HC01
Corynebacterium\_diphtheriae\_HC01
Corynebacterium\_diphtheriae\_HC01
Corynebacterium\_diphtheriae\_HC01
Corynebacterium\_diphtheriae\_HC01
Corynebacterium\_diphtheriae\_HC01
Corynebacterium\_diphtheriae\_HC01
Corynebacterium\_diphtheriae\_HC01
Corynebacterium\_diphtheriae\_HC01
Corynebacterium\_diphtheriae\_HC01
Corynebacterium\_diphtheriae\_HC01
Corynebacterium\_diphtheriae\_HC01
Corynebacterium\_diphtheriae\_HC01
Corynebacterium\_diphtheriae\_HC01
Corynebacterium\_diphtheriae\_HC01
Corynebacterium\_diphtheriae\_HC01
no rank

2.502.502.502.502.502.502.502.502.502.502.502.502.502.502.502.502.502.502.502.50
11111111111111111111
3.564953.302593.197222.945910000000000000000
1310970000000000000000
698963

Corynebacterium\_diphtheriae\_C7\_(beta)
Corynebacterium\_diphtheriae\_C7\_(beta)
Corynebacterium\_diphtheriae\_C7\_(beta)
Corynebacterium\_diphtheriae\_C7\_(beta)
Corynebacterium\_diphtheriae\_C7\_(beta)
Corynebacterium\_diphtheriae\_C7\_(beta)
Corynebacterium\_diphtheriae\_C7\_(beta)
Corynebacterium\_diphtheriae\_C7\_(beta)
Corynebacterium\_diphtheriae\_C7\_(beta)
Corynebacterium\_diphtheriae\_C7\_(beta)
Corynebacterium\_diphtheriae\_C7\_(beta)
Corynebacterium\_diphtheriae\_C7\_(beta)
Corynebacterium\_diphtheriae\_C7\_(beta)
Corynebacterium\_diphtheriae\_C7\_(beta)
Corynebacterium\_diphtheriae\_C7\_(beta)
Corynebacterium\_diphtheriae\_C7\_(beta)
Corynebacterium\_diphtheriae\_C7\_(beta)
Corynebacterium\_diphtheriae\_C7\_(beta)
Corynebacterium\_diphtheriae\_C7\_(beta)
Corynebacterium\_diphtheriae\_C7\_(beta)
no rank

2.452.452.452.452.452.452.452.452.452.452.452.452.452.452.452.452.452.452.452.45
11111111111111111111
3.639063.197223.079443.079441000100000000000
149881000100000000000
698972

Corynebacterium\_diphtheriae\_INCA\_402
Corynebacterium\_diphtheriae\_INCA\_402
Corynebacterium\_diphtheriae\_INCA\_402
Corynebacterium\_diphtheriae\_INCA\_402
Corynebacterium\_diphtheriae\_INCA\_402
Corynebacterium\_diphtheriae\_INCA\_402
Corynebacterium\_diphtheriae\_INCA\_402
Corynebacterium\_diphtheriae\_INCA\_402
Corynebacterium\_diphtheriae\_INCA\_402
Corynebacterium\_diphtheriae\_INCA\_402
Corynebacterium\_diphtheriae\_INCA\_402
Corynebacterium\_diphtheriae\_INCA\_402
Corynebacterium\_diphtheriae\_INCA\_402
Corynebacterium\_diphtheriae\_INCA\_402
Corynebacterium\_diphtheriae\_INCA\_402
Corynebacterium\_diphtheriae\_INCA\_402
Corynebacterium\_diphtheriae\_INCA\_402
Corynebacterium\_diphtheriae\_INCA\_402
Corynebacterium\_diphtheriae\_INCA\_402
Corynebacterium\_diphtheriae\_INCA\_402
no rank

2.482.482.482.482.482.482.482.482.482.482.482.482.482.482.482.482.482.482.482.48
11111111111111111111
3.772593.197223.197223.197221.69315000000000000000
169992000000000000000
698969

Corynebacterium\_diphtheriae\_HC03
Corynebacterium\_diphtheriae\_HC03
Corynebacterium\_diphtheriae\_HC03
Corynebacterium\_diphtheriae\_HC03
Corynebacterium\_diphtheriae\_HC03
Corynebacterium\_diphtheriae\_HC03
Corynebacterium\_diphtheriae\_HC03
Corynebacterium\_diphtheriae\_HC03
Corynebacterium\_diphtheriae\_HC03
Corynebacterium\_diphtheriae\_HC03
Corynebacterium\_diphtheriae\_HC03
Corynebacterium\_diphtheriae\_HC03
Corynebacterium\_diphtheriae\_HC03
Corynebacterium\_diphtheriae\_HC03
Corynebacterium\_diphtheriae\_HC03
Corynebacterium\_diphtheriae\_HC03
Corynebacterium\_diphtheriae\_HC03
Corynebacterium\_diphtheriae\_HC03
Corynebacterium\_diphtheriae\_HC03
Corynebacterium\_diphtheriae\_HC03
no rank

2.482.482.482.482.482.482.482.482.482.482.482.482.482.482.482.482.482.482.482.48
11111111111111111111
3.484913.302593.39793.197221.693151.6931511.693151.6931511111111111
12101192212211111111111
698970

Corynebacterium\_diphtheriae\_HC04
Corynebacterium\_diphtheriae\_HC04
Corynebacterium\_diphtheriae\_HC04
Corynebacterium\_diphtheriae\_HC04
Corynebacterium\_diphtheriae\_HC04
Corynebacterium\_diphtheriae\_HC04
Corynebacterium\_diphtheriae\_HC04
Corynebacterium\_diphtheriae\_HC04
Corynebacterium\_diphtheriae\_HC04
Corynebacterium\_diphtheriae\_HC04
Corynebacterium\_diphtheriae\_HC04
Corynebacterium\_diphtheriae\_HC04
Corynebacterium\_diphtheriae\_HC04
Corynebacterium\_diphtheriae\_HC04
Corynebacterium\_diphtheriae\_HC04
Corynebacterium\_diphtheriae\_HC04
Corynebacterium\_diphtheriae\_HC04
Corynebacterium\_diphtheriae\_HC04
Corynebacterium\_diphtheriae\_HC04
Corynebacterium\_diphtheriae\_HC04
no rank

2.432.432.432.432.432.432.432.432.432.432.432.432.432.432.432.432.432.432.432.43
11111111111111111111
3.639063.302593.197223.079441100000000000000
1410981100000000000000
698966

Corynebacterium\_diphtheriae\_241
Corynebacterium\_diphtheriae\_241
Corynebacterium\_diphtheriae\_241
Corynebacterium\_diphtheriae\_241
Corynebacterium\_diphtheriae\_241
Corynebacterium\_diphtheriae\_241
Corynebacterium\_diphtheriae\_241
Corynebacterium\_diphtheriae\_241
Corynebacterium\_diphtheriae\_241
Corynebacterium\_diphtheriae\_241
Corynebacterium\_diphtheriae\_241
Corynebacterium\_diphtheriae\_241
Corynebacterium\_diphtheriae\_241
Corynebacterium\_diphtheriae\_241
Corynebacterium\_diphtheriae\_241
Corynebacterium\_diphtheriae\_241
Corynebacterium\_diphtheriae\_241
Corynebacterium\_diphtheriae\_241
Corynebacterium\_diphtheriae\_241
Corynebacterium\_diphtheriae\_241
no rank

2.372.372.372.372.372.372.372.372.372.372.372.372.372.372.372.372.372.372.372.37
11111111111111111111
3.833213.39793.302593.302591.69315000100000000000
171110102000100000000000
43771
species

2.372.372.372.372.372.372.372.372.372.372.372.372.372.372.372.372.372.372.372.37
11111111111111111111
3.833213.39793.302593.302591.69315000100000000000
171110102000100000000000
504474

Corynebacterium\_urealyticum\_DSM\_7109
Corynebacterium\_urealyticum\_DSM\_7109
Corynebacterium\_urealyticum\_DSM\_7109
Corynebacterium\_urealyticum\_DSM\_7109
Corynebacterium\_urealyticum\_DSM\_7109
Corynebacterium\_urealyticum\_DSM\_7109
Corynebacterium\_urealyticum\_DSM\_7109
Corynebacterium\_urealyticum\_DSM\_7109
Corynebacterium\_urealyticum\_DSM\_7109
Corynebacterium\_urealyticum\_DSM\_7109
Corynebacterium\_urealyticum\_DSM\_7109
Corynebacterium\_urealyticum\_DSM\_7109
Corynebacterium\_urealyticum\_DSM\_7109
Corynebacterium\_urealyticum\_DSM\_7109
Corynebacterium\_urealyticum\_DSM\_7109
Corynebacterium\_urealyticum\_DSM\_7109
Corynebacterium\_urealyticum\_DSM\_7109
Corynebacterium\_urealyticum\_DSM\_7109
Corynebacterium\_urealyticum\_DSM\_7109
Corynebacterium\_urealyticum\_DSM\_7109
no rank

7.697.697.697.697.697.697.697.697.697.697.697.697.697.697.697.697.697.697.697.69
33333333333333333333
2.523912.523912.523912.462030.325098000000000000000
55541000000000000000
65058
species

2.612.612.612.612.612.612.612.612.612.612.612.612.612.612.612.612.612.612.612.61
11111111111111111111
2.791762.791762.791762.609440000000000000000
66650000000000000000
945712

Corynebacterium\_ulcerans\_BR-AD22
Corynebacterium\_ulcerans\_BR-AD22
Corynebacterium\_ulcerans\_BR-AD22
Corynebacterium\_ulcerans\_BR-AD22
Corynebacterium\_ulcerans\_BR-AD22
Corynebacterium\_ulcerans\_BR-AD22
Corynebacterium\_ulcerans\_BR-AD22
Corynebacterium\_ulcerans\_BR-AD22
Corynebacterium\_ulcerans\_BR-AD22
Corynebacterium\_ulcerans\_BR-AD22
Corynebacterium\_ulcerans\_BR-AD22
Corynebacterium\_ulcerans\_BR-AD22
Corynebacterium\_ulcerans\_BR-AD22
Corynebacterium\_ulcerans\_BR-AD22
Corynebacterium\_ulcerans\_BR-AD22
Corynebacterium\_ulcerans\_BR-AD22
Corynebacterium\_ulcerans\_BR-AD22
Corynebacterium\_ulcerans\_BR-AD22
Corynebacterium\_ulcerans\_BR-AD22
Corynebacterium\_ulcerans\_BR-AD22
no rank

2.582.582.582.582.582.582.582.582.582.582.582.582.582.582.582.582.582.582.582.58
11111111111111111111
2.386292.386292.386292.386290000000000000000
44440000000000000000
996634

Corynebacterium\_ulcerans\_0102
Corynebacterium\_ulcerans\_0102
Corynebacterium\_ulcerans\_0102
Corynebacterium\_ulcerans\_0102
Corynebacterium\_ulcerans\_0102
Corynebacterium\_ulcerans\_0102
Corynebacterium\_ulcerans\_0102
Corynebacterium\_ulcerans\_0102
Corynebacterium\_ulcerans\_0102
Corynebacterium\_ulcerans\_0102
Corynebacterium\_ulcerans\_0102
Corynebacterium\_ulcerans\_0102
Corynebacterium\_ulcerans\_0102
Corynebacterium\_ulcerans\_0102
Corynebacterium\_ulcerans\_0102
Corynebacterium\_ulcerans\_0102
Corynebacterium\_ulcerans\_0102
Corynebacterium\_ulcerans\_0102
Corynebacterium\_ulcerans\_0102
Corynebacterium\_ulcerans\_0102
no rank

2.502.502.502.502.502.502.502.502.502.502.502.502.502.502.502.502.502.502.502.50
11111111111111111111
2.386292.386292.386292.386291000000000000000
44441000000000000000
945711

Corynebacterium\_ulcerans\_809
Corynebacterium\_ulcerans\_809
Corynebacterium\_ulcerans\_809
Corynebacterium\_ulcerans\_809
Corynebacterium\_ulcerans\_809
Corynebacterium\_ulcerans\_809
Corynebacterium\_ulcerans\_809
Corynebacterium\_ulcerans\_809
Corynebacterium\_ulcerans\_809
Corynebacterium\_ulcerans\_809
Corynebacterium\_ulcerans\_809
Corynebacterium\_ulcerans\_809
Corynebacterium\_ulcerans\_809
Corynebacterium\_ulcerans\_809
Corynebacterium\_ulcerans\_809
Corynebacterium\_ulcerans\_809
Corynebacterium\_ulcerans\_809
Corynebacterium\_ulcerans\_809
Corynebacterium\_ulcerans\_809
Corynebacterium\_ulcerans\_809
no rank

3.223.223.223.223.223.223.223.223.223.223.223.223.223.223.223.223.223.223.223.22
11111111111111111111
4.044523.944443.995733.833211000000000000000
211920171000000000000000
152794
species

3.223.223.223.223.223.223.223.223.223.223.223.223.223.223.223.223.223.223.223.22
11111111111111111111
4.044523.944443.995733.833211000000000000000
211920171000000000000000
196164

Corynebacterium\_efficiens\_YS-314
Corynebacterium\_efficiens\_YS-314
Corynebacterium\_efficiens\_YS-314
Corynebacterium\_efficiens\_YS-314
Corynebacterium\_efficiens\_YS-314
Corynebacterium\_efficiens\_YS-314
Corynebacterium\_efficiens\_YS-314
Corynebacterium\_efficiens\_YS-314
Corynebacterium\_efficiens\_YS-314
Corynebacterium\_efficiens\_YS-314
Corynebacterium\_efficiens\_YS-314
Corynebacterium\_efficiens\_YS-314
Corynebacterium\_efficiens\_YS-314
Corynebacterium\_efficiens\_YS-314
Corynebacterium\_efficiens\_YS-314
Corynebacterium\_efficiens\_YS-314
Corynebacterium\_efficiens\_YS-314
Corynebacterium\_efficiens\_YS-314
Corynebacterium\_efficiens\_YS-314
Corynebacterium\_efficiens\_YS-314
no rank

2.452.452.452.452.452.452.452.452.452.452.452.452.452.452.452.452.452.452.452.45
11111111111111111111
2.098612.098612.098612.098610000000000000000
33330000000000000000
161879
species

2.452.452.452.452.452.452.452.452.452.452.452.452.452.452.452.452.452.452.452.45
11111111111111111111
2.098612.098612.098612.098610000000000000000
33330000000000000000
645127

Corynebacterium\_kroppenstedtii\_DSM\_44385
Corynebacterium\_kroppenstedtii\_DSM\_44385
Corynebacterium\_kroppenstedtii\_DSM\_44385
Corynebacterium\_kroppenstedtii\_DSM\_44385
Corynebacterium\_kroppenstedtii\_DSM\_44385
Corynebacterium\_kroppenstedtii\_DSM\_44385
Corynebacterium\_kroppenstedtii\_DSM\_44385
Corynebacterium\_kroppenstedtii\_DSM\_44385
Corynebacterium\_kroppenstedtii\_DSM\_44385
Corynebacterium\_kroppenstedtii\_DSM\_44385
Corynebacterium\_kroppenstedtii\_DSM\_44385
Corynebacterium\_kroppenstedtii\_DSM\_44385
Corynebacterium\_kroppenstedtii\_DSM\_44385
Corynebacterium\_kroppenstedtii\_DSM\_44385
Corynebacterium\_kroppenstedtii\_DSM\_44385
Corynebacterium\_kroppenstedtii\_DSM\_44385
Corynebacterium\_kroppenstedtii\_DSM\_44385
Corynebacterium\_kroppenstedtii\_DSM\_44385
Corynebacterium\_kroppenstedtii\_DSM\_44385
Corynebacterium\_kroppenstedtii\_DSM\_44385
no rank

2.602.602.602.602.602.602.602.602.602.602.602.602.602.602.602.602.602.602.602.60
11111111111111111111
3.833213.079442.945913.0794410101.6931500000000000
178781010200000000000
258224
species

2.602.602.602.602.602.602.602.602.602.602.602.602.602.602.602.602.602.602.602.60
11111111111111111111
3.833213.079442.945913.0794410101.6931500000000000
178781010200000000000
662755

Corynebacterium\_resistens\_DSM\_45100
Corynebacterium\_resistens\_DSM\_45100
Corynebacterium\_resistens\_DSM\_45100
Corynebacterium\_resistens\_DSM\_45100
Corynebacterium\_resistens\_DSM\_45100
Corynebacterium\_resistens\_DSM\_45100
Corynebacterium\_resistens\_DSM\_45100
Corynebacterium\_resistens\_DSM\_45100
Corynebacterium\_resistens\_DSM\_45100
Corynebacterium\_resistens\_DSM\_45100
Corynebacterium\_resistens\_DSM\_45100
Corynebacterium\_resistens\_DSM\_45100
Corynebacterium\_resistens\_DSM\_45100
Corynebacterium\_resistens\_DSM\_45100
Corynebacterium\_resistens\_DSM\_45100
Corynebacterium\_resistens\_DSM\_45100
Corynebacterium\_resistens\_DSM\_45100
Corynebacterium\_resistens\_DSM\_45100
Corynebacterium\_resistens\_DSM\_45100
Corynebacterium\_resistens\_DSM\_45100
no rank

34.8634.8634.8634.8634.8634.8634.8634.8634.8634.8634.8634.8634.8634.8634.8634.8634.8634.8634.8634.86
1515151515151515151515151515151515151515
2.413282.413282.413282.413280.7657820.1336780.13367800.3133090.06712560000000000
44441000100000000000
1719
species

2.332.332.332.332.332.332.332.332.332.332.332.332.332.332.332.332.332.332.332.33
11111111111111111111
2.386292.386292.386292.386290000000000000000
44440000000000000000
681645

Corynebacterium\_pseudotuberculosis\_C231
Corynebacterium\_pseudotuberculosis\_C231
Corynebacterium\_pseudotuberculosis\_C231
Corynebacterium\_pseudotuberculosis\_C231
Corynebacterium\_pseudotuberculosis\_C231
Corynebacterium\_pseudotuberculosis\_C231
Corynebacterium\_pseudotuberculosis\_C231
Corynebacterium\_pseudotuberculosis\_C231
Corynebacterium\_pseudotuberculosis\_C231
Corynebacterium\_pseudotuberculosis\_C231
Corynebacterium\_pseudotuberculosis\_C231
Corynebacterium\_pseudotuberculosis\_C231
Corynebacterium\_pseudotuberculosis\_C231
Corynebacterium\_pseudotuberculosis\_C231
Corynebacterium\_pseudotuberculosis\_C231
Corynebacterium\_pseudotuberculosis\_C231
Corynebacterium\_pseudotuberculosis\_C231
Corynebacterium\_pseudotuberculosis\_C231
Corynebacterium\_pseudotuberculosis\_C231
Corynebacterium\_pseudotuberculosis\_C231
no rank

2.282.282.282.282.282.282.282.282.282.282.282.282.282.282.282.282.282.282.282.28
11111111111111111111
2.386292.386292.386292.386290000000000000000
44440000000000000000
1087454

Corynebacterium\_pseudotuberculosis\_1\_06-A
Corynebacterium\_pseudotuberculosis\_1\_06-A
Corynebacterium\_pseudotuberculosis\_1\_06-A
Corynebacterium\_pseudotuberculosis\_1\_06-A
Corynebacterium\_pseudotuberculosis\_1\_06-A
Corynebacterium\_pseudotuberculosis\_1\_06-A
Corynebacterium\_pseudotuberculosis\_1\_06-A
Corynebacterium\_pseudotuberculosis\_1\_06-A
Corynebacterium\_pseudotuberculosis\_1\_06-A
Corynebacterium\_pseudotuberculosis\_1\_06-A
Corynebacterium\_pseudotuberculosis\_1\_06-A
Corynebacterium\_pseudotuberculosis\_1\_06-A
Corynebacterium\_pseudotuberculosis\_1\_06-A
Corynebacterium\_pseudotuberculosis\_1\_06-A
Corynebacterium\_pseudotuberculosis\_1\_06-A
Corynebacterium\_pseudotuberculosis\_1\_06-A
Corynebacterium\_pseudotuberculosis\_1\_06-A
Corynebacterium\_pseudotuberculosis\_1\_06-A
Corynebacterium\_pseudotuberculosis\_1\_06-A
Corynebacterium\_pseudotuberculosis\_1\_06-A
no rank

2.342.342.342.342.342.342.342.342.342.342.342.342.342.342.342.342.342.342.342.34
11111111111111111111
2.386292.386292.386292.386290000010000000000
44440000010000000000
765874

Corynebacterium\_pseudotuberculosis\_FRC41
Corynebacterium\_pseudotuberculosis\_FRC41
Corynebacterium\_pseudotuberculosis\_FRC41
Corynebacterium\_pseudotuberculosis\_FRC41
Corynebacterium\_pseudotuberculosis\_FRC41
Corynebacterium\_pseudotuberculosis\_FRC41
Corynebacterium\_pseudotuberculosis\_FRC41
Corynebacterium\_pseudotuberculosis\_FRC41
Corynebacterium\_pseudotuberculosis\_FRC41
Corynebacterium\_pseudotuberculosis\_FRC41
Corynebacterium\_pseudotuberculosis\_FRC41
Corynebacterium\_pseudotuberculosis\_FRC41
Corynebacterium\_pseudotuberculosis\_FRC41
Corynebacterium\_pseudotuberculosis\_FRC41
Corynebacterium\_pseudotuberculosis\_FRC41
Corynebacterium\_pseudotuberculosis\_FRC41
Corynebacterium\_pseudotuberculosis\_FRC41
Corynebacterium\_pseudotuberculosis\_FRC41
Corynebacterium\_pseudotuberculosis\_FRC41
Corynebacterium\_pseudotuberculosis\_FRC41
no rank

2.322.322.322.322.322.322.322.322.322.322.322.322.322.322.322.322.322.322.322.32
11111111111111111111
2.791762.791762.791762.791761110100000000000
66661110100000000000
935697

Corynebacterium\_pseudotuberculosis\_CIP\_52.97
Corynebacterium\_pseudotuberculosis\_CIP\_52.97
Corynebacterium\_pseudotuberculosis\_CIP\_52.97
Corynebacterium\_pseudotuberculosis\_CIP\_52.97
Corynebacterium\_pseudotuberculosis\_CIP\_52.97
Corynebacterium\_pseudotuberculosis\_CIP\_52.97
Corynebacterium\_pseudotuberculosis\_CIP\_52.97
Corynebacterium\_pseudotuberculosis\_CIP\_52.97
Corynebacterium\_pseudotuberculosis\_CIP\_52.97
Corynebacterium\_pseudotuberculosis\_CIP\_52.97
Corynebacterium\_pseudotuberculosis\_CIP\_52.97
Corynebacterium\_pseudotuberculosis\_CIP\_52.97
Corynebacterium\_pseudotuberculosis\_CIP\_52.97
Corynebacterium\_pseudotuberculosis\_CIP\_52.97
Corynebacterium\_pseudotuberculosis\_CIP\_52.97
Corynebacterium\_pseudotuberculosis\_CIP\_52.97
Corynebacterium\_pseudotuberculosis\_CIP\_52.97
Corynebacterium\_pseudotuberculosis\_CIP\_52.97
Corynebacterium\_pseudotuberculosis\_CIP\_52.97
Corynebacterium\_pseudotuberculosis\_CIP\_52.97
no rank

2.302.302.302.302.302.302.302.302.302.302.302.302.302.302.302.302.302.302.302.30
11111111111111111111
2.386292.386292.386292.386290000100000000000
44440000100000000000
1087451

Corynebacterium\_pseudotuberculosis\_31
Corynebacterium\_pseudotuberculosis\_31
Corynebacterium\_pseudotuberculosis\_31
Corynebacterium\_pseudotuberculosis\_31
Corynebacterium\_pseudotuberculosis\_31
Corynebacterium\_pseudotuberculosis\_31
Corynebacterium\_pseudotuberculosis\_31
Corynebacterium\_pseudotuberculosis\_31
Corynebacterium\_pseudotuberculosis\_31
Corynebacterium\_pseudotuberculosis\_31
Corynebacterium\_pseudotuberculosis\_31
Corynebacterium\_pseudotuberculosis\_31
Corynebacterium\_pseudotuberculosis\_31
Corynebacterium\_pseudotuberculosis\_31
Corynebacterium\_pseudotuberculosis\_31
Corynebacterium\_pseudotuberculosis\_31
Corynebacterium\_pseudotuberculosis\_31
Corynebacterium\_pseudotuberculosis\_31
Corynebacterium\_pseudotuberculosis\_31
Corynebacterium\_pseudotuberculosis\_31
no rank

2.342.342.342.342.342.342.342.342.342.342.342.342.342.342.342.342.342.342.342.34
11111111111111111111
2.386292.386292.386292.386291.69315000000000000000
44442000000000000000
935298

Corynebacterium\_pseudotuberculosis\_PAT10
Corynebacterium\_pseudotuberculosis\_PAT10
Corynebacterium\_pseudotuberculosis\_PAT10
Corynebacterium\_pseudotuberculosis\_PAT10
Corynebacterium\_pseudotuberculosis\_PAT10
Corynebacterium\_pseudotuberculosis\_PAT10
Corynebacterium\_pseudotuberculosis\_PAT10
Corynebacterium\_pseudotuberculosis\_PAT10
Corynebacterium\_pseudotuberculosis\_PAT10
Corynebacterium\_pseudotuberculosis\_PAT10
Corynebacterium\_pseudotuberculosis\_PAT10
Corynebacterium\_pseudotuberculosis\_PAT10
Corynebacterium\_pseudotuberculosis\_PAT10
Corynebacterium\_pseudotuberculosis\_PAT10
Corynebacterium\_pseudotuberculosis\_PAT10
Corynebacterium\_pseudotuberculosis\_PAT10
Corynebacterium\_pseudotuberculosis\_PAT10
Corynebacterium\_pseudotuberculosis\_PAT10
Corynebacterium\_pseudotuberculosis\_PAT10
Corynebacterium\_pseudotuberculosis\_PAT10
no rank

2.292.292.292.292.292.292.292.292.292.292.292.292.292.292.292.292.292.292.292.29
11111111111111111111
2.386292.386292.386292.386291.69315000000000000000
44442000000000000000
1161911

Corynebacterium\_pseudotuberculosis\_Cp162
Corynebacterium\_pseudotuberculosis\_Cp162
Corynebacterium\_pseudotuberculosis\_Cp162
Corynebacterium\_pseudotuberculosis\_Cp162
Corynebacterium\_pseudotuberculosis\_Cp162
Corynebacterium\_pseudotuberculosis\_Cp162
Corynebacterium\_pseudotuberculosis\_Cp162
Corynebacterium\_pseudotuberculosis\_Cp162
Corynebacterium\_pseudotuberculosis\_Cp162
Corynebacterium\_pseudotuberculosis\_Cp162
Corynebacterium\_pseudotuberculosis\_Cp162
Corynebacterium\_pseudotuberculosis\_Cp162
Corynebacterium\_pseudotuberculosis\_Cp162
Corynebacterium\_pseudotuberculosis\_Cp162
Corynebacterium\_pseudotuberculosis\_Cp162
Corynebacterium\_pseudotuberculosis\_Cp162
Corynebacterium\_pseudotuberculosis\_Cp162
Corynebacterium\_pseudotuberculosis\_Cp162
Corynebacterium\_pseudotuberculosis\_Cp162
Corynebacterium\_pseudotuberculosis\_Cp162
no rank

2.342.342.342.342.342.342.342.342.342.342.342.342.342.342.342.342.342.342.342.34
11111111111111111111
2.386292.386292.386292.386291.69315000000000000000
44442000000000000000
889513

Corynebacterium\_pseudotuberculosis\_I19
Corynebacterium\_pseudotuberculosis\_I19
Corynebacterium\_pseudotuberculosis\_I19
Corynebacterium\_pseudotuberculosis\_I19
Corynebacterium\_pseudotuberculosis\_I19
Corynebacterium\_pseudotuberculosis\_I19
Corynebacterium\_pseudotuberculosis\_I19
Corynebacterium\_pseudotuberculosis\_I19
Corynebacterium\_pseudotuberculosis\_I19
Corynebacterium\_pseudotuberculosis\_I19
Corynebacterium\_pseudotuberculosis\_I19
Corynebacterium\_pseudotuberculosis\_I19
Corynebacterium\_pseudotuberculosis\_I19
Corynebacterium\_pseudotuberculosis\_I19
Corynebacterium\_pseudotuberculosis\_I19
Corynebacterium\_pseudotuberculosis\_I19
Corynebacterium\_pseudotuberculosis\_I19
Corynebacterium\_pseudotuberculosis\_I19
Corynebacterium\_pseudotuberculosis\_I19
Corynebacterium\_pseudotuberculosis\_I19
no rank

2.342.342.342.342.342.342.342.342.342.342.342.342.342.342.342.342.342.342.342.34
11111111111111111111
2.386292.386292.386292.386291.69315000000000000000
44442000000000000000
1087453

Corynebacterium\_pseudotuberculosis\_42\_02-A
Corynebacterium\_pseudotuberculosis\_42\_02-A
Corynebacterium\_pseudotuberculosis\_42\_02-A
Corynebacterium\_pseudotuberculosis\_42\_02-A
Corynebacterium\_pseudotuberculosis\_42\_02-A
Corynebacterium\_pseudotuberculosis\_42\_02-A
Corynebacterium\_pseudotuberculosis\_42\_02-A
Corynebacterium\_pseudotuberculosis\_42\_02-A
Corynebacterium\_pseudotuberculosis\_42\_02-A
Corynebacterium\_pseudotuberculosis\_42\_02-A
Corynebacterium\_pseudotuberculosis\_42\_02-A
Corynebacterium\_pseudotuberculosis\_42\_02-A
Corynebacterium\_pseudotuberculosis\_42\_02-A
Corynebacterium\_pseudotuberculosis\_42\_02-A
Corynebacterium\_pseudotuberculosis\_42\_02-A
Corynebacterium\_pseudotuberculosis\_42\_02-A
Corynebacterium\_pseudotuberculosis\_42\_02-A
Corynebacterium\_pseudotuberculosis\_42\_02-A
Corynebacterium\_pseudotuberculosis\_42\_02-A
Corynebacterium\_pseudotuberculosis\_42\_02-A
no rank

2.312.312.312.312.312.312.312.312.312.312.312.312.312.312.312.312.312.312.312.31
11111111111111111111
2.386292.386292.386292.386290000000000000000
44440000000000000000
1168865

Corynebacterium\_pseudotuberculosis\_258
Corynebacterium\_pseudotuberculosis\_258
Corynebacterium\_pseudotuberculosis\_258
Corynebacterium\_pseudotuberculosis\_258
Corynebacterium\_pseudotuberculosis\_258
Corynebacterium\_pseudotuberculosis\_258
Corynebacterium\_pseudotuberculosis\_258
Corynebacterium\_pseudotuberculosis\_258
Corynebacterium\_pseudotuberculosis\_258
Corynebacterium\_pseudotuberculosis\_258
Corynebacterium\_pseudotuberculosis\_258
Corynebacterium\_pseudotuberculosis\_258
Corynebacterium\_pseudotuberculosis\_258
Corynebacterium\_pseudotuberculosis\_258
Corynebacterium\_pseudotuberculosis\_258
Corynebacterium\_pseudotuberculosis\_258
Corynebacterium\_pseudotuberculosis\_258
Corynebacterium\_pseudotuberculosis\_258
Corynebacterium\_pseudotuberculosis\_258
Corynebacterium\_pseudotuberculosis\_258
no rank

2.342.342.342.342.342.342.342.342.342.342.342.342.342.342.342.342.342.342.342.34
11111111111111111111
2.386292.386292.386292.3862900001.6931500000000000
44440000200000000000
1087452

Corynebacterium\_pseudotuberculosis\_3\_99-5
Corynebacterium\_pseudotuberculosis\_3\_99-5
Corynebacterium\_pseudotuberculosis\_3\_99-5
Corynebacterium\_pseudotuberculosis\_3\_99-5
Corynebacterium\_pseudotuberculosis\_3\_99-5
Corynebacterium\_pseudotuberculosis\_3\_99-5
Corynebacterium\_pseudotuberculosis\_3\_99-5
Corynebacterium\_pseudotuberculosis\_3\_99-5
Corynebacterium\_pseudotuberculosis\_3\_99-5
Corynebacterium\_pseudotuberculosis\_3\_99-5
Corynebacterium\_pseudotuberculosis\_3\_99-5
Corynebacterium\_pseudotuberculosis\_3\_99-5
Corynebacterium\_pseudotuberculosis\_3\_99-5
Corynebacterium\_pseudotuberculosis\_3\_99-5
Corynebacterium\_pseudotuberculosis\_3\_99-5
Corynebacterium\_pseudotuberculosis\_3\_99-5
Corynebacterium\_pseudotuberculosis\_3\_99-5
Corynebacterium\_pseudotuberculosis\_3\_99-5
Corynebacterium\_pseudotuberculosis\_3\_99-5
Corynebacterium\_pseudotuberculosis\_3\_99-5
no rank

2.342.342.342.342.342.342.342.342.342.342.342.342.342.342.342.342.342.342.342.34
11111111111111111111
2.386292.386292.386292.386291.69315010000000000000
44442010000000000000
1089446

Corynebacterium\_pseudotuberculosis\_267
Corynebacterium\_pseudotuberculosis\_267
Corynebacterium\_pseudotuberculosis\_267
Corynebacterium\_pseudotuberculosis\_267
Corynebacterium\_pseudotuberculosis\_267
Corynebacterium\_pseudotuberculosis\_267
Corynebacterium\_pseudotuberculosis\_267
Corynebacterium\_pseudotuberculosis\_267
Corynebacterium\_pseudotuberculosis\_267
Corynebacterium\_pseudotuberculosis\_267
Corynebacterium\_pseudotuberculosis\_267
Corynebacterium\_pseudotuberculosis\_267
Corynebacterium\_pseudotuberculosis\_267
Corynebacterium\_pseudotuberculosis\_267
Corynebacterium\_pseudotuberculosis\_267
Corynebacterium\_pseudotuberculosis\_267
Corynebacterium\_pseudotuberculosis\_267
Corynebacterium\_pseudotuberculosis\_267
Corynebacterium\_pseudotuberculosis\_267
Corynebacterium\_pseudotuberculosis\_267
no rank

2.342.342.342.342.342.342.342.342.342.342.342.342.342.342.342.342.342.342.342.34
11111111111111111111
2.386292.386292.386292.386291100100000000000
44441100100000000000
1117942

Corynebacterium\_pseudotuberculosis\_P54B96
Corynebacterium\_pseudotuberculosis\_P54B96
Corynebacterium\_pseudotuberculosis\_P54B96
Corynebacterium\_pseudotuberculosis\_P54B96
Corynebacterium\_pseudotuberculosis\_P54B96
Corynebacterium\_pseudotuberculosis\_P54B96
Corynebacterium\_pseudotuberculosis\_P54B96
Corynebacterium\_pseudotuberculosis\_P54B96
Corynebacterium\_pseudotuberculosis\_P54B96
Corynebacterium\_pseudotuberculosis\_P54B96
Corynebacterium\_pseudotuberculosis\_P54B96
Corynebacterium\_pseudotuberculosis\_P54B96
Corynebacterium\_pseudotuberculosis\_P54B96
Corynebacterium\_pseudotuberculosis\_P54B96
Corynebacterium\_pseudotuberculosis\_P54B96
Corynebacterium\_pseudotuberculosis\_P54B96
Corynebacterium\_pseudotuberculosis\_P54B96
Corynebacterium\_pseudotuberculosis\_P54B96
Corynebacterium\_pseudotuberculosis\_P54B96
Corynebacterium\_pseudotuberculosis\_P54B96
no rank

2.342.342.342.342.342.342.342.342.342.342.342.342.342.342.342.342.342.342.342.34
11111111111111111111
2.386292.386292.386292.386290000000000000000
44440000000000000000
679896

Corynebacterium\_pseudotuberculosis\_1002
Corynebacterium\_pseudotuberculosis\_1002
Corynebacterium\_pseudotuberculosis\_1002
Corynebacterium\_pseudotuberculosis\_1002
Corynebacterium\_pseudotuberculosis\_1002
Corynebacterium\_pseudotuberculosis\_1002
Corynebacterium\_pseudotuberculosis\_1002
Corynebacterium\_pseudotuberculosis\_1002
Corynebacterium\_pseudotuberculosis\_1002
Corynebacterium\_pseudotuberculosis\_1002
Corynebacterium\_pseudotuberculosis\_1002
Corynebacterium\_pseudotuberculosis\_1002
Corynebacterium\_pseudotuberculosis\_1002
Corynebacterium\_pseudotuberculosis\_1002
Corynebacterium\_pseudotuberculosis\_1002
Corynebacterium\_pseudotuberculosis\_1002
Corynebacterium\_pseudotuberculosis\_1002
Corynebacterium\_pseudotuberculosis\_1002
Corynebacterium\_pseudotuberculosis\_1002
Corynebacterium\_pseudotuberculosis\_1002
no rank

2.312.312.312.312.312.312.312.312.312.312.312.312.312.312.312.312.312.312.312.31
11111111111111111111
2.386292.386292.386292.386291000000000000000
44441000000000000000
1074485

Corynebacterium\_pseudotuberculosis\_316
Corynebacterium\_pseudotuberculosis\_316
Corynebacterium\_pseudotuberculosis\_316
Corynebacterium\_pseudotuberculosis\_316
Corynebacterium\_pseudotuberculosis\_316
Corynebacterium\_pseudotuberculosis\_316
Corynebacterium\_pseudotuberculosis\_316
Corynebacterium\_pseudotuberculosis\_316
Corynebacterium\_pseudotuberculosis\_316
Corynebacterium\_pseudotuberculosis\_316
Corynebacterium\_pseudotuberculosis\_316
Corynebacterium\_pseudotuberculosis\_316
Corynebacterium\_pseudotuberculosis\_316
Corynebacterium\_pseudotuberculosis\_316
Corynebacterium\_pseudotuberculosis\_316
Corynebacterium\_pseudotuberculosis\_316
Corynebacterium\_pseudotuberculosis\_316
Corynebacterium\_pseudotuberculosis\_316
Corynebacterium\_pseudotuberculosis\_316
Corynebacterium\_pseudotuberculosis\_316
no rank

9.959.959.959.959.959.959.959.959.959.959.959.959.959.959.959.959.959.959.959.95
33333333333333333333
3.697713.473033.414153.190191.228490000.33768800000000000
15121191000100000000000
1718
species

3.363.363.363.363.363.363.363.363.363.363.363.363.363.363.363.363.363.363.363.36
11111111111111111111
2.945913.079443.197222.791761000100000000000
78961000100000000000
340322

Corynebacterium\_glutamicum\_R
Corynebacterium\_glutamicum\_R
Corynebacterium\_glutamicum\_R
Corynebacterium\_glutamicum\_R
Corynebacterium\_glutamicum\_R
Corynebacterium\_glutamicum\_R
Corynebacterium\_glutamicum\_R
Corynebacterium\_glutamicum\_R
Corynebacterium\_glutamicum\_R
Corynebacterium\_glutamicum\_R
Corynebacterium\_glutamicum\_R
Corynebacterium\_glutamicum\_R
Corynebacterium\_glutamicum\_R
Corynebacterium\_glutamicum\_R
Corynebacterium\_glutamicum\_R
Corynebacterium\_glutamicum\_R
Corynebacterium\_glutamicum\_R
Corynebacterium\_glutamicum\_R
Corynebacterium\_glutamicum\_R
Corynebacterium\_glutamicum\_R
no rank

6.596.596.596.596.596.596.596.596.596.596.596.596.596.596.596.596.596.596.596.59
22222222222222222222
4.081033.673713.524753.393331.345000000000000000
221412111000000000000000
196627

Corynebacterium\_glutamicum\_ATCC\_13032Corynebacterium\_glutamicum\_ATCC\_13032
Corynebacterium\_glutamicum\_ATCC\_13032Corynebacterium\_glutamicum\_ATCC\_13032
Corynebacterium\_glutamicum\_ATCC\_13032Corynebacterium\_glutamicum\_ATCC\_13032
Corynebacterium\_glutamicum\_ATCC\_13032Corynebacterium\_glutamicum\_ATCC\_13032
Corynebacterium\_glutamicum\_ATCC\_13032Corynebacterium\_glutamicum\_ATCC\_13032
Corynebacterium\_glutamicum\_ATCC\_13032Corynebacterium\_glutamicum\_ATCC\_13032
Corynebacterium\_glutamicum\_ATCC\_13032Corynebacterium\_glutamicum\_ATCC\_13032
Corynebacterium\_glutamicum\_ATCC\_13032Corynebacterium\_glutamicum\_ATCC\_13032
Corynebacterium\_glutamicum\_ATCC\_13032Corynebacterium\_glutamicum\_ATCC\_13032
Corynebacterium\_glutamicum\_ATCC\_13032Corynebacterium\_glutamicum\_ATCC\_13032
Corynebacterium\_glutamicum\_ATCC\_13032Corynebacterium\_glutamicum\_ATCC\_13032
Corynebacterium\_glutamicum\_ATCC\_13032Corynebacterium\_glutamicum\_ATCC\_13032
Corynebacterium\_glutamicum\_ATCC\_13032Corynebacterium\_glutamicum\_ATCC\_13032
Corynebacterium\_glutamicum\_ATCC\_13032Corynebacterium\_glutamicum\_ATCC\_13032
Corynebacterium\_glutamicum\_ATCC\_13032Corynebacterium\_glutamicum\_ATCC\_13032
Corynebacterium\_glutamicum\_ATCC\_13032Corynebacterium\_glutamicum\_ATCC\_13032
Corynebacterium\_glutamicum\_ATCC\_13032Corynebacterium\_glutamicum\_ATCC\_13032
Corynebacterium\_glutamicum\_ATCC\_13032Corynebacterium\_glutamicum\_ATCC\_13032
Corynebacterium\_glutamicum\_ATCC\_13032Corynebacterium\_glutamicum\_ATCC\_13032
Corynebacterium\_glutamicum\_ATCC\_13032Corynebacterium\_glutamicum\_ATCC\_13032
no rank

3.433.433.433.433.433.433.433.433.433.433.433.433.433.433.433.433.433.433.433.43
11111111111111111111
4.40124.36734.218884.33222.609442.609442.098612.098612.945912.386292.098612.0986111000000
302925285533743311000000
1727
species

3.433.433.433.433.433.433.433.433.433.433.433.433.433.433.433.433.433.433.433.43
11111111111111111111
4.40124.36734.218884.33222.609442.609442.098612.098612.945912.386292.098612.0986111000000
302925285533743311000000
858619

Corynebacterium\_variabile\_DSM\_44702
Corynebacterium\_variabile\_DSM\_44702
Corynebacterium\_variabile\_DSM\_44702
Corynebacterium\_variabile\_DSM\_44702
Corynebacterium\_variabile\_DSM\_44702
Corynebacterium\_variabile\_DSM\_44702
Corynebacterium\_variabile\_DSM\_44702
Corynebacterium\_variabile\_DSM\_44702
Corynebacterium\_variabile\_DSM\_44702
Corynebacterium\_variabile\_DSM\_44702
Corynebacterium\_variabile\_DSM\_44702
Corynebacterium\_variabile\_DSM\_44702
Corynebacterium\_variabile\_DSM\_44702
Corynebacterium\_variabile\_DSM\_44702
Corynebacterium\_variabile\_DSM\_44702
Corynebacterium\_variabile\_DSM\_44702
Corynebacterium\_variabile\_DSM\_44702
Corynebacterium\_variabile\_DSM\_44702
Corynebacterium\_variabile\_DSM\_44702
Corynebacterium\_variabile\_DSM\_44702
no rank

2.482.482.482.482.482.482.482.482.482.482.482.482.482.482.482.482.482.482.482.48
11111111111111111111
4.33224.135493.944443.944441000010000000000
282319191000010000000000
38289
species

2.482.482.482.482.482.482.482.482.482.482.482.482.482.482.482.482.482.482.482.48
11111111111111111111
4.33224.135493.944443.944441000010000000000
282319191000010000000000
306537

Corynebacterium\_jeikeium\_K411
Corynebacterium\_jeikeium\_K411
Corynebacterium\_jeikeium\_K411
Corynebacterium\_jeikeium\_K411
Corynebacterium\_jeikeium\_K411
Corynebacterium\_jeikeium\_K411
Corynebacterium\_jeikeium\_K411
Corynebacterium\_jeikeium\_K411
Corynebacterium\_jeikeium\_K411
Corynebacterium\_jeikeium\_K411
Corynebacterium\_jeikeium\_K411
Corynebacterium\_jeikeium\_K411
Corynebacterium\_jeikeium\_K411
Corynebacterium\_jeikeium\_K411
Corynebacterium\_jeikeium\_K411
Corynebacterium\_jeikeium\_K411
Corynebacterium\_jeikeium\_K411
Corynebacterium\_jeikeium\_K411
Corynebacterium\_jeikeium\_K411
Corynebacterium\_jeikeium\_K411
no rank

52.3952.3952.3952.3952.3952.3952.3952.3952.3952.3952.3952.3952.3952.3952.3952.3952.3952.3952.3952.39
77777777777777777777
3.684313.547053.572173.501511.27940.9735360.5701580.5701580.9015850.7387020.5701580.5168940.4831370.4831370.3885580.4418220.313486000
151313121111111111111000
85025
family

21.9221.9221.9221.9221.9221.9221.9221.9221.9221.9221.9221.9221.9221.9221.9221.9221.9221.9221.9221.92
33333333333333333333
2.648212.609892.705992.565660.430657000000000000000
55651000000000000000
1817
genus

6.296.296.296.296.296.296.296.296.296.296.296.296.296.296.296.296.296.296.296.29
11111111111111111111
3.079442.945912.945912.791760000000000000000
87760000000000000000
37329
species

6.296.296.296.296.296.296.296.296.296.296.296.296.296.296.296.296.296.296.296.29
11111111111111111111
3.079442.945912.945912.791760000000000000000
87760000000000000000
247156

Nocardia\_farcinica\_IFM\_10152
Nocardia\_farcinica\_IFM\_10152
Nocardia\_farcinica\_IFM\_10152
Nocardia\_farcinica\_IFM\_10152
Nocardia\_farcinica\_IFM\_10152
Nocardia\_farcinica\_IFM\_10152
Nocardia\_farcinica\_IFM\_10152
Nocardia\_farcinica\_IFM\_10152
Nocardia\_farcinica\_IFM\_10152
Nocardia\_farcinica\_IFM\_10152
Nocardia\_farcinica\_IFM\_10152
Nocardia\_farcinica\_IFM\_10152
Nocardia\_farcinica\_IFM\_10152
Nocardia\_farcinica\_IFM\_10152
Nocardia\_farcinica\_IFM\_10152
Nocardia\_farcinica\_IFM\_10152
Nocardia\_farcinica\_IFM\_10152
Nocardia\_farcinica\_IFM\_10152
Nocardia\_farcinica\_IFM\_10152
Nocardia\_farcinica\_IFM\_10152
no rank

9.449.449.449.449.449.449.449.449.449.449.449.449.449.449.449.449.449.449.449.44
11111111111111111111
2.386292.386292.609442.386291000000000000000
44541000000000000000
37326
species

9.449.449.449.449.449.449.449.449.449.449.449.449.449.449.449.449.449.449.449.44
11111111111111111111
2.386292.386292.609442.386291000000000000000
44541000000000000000
1133849

Nocardia\_brasiliensis\_ATCC\_700358
Nocardia\_brasiliensis\_ATCC\_700358
Nocardia\_brasiliensis\_ATCC\_700358
Nocardia\_brasiliensis\_ATCC\_700358
Nocardia\_brasiliensis\_ATCC\_700358
Nocardia\_brasiliensis\_ATCC\_700358
Nocardia\_brasiliensis\_ATCC\_700358
Nocardia\_brasiliensis\_ATCC\_700358
Nocardia\_brasiliensis\_ATCC\_700358
Nocardia\_brasiliensis\_ATCC\_700358
Nocardia\_brasiliensis\_ATCC\_700358
Nocardia\_brasiliensis\_ATCC\_700358
Nocardia\_brasiliensis\_ATCC\_700358
Nocardia\_brasiliensis\_ATCC\_700358
Nocardia\_brasiliensis\_ATCC\_700358
Nocardia\_brasiliensis\_ATCC\_700358
Nocardia\_brasiliensis\_ATCC\_700358
Nocardia\_brasiliensis\_ATCC\_700358
Nocardia\_brasiliensis\_ATCC\_700358
Nocardia\_brasiliensis\_ATCC\_700358
no rank

6.196.196.196.196.196.196.196.196.196.196.196.196.196.196.196.196.196.196.196.19
11111111111111111111
2.609442.609442.609442.609440000000000000000
55550000000000000000
135487
species

6.196.196.196.196.196.196.196.196.196.196.196.196.196.196.196.196.196.196.196.19
11111111111111111111
2.609442.609442.609442.609440000000000000000
55550000000000000000
1127134

Nocardia\_cyriacigeorgica\_GUH-2
Nocardia\_cyriacigeorgica\_GUH-2
Nocardia\_cyriacigeorgica\_GUH-2
Nocardia\_cyriacigeorgica\_GUH-2
Nocardia\_cyriacigeorgica\_GUH-2
Nocardia\_cyriacigeorgica\_GUH-2
Nocardia\_cyriacigeorgica\_GUH-2
Nocardia\_cyriacigeorgica\_GUH-2
Nocardia\_cyriacigeorgica\_GUH-2
Nocardia\_cyriacigeorgica\_GUH-2
Nocardia\_cyriacigeorgica\_GUH-2
Nocardia\_cyriacigeorgica\_GUH-2
Nocardia\_cyriacigeorgica\_GUH-2
Nocardia\_cyriacigeorgica\_GUH-2
Nocardia\_cyriacigeorgica\_GUH-2
Nocardia\_cyriacigeorgica\_GUH-2
Nocardia\_cyriacigeorgica\_GUH-2
Nocardia\_cyriacigeorgica\_GUH-2
Nocardia\_cyriacigeorgica\_GUH-2
Nocardia\_cyriacigeorgica\_GUH-2
no rank

30.4730.4730.4730.4730.4730.4730.4730.4730.4730.4730.4730.4730.4730.4730.4730.4730.4730.4730.4730.47
44444444444444444444
4.429684.221244.19534.174761.889981.673890.9803280.9803281.550181.270120.9803280.8887450.8307040.8307040.6680850.7596670.539006000
312524242211211111111000
1827
genus

6.906.906.906.906.906.906.906.906.906.906.906.906.906.906.906.906.906.906.906.90
11111111111111111111
3.944443.772593.772593.708051.69315000000000000000
191616152000000000000000
1833
species

6.906.906.906.906.906.906.906.906.906.906.906.906.906.906.906.906.906.906.906.90
11111111111111111111
3.944443.772593.772593.708051.69315000000000000000
191616152000000000000000
234621

Rhodococcus\_erythropolis\_PR4
Rhodococcus\_erythropolis\_PR4
Rhodococcus\_erythropolis\_PR4
Rhodococcus\_erythropolis\_PR4
Rhodococcus\_erythropolis\_PR4
Rhodococcus\_erythropolis\_PR4
Rhodococcus\_erythropolis\_PR4
Rhodococcus\_erythropolis\_PR4
Rhodococcus\_erythropolis\_PR4
Rhodococcus\_erythropolis\_PR4
Rhodococcus\_erythropolis\_PR4
Rhodococcus\_erythropolis\_PR4
Rhodococcus\_erythropolis\_PR4
Rhodococcus\_erythropolis\_PR4
Rhodococcus\_erythropolis\_PR4
Rhodococcus\_erythropolis\_PR4
Rhodococcus\_erythropolis\_PR4
Rhodococcus\_erythropolis\_PR4
Rhodococcus\_erythropolis\_PR4
Rhodococcus\_erythropolis\_PR4
no rank

9.709.709.709.709.709.709.709.709.709.709.709.709.709.709.709.709.709.709.709.70
11111111111111111111
5.418845.189655.24855.143133.302593.197223.079443.079443.079443.079443.079442.791762.609442.609442.098612.386291.69315000
8366706310988888655342000
132919
species

9.709.709.709.709.709.709.709.709.709.709.709.709.709.709.709.709.709.709.709.70
11111111111111111111
5.418845.189655.24855.143133.302593.197223.079443.079443.079443.079443.079442.791762.609442.609442.098612.386291.69315000
8366706310988888655342000
101510

Rhodococcus\_jostii\_RHA1
Rhodococcus\_jostii\_RHA1
Rhodococcus\_jostii\_RHA1
Rhodococcus\_jostii\_RHA1
Rhodococcus\_jostii\_RHA1
Rhodococcus\_jostii\_RHA1
Rhodococcus\_jostii\_RHA1
Rhodococcus\_jostii\_RHA1
Rhodococcus\_jostii\_RHA1
Rhodococcus\_jostii\_RHA1
Rhodococcus\_jostii\_RHA1
Rhodococcus\_jostii\_RHA1
Rhodococcus\_jostii\_RHA1
Rhodococcus\_jostii\_RHA1
Rhodococcus\_jostii\_RHA1
Rhodococcus\_jostii\_RHA1
Rhodococcus\_jostii\_RHA1
Rhodococcus\_jostii\_RHA1
Rhodococcus\_jostii\_RHA1
Rhodococcus\_jostii\_RHA1
no rank

8.838.838.838.838.838.838.838.838.838.838.838.838.838.838.838.838.838.838.838.83
11111111111111111111
4.76124.555354.40124.4965111.6931500110000000000
433530331200110000000000
37919
species

8.838.838.838.838.838.838.838.838.838.838.838.838.838.838.838.838.838.838.838.83
11111111111111111111
4.76124.555354.40124.4965111.6931500110000000000
433530331200110000000000
632772

Rhodococcus\_opacus\_B4
Rhodococcus\_opacus\_B4
Rhodococcus\_opacus\_B4
Rhodococcus\_opacus\_B4
Rhodococcus\_opacus\_B4
Rhodococcus\_opacus\_B4
Rhodococcus\_opacus\_B4
Rhodococcus\_opacus\_B4
Rhodococcus\_opacus\_B4
Rhodococcus\_opacus\_B4
Rhodococcus\_opacus\_B4
Rhodococcus\_opacus\_B4
Rhodococcus\_opacus\_B4
Rhodococcus\_opacus\_B4
Rhodococcus\_opacus\_B4
Rhodococcus\_opacus\_B4
Rhodococcus\_opacus\_B4
Rhodococcus\_opacus\_B4
Rhodococcus\_opacus\_B4
Rhodococcus\_opacus\_B4
no rank

5.045.045.045.045.045.045.045.045.045.045.045.045.045.045.045.045.045.045.045.04
11111111111111111111
2.609442.386292.386292.3862911001.6931500000000000
54441100200000000000
43767
species

5.045.045.045.045.045.045.045.045.045.045.045.045.045.045.045.045.045.045.045.04
11111111111111111111
2.609442.386292.386292.3862911001.6931500000000000
54441100200000000000
685727

Rhodococcus\_equi\_103S
Rhodococcus\_equi\_103S
Rhodococcus\_equi\_103S
Rhodococcus\_equi\_103S
Rhodococcus\_equi\_103S
Rhodococcus\_equi\_103S
Rhodococcus\_equi\_103S
Rhodococcus\_equi\_103S
Rhodococcus\_equi\_103S
Rhodococcus\_equi\_103S
Rhodococcus\_equi\_103S
Rhodococcus\_equi\_103S
Rhodococcus\_equi\_103S
Rhodococcus\_equi\_103S
Rhodococcus\_equi\_103S
Rhodococcus\_equi\_103S
Rhodococcus\_equi\_103S
Rhodococcus\_equi\_103S
Rhodococcus\_equi\_103S
Rhodococcus\_equi\_103S
no rank

252.81252.81252.81252.81252.81252.81252.81252.81252.81252.81252.81252.81252.81252.81252.81252.81252.81252.81252.81252.81
5050505050505050505050505050505050505050
3.678543.221322.96822.831211.151141.066431.074951.051241.11851.082171.042411.033820.8636420.7482090.7105780.6938660.5705190.5265920.5457570.545757
159761111111111111111
1762
family

247.95247.95247.95247.95247.95247.95247.95247.95247.95247.95247.95247.95247.95247.95247.95247.95247.95247.95247.95247.95
4949494949494949494949494949494949494949
3.687973.221792.971662.835551.17371.087331.096021.071841.140421.103381.062841.054080.880570.7628750.7245050.7074670.5817010.5369140.5564540.556454
159761111111111111111
1763
genus

6.266.266.266.266.266.266.266.266.266.266.266.266.266.266.266.266.266.266.266.26
11111111111111111111
4.637594.33224.40124.33223.079442.945913.079442.791763.079442.945912.609442.609442.791762.0986111.693151000
382830288786875563121000
189918

Mycobacterium\_sp.\_KMS
Mycobacterium\_sp.\_KMS
Mycobacterium\_sp.\_KMS
Mycobacterium\_sp.\_KMS
Mycobacterium\_sp.\_KMS
Mycobacterium\_sp.\_KMS
Mycobacterium\_sp.\_KMS
Mycobacterium\_sp.\_KMS
Mycobacterium\_sp.\_KMS
Mycobacterium\_sp.\_KMS
Mycobacterium\_sp.\_KMS
Mycobacterium\_sp.\_KMS
Mycobacterium\_sp.\_KMS
Mycobacterium\_sp.\_KMS
Mycobacterium\_sp.\_KMS
Mycobacterium\_sp.\_KMS
Mycobacterium\_sp.\_KMS
Mycobacterium\_sp.\_KMS
Mycobacterium\_sp.\_KMS
Mycobacterium\_sp.\_KMS
species

11.7611.7611.7611.7611.7611.7611.7611.7611.7611.7611.7611.7611.7611.7611.7611.7611.7611.7611.7611.76
22222222222222222222
4.836874.554734.419624.281342.25181.419621.419621.419621.419621.326911.326911.419620.8609710.8609710.8609710.8609710000
463531273222211211110000
1804
species

5.985.985.985.985.985.985.985.985.985.985.985.985.985.985.985.985.985.985.985.98
11111111111111111111
4.637594.40124.135494.044522.791762.791762.791762.791762.791762.609442.609442.791761.693151.693151.693151.693150000
383023216666655622220000
350054

Mycobacterium\_gilvum\_PYR-GCK
Mycobacterium\_gilvum\_PYR-GCK
Mycobacterium\_gilvum\_PYR-GCK
Mycobacterium\_gilvum\_PYR-GCK
Mycobacterium\_gilvum\_PYR-GCK
Mycobacterium\_gilvum\_PYR-GCK
Mycobacterium\_gilvum\_PYR-GCK
Mycobacterium\_gilvum\_PYR-GCK
Mycobacterium\_gilvum\_PYR-GCK
Mycobacterium\_gilvum\_PYR-GCK
Mycobacterium\_gilvum\_PYR-GCK
Mycobacterium\_gilvum\_PYR-GCK
Mycobacterium\_gilvum\_PYR-GCK
Mycobacterium\_gilvum\_PYR-GCK
Mycobacterium\_gilvum\_PYR-GCK
Mycobacterium\_gilvum\_PYR-GCK
Mycobacterium\_gilvum\_PYR-GCK
Mycobacterium\_gilvum\_PYR-GCK
Mycobacterium\_gilvum\_PYR-GCK
Mycobacterium\_gilvum\_PYR-GCK
no rank

5.785.785.785.785.785.785.785.785.785.785.785.785.785.785.785.785.785.785.785.78
11111111111111111111
5.043054.713574.713574.526361.69315000000000000000
574141342000000000000000
278137

Mycobacterium\_gilvum\_Spyr1
Mycobacterium\_gilvum\_Spyr1
Mycobacterium\_gilvum\_Spyr1
Mycobacterium\_gilvum\_Spyr1
Mycobacterium\_gilvum\_Spyr1
Mycobacterium\_gilvum\_Spyr1
Mycobacterium\_gilvum\_Spyr1
Mycobacterium\_gilvum\_Spyr1
Mycobacterium\_gilvum\_Spyr1
Mycobacterium\_gilvum\_Spyr1
Mycobacterium\_gilvum\_Spyr1
Mycobacterium\_gilvum\_Spyr1
Mycobacterium\_gilvum\_Spyr1
Mycobacterium\_gilvum\_Spyr1
Mycobacterium\_gilvum\_Spyr1
Mycobacterium\_gilvum\_Spyr1
Mycobacterium\_gilvum\_Spyr1
Mycobacterium\_gilvum\_Spyr1
Mycobacterium\_gilvum\_Spyr1
Mycobacterium\_gilvum\_Spyr1
no rank

105.88105.88105.88105.88105.88105.88105.88105.88105.88105.88105.88105.88105.88105.88105.88105.88105.88105.88105.88105.88
2424242424242424242424242424242424242424
3.46732.74022.304342.128871.024451.029371.024451.024451.024451.024451.024451.024450.9958410.9387560.9673640.9672330.9387560.9387560.9387560.938756
126431111111111111111
77643
species group

17.4417.4417.4417.4417.4417.4417.4417.4417.4417.4417.4417.4417.4417.4417.4417.4417.4417.4417.4417.44
44444444444444444444
2.902472.17071.850991.427881.270831.270831.270831.270831.270831.270831.270831.270831.097150.7505730.9242580.9234630.7505730.7505730.7505730.750573
73221111111111111111
1765
species

13.0913.0913.0913.0913.0913.0913.0913.0913.0913.0913.0913.0913.0913.0913.0913.0913.0913.0913.0913.09
33333333333333333333
3.0742.194652.133791.902381.693151.693151.693151.693151.693151.693151.693151.693151.4617511.23141.230341111
83322222222221111111
33892
no rank

4.374.374.374.374.374.374.374.374.374.374.374.374.374.374.374.374.374.374.374.37
11111111111111111111
2.945912.791762.609442.609442.386292.386292.386292.386292.386292.386292.386292.386291.6931511.6931511111
76554444444421211111
410289

Mycobacterium\_bovis\_BCG\_str.\_Pasteur\_1173P2
Mycobacterium\_bovis\_BCG\_str.\_Pasteur\_1173P2
Mycobacterium\_bovis\_BCG\_str.\_Pasteur\_1173P2
Mycobacterium\_bovis\_BCG\_str.\_Pasteur\_1173P2
Mycobacterium\_bovis\_BCG\_str.\_Pasteur\_1173P2
Mycobacterium\_bovis\_BCG\_str.\_Pasteur\_1173P2
Mycobacterium\_bovis\_BCG\_str.\_Pasteur\_1173P2
Mycobacterium\_bovis\_BCG\_str.\_Pasteur\_1173P2
Mycobacterium\_bovis\_BCG\_str.\_Pasteur\_1173P2
Mycobacterium\_bovis\_BCG\_str.\_Pasteur\_1173P2
Mycobacterium\_bovis\_BCG\_str.\_Pasteur\_1173P2
Mycobacterium\_bovis\_BCG\_str.\_Pasteur\_1173P2
Mycobacterium\_bovis\_BCG\_str.\_Pasteur\_1173P2
Mycobacterium\_bovis\_BCG\_str.\_Pasteur\_1173P2
Mycobacterium\_bovis\_BCG\_str.\_Pasteur\_1173P2
Mycobacterium\_bovis\_BCG\_str.\_Pasteur\_1173P2
Mycobacterium\_bovis\_BCG\_str.\_Pasteur\_1173P2
Mycobacterium\_bovis\_BCG\_str.\_Pasteur\_1173P2
Mycobacterium\_bovis\_BCG\_str.\_Pasteur\_1173P2
Mycobacterium\_bovis\_BCG\_str.\_Pasteur\_1173P2
no rank

4.374.374.374.374.374.374.374.374.374.374.374.374.374.374.374.374.374.374.374.37
11111111111111111111
3.079441.693151.6931511111111111111111
82211111111111111111
561275

Mycobacterium\_bovis\_BCG\_str.\_Tokyo\_172
Mycobacterium\_bovis\_BCG\_str.\_Tokyo\_172
Mycobacterium\_bovis\_BCG\_str.\_Tokyo\_172
Mycobacterium\_bovis\_BCG\_str.\_Tokyo\_172
Mycobacterium\_bovis\_BCG\_str.\_Tokyo\_172
Mycobacterium\_bovis\_BCG\_str.\_Tokyo\_172
Mycobacterium\_bovis\_BCG\_str.\_Tokyo\_172
Mycobacterium\_bovis\_BCG\_str.\_Tokyo\_172
Mycobacterium\_bovis\_BCG\_str.\_Tokyo\_172
Mycobacterium\_bovis\_BCG\_str.\_Tokyo\_172
Mycobacterium\_bovis\_BCG\_str.\_Tokyo\_172
Mycobacterium\_bovis\_BCG\_str.\_Tokyo\_172
Mycobacterium\_bovis\_BCG\_str.\_Tokyo\_172
Mycobacterium\_bovis\_BCG\_str.\_Tokyo\_172
Mycobacterium\_bovis\_BCG\_str.\_Tokyo\_172
Mycobacterium\_bovis\_BCG\_str.\_Tokyo\_172
Mycobacterium\_bovis\_BCG\_str.\_Tokyo\_172
Mycobacterium\_bovis\_BCG\_str.\_Tokyo\_172
Mycobacterium\_bovis\_BCG\_str.\_Tokyo\_172
Mycobacterium\_bovis\_BCG\_str.\_Tokyo\_172
no rank

4.354.354.354.354.354.354.354.354.354.354.354.354.354.354.354.354.354.354.354.35
11111111111111111111
3.197222.098612.098612.098611.693151.693151.693151.693151.693151.693151.693151.693151.69315111.693151111
93332222222221121111
717522

Mycobacterium\_bovis\_BCG\_str.\_Mexico
Mycobacterium\_bovis\_BCG\_str.\_Mexico
Mycobacterium\_bovis\_BCG\_str.\_Mexico
Mycobacterium\_bovis\_BCG\_str.\_Mexico
Mycobacterium\_bovis\_BCG\_str.\_Mexico
Mycobacterium\_bovis\_BCG\_str.\_Mexico
Mycobacterium\_bovis\_BCG\_str.\_Mexico
Mycobacterium\_bovis\_BCG\_str.\_Mexico
Mycobacterium\_bovis\_BCG\_str.\_Mexico
Mycobacterium\_bovis\_BCG\_str.\_Mexico
Mycobacterium\_bovis\_BCG\_str.\_Mexico
Mycobacterium\_bovis\_BCG\_str.\_Mexico
Mycobacterium\_bovis\_BCG\_str.\_Mexico
Mycobacterium\_bovis\_BCG\_str.\_Mexico
Mycobacterium\_bovis\_BCG\_str.\_Mexico
Mycobacterium\_bovis\_BCG\_str.\_Mexico
Mycobacterium\_bovis\_BCG\_str.\_Mexico
Mycobacterium\_bovis\_BCG\_str.\_Mexico
Mycobacterium\_bovis\_BCG\_str.\_Mexico
Mycobacterium\_bovis\_BCG\_str.\_Mexico
no rank

4.354.354.354.354.354.354.354.354.354.354.354.354.354.354.354.354.354.354.354.35
11111111111111111111
2.386292.09861100000000000000000
43100000000000000000
233413

Mycobacterium\_bovis\_AF2122\_97
Mycobacterium\_bovis\_AF2122\_97
Mycobacterium\_bovis\_AF2122\_97
Mycobacterium\_bovis\_AF2122\_97
Mycobacterium\_bovis\_AF2122\_97
Mycobacterium\_bovis\_AF2122\_97
Mycobacterium\_bovis\_AF2122\_97
Mycobacterium\_bovis\_AF2122\_97
Mycobacterium\_bovis\_AF2122\_97
Mycobacterium\_bovis\_AF2122\_97
Mycobacterium\_bovis\_AF2122\_97
Mycobacterium\_bovis\_AF2122\_97
Mycobacterium\_bovis\_AF2122\_97
Mycobacterium\_bovis\_AF2122\_97
Mycobacterium\_bovis\_AF2122\_97
Mycobacterium\_bovis\_AF2122\_97
Mycobacterium\_bovis\_AF2122\_97
Mycobacterium\_bovis\_AF2122\_97
Mycobacterium\_bovis\_AF2122\_97
Mycobacterium\_bovis\_AF2122\_97
no rank

61.6761.6761.6761.6761.6761.6761.6761.6761.6761.6761.6761.6761.6761.6761.6761.6761.6761.6761.6761.67
1414141414141414141414141414141414141414
3.252062.461991.890351.742340.4237710.4237710.4237710.4237710.4237710.4237710.4237710.4237710.4237710.4237710.4237710.4237710.4237710.4237710.4237710.423771
104221111111111111111
1773
species

4.424.424.424.424.424.424.424.424.424.424.424.424.424.424.424.424.424.424.424.42
11111111111111111111
3.639062.791761.693152.386290000000000000000
146240000000000000000
336982

Mycobacterium\_tuberculosis\_F11
Mycobacterium\_tuberculosis\_F11
Mycobacterium\_tuberculosis\_F11
Mycobacterium\_tuberculosis\_F11
Mycobacterium\_tuberculosis\_F11
Mycobacterium\_tuberculosis\_F11
Mycobacterium\_tuberculosis\_F11
Mycobacterium\_tuberculosis\_F11
Mycobacterium\_tuberculosis\_F11
Mycobacterium\_tuberculosis\_F11
Mycobacterium\_tuberculosis\_F11
Mycobacterium\_tuberculosis\_F11
Mycobacterium\_tuberculosis\_F11
Mycobacterium\_tuberculosis\_F11
Mycobacterium\_tuberculosis\_F11
Mycobacterium\_tuberculosis\_F11
Mycobacterium\_tuberculosis\_F11
Mycobacterium\_tuberculosis\_F11
Mycobacterium\_tuberculosis\_F11
Mycobacterium\_tuberculosis\_F11
no rank

4.414.414.414.414.414.414.414.414.414.414.414.414.414.414.414.414.414.414.414.41
11111111111111111111
3.302592.098612.386292.386290000000000000000
103440000000000000000
1091501

Mycobacterium\_tuberculosis\_RGTB423
Mycobacterium\_tuberculosis\_RGTB423
Mycobacterium\_tuberculosis\_RGTB423
Mycobacterium\_tuberculosis\_RGTB423
Mycobacterium\_tuberculosis\_RGTB423
Mycobacterium\_tuberculosis\_RGTB423
Mycobacterium\_tuberculosis\_RGTB423
Mycobacterium\_tuberculosis\_RGTB423
Mycobacterium\_tuberculosis\_RGTB423
Mycobacterium\_tuberculosis\_RGTB423
Mycobacterium\_tuberculosis\_RGTB423
Mycobacterium\_tuberculosis\_RGTB423
Mycobacterium\_tuberculosis\_RGTB423
Mycobacterium\_tuberculosis\_RGTB423
Mycobacterium\_tuberculosis\_RGTB423
Mycobacterium\_tuberculosis\_RGTB423
Mycobacterium\_tuberculosis\_RGTB423
Mycobacterium\_tuberculosis\_RGTB423
Mycobacterium\_tuberculosis\_RGTB423
Mycobacterium\_tuberculosis\_RGTB423
no rank

4.384.384.384.384.384.384.384.384.384.384.384.384.384.384.384.384.384.384.384.38
11111111111111111111
3.079442.609442.386292.098610000000000000000
85430000000000000000
1091500

Mycobacterium\_tuberculosis\_RGTB327
Mycobacterium\_tuberculosis\_RGTB327
Mycobacterium\_tuberculosis\_RGTB327
Mycobacterium\_tuberculosis\_RGTB327
Mycobacterium\_tuberculosis\_RGTB327
Mycobacterium\_tuberculosis\_RGTB327
Mycobacterium\_tuberculosis\_RGTB327
Mycobacterium\_tuberculosis\_RGTB327
Mycobacterium\_tuberculosis\_RGTB327
Mycobacterium\_tuberculosis\_RGTB327
Mycobacterium\_tuberculosis\_RGTB327
Mycobacterium\_tuberculosis\_RGTB327
Mycobacterium\_tuberculosis\_RGTB327
Mycobacterium\_tuberculosis\_RGTB327
Mycobacterium\_tuberculosis\_RGTB327
Mycobacterium\_tuberculosis\_RGTB327
Mycobacterium\_tuberculosis\_RGTB327
Mycobacterium\_tuberculosis\_RGTB327
Mycobacterium\_tuberculosis\_RGTB327
Mycobacterium\_tuberculosis\_RGTB327
no rank

4.404.404.404.404.404.404.404.404.404.404.404.404.404.404.404.404.404.404.404.40
11111111111111111111
2.386292.09861000000000000000000
43000000000000000000
83331

Mycobacterium\_tuberculosis\_CDC1551
Mycobacterium\_tuberculosis\_CDC1551
Mycobacterium\_tuberculosis\_CDC1551
Mycobacterium\_tuberculosis\_CDC1551
Mycobacterium\_tuberculosis\_CDC1551
Mycobacterium\_tuberculosis\_CDC1551
Mycobacterium\_tuberculosis\_CDC1551
Mycobacterium\_tuberculosis\_CDC1551
Mycobacterium\_tuberculosis\_CDC1551
Mycobacterium\_tuberculosis\_CDC1551
Mycobacterium\_tuberculosis\_CDC1551
Mycobacterium\_tuberculosis\_CDC1551
Mycobacterium\_tuberculosis\_CDC1551
Mycobacterium\_tuberculosis\_CDC1551
Mycobacterium\_tuberculosis\_CDC1551
Mycobacterium\_tuberculosis\_CDC1551
Mycobacterium\_tuberculosis\_CDC1551
Mycobacterium\_tuberculosis\_CDC1551
Mycobacterium\_tuberculosis\_CDC1551
Mycobacterium\_tuberculosis\_CDC1551
no rank

4.404.404.404.404.404.404.404.404.404.404.404.404.404.404.404.404.404.404.404.40
11111111111111111111
2.945912.38629110000000000000000
74110000000000000000
478434

Mycobacterium\_tuberculosis\_KZN\_1435
Mycobacterium\_tuberculosis\_KZN\_1435
Mycobacterium\_tuberculosis\_KZN\_1435
Mycobacterium\_tuberculosis\_KZN\_1435
Mycobacterium\_tuberculosis\_KZN\_1435
Mycobacterium\_tuberculosis\_KZN\_1435
Mycobacterium\_tuberculosis\_KZN\_1435
Mycobacterium\_tuberculosis\_KZN\_1435
Mycobacterium\_tuberculosis\_KZN\_1435
Mycobacterium\_tuberculosis\_KZN\_1435
Mycobacterium\_tuberculosis\_KZN\_1435
Mycobacterium\_tuberculosis\_KZN\_1435
Mycobacterium\_tuberculosis\_KZN\_1435
Mycobacterium\_tuberculosis\_KZN\_1435
Mycobacterium\_tuberculosis\_KZN\_1435
Mycobacterium\_tuberculosis\_KZN\_1435
Mycobacterium\_tuberculosis\_KZN\_1435
Mycobacterium\_tuberculosis\_KZN\_1435
Mycobacterium\_tuberculosis\_KZN\_1435
Mycobacterium\_tuberculosis\_KZN\_1435
no rank

4.404.404.404.404.404.404.404.404.404.404.404.404.404.404.404.404.404.404.404.40
11111111111111111111
2.7917611.6931510000000000000000
61210000000000000000
478435

Mycobacterium\_tuberculosis\_KZN\_605
Mycobacterium\_tuberculosis\_KZN\_605
Mycobacterium\_tuberculosis\_KZN\_605
Mycobacterium\_tuberculosis\_KZN\_605
Mycobacterium\_tuberculosis\_KZN\_605
Mycobacterium\_tuberculosis\_KZN\_605
Mycobacterium\_tuberculosis\_KZN\_605
Mycobacterium\_tuberculosis\_KZN\_605
Mycobacterium\_tuberculosis\_KZN\_605
Mycobacterium\_tuberculosis\_KZN\_605
Mycobacterium\_tuberculosis\_KZN\_605
Mycobacterium\_tuberculosis\_KZN\_605
Mycobacterium\_tuberculosis\_KZN\_605
Mycobacterium\_tuberculosis\_KZN\_605
Mycobacterium\_tuberculosis\_KZN\_605
Mycobacterium\_tuberculosis\_KZN\_605
Mycobacterium\_tuberculosis\_KZN\_605
Mycobacterium\_tuberculosis\_KZN\_605
Mycobacterium\_tuberculosis\_KZN\_605
Mycobacterium\_tuberculosis\_KZN\_605
no rank

4.424.424.424.424.424.424.424.424.424.424.424.424.424.424.424.424.424.424.424.42
11111111111111111111
5.912655.912655.912655.912655.912655.912655.912655.912655.912655.912655.912655.912655.912655.912655.912655.912655.912655.912655.912655.91265
136136136136136136136136136136136136136136136136136136136136
1097669

Mycobacterium\_tuberculosis\_UT205
Mycobacterium\_tuberculosis\_UT205
Mycobacterium\_tuberculosis\_UT205
Mycobacterium\_tuberculosis\_UT205
Mycobacterium\_tuberculosis\_UT205
Mycobacterium\_tuberculosis\_UT205
Mycobacterium\_tuberculosis\_UT205
Mycobacterium\_tuberculosis\_UT205
Mycobacterium\_tuberculosis\_UT205
Mycobacterium\_tuberculosis\_UT205
Mycobacterium\_tuberculosis\_UT205
Mycobacterium\_tuberculosis\_UT205
Mycobacterium\_tuberculosis\_UT205
Mycobacterium\_tuberculosis\_UT205
Mycobacterium\_tuberculosis\_UT205
Mycobacterium\_tuberculosis\_UT205
Mycobacterium\_tuberculosis\_UT205
Mycobacterium\_tuberculosis\_UT205
Mycobacterium\_tuberculosis\_UT205
Mycobacterium\_tuberculosis\_UT205
no rank

4.394.394.394.394.394.394.394.394.394.394.394.394.394.394.394.394.394.394.394.39
11111111111111111111
3.079441.693151.6931500000000000000000
82200000000000000000
478433

Mycobacterium\_tuberculosis\_KZN\_4207
Mycobacterium\_tuberculosis\_KZN\_4207
Mycobacterium\_tuberculosis\_KZN\_4207
Mycobacterium\_tuberculosis\_KZN\_4207
Mycobacterium\_tuberculosis\_KZN\_4207
Mycobacterium\_tuberculosis\_KZN\_4207
Mycobacterium\_tuberculosis\_KZN\_4207
Mycobacterium\_tuberculosis\_KZN\_4207
Mycobacterium\_tuberculosis\_KZN\_4207
Mycobacterium\_tuberculosis\_KZN\_4207
Mycobacterium\_tuberculosis\_KZN\_4207
Mycobacterium\_tuberculosis\_KZN\_4207
Mycobacterium\_tuberculosis\_KZN\_4207
Mycobacterium\_tuberculosis\_KZN\_4207
Mycobacterium\_tuberculosis\_KZN\_4207
Mycobacterium\_tuberculosis\_KZN\_4207
Mycobacterium\_tuberculosis\_KZN\_4207
Mycobacterium\_tuberculosis\_KZN\_4207
Mycobacterium\_tuberculosis\_KZN\_4207
Mycobacterium\_tuberculosis\_KZN\_4207
no rank

4.404.404.404.404.404.404.404.404.404.404.404.404.404.404.404.404.404.404.404.40
11111111111111111111
3.39792.0986101.693150000000000000000
113020000000000000000
707235

Mycobacterium\_tuberculosis\_CTRI-2
Mycobacterium\_tuberculosis\_CTRI-2
Mycobacterium\_tuberculosis\_CTRI-2
Mycobacterium\_tuberculosis\_CTRI-2
Mycobacterium\_tuberculosis\_CTRI-2
Mycobacterium\_tuberculosis\_CTRI-2
Mycobacterium\_tuberculosis\_CTRI-2
Mycobacterium\_tuberculosis\_CTRI-2
Mycobacterium\_tuberculosis\_CTRI-2
Mycobacterium\_tuberculosis\_CTRI-2
Mycobacterium\_tuberculosis\_CTRI-2
Mycobacterium\_tuberculosis\_CTRI-2
Mycobacterium\_tuberculosis\_CTRI-2
Mycobacterium\_tuberculosis\_CTRI-2
Mycobacterium\_tuberculosis\_CTRI-2
Mycobacterium\_tuberculosis\_CTRI-2
Mycobacterium\_tuberculosis\_CTRI-2
Mycobacterium\_tuberculosis\_CTRI-2
Mycobacterium\_tuberculosis\_CTRI-2
Mycobacterium\_tuberculosis\_CTRI-2
no rank

4.424.424.424.424.424.424.424.424.424.424.424.424.424.424.424.424.424.424.424.42
11111111111111111111
3.302592.791762.098611.693150000000000000000
106320000000000000000
419947

Mycobacterium\_tuberculosis\_H37Ra
Mycobacterium\_tuberculosis\_H37Ra
Mycobacterium\_tuberculosis\_H37Ra
Mycobacterium\_tuberculosis\_H37Ra
Mycobacterium\_tuberculosis\_H37Ra
Mycobacterium\_tuberculosis\_H37Ra
Mycobacterium\_tuberculosis\_H37Ra
Mycobacterium\_tuberculosis\_H37Ra
Mycobacterium\_tuberculosis\_H37Ra
Mycobacterium\_tuberculosis\_H37Ra
Mycobacterium\_tuberculosis\_H37Ra
Mycobacterium\_tuberculosis\_H37Ra
Mycobacterium\_tuberculosis\_H37Ra
Mycobacterium\_tuberculosis\_H37Ra
Mycobacterium\_tuberculosis\_H37Ra
Mycobacterium\_tuberculosis\_H37Ra
Mycobacterium\_tuberculosis\_H37Ra
Mycobacterium\_tuberculosis\_H37Ra
Mycobacterium\_tuberculosis\_H37Ra
Mycobacterium\_tuberculosis\_H37Ra
no rank

8.828.828.828.828.828.828.828.828.828.828.828.828.828.828.828.828.828.828.828.82
22222222222222222222
3.047172.445192.098612.098610000000000000000
84330000000000000000
83332

Mycobacterium\_tuberculosis\_H37RvMycobacterium\_tuberculosis\_H37Rv
Mycobacterium\_tuberculosis\_H37RvMycobacterium\_tuberculosis\_H37Rv
Mycobacterium\_tuberculosis\_H37RvMycobacterium\_tuberculosis\_H37Rv
Mycobacterium\_tuberculosis\_H37RvMycobacterium\_tuberculosis\_H37Rv
Mycobacterium\_tuberculosis\_H37RvMycobacterium\_tuberculosis\_H37Rv
Mycobacterium\_tuberculosis\_H37RvMycobacterium\_tuberculosis\_H37Rv
Mycobacterium\_tuberculosis\_H37RvMycobacterium\_tuberculosis\_H37Rv
Mycobacterium\_tuberculosis\_H37RvMycobacterium\_tuberculosis\_H37Rv
Mycobacterium\_tuberculosis\_H37RvMycobacterium\_tuberculosis\_H37Rv
Mycobacterium\_tuberculosis\_H37RvMycobacterium\_tuberculosis\_H37Rv
Mycobacterium\_tuberculosis\_H37RvMycobacterium\_tuberculosis\_H37Rv
Mycobacterium\_tuberculosis\_H37RvMycobacterium\_tuberculosis\_H37Rv
Mycobacterium\_tuberculosis\_H37RvMycobacterium\_tuberculosis\_H37Rv
Mycobacterium\_tuberculosis\_H37RvMycobacterium\_tuberculosis\_H37Rv
Mycobacterium\_tuberculosis\_H37RvMycobacterium\_tuberculosis\_H37Rv
Mycobacterium\_tuberculosis\_H37RvMycobacterium\_tuberculosis\_H37Rv
Mycobacterium\_tuberculosis\_H37RvMycobacterium\_tuberculosis\_H37Rv
Mycobacterium\_tuberculosis\_H37RvMycobacterium\_tuberculosis\_H37Rv
Mycobacterium\_tuberculosis\_H37RvMycobacterium\_tuberculosis\_H37Rv
Mycobacterium\_tuberculosis\_H37RvMycobacterium\_tuberculosis\_H37Rv
no rank

4.404.404.404.404.404.404.404.404.404.404.404.404.404.404.404.404.404.404.404.40
11111111111111111111
2.791762.386291.6931510000000000000000
64210000000000000000
443149

Mycobacterium\_tuberculosis\_CCDC5079
Mycobacterium\_tuberculosis\_CCDC5079
Mycobacterium\_tuberculosis\_CCDC5079
Mycobacterium\_tuberculosis\_CCDC5079
Mycobacterium\_tuberculosis\_CCDC5079
Mycobacterium\_tuberculosis\_CCDC5079
Mycobacterium\_tuberculosis\_CCDC5079
Mycobacterium\_tuberculosis\_CCDC5079
Mycobacterium\_tuberculosis\_CCDC5079
Mycobacterium\_tuberculosis\_CCDC5079
Mycobacterium\_tuberculosis\_CCDC5079
Mycobacterium\_tuberculosis\_CCDC5079
Mycobacterium\_tuberculosis\_CCDC5079
Mycobacterium\_tuberculosis\_CCDC5079
Mycobacterium\_tuberculosis\_CCDC5079
Mycobacterium\_tuberculosis\_CCDC5079
Mycobacterium\_tuberculosis\_CCDC5079
Mycobacterium\_tuberculosis\_CCDC5079
Mycobacterium\_tuberculosis\_CCDC5079
Mycobacterium\_tuberculosis\_CCDC5079
no rank

4.414.414.414.414.414.414.414.414.414.414.414.414.414.414.414.414.414.414.414.41
11111111111111111111
2.791761.693151.6931510000000000000000
62210000000000000000
443150

Mycobacterium\_tuberculosis\_CCDC5180
Mycobacterium\_tuberculosis\_CCDC5180
Mycobacterium\_tuberculosis\_CCDC5180
Mycobacterium\_tuberculosis\_CCDC5180
Mycobacterium\_tuberculosis\_CCDC5180
Mycobacterium\_tuberculosis\_CCDC5180
Mycobacterium\_tuberculosis\_CCDC5180
Mycobacterium\_tuberculosis\_CCDC5180
Mycobacterium\_tuberculosis\_CCDC5180
Mycobacterium\_tuberculosis\_CCDC5180
Mycobacterium\_tuberculosis\_CCDC5180
Mycobacterium\_tuberculosis\_CCDC5180
Mycobacterium\_tuberculosis\_CCDC5180
Mycobacterium\_tuberculosis\_CCDC5180
Mycobacterium\_tuberculosis\_CCDC5180
Mycobacterium\_tuberculosis\_CCDC5180
Mycobacterium\_tuberculosis\_CCDC5180
Mycobacterium\_tuberculosis\_CCDC5180
Mycobacterium\_tuberculosis\_CCDC5180
Mycobacterium\_tuberculosis\_CCDC5180
no rank

4.394.394.394.394.394.394.394.394.394.394.394.394.394.394.394.394.394.394.394.39
11111111111111111111
2.945911000000000000000000
71000000000000000000
33894
species

4.394.394.394.394.394.394.394.394.394.394.394.394.394.394.394.394.394.394.394.39
11111111111111111111
2.945911000000000000000000
71000000000000000000
572418

Mycobacterium\_africanum\_GM041182
Mycobacterium\_africanum\_GM041182
Mycobacterium\_africanum\_GM041182
Mycobacterium\_africanum\_GM041182
Mycobacterium\_africanum\_GM041182
Mycobacterium\_africanum\_GM041182
Mycobacterium\_africanum\_GM041182
Mycobacterium\_africanum\_GM041182
Mycobacterium\_africanum\_GM041182
Mycobacterium\_africanum\_GM041182
Mycobacterium\_africanum\_GM041182
Mycobacterium\_africanum\_GM041182
Mycobacterium\_africanum\_GM041182
Mycobacterium\_africanum\_GM041182
Mycobacterium\_africanum\_GM041182
Mycobacterium\_africanum\_GM041182
Mycobacterium\_africanum\_GM041182
Mycobacterium\_africanum\_GM041182
Mycobacterium\_africanum\_GM041182
Mycobacterium\_africanum\_GM041182
no rank

22.3822.3822.3822.3822.3822.3822.3822.3822.3822.3822.3822.3822.3822.3822.3822.3822.3822.3822.3822.38
55555555555555555555
4.602834.292014.25044.157852.688632.711892.688632.688632.688632.688632.688632.688632.688632.688632.688632.688632.688632.688632.688632.68863
372726245655555555555555
78331
species

4.534.534.534.534.534.534.534.534.534.534.534.534.534.534.534.534.534.534.534.53
11111111111111111111
4.951244.850154.784194.76123.564953.564953.564953.564953.564953.564953.564953.564953.564953.564953.564953.564953.564953.564953.564953.56495
5247444313131313131313131313131313131313
1205674

Mycobacterium\_canettii\_CIPT\_140070010
Mycobacterium\_canettii\_CIPT\_140070010
Mycobacterium\_canettii\_CIPT\_140070010
Mycobacterium\_canettii\_CIPT\_140070010
Mycobacterium\_canettii\_CIPT\_140070010
Mycobacterium\_canettii\_CIPT\_140070010
Mycobacterium\_canettii\_CIPT\_140070010
Mycobacterium\_canettii\_CIPT\_140070010
Mycobacterium\_canettii\_CIPT\_140070010
Mycobacterium\_canettii\_CIPT\_140070010
Mycobacterium\_canettii\_CIPT\_140070010
Mycobacterium\_canettii\_CIPT\_140070010
Mycobacterium\_canettii\_CIPT\_140070010
Mycobacterium\_canettii\_CIPT\_140070010
Mycobacterium\_canettii\_CIPT\_140070010
Mycobacterium\_canettii\_CIPT\_140070010
Mycobacterium\_canettii\_CIPT\_140070010
Mycobacterium\_canettii\_CIPT\_140070010
Mycobacterium\_canettii\_CIPT\_140070010
Mycobacterium\_canettii\_CIPT\_140070010
no rank

4.524.524.524.524.524.524.524.524.524.524.524.524.524.524.524.524.524.524.524.52
11111111111111111111
4.637594.33224.135494.044523.39793.39793.39793.39793.39793.39793.39793.39793.39793.39793.39793.39793.39793.39793.39793.3979
3828232111111111111111111111111111111111
1205677

Mycobacterium\_canettii\_CIPT\_140070017
Mycobacterium\_canettii\_CIPT\_140070017
Mycobacterium\_canettii\_CIPT\_140070017
Mycobacterium\_canettii\_CIPT\_140070017
Mycobacterium\_canettii\_CIPT\_140070017
Mycobacterium\_canettii\_CIPT\_140070017
Mycobacterium\_canettii\_CIPT\_140070017
Mycobacterium\_canettii\_CIPT\_140070017
Mycobacterium\_canettii\_CIPT\_140070017
Mycobacterium\_canettii\_CIPT\_140070017
Mycobacterium\_canettii\_CIPT\_140070017
Mycobacterium\_canettii\_CIPT\_140070017
Mycobacterium\_canettii\_CIPT\_140070017
Mycobacterium\_canettii\_CIPT\_140070017
Mycobacterium\_canettii\_CIPT\_140070017
Mycobacterium\_canettii\_CIPT\_140070017
Mycobacterium\_canettii\_CIPT\_140070017
Mycobacterium\_canettii\_CIPT\_140070017
Mycobacterium\_canettii\_CIPT\_140070017
Mycobacterium\_canettii\_CIPT\_140070017
no rank

4.434.434.434.434.434.434.434.434.434.434.434.434.434.434.434.434.434.434.434.43
11111111111111111111
4.555354.178054.25814.178053.39793.39793.39793.39793.39793.39793.39793.39793.39793.39793.39793.39793.39793.39793.39793.3979
3524262411111111111111111111111111111111
1205676

Mycobacterium\_canettii\_CIPT\_140060008
Mycobacterium\_canettii\_CIPT\_140060008
Mycobacterium\_canettii\_CIPT\_140060008
Mycobacterium\_canettii\_CIPT\_140060008
Mycobacterium\_canettii\_CIPT\_140060008
Mycobacterium\_canettii\_CIPT\_140060008
Mycobacterium\_canettii\_CIPT\_140060008
Mycobacterium\_canettii\_CIPT\_140060008
Mycobacterium\_canettii\_CIPT\_140060008
Mycobacterium\_canettii\_CIPT\_140060008
Mycobacterium\_canettii\_CIPT\_140060008
Mycobacterium\_canettii\_CIPT\_140060008
Mycobacterium\_canettii\_CIPT\_140060008
Mycobacterium\_canettii\_CIPT\_140060008
Mycobacterium\_canettii\_CIPT\_140060008
Mycobacterium\_canettii\_CIPT\_140060008
Mycobacterium\_canettii\_CIPT\_140060008
Mycobacterium\_canettii\_CIPT\_140060008
Mycobacterium\_canettii\_CIPT\_140060008
Mycobacterium\_canettii\_CIPT\_140060008
no rank

4.424.424.424.424.424.424.424.424.424.424.424.424.424.424.424.424.424.424.424.42
11111111111111111111
4.496514.25814.178054.091043.079443.197223.079443.079443.079443.079443.079443.079443.079443.079443.079443.079443.079443.079443.079443.07944
332624228988888888888888
1205675

Mycobacterium\_canettii\_CIPT\_140070008
Mycobacterium\_canettii\_CIPT\_140070008
Mycobacterium\_canettii\_CIPT\_140070008
Mycobacterium\_canettii\_CIPT\_140070008
Mycobacterium\_canettii\_CIPT\_140070008
Mycobacterium\_canettii\_CIPT\_140070008
Mycobacterium\_canettii\_CIPT\_140070008
Mycobacterium\_canettii\_CIPT\_140070008
Mycobacterium\_canettii\_CIPT\_140070008
Mycobacterium\_canettii\_CIPT\_140070008
Mycobacterium\_canettii\_CIPT\_140070008
Mycobacterium\_canettii\_CIPT\_140070008
Mycobacterium\_canettii\_CIPT\_140070008
Mycobacterium\_canettii\_CIPT\_140070008
Mycobacterium\_canettii\_CIPT\_140070008
Mycobacterium\_canettii\_CIPT\_140070008
Mycobacterium\_canettii\_CIPT\_140070008
Mycobacterium\_canettii\_CIPT\_140070008
Mycobacterium\_canettii\_CIPT\_140070008
Mycobacterium\_canettii\_CIPT\_140070008
no rank

4.484.484.484.484.484.484.484.484.484.484.484.484.484.484.484.484.484.484.484.48
11111111111111111111
4.36733.833213.890373.708050000000000000000
291718150000000000000000
1048245

Mycobacterium\_canettii\_CIPT\_140010059
Mycobacterium\_canettii\_CIPT\_140010059
Mycobacterium\_canettii\_CIPT\_140010059
Mycobacterium\_canettii\_CIPT\_140010059
Mycobacterium\_canettii\_CIPT\_140010059
Mycobacterium\_canettii\_CIPT\_140010059
Mycobacterium\_canettii\_CIPT\_140010059
Mycobacterium\_canettii\_CIPT\_140010059
Mycobacterium\_canettii\_CIPT\_140010059
Mycobacterium\_canettii\_CIPT\_140010059
Mycobacterium\_canettii\_CIPT\_140010059
Mycobacterium\_canettii\_CIPT\_140010059
Mycobacterium\_canettii\_CIPT\_140010059
Mycobacterium\_canettii\_CIPT\_140010059
Mycobacterium\_canettii\_CIPT\_140010059
Mycobacterium\_canettii\_CIPT\_140010059
Mycobacterium\_canettii\_CIPT\_140010059
Mycobacterium\_canettii\_CIPT\_140010059
Mycobacterium\_canettii\_CIPT\_140010059
Mycobacterium\_canettii\_CIPT\_140010059
no rank

6.496.496.496.496.496.496.496.496.496.496.496.496.496.496.496.496.496.496.496.49
11111111111111111111
4.713574.496514.36734.33221.693151.693151.693151.693152.098611.693151.693151.6931510000000
413329282222322210000000
110539
species

6.496.496.496.496.496.496.496.496.496.496.496.496.496.496.496.496.496.496.496.49
11111111111111111111
4.713574.496514.36734.33221.693151.693151.693151.693152.098611.693151.693151.6931510000000
413329282222322210000000
350058

Mycobacterium\_vanbaalenii\_PYR-1
Mycobacterium\_vanbaalenii\_PYR-1
Mycobacterium\_vanbaalenii\_PYR-1
Mycobacterium\_vanbaalenii\_PYR-1
Mycobacterium\_vanbaalenii\_PYR-1
Mycobacterium\_vanbaalenii\_PYR-1
Mycobacterium\_vanbaalenii\_PYR-1
Mycobacterium\_vanbaalenii\_PYR-1
Mycobacterium\_vanbaalenii\_PYR-1
Mycobacterium\_vanbaalenii\_PYR-1
Mycobacterium\_vanbaalenii\_PYR-1
Mycobacterium\_vanbaalenii\_PYR-1
Mycobacterium\_vanbaalenii\_PYR-1
Mycobacterium\_vanbaalenii\_PYR-1
Mycobacterium\_vanbaalenii\_PYR-1
Mycobacterium\_vanbaalenii\_PYR-1
Mycobacterium\_vanbaalenii\_PYR-1
Mycobacterium\_vanbaalenii\_PYR-1
Mycobacterium\_vanbaalenii\_PYR-1
Mycobacterium\_vanbaalenii\_PYR-1
no rank

10.1610.1610.1610.1610.1610.1610.1610.1610.1610.1610.1610.1610.1610.1610.1610.1610.1610.1610.1610.16
22222222222222222222
0.4990160.4990160.4990160.4990160.4990160.4990160.4990160.4990160.4990160.4990160.4990160.4990160.4990160.4990160.4990160.4990160.4990160.4990160.4990160.499016
11111111111111111111
670516
species group

10.1610.1610.1610.1610.1610.1610.1610.1610.1610.1610.1610.1610.1610.1610.1610.1610.1610.1610.1610.16
22222222222222222222
0.4990160.4990160.4990160.4990160.4990160.4990160.4990160.4990160.4990160.4990160.4990160.4990160.4990160.4990160.4990160.4990160.4990160.4990160.4990160.499016
11111111111111111111
670506
species subgroup

10.1610.1610.1610.1610.1610.1610.1610.1610.1610.1610.1610.1610.1610.1610.1610.1610.1610.1610.1610.16
22222222222222222222
0.4990160.4990160.4990160.4990160.4990160.4990160.4990160.4990160.4990160.4990160.4990160.4990160.4990160.4990160.4990160.4990160.4990160.4990160.4990160.499016
11111111111111111111
36809
species

5.075.075.075.075.075.075.075.075.075.075.075.075.075.075.075.075.075.075.075.07
11111111111111111111
11111111111111111111
11111111111111111111
319705
subspecies

5.075.075.075.075.075.075.075.075.075.075.075.075.075.075.075.075.075.075.075.07
11111111111111111111
11111111111111111111
11111111111111111111
1198627

Mycobacterium\_massiliense\_str.\_GO\_06
Mycobacterium\_massiliense\_str.\_GO\_06
Mycobacterium\_massiliense\_str.\_GO\_06
Mycobacterium\_massiliense\_str.\_GO\_06
Mycobacterium\_massiliense\_str.\_GO\_06
Mycobacterium\_massiliense\_str.\_GO\_06
Mycobacterium\_massiliense\_str.\_GO\_06
Mycobacterium\_massiliense\_str.\_GO\_06
Mycobacterium\_massiliense\_str.\_GO\_06
Mycobacterium\_massiliense\_str.\_GO\_06
Mycobacterium\_massiliense\_str.\_GO\_06
Mycobacterium\_massiliense\_str.\_GO\_06
Mycobacterium\_massiliense\_str.\_GO\_06
Mycobacterium\_massiliense\_str.\_GO\_06
Mycobacterium\_massiliense\_str.\_GO\_06
Mycobacterium\_massiliense\_str.\_GO\_06
Mycobacterium\_massiliense\_str.\_GO\_06
Mycobacterium\_massiliense\_str.\_GO\_06
Mycobacterium\_massiliense\_str.\_GO\_06
Mycobacterium\_massiliense\_str.\_GO\_06
no rank

5.095.095.095.095.095.095.095.095.095.095.095.095.095.095.095.095.095.095.095.09
11111111111111111111
00000000000000000000
00000000000000000000
561007

Mycobacterium\_abscessus\_ATCC\_19977
Mycobacterium\_abscessus\_ATCC\_19977
Mycobacterium\_abscessus\_ATCC\_19977
Mycobacterium\_abscessus\_ATCC\_19977
Mycobacterium\_abscessus\_ATCC\_19977
Mycobacterium\_abscessus\_ATCC\_19977
Mycobacterium\_abscessus\_ATCC\_19977
Mycobacterium\_abscessus\_ATCC\_19977
Mycobacterium\_abscessus\_ATCC\_19977
Mycobacterium\_abscessus\_ATCC\_19977
Mycobacterium\_abscessus\_ATCC\_19977
Mycobacterium\_abscessus\_ATCC\_19977
Mycobacterium\_abscessus\_ATCC\_19977
Mycobacterium\_abscessus\_ATCC\_19977
Mycobacterium\_abscessus\_ATCC\_19977
Mycobacterium\_abscessus\_ATCC\_19977
Mycobacterium\_abscessus\_ATCC\_19977
Mycobacterium\_abscessus\_ATCC\_19977
Mycobacterium\_abscessus\_ATCC\_19977
Mycobacterium\_abscessus\_ATCC\_19977
no rank

6.056.056.056.056.056.056.056.056.056.056.056.056.056.056.056.056.056.056.056.05
11111111111111111111
4.33223.890373.890373.833212.0986111.6931512.0986111100000000
281818173121311100000000
164757

Mycobacterium\_sp.\_JLS
Mycobacterium\_sp.\_JLS
Mycobacterium\_sp.\_JLS
Mycobacterium\_sp.\_JLS
Mycobacterium\_sp.\_JLS
Mycobacterium\_sp.\_JLS
Mycobacterium\_sp.\_JLS
Mycobacterium\_sp.\_JLS
Mycobacterium\_sp.\_JLS
Mycobacterium\_sp.\_JLS
Mycobacterium\_sp.\_JLS
Mycobacterium\_sp.\_JLS
Mycobacterium\_sp.\_JLS
Mycobacterium\_sp.\_JLS
Mycobacterium\_sp.\_JLS
Mycobacterium\_sp.\_JLS
Mycobacterium\_sp.\_JLS
Mycobacterium\_sp.\_JLS
Mycobacterium\_sp.\_JLS
Mycobacterium\_sp.\_JLS
species

32.2132.2132.2132.2132.2132.2132.2132.2132.2132.2132.2132.2132.2132.2132.2132.2132.2132.2132.2132.21
66666666666666666666
3.435193.112052.830152.722140.17354900000.1735490000000000
118660000000000000000
120793
species group

10.3110.3110.3110.3110.3110.3110.3110.3110.3110.3110.3110.3110.3110.3110.3110.3110.3110.3110.3110.31
22222222222222222222
3.94863.292413.050972.830640000000000000000
1910860000000000000000
1764
species

4.834.834.834.834.834.834.834.834.834.834.834.834.834.834.834.834.834.834.834.83
11111111111111111111
3.39792.386292.098611.693150000000000000000
114320000000000000000
1770
subspecies

4.834.834.834.834.834.834.834.834.834.834.834.834.834.834.834.834.834.834.834.83
11111111111111111111
3.39792.386292.098611.693150000000000000000
114320000000000000000
262316

Mycobacterium\_avium\_subsp.\_paratuberculosis\_K-10
Mycobacterium\_avium\_subsp.\_paratuberculosis\_K-10
Mycobacterium\_avium\_subsp.\_paratuberculosis\_K-10
Mycobacterium\_avium\_subsp.\_paratuberculosis\_K-10
Mycobacterium\_avium\_subsp.\_paratuberculosis\_K-10
Mycobacterium\_avium\_subsp.\_paratuberculosis\_K-10
Mycobacterium\_avium\_subsp.\_paratuberculosis\_K-10
Mycobacterium\_avium\_subsp.\_paratuberculosis\_K-10
Mycobacterium\_avium\_subsp.\_paratuberculosis\_K-10
Mycobacterium\_avium\_subsp.\_paratuberculosis\_K-10
Mycobacterium\_avium\_subsp.\_paratuberculosis\_K-10
Mycobacterium\_avium\_subsp.\_paratuberculosis\_K-10
Mycobacterium\_avium\_subsp.\_paratuberculosis\_K-10
Mycobacterium\_avium\_subsp.\_paratuberculosis\_K-10
Mycobacterium\_avium\_subsp.\_paratuberculosis\_K-10
Mycobacterium\_avium\_subsp.\_paratuberculosis\_K-10
Mycobacterium\_avium\_subsp.\_paratuberculosis\_K-10
Mycobacterium\_avium\_subsp.\_paratuberculosis\_K-10
Mycobacterium\_avium\_subsp.\_paratuberculosis\_K-10
Mycobacterium\_avium\_subsp.\_paratuberculosis\_K-10
no rank

5.485.485.485.485.485.485.485.485.485.485.485.485.485.485.485.485.485.485.485.48
11111111111111111111
4.433994.091043.890373.833210000000000000000
312218170000000000000000
243243

Mycobacterium\_avium\_104
Mycobacterium\_avium\_104
Mycobacterium\_avium\_104
Mycobacterium\_avium\_104
Mycobacterium\_avium\_104
Mycobacterium\_avium\_104
Mycobacterium\_avium\_104
Mycobacterium\_avium\_104
Mycobacterium\_avium\_104
Mycobacterium\_avium\_104
Mycobacterium\_avium\_104
Mycobacterium\_avium\_104
Mycobacterium\_avium\_104
Mycobacterium\_avium\_104
Mycobacterium\_avium\_104
Mycobacterium\_avium\_104
Mycobacterium\_avium\_104
Mycobacterium\_avium\_104
Mycobacterium\_avium\_104
Mycobacterium\_avium\_104
no rank

5.595.595.595.595.595.595.595.595.595.595.595.595.595.595.595.595.595.595.595.59
11111111111111111111
3.944443.708053.772593.772591000010000000000
191516161000010000000000
35617
species

5.595.595.595.595.595.595.595.595.595.595.595.595.595.595.595.595.595.595.595.59
11111111111111111111
3.944443.708053.772593.772591000010000000000
191516161000010000000000
1232724

Mycobacterium\_indicus\_pranii\_MTCC\_9506
Mycobacterium\_indicus\_pranii\_MTCC\_9506
Mycobacterium\_indicus\_pranii\_MTCC\_9506
Mycobacterium\_indicus\_pranii\_MTCC\_9506
Mycobacterium\_indicus\_pranii\_MTCC\_9506
Mycobacterium\_indicus\_pranii\_MTCC\_9506
Mycobacterium\_indicus\_pranii\_MTCC\_9506
Mycobacterium\_indicus\_pranii\_MTCC\_9506
Mycobacterium\_indicus\_pranii\_MTCC\_9506
Mycobacterium\_indicus\_pranii\_MTCC\_9506
Mycobacterium\_indicus\_pranii\_MTCC\_9506
Mycobacterium\_indicus\_pranii\_MTCC\_9506
Mycobacterium\_indicus\_pranii\_MTCC\_9506
Mycobacterium\_indicus\_pranii\_MTCC\_9506
Mycobacterium\_indicus\_pranii\_MTCC\_9506
Mycobacterium\_indicus\_pranii\_MTCC\_9506
Mycobacterium\_indicus\_pranii\_MTCC\_9506
Mycobacterium\_indicus\_pranii\_MTCC\_9506
Mycobacterium\_indicus\_pranii\_MTCC\_9506
Mycobacterium\_indicus\_pranii\_MTCC\_9506
no rank

16.3116.3116.3116.3116.3116.3116.3116.3116.3116.3116.3116.3116.3116.3116.3116.3116.3116.3116.3116.31
33333333333333333333
2.936112.793772.367552.293530000000000000000
76440000000000000000
1767
species

5.505.505.505.505.505.505.505.505.505.505.505.505.505.505.505.505.505.505.505.50
11111111111111111111
3.39793.079442.791762.791760000000000000000
118660000000000000000
1138383

Mycobacterium\_intracellulare\_MOTT-64
Mycobacterium\_intracellulare\_MOTT-64
Mycobacterium\_intracellulare\_MOTT-64
Mycobacterium\_intracellulare\_MOTT-64
Mycobacterium\_intracellulare\_MOTT-64
Mycobacterium\_intracellulare\_MOTT-64
Mycobacterium\_intracellulare\_MOTT-64
Mycobacterium\_intracellulare\_MOTT-64
Mycobacterium\_intracellulare\_MOTT-64
Mycobacterium\_intracellulare\_MOTT-64
Mycobacterium\_intracellulare\_MOTT-64
Mycobacterium\_intracellulare\_MOTT-64
Mycobacterium\_intracellulare\_MOTT-64
Mycobacterium\_intracellulare\_MOTT-64
Mycobacterium\_intracellulare\_MOTT-64
Mycobacterium\_intracellulare\_MOTT-64
Mycobacterium\_intracellulare\_MOTT-64
Mycobacterium\_intracellulare\_MOTT-64
Mycobacterium\_intracellulare\_MOTT-64
Mycobacterium\_intracellulare\_MOTT-64
no rank

5.415.415.415.415.415.415.415.415.415.415.415.415.415.415.415.415.415.415.415.41
11111111111111111111
3.302593.197222.609442.386290000000000000000
109540000000000000000
1138382

Mycobacterium\_intracellulare\_MOTT-02
Mycobacterium\_intracellulare\_MOTT-02
Mycobacterium\_intracellulare\_MOTT-02
Mycobacterium\_intracellulare\_MOTT-02
Mycobacterium\_intracellulare\_MOTT-02
Mycobacterium\_intracellulare\_MOTT-02
Mycobacterium\_intracellulare\_MOTT-02
Mycobacterium\_intracellulare\_MOTT-02
Mycobacterium\_intracellulare\_MOTT-02
Mycobacterium\_intracellulare\_MOTT-02
Mycobacterium\_intracellulare\_MOTT-02
Mycobacterium\_intracellulare\_MOTT-02
Mycobacterium\_intracellulare\_MOTT-02
Mycobacterium\_intracellulare\_MOTT-02
Mycobacterium\_intracellulare\_MOTT-02
Mycobacterium\_intracellulare\_MOTT-02
Mycobacterium\_intracellulare\_MOTT-02
Mycobacterium\_intracellulare\_MOTT-02
Mycobacterium\_intracellulare\_MOTT-02
Mycobacterium\_intracellulare\_MOTT-02
no rank

5.405.405.405.405.405.405.405.405.405.405.405.405.405.405.405.405.405.405.405.40
11111111111111111111
2.098612.098611.693151.693150000000000000000
33220000000000000000
487521

Mycobacterium\_intracellulare\_ATCC\_13950
Mycobacterium\_intracellulare\_ATCC\_13950
Mycobacterium\_intracellulare\_ATCC\_13950
Mycobacterium\_intracellulare\_ATCC\_13950
Mycobacterium\_intracellulare\_ATCC\_13950
Mycobacterium\_intracellulare\_ATCC\_13950
Mycobacterium\_intracellulare\_ATCC\_13950
Mycobacterium\_intracellulare\_ATCC\_13950
Mycobacterium\_intracellulare\_ATCC\_13950
Mycobacterium\_intracellulare\_ATCC\_13950
Mycobacterium\_intracellulare\_ATCC\_13950
Mycobacterium\_intracellulare\_ATCC\_13950
Mycobacterium\_intracellulare\_ATCC\_13950
Mycobacterium\_intracellulare\_ATCC\_13950
Mycobacterium\_intracellulare\_ATCC\_13950
Mycobacterium\_intracellulare\_ATCC\_13950
Mycobacterium\_intracellulare\_ATCC\_13950
Mycobacterium\_intracellulare\_ATCC\_13950
Mycobacterium\_intracellulare\_ATCC\_13950
Mycobacterium\_intracellulare\_ATCC\_13950
no rank

6.666.666.666.666.666.666.666.666.666.666.666.666.666.666.666.666.666.666.666.66
11111111111111111111
4.931834.637594.663564.526361.693151.693151.693151.693152.098611.693151.693151.6931510100000
513839342222322210100000
1781
species

6.666.666.666.666.666.666.666.666.666.666.666.666.666.666.666.666.666.666.666.66
11111111111111111111
4.931834.637594.663564.526361.693151.693151.693151.693152.098611.693151.693151.6931510100000
513839342222322210100000
216594

Mycobacterium\_marinum\_M
Mycobacterium\_marinum\_M
Mycobacterium\_marinum\_M
Mycobacterium\_marinum\_M
Mycobacterium\_marinum\_M
Mycobacterium\_marinum\_M
Mycobacterium\_marinum\_M
Mycobacterium\_marinum\_M
Mycobacterium\_marinum\_M
Mycobacterium\_marinum\_M
Mycobacterium\_marinum\_M
Mycobacterium\_marinum\_M
Mycobacterium\_marinum\_M
Mycobacterium\_marinum\_M
Mycobacterium\_marinum\_M
Mycobacterium\_marinum\_M
Mycobacterium\_marinum\_M
Mycobacterium\_marinum\_M
Mycobacterium\_marinum\_M
Mycobacterium\_marinum\_M
no rank

5.925.925.925.925.925.925.925.925.925.925.925.925.925.925.925.925.925.925.925.92
11111111111111111111
3.995733.944443.564953.484911.693151.693151.693151.693151.693151.693151.693151.6931511000000
201913122222222211000000
164756

Mycobacterium\_sp.\_MCS
Mycobacterium\_sp.\_MCS
Mycobacterium\_sp.\_MCS
Mycobacterium\_sp.\_MCS
Mycobacterium\_sp.\_MCS
Mycobacterium\_sp.\_MCS
Mycobacterium\_sp.\_MCS
Mycobacterium\_sp.\_MCS
Mycobacterium\_sp.\_MCS
Mycobacterium\_sp.\_MCS
Mycobacterium\_sp.\_MCS
Mycobacterium\_sp.\_MCS
Mycobacterium\_sp.\_MCS
Mycobacterium\_sp.\_MCS
Mycobacterium\_sp.\_MCS
Mycobacterium\_sp.\_MCS
Mycobacterium\_sp.\_MCS
Mycobacterium\_sp.\_MCS
Mycobacterium\_sp.\_MCS
Mycobacterium\_sp.\_MCS
species

6.426.426.426.426.426.426.426.426.426.426.426.426.426.426.426.426.426.426.426.42
11111111111111111111
4.044523.772593.639063.39792.609442.386292.386292.386292.386292.386292.386292.098610111.693150000
211614115444444301120000
36814
species

6.426.426.426.426.426.426.426.426.426.426.426.426.426.426.426.426.426.426.426.42
11111111111111111111
4.044523.772593.639063.39792.609442.386292.386292.386292.386292.386292.386292.098610111.693150000
211614115444444301120000
710685

Mycobacterium\_rhodesiae\_NBB3
Mycobacterium\_rhodesiae\_NBB3
Mycobacterium\_rhodesiae\_NBB3
Mycobacterium\_rhodesiae\_NBB3
Mycobacterium\_rhodesiae\_NBB3
Mycobacterium\_rhodesiae\_NBB3
Mycobacterium\_rhodesiae\_NBB3
Mycobacterium\_rhodesiae\_NBB3
Mycobacterium\_rhodesiae\_NBB3
Mycobacterium\_rhodesiae\_NBB3
Mycobacterium\_rhodesiae\_NBB3
Mycobacterium\_rhodesiae\_NBB3
Mycobacterium\_rhodesiae\_NBB3
Mycobacterium\_rhodesiae\_NBB3
Mycobacterium\_rhodesiae\_NBB3
Mycobacterium\_rhodesiae\_NBB3
Mycobacterium\_rhodesiae\_NBB3
Mycobacterium\_rhodesiae\_NBB3
Mycobacterium\_rhodesiae\_NBB3
Mycobacterium\_rhodesiae\_NBB3
no rank

5.815.815.815.815.815.815.815.815.815.815.815.815.815.815.815.815.815.815.815.81
11111111111111111111
5.442654.988984.931834.912021.693152.098611.693151.693151.693152.098611.693151.693151.693151.693151.693151.693151.693151.693151.693151.69315
855451502322232222222222
1809
species

5.815.815.815.815.815.815.815.815.815.815.815.815.815.815.815.815.815.815.815.81
11111111111111111111
5.442654.988984.931834.912021.693152.098611.693151.693151.693152.098611.693151.693151.693151.693151.693151.693151.693151.693151.693151.69315
855451502322232222222222
362242

Mycobacterium\_ulcerans\_Agy99
Mycobacterium\_ulcerans\_Agy99
Mycobacterium\_ulcerans\_Agy99
Mycobacterium\_ulcerans\_Agy99
Mycobacterium\_ulcerans\_Agy99
Mycobacterium\_ulcerans\_Agy99
Mycobacterium\_ulcerans\_Agy99
Mycobacterium\_ulcerans\_Agy99
Mycobacterium\_ulcerans\_Agy99
Mycobacterium\_ulcerans\_Agy99
Mycobacterium\_ulcerans\_Agy99
Mycobacterium\_ulcerans\_Agy99
Mycobacterium\_ulcerans\_Agy99
Mycobacterium\_ulcerans\_Agy99
Mycobacterium\_ulcerans\_Agy99
Mycobacterium\_ulcerans\_Agy99
Mycobacterium\_ulcerans\_Agy99
Mycobacterium\_ulcerans\_Agy99
Mycobacterium\_ulcerans\_Agy99
Mycobacterium\_ulcerans\_Agy99
no rank

4.644.644.644.644.644.644.644.644.644.644.644.644.644.644.644.644.644.644.644.64
11111111111111111111
3.079442.945912.791762.609440000100000000000
87650000100000000000
875328

Mycobacterium\_sp.\_JDM601
Mycobacterium\_sp.\_JDM601
Mycobacterium\_sp.\_JDM601
Mycobacterium\_sp.\_JDM601
Mycobacterium\_sp.\_JDM601
Mycobacterium\_sp.\_JDM601
Mycobacterium\_sp.\_JDM601
Mycobacterium\_sp.\_JDM601
Mycobacterium\_sp.\_JDM601
Mycobacterium\_sp.\_JDM601
Mycobacterium\_sp.\_JDM601
Mycobacterium\_sp.\_JDM601
Mycobacterium\_sp.\_JDM601
Mycobacterium\_sp.\_JDM601
Mycobacterium\_sp.\_JDM601
Mycobacterium\_sp.\_JDM601
Mycobacterium\_sp.\_JDM601
Mycobacterium\_sp.\_JDM601
Mycobacterium\_sp.\_JDM601
Mycobacterium\_sp.\_JDM601
species

6.346.346.346.346.346.346.346.346.346.346.346.346.346.346.346.346.346.346.346.34
11111111111111111111
4.737674.25814.33224.091042.609442.609442.609442.609442.386292.609442.609442.386292.098611.69315100000
422628225555455432100000
1800
species

6.346.346.346.346.346.346.346.346.346.346.346.346.346.346.346.346.346.346.346.34
11111111111111111111
4.737674.25814.33224.091042.609442.609442.609442.609442.386292.609442.609442.386292.098611.69315100000
422628225555455432100000
710421

Mycobacterium\_chubuense\_NBB4
Mycobacterium\_chubuense\_NBB4
Mycobacterium\_chubuense\_NBB4
Mycobacterium\_chubuense\_NBB4
Mycobacterium\_chubuense\_NBB4
Mycobacterium\_chubuense\_NBB4
Mycobacterium\_chubuense\_NBB4
Mycobacterium\_chubuense\_NBB4
Mycobacterium\_chubuense\_NBB4
Mycobacterium\_chubuense\_NBB4
Mycobacterium\_chubuense\_NBB4
Mycobacterium\_chubuense\_NBB4
Mycobacterium\_chubuense\_NBB4
Mycobacterium\_chubuense\_NBB4
Mycobacterium\_chubuense\_NBB4
Mycobacterium\_chubuense\_NBB4
Mycobacterium\_chubuense\_NBB4
Mycobacterium\_chubuense\_NBB4
Mycobacterium\_chubuense\_NBB4
Mycobacterium\_chubuense\_NBB4
no rank

6.546.546.546.546.546.546.546.546.546.546.546.546.546.546.546.546.546.546.546.54
22222222222222222222
3.087193.138333.079442.945910000000000000000
88870000000000000000
1769
species

3.273.273.273.273.273.273.273.273.273.273.273.273.273.273.273.273.273.273.273.27
11111111111111111111
3.564953.197223.079442.945910000000000000000
139870000000000000000
272631

Mycobacterium\_leprae\_TN
Mycobacterium\_leprae\_TN
Mycobacterium\_leprae\_TN
Mycobacterium\_leprae\_TN
Mycobacterium\_leprae\_TN
Mycobacterium\_leprae\_TN
Mycobacterium\_leprae\_TN
Mycobacterium\_leprae\_TN
Mycobacterium\_leprae\_TN
Mycobacterium\_leprae\_TN
Mycobacterium\_leprae\_TN
Mycobacterium\_leprae\_TN
Mycobacterium\_leprae\_TN
Mycobacterium\_leprae\_TN
Mycobacterium\_leprae\_TN
Mycobacterium\_leprae\_TN
Mycobacterium\_leprae\_TN
Mycobacterium\_leprae\_TN
Mycobacterium\_leprae\_TN
Mycobacterium\_leprae\_TN
no rank

3.273.273.273.273.273.273.273.273.273.273.273.273.273.273.273.273.273.273.273.27
11111111111111111111
2.609443.079443.079442.945910000000000000000
58870000000000000000
561304

Mycobacterium\_leprae\_Br4923
Mycobacterium\_leprae\_Br4923
Mycobacterium\_leprae\_Br4923
Mycobacterium\_leprae\_Br4923
Mycobacterium\_leprae\_Br4923
Mycobacterium\_leprae\_Br4923
Mycobacterium\_leprae\_Br4923
Mycobacterium\_leprae\_Br4923
Mycobacterium\_leprae\_Br4923
Mycobacterium\_leprae\_Br4923
Mycobacterium\_leprae\_Br4923
Mycobacterium\_leprae\_Br4923
Mycobacterium\_leprae\_Br4923
Mycobacterium\_leprae\_Br4923
Mycobacterium\_leprae\_Br4923
Mycobacterium\_leprae\_Br4923
Mycobacterium\_leprae\_Br4923
Mycobacterium\_leprae\_Br4923
Mycobacterium\_leprae\_Br4923
Mycobacterium\_leprae\_Br4923
no rank

5.615.615.615.615.615.615.615.615.615.615.615.615.615.615.615.615.615.615.615.61
11111111111111111111
3.564953.197223.079442.945911.693151.693151.693151.693151.693151.693151.693151.693151.693150000000
139872222222220000000
1168287

Mycobacterium\_sp.\_MOTT36Y
Mycobacterium\_sp.\_MOTT36Y
Mycobacterium\_sp.\_MOTT36Y
Mycobacterium\_sp.\_MOTT36Y
Mycobacterium\_sp.\_MOTT36Y
Mycobacterium\_sp.\_MOTT36Y
Mycobacterium\_sp.\_MOTT36Y
Mycobacterium\_sp.\_MOTT36Y
Mycobacterium\_sp.\_MOTT36Y
Mycobacterium\_sp.\_MOTT36Y
Mycobacterium\_sp.\_MOTT36Y
Mycobacterium\_sp.\_MOTT36Y
Mycobacterium\_sp.\_MOTT36Y
Mycobacterium\_sp.\_MOTT36Y
Mycobacterium\_sp.\_MOTT36Y
Mycobacterium\_sp.\_MOTT36Y
Mycobacterium\_sp.\_MOTT36Y
Mycobacterium\_sp.\_MOTT36Y
Mycobacterium\_sp.\_MOTT36Y
Mycobacterium\_sp.\_MOTT36Y
species

21.2021.2021.2021.2021.2021.2021.2021.2021.2021.2021.2021.2021.2021.2021.2021.2021.2021.2021.2021.20
33333333333333333333
4.258543.935333.9243.829151.345061.345061.345061.345061.345061.345061.345061.345061.345061.345061.250211.250211.116520.8879761.116521.11652
261919171111111111111111
1772
species

7.227.227.227.227.227.227.227.227.227.227.227.227.227.227.227.227.227.227.227.22
11111111111111111111
3.197222.791762.791762.791760000000000000000
96660000000000000000
710686

Mycobacterium\_smegmatis\_JS623
Mycobacterium\_smegmatis\_JS623
Mycobacterium\_smegmatis\_JS623
Mycobacterium\_smegmatis\_JS623
Mycobacterium\_smegmatis\_JS623
Mycobacterium\_smegmatis\_JS623
Mycobacterium\_smegmatis\_JS623
Mycobacterium\_smegmatis\_JS623
Mycobacterium\_smegmatis\_JS623
Mycobacterium\_smegmatis\_JS623
Mycobacterium\_smegmatis\_JS623
Mycobacterium\_smegmatis\_JS623
Mycobacterium\_smegmatis\_JS623
Mycobacterium\_smegmatis\_JS623
Mycobacterium\_smegmatis\_JS623
Mycobacterium\_smegmatis\_JS623
Mycobacterium\_smegmatis\_JS623
Mycobacterium\_smegmatis\_JS623
Mycobacterium\_smegmatis\_JS623
Mycobacterium\_smegmatis\_JS623
no rank

13.9813.9813.9813.9813.9813.9813.9813.9813.9813.9813.9813.9813.9813.9813.9813.9813.9813.9813.9813.98
22222222222222222222
4.806664.525934.508754.364912.039722.039722.039722.039722.039722.039722.039722.039722.039722.039721.895881.895881.693151.346571.693151.69315
453433293333333333222122
246196

Mycobacterium\_smegmatis\_str.\_MC2\_155Mycobacterium\_smegmatis\_str.\_MC2\_155
Mycobacterium\_smegmatis\_str.\_MC2\_155Mycobacterium\_smegmatis\_str.\_MC2\_155
Mycobacterium\_smegmatis\_str.\_MC2\_155Mycobacterium\_smegmatis\_str.\_MC2\_155
Mycobacterium\_smegmatis\_str.\_MC2\_155Mycobacterium\_smegmatis\_str.\_MC2\_155
Mycobacterium\_smegmatis\_str.\_MC2\_155Mycobacterium\_smegmatis\_str.\_MC2\_155
Mycobacterium\_smegmatis\_str.\_MC2\_155Mycobacterium\_smegmatis\_str.\_MC2\_155
Mycobacterium\_smegmatis\_str.\_MC2\_155Mycobacterium\_smegmatis\_str.\_MC2\_155
Mycobacterium\_smegmatis\_str.\_MC2\_155Mycobacterium\_smegmatis\_str.\_MC2\_155
Mycobacterium\_smegmatis\_str.\_MC2\_155Mycobacterium\_smegmatis\_str.\_MC2\_155
Mycobacterium\_smegmatis\_str.\_MC2\_155Mycobacterium\_smegmatis\_str.\_MC2\_155
Mycobacterium\_smegmatis\_str.\_MC2\_155Mycobacterium\_smegmatis\_str.\_MC2\_155
Mycobacterium\_smegmatis\_str.\_MC2\_155Mycobacterium\_smegmatis\_str.\_MC2\_155
Mycobacterium\_smegmatis\_str.\_MC2\_155Mycobacterium\_smegmatis\_str.\_MC2\_155
Mycobacterium\_smegmatis\_str.\_MC2\_155Mycobacterium\_smegmatis\_str.\_MC2\_155
Mycobacterium\_smegmatis\_str.\_MC2\_155Mycobacterium\_smegmatis\_str.\_MC2\_155
Mycobacterium\_smegmatis\_str.\_MC2\_155Mycobacterium\_smegmatis\_str.\_MC2\_155
Mycobacterium\_smegmatis\_str.\_MC2\_155Mycobacterium\_smegmatis\_str.\_MC2\_155
Mycobacterium\_smegmatis\_str.\_MC2\_155Mycobacterium\_smegmatis\_str.\_MC2\_155
Mycobacterium\_smegmatis\_str.\_MC2\_155Mycobacterium\_smegmatis\_str.\_MC2\_155
Mycobacterium\_smegmatis\_str.\_MC2\_155Mycobacterium\_smegmatis\_str.\_MC2\_155
no rank

4.864.864.864.864.864.864.864.864.864.864.864.864.864.864.864.864.864.864.864.86
11111111111111111111
3.197223.197222.791762.609440000000000000000
99650000000000000000
992401
genus

4.864.864.864.864.864.864.864.864.864.864.864.864.864.864.864.864.864.864.864.86
11111111111111111111
3.197223.197222.791762.609440000000000000000
99650000000000000000
639313
species

4.864.864.864.864.864.864.864.864.864.864.864.864.864.864.864.864.864.864.864.86
11111111111111111111
3.197223.197222.791762.609440000000000000000
99650000000000000000
443218

Amycolicicoccus\_subflavus\_DQS3-9A1
Amycolicicoccus\_subflavus\_DQS3-9A1
Amycolicicoccus\_subflavus\_DQS3-9A1
Amycolicicoccus\_subflavus\_DQS3-9A1
Amycolicicoccus\_subflavus\_DQS3-9A1
Amycolicicoccus\_subflavus\_DQS3-9A1
Amycolicicoccus\_subflavus\_DQS3-9A1
Amycolicicoccus\_subflavus\_DQS3-9A1
Amycolicicoccus\_subflavus\_DQS3-9A1
Amycolicicoccus\_subflavus\_DQS3-9A1
Amycolicicoccus\_subflavus\_DQS3-9A1
Amycolicicoccus\_subflavus\_DQS3-9A1
Amycolicicoccus\_subflavus\_DQS3-9A1
Amycolicicoccus\_subflavus\_DQS3-9A1
Amycolicicoccus\_subflavus\_DQS3-9A1
Amycolicicoccus\_subflavus\_DQS3-9A1
Amycolicicoccus\_subflavus\_DQS3-9A1
Amycolicicoccus\_subflavus\_DQS3-9A1
Amycolicicoccus\_subflavus\_DQS3-9A1
Amycolicicoccus\_subflavus\_DQS3-9A1
no rank

4.484.484.484.484.484.484.484.484.484.484.484.484.484.484.484.484.484.484.484.48
11111111111111111111
4.465744.135494.044523.833211000000000000000
322321171000000000000000
85028
family

4.484.484.484.484.484.484.484.484.484.484.484.484.484.484.484.484.484.484.484.48
11111111111111111111
4.465744.135494.044523.833211000000000000000
322321171000000000000000
2060
genus

4.484.484.484.484.484.484.484.484.484.484.484.484.484.484.484.484.484.484.484.48
11111111111111111111
4.465744.135494.044523.833211000000000000000
322321171000000000000000
2061
species

4.484.484.484.484.484.484.484.484.484.484.484.484.484.484.484.484.484.484.484.48
11111111111111111111
4.465744.135494.044523.833211000000000000000
322321171000000000000000
521096

Tsukamurella\_paurometabola\_DSM\_20162
Tsukamurella\_paurometabola\_DSM\_20162
Tsukamurella\_paurometabola\_DSM\_20162
Tsukamurella\_paurometabola\_DSM\_20162
Tsukamurella\_paurometabola\_DSM\_20162
Tsukamurella\_paurometabola\_DSM\_20162
Tsukamurella\_paurometabola\_DSM\_20162
Tsukamurella\_paurometabola\_DSM\_20162
Tsukamurella\_paurometabola\_DSM\_20162
Tsukamurella\_paurometabola\_DSM\_20162
Tsukamurella\_paurometabola\_DSM\_20162
Tsukamurella\_paurometabola\_DSM\_20162
Tsukamurella\_paurometabola\_DSM\_20162
Tsukamurella\_paurometabola\_DSM\_20162
Tsukamurella\_paurometabola\_DSM\_20162
Tsukamurella\_paurometabola\_DSM\_20162
Tsukamurella\_paurometabola\_DSM\_20162
Tsukamurella\_paurometabola\_DSM\_20162
Tsukamurella\_paurometabola\_DSM\_20162
Tsukamurella\_paurometabola\_DSM\_20162
no rank

3.163.163.163.163.163.163.163.163.163.163.163.163.163.163.163.163.163.163.163.16
11111111111111111111
2.098612.098612.098612.098610000000000000000
33330000000000000000
316606
family

3.163.163.163.163.163.163.163.163.163.163.163.163.163.163.163.163.163.163.163.16
11111111111111111111
2.098612.098612.098612.098610000000000000000
33330000000000000000
286801
genus

3.163.163.163.163.163.163.163.163.163.163.163.163.163.163.163.163.163.163.163.16
11111111111111111111
2.098612.098612.098612.098610000000000000000
33330000000000000000
286802
species

3.163.163.163.163.163.163.163.163.163.163.163.163.163.163.163.163.163.163.163.16
11111111111111111111
2.098612.098612.098612.098610000000000000000
33330000000000000000
640132

Segniliparus\_rotundus\_DSM\_44985
Segniliparus\_rotundus\_DSM\_44985
Segniliparus\_rotundus\_DSM\_44985
Segniliparus\_rotundus\_DSM\_44985
Segniliparus\_rotundus\_DSM\_44985
Segniliparus\_rotundus\_DSM\_44985
Segniliparus\_rotundus\_DSM\_44985
Segniliparus\_rotundus\_DSM\_44985
Segniliparus\_rotundus\_DSM\_44985
Segniliparus\_rotundus\_DSM\_44985
Segniliparus\_rotundus\_DSM\_44985
Segniliparus\_rotundus\_DSM\_44985
Segniliparus\_rotundus\_DSM\_44985
Segniliparus\_rotundus\_DSM\_44985
Segniliparus\_rotundus\_DSM\_44985
Segniliparus\_rotundus\_DSM\_44985
Segniliparus\_rotundus\_DSM\_44985
Segniliparus\_rotundus\_DSM\_44985
Segniliparus\_rotundus\_DSM\_44985
Segniliparus\_rotundus\_DSM\_44985
no rank

17.0217.0217.0217.0217.0217.0217.0217.0217.0217.0217.0217.0217.0217.0217.0217.0217.0217.0217.0217.02
33333333333333333333
3.966353.479033.312143.172231.215440.5262480.5262480.5262480.5262480.5262480.5262480.5262480.526248000.3108110000
19121091111111110010000
85026
family

17.0217.0217.0217.0217.0217.0217.0217.0217.0217.0217.0217.0217.0217.0217.0217.0217.0217.0217.0217.02
33333333333333333333
3.966353.479033.312143.172231.215440.5262480.5262480.5262480.5262480.5262480.5262480.5262480.526248000.3108110000
19121091111111110010000
2053
genus

5.845.845.845.845.845.845.845.845.845.845.845.845.845.845.845.845.845.845.845.84
11111111111111111111
3.484913.197222.945912.791761000000000000000
129761000000000000000
84595
species

5.845.845.845.845.845.845.845.845.845.845.845.845.845.845.845.845.845.845.845.84
11111111111111111111
3.484913.197222.945912.791761000000000000000
129761000000000000000
1112204

Gordonia\_polyisoprenivorans\_VH2
Gordonia\_polyisoprenivorans\_VH2
Gordonia\_polyisoprenivorans\_VH2
Gordonia\_polyisoprenivorans\_VH2
Gordonia\_polyisoprenivorans\_VH2
Gordonia\_polyisoprenivorans\_VH2
Gordonia\_polyisoprenivorans\_VH2
Gordonia\_polyisoprenivorans\_VH2
Gordonia\_polyisoprenivorans\_VH2
Gordonia\_polyisoprenivorans\_VH2
Gordonia\_polyisoprenivorans\_VH2
Gordonia\_polyisoprenivorans\_VH2
Gordonia\_polyisoprenivorans\_VH2
Gordonia\_polyisoprenivorans\_VH2
Gordonia\_polyisoprenivorans\_VH2
Gordonia\_polyisoprenivorans\_VH2
Gordonia\_polyisoprenivorans\_VH2
Gordonia\_polyisoprenivorans\_VH2
Gordonia\_polyisoprenivorans\_VH2
Gordonia\_polyisoprenivorans\_VH2
no rank

5.295.295.295.295.295.295.295.295.295.295.295.295.295.295.295.295.295.295.295.29
11111111111111111111
4.713574.555354.295844.218881.693151.693151.693151.693151.693151.693151.693151.693151.693150010000
413527252222222220010000
2054
species

5.295.295.295.295.295.295.295.295.295.295.295.295.295.295.295.295.295.295.295.29
11111111111111111111
4.713574.555354.295844.218881.693151.693151.693151.693151.693151.693151.693151.693151.693150010000
413527252222222220010000
526226

Gordonia\_bronchialis\_DSM\_43247
Gordonia\_bronchialis\_DSM\_43247
Gordonia\_bronchialis\_DSM\_43247
Gordonia\_bronchialis\_DSM\_43247
Gordonia\_bronchialis\_DSM\_43247
Gordonia\_bronchialis\_DSM\_43247
Gordonia\_bronchialis\_DSM\_43247
Gordonia\_bronchialis\_DSM\_43247
Gordonia\_bronchialis\_DSM\_43247
Gordonia\_bronchialis\_DSM\_43247
Gordonia\_bronchialis\_DSM\_43247
Gordonia\_bronchialis\_DSM\_43247
Gordonia\_bronchialis\_DSM\_43247
Gordonia\_bronchialis\_DSM\_43247
Gordonia\_bronchialis\_DSM\_43247
Gordonia\_bronchialis\_DSM\_43247
Gordonia\_bronchialis\_DSM\_43247
Gordonia\_bronchialis\_DSM\_43247
Gordonia\_bronchialis\_DSM\_43247
Gordonia\_bronchialis\_DSM\_43247
no rank

5.895.895.895.895.895.895.895.895.895.895.895.895.895.895.895.895.895.895.895.89
11111111111111111111
3.772592.791762.791762.609441000000000000000
166651000000000000000
337191

Gordonia\_sp.\_KTR9
Gordonia\_sp.\_KTR9
Gordonia\_sp.\_KTR9
Gordonia\_sp.\_KTR9
Gordonia\_sp.\_KTR9
Gordonia\_sp.\_KTR9
Gordonia\_sp.\_KTR9
Gordonia\_sp.\_KTR9
Gordonia\_sp.\_KTR9
Gordonia\_sp.\_KTR9
Gordonia\_sp.\_KTR9
Gordonia\_sp.\_KTR9
Gordonia\_sp.\_KTR9
Gordonia\_sp.\_KTR9
Gordonia\_sp.\_KTR9
Gordonia\_sp.\_KTR9
Gordonia\_sp.\_KTR9
Gordonia\_sp.\_KTR9
Gordonia\_sp.\_KTR9
Gordonia\_sp.\_KTR9
species

32.0432.0432.0432.0432.0432.0432.0432.0432.0432.0432.0432.0432.0432.0432.0432.0432.0432.0432.0432.04
55555555555555555555
3.251553.18032.923732.888320.38015000000000000000
109771000000000000000
85012
suborder

5.645.645.645.645.645.645.645.645.645.645.645.645.645.645.645.645.645.645.645.64
11111111111111111111
2.386292.609442.386292.386291000000000000000
45441000000000000000
2012
family

5.645.645.645.645.645.645.645.645.645.645.645.645.645.645.645.645.645.645.645.64
11111111111111111111
2.386292.609442.386292.386291000000000000000
45441000000000000000
2019
genus

5.645.645.645.645.645.645.645.645.645.645.645.645.645.645.645.645.645.645.645.64
11111111111111111111
2.386292.609442.386292.386291000000000000000
45441000000000000000
2020
species

5.645.645.645.645.645.645.645.645.645.645.645.645.645.645.645.645.645.645.645.64
11111111111111111111
2.386292.609442.386292.386291000000000000000
45441000000000000000
471852

Thermomonospora\_curvata\_DSM\_43183
Thermomonospora\_curvata\_DSM\_43183
Thermomonospora\_curvata\_DSM\_43183
Thermomonospora\_curvata\_DSM\_43183
Thermomonospora\_curvata\_DSM\_43183
Thermomonospora\_curvata\_DSM\_43183
Thermomonospora\_curvata\_DSM\_43183
Thermomonospora\_curvata\_DSM\_43183
Thermomonospora\_curvata\_DSM\_43183
Thermomonospora\_curvata\_DSM\_43183
Thermomonospora\_curvata\_DSM\_43183
Thermomonospora\_curvata\_DSM\_43183
Thermomonospora\_curvata\_DSM\_43183
Thermomonospora\_curvata\_DSM\_43183
Thermomonospora\_curvata\_DSM\_43183
Thermomonospora\_curvata\_DSM\_43183
Thermomonospora\_curvata\_DSM\_43183
Thermomonospora\_curvata\_DSM\_43183
Thermomonospora\_curvata\_DSM\_43183
Thermomonospora\_curvata\_DSM\_43183
no rank

16.0316.0316.0316.0316.0316.0316.0316.0316.0316.0316.0316.0316.0316.0316.0316.0316.0316.0316.0316.03
33333333333333333333
3.074552.958772.867732.865110.407985000000000000000
87661000000000000000
83676
family

3.643.643.643.643.643.643.643.643.643.643.643.643.643.643.643.643.643.643.643.64
11111111111111111111
2.791762.609442.609442.386290000000000000000
65540000000000000000
83677
genus

3.643.643.643.643.643.643.643.643.643.643.643.643.643.643.643.643.643.643.643.64
11111111111111111111
2.791762.609442.609442.386290000000000000000
65540000000000000000
2021
species

3.643.643.643.643.643.643.643.643.643.643.643.643.643.643.643.643.643.643.643.64
11111111111111111111
2.791762.609442.609442.386290000000000000000
65540000000000000000
269800

Thermobifida\_fusca\_YX
Thermobifida\_fusca\_YX
Thermobifida\_fusca\_YX
Thermobifida\_fusca\_YX
Thermobifida\_fusca\_YX
Thermobifida\_fusca\_YX
Thermobifida\_fusca\_YX
Thermobifida\_fusca\_YX
Thermobifida\_fusca\_YX
Thermobifida\_fusca\_YX
Thermobifida\_fusca\_YX
Thermobifida\_fusca\_YX
Thermobifida\_fusca\_YX
Thermobifida\_fusca\_YX
Thermobifida\_fusca\_YX
Thermobifida\_fusca\_YX
Thermobifida\_fusca\_YX
Thermobifida\_fusca\_YX
Thermobifida\_fusca\_YX
Thermobifida\_fusca\_YX
no rank

12.3912.3912.3912.3912.3912.3912.3912.3912.3912.3912.3912.3912.3912.3912.3912.3912.3912.3912.3912.39
22222222222222222222
3.157633.06142.943613.005780.527845000000000000000
98771000000000000000
2013
genus

6.546.546.546.546.546.546.546.546.546.546.546.546.546.546.546.546.546.546.546.54
11111111111111111111
3.484913.302593.079443.197221000000000000000
1210891000000000000000
2014
species

6.546.546.546.546.546.546.546.546.546.546.546.546.546.546.546.546.546.546.546.54
11111111111111111111
3.484913.302593.079443.197221000000000000000
1210891000000000000000
568208
subspecies

6.546.546.546.546.546.546.546.546.546.546.546.546.546.546.546.546.546.546.546.54
11111111111111111111
3.484913.302593.079443.197221000000000000000
1210891000000000000000
446468

Nocardiopsis\_dassonvillei\_subsp.\_dassonvillei\_DSM\_43111
Nocardiopsis\_dassonvillei\_subsp.\_dassonvillei\_DSM\_43111
Nocardiopsis\_dassonvillei\_subsp.\_dassonvillei\_DSM\_43111
Nocardiopsis\_dassonvillei\_subsp.\_dassonvillei\_DSM\_43111
Nocardiopsis\_dassonvillei\_subsp.\_dassonvillei\_DSM\_43111
Nocardiopsis\_dassonvillei\_subsp.\_dassonvillei\_DSM\_43111
Nocardiopsis\_dassonvillei\_subsp.\_dassonvillei\_DSM\_43111
Nocardiopsis\_dassonvillei\_subsp.\_dassonvillei\_DSM\_43111
Nocardiopsis\_dassonvillei\_subsp.\_dassonvillei\_DSM\_43111
Nocardiopsis\_dassonvillei\_subsp.\_dassonvillei\_DSM\_43111
Nocardiopsis\_dassonvillei\_subsp.\_dassonvillei\_DSM\_43111
Nocardiopsis\_dassonvillei\_subsp.\_dassonvillei\_DSM\_43111
Nocardiopsis\_dassonvillei\_subsp.\_dassonvillei\_DSM\_43111
Nocardiopsis\_dassonvillei\_subsp.\_dassonvillei\_DSM\_43111
Nocardiopsis\_dassonvillei\_subsp.\_dassonvillei\_DSM\_43111
Nocardiopsis\_dassonvillei\_subsp.\_dassonvillei\_DSM\_43111
Nocardiopsis\_dassonvillei\_subsp.\_dassonvillei\_DSM\_43111
Nocardiopsis\_dassonvillei\_subsp.\_dassonvillei\_DSM\_43111
Nocardiopsis\_dassonvillei\_subsp.\_dassonvillei\_DSM\_43111
Nocardiopsis\_dassonvillei\_subsp.\_dassonvillei\_DSM\_43111
no rank

5.855.855.855.855.855.855.855.855.855.855.855.855.855.855.855.855.855.855.855.85
11111111111111111111
2.791762.791762.791762.791760000000000000000
66660000000000000000
53437
species

5.855.855.855.855.855.855.855.855.855.855.855.855.855.855.855.855.855.855.855.85
11111111111111111111
2.791762.791762.791762.791760000000000000000
66660000000000000000
1205910

Nocardiopsis\_alba\_ATCC\_BAA-2165
Nocardiopsis\_alba\_ATCC\_BAA-2165
Nocardiopsis\_alba\_ATCC\_BAA-2165
Nocardiopsis\_alba\_ATCC\_BAA-2165
Nocardiopsis\_alba\_ATCC\_BAA-2165
Nocardiopsis\_alba\_ATCC\_BAA-2165
Nocardiopsis\_alba\_ATCC\_BAA-2165
Nocardiopsis\_alba\_ATCC\_BAA-2165
Nocardiopsis\_alba\_ATCC\_BAA-2165
Nocardiopsis\_alba\_ATCC\_BAA-2165
Nocardiopsis\_alba\_ATCC\_BAA-2165
Nocardiopsis\_alba\_ATCC\_BAA-2165
Nocardiopsis\_alba\_ATCC\_BAA-2165
Nocardiopsis\_alba\_ATCC\_BAA-2165
Nocardiopsis\_alba\_ATCC\_BAA-2165
Nocardiopsis\_alba\_ATCC\_BAA-2165
Nocardiopsis\_alba\_ATCC\_BAA-2165
Nocardiopsis\_alba\_ATCC\_BAA-2165
Nocardiopsis\_alba\_ATCC\_BAA-2165
Nocardiopsis\_alba\_ATCC\_BAA-2165
no rank

10.3710.3710.3710.3710.3710.3710.3710.3710.3710.3710.3710.3710.3710.3710.3710.3710.3710.3710.3710.37
11111111111111111111
3.995733.833213.302593.197220000000000000000
20171090000000000000000
2004
family

10.3710.3710.3710.3710.3710.3710.3710.3710.3710.3710.3710.3710.3710.3710.3710.3710.3710.3710.3710.37
11111111111111111111
3.995733.833213.302593.197220000000000000000
20171090000000000000000
2000
genus

10.3710.3710.3710.3710.3710.3710.3710.3710.3710.3710.3710.3710.3710.3710.3710.3710.3710.3710.3710.37
11111111111111111111
3.995733.833213.302593.197220000000000000000
20171090000000000000000
2001
species

10.3710.3710.3710.3710.3710.3710.3710.3710.3710.3710.3710.3710.3710.3710.3710.3710.3710.3710.3710.37
11111111111111111111
3.995733.833213.302593.197220000000000000000
20171090000000000000000
479432

Streptosporangium\_roseum\_DSM\_43021
Streptosporangium\_roseum\_DSM\_43021
Streptosporangium\_roseum\_DSM\_43021
Streptosporangium\_roseum\_DSM\_43021
Streptosporangium\_roseum\_DSM\_43021
Streptosporangium\_roseum\_DSM\_43021
Streptosporangium\_roseum\_DSM\_43021
Streptosporangium\_roseum\_DSM\_43021
Streptosporangium\_roseum\_DSM\_43021
Streptosporangium\_roseum\_DSM\_43021
Streptosporangium\_roseum\_DSM\_43021
Streptosporangium\_roseum\_DSM\_43021
Streptosporangium\_roseum\_DSM\_43021
Streptosporangium\_roseum\_DSM\_43021
Streptosporangium\_roseum\_DSM\_43021
Streptosporangium\_roseum\_DSM\_43021
Streptosporangium\_roseum\_DSM\_43021
Streptosporangium\_roseum\_DSM\_43021
Streptosporangium\_roseum\_DSM\_43021
Streptosporangium\_roseum\_DSM\_43021
no rank

98.3298.3298.3298.3298.3298.3298.3298.3298.3298.3298.3298.3298.3298.3298.3298.3298.3298.3298.3298.32
2828282828282828282828282828282828282828
3.062532.822122.731812.675590.6668490.3984070.2764650.3018920.6684520.3044350.2131170.1982820.1082180.1082180.1082180.1082180.0873094000.0515663
86651100100000000000
85006
suborder

3.613.613.613.613.613.613.613.613.613.613.613.613.613.613.613.613.613.613.613.61
11111111111111111111
2.791762.098612.098612.098610000100000000000
63330000100000000000
85020
family

3.613.613.613.613.613.613.613.613.613.613.613.613.613.613.613.613.613.613.613.61
11111111111111111111
2.791762.098612.098612.098610000100000000000
63330000100000000000
43668
genus

3.613.613.613.613.613.613.613.613.613.613.613.613.613.613.613.613.613.613.613.61
11111111111111111111
2.791762.098612.098612.098610000100000000000
63330000100000000000
43669
species

3.613.613.613.613.613.613.613.613.613.613.613.613.613.613.613.613.613.613.613.61
11111111111111111111
2.791762.098612.098612.098610000100000000000
63330000100000000000
446465

Brachybacterium\_faecium\_DSM\_4810
Brachybacterium\_faecium\_DSM\_4810
Brachybacterium\_faecium\_DSM\_4810
Brachybacterium\_faecium\_DSM\_4810
Brachybacterium\_faecium\_DSM\_4810
Brachybacterium\_faecium\_DSM\_4810
Brachybacterium\_faecium\_DSM\_4810
Brachybacterium\_faecium\_DSM\_4810
Brachybacterium\_faecium\_DSM\_4810
Brachybacterium\_faecium\_DSM\_4810
Brachybacterium\_faecium\_DSM\_4810
Brachybacterium\_faecium\_DSM\_4810
Brachybacterium\_faecium\_DSM\_4810
Brachybacterium\_faecium\_DSM\_4810
Brachybacterium\_faecium\_DSM\_4810
Brachybacterium\_faecium\_DSM\_4810
Brachybacterium\_faecium\_DSM\_4810
Brachybacterium\_faecium\_DSM\_4810
Brachybacterium\_faecium\_DSM\_4810
Brachybacterium\_faecium\_DSM\_4810
no rank

41.9541.9541.9541.9541.9541.9541.9541.9541.9541.9541.9541.9541.9541.9541.9541.9541.9541.9541.9541.95
1111111111111111111111111111111111111111
3.526973.29283.200453.190391.246920.846040.6479620.7075571.034120.6479620.4994920.4647230.2536340.2536340.2536340.2536340.204631000.120858
1310991111111100000000
1268
family

3.163.163.163.163.163.163.163.163.163.163.163.163.163.163.163.163.163.163.163.16
11111111111111111111
3.772593.079442.386292.609441000100000000000
168451000100000000000
1645
genus

3.163.163.163.163.163.163.163.163.163.163.163.163.163.163.163.163.163.163.163.16
11111111111111111111
3.772593.079442.386292.609441000100000000000
168451000100000000000
1646
species

3.163.163.163.163.163.163.163.163.163.163.163.163.163.163.163.163.163.163.163.16
11111111111111111111
3.772593.079442.386292.609441000100000000000
168451000100000000000
288705

Renibacterium\_salmoninarum\_ATCC\_33209
Renibacterium\_salmoninarum\_ATCC\_33209
Renibacterium\_salmoninarum\_ATCC\_33209
Renibacterium\_salmoninarum\_ATCC\_33209
Renibacterium\_salmoninarum\_ATCC\_33209
Renibacterium\_salmoninarum\_ATCC\_33209
Renibacterium\_salmoninarum\_ATCC\_33209
Renibacterium\_salmoninarum\_ATCC\_33209
Renibacterium\_salmoninarum\_ATCC\_33209
Renibacterium\_salmoninarum\_ATCC\_33209
Renibacterium\_salmoninarum\_ATCC\_33209
Renibacterium\_salmoninarum\_ATCC\_33209
Renibacterium\_salmoninarum\_ATCC\_33209
Renibacterium\_salmoninarum\_ATCC\_33209
Renibacterium\_salmoninarum\_ATCC\_33209
Renibacterium\_salmoninarum\_ATCC\_33209
Renibacterium\_salmoninarum\_ATCC\_33209
Renibacterium\_salmoninarum\_ATCC\_33209
Renibacterium\_salmoninarum\_ATCC\_33209
Renibacterium\_salmoninarum\_ATCC\_33209
no rank

4.774.774.774.774.774.774.774.774.774.774.774.774.774.774.774.774.774.774.774.77
22222222222222222222
2.503712.503712.503712.503710.8022040.526205000.47379500000000000
44441100100000000000
32207
genus

2.512.512.512.512.512.512.512.512.512.512.512.512.512.512.512.512.512.512.512.51
11111111111111111111
2.609442.609442.609442.609440100000000000000
55550100000000000000
2047
species

2.512.512.512.512.512.512.512.512.512.512.512.512.512.512.512.512.512.512.512.51
11111111111111111111
2.609442.609442.609442.609440100000000000000
55550100000000000000
762948

Rothia\_dentocariosa\_ATCC\_17931
Rothia\_dentocariosa\_ATCC\_17931
Rothia\_dentocariosa\_ATCC\_17931
Rothia\_dentocariosa\_ATCC\_17931
Rothia\_dentocariosa\_ATCC\_17931
Rothia\_dentocariosa\_ATCC\_17931
Rothia\_dentocariosa\_ATCC\_17931
Rothia\_dentocariosa\_ATCC\_17931
Rothia\_dentocariosa\_ATCC\_17931
Rothia\_dentocariosa\_ATCC\_17931
Rothia\_dentocariosa\_ATCC\_17931
Rothia\_dentocariosa\_ATCC\_17931
Rothia\_dentocariosa\_ATCC\_17931
Rothia\_dentocariosa\_ATCC\_17931
Rothia\_dentocariosa\_ATCC\_17931
Rothia\_dentocariosa\_ATCC\_17931
Rothia\_dentocariosa\_ATCC\_17931
Rothia\_dentocariosa\_ATCC\_17931
Rothia\_dentocariosa\_ATCC\_17931
Rothia\_dentocariosa\_ATCC\_17931
no rank

2.262.262.262.262.262.262.262.262.262.262.262.262.262.262.262.262.262.262.262.26
11111111111111111111
2.386292.386292.386292.386291.69315000100000000000
44442000100000000000
43675
species

2.262.262.262.262.262.262.262.262.262.262.262.262.262.262.262.262.262.262.262.26
11111111111111111111
2.386292.386292.386292.386291.69315000100000000000
44442000100000000000
680646

Rothia\_mucilaginosa\_DY-18
Rothia\_mucilaginosa\_DY-18
Rothia\_mucilaginosa\_DY-18
Rothia\_mucilaginosa\_DY-18
Rothia\_mucilaginosa\_DY-18
Rothia\_mucilaginosa\_DY-18
Rothia\_mucilaginosa\_DY-18
Rothia\_mucilaginosa\_DY-18
Rothia\_mucilaginosa\_DY-18
Rothia\_mucilaginosa\_DY-18
Rothia\_mucilaginosa\_DY-18
Rothia\_mucilaginosa\_DY-18
Rothia\_mucilaginosa\_DY-18
Rothia\_mucilaginosa\_DY-18
Rothia\_mucilaginosa\_DY-18
Rothia\_mucilaginosa\_DY-18
Rothia\_mucilaginosa\_DY-18
Rothia\_mucilaginosa\_DY-18
Rothia\_mucilaginosa\_DY-18
Rothia\_mucilaginosa\_DY-18
no rank

2.702.702.702.702.702.702.702.702.702.702.702.702.702.702.702.702.702.702.702.70
11111111111111111111
2.609442.609442.609442.609440000000000000000
55550000000000000000
57493
genus

2.702.702.702.702.702.702.702.702.702.702.702.702.702.702.702.702.702.702.702.70
11111111111111111111
2.609442.609442.609442.609440000000000000000
55550000000000000000
72000
species

2.702.702.702.702.702.702.702.702.702.702.702.702.702.702.702.702.702.702.702.70
11111111111111111111
2.609442.609442.609442.609440000000000000000
55550000000000000000
378753

Kocuria\_rhizophila\_DC2201
Kocuria\_rhizophila\_DC2201
Kocuria\_rhizophila\_DC2201
Kocuria\_rhizophila\_DC2201
Kocuria\_rhizophila\_DC2201
Kocuria\_rhizophila\_DC2201
Kocuria\_rhizophila\_DC2201
Kocuria\_rhizophila\_DC2201
Kocuria\_rhizophila\_DC2201
Kocuria\_rhizophila\_DC2201
Kocuria\_rhizophila\_DC2201
Kocuria\_rhizophila\_DC2201
Kocuria\_rhizophila\_DC2201
Kocuria\_rhizophila\_DC2201
Kocuria\_rhizophila\_DC2201
Kocuria\_rhizophila\_DC2201
Kocuria\_rhizophila\_DC2201
Kocuria\_rhizophila\_DC2201
Kocuria\_rhizophila\_DC2201
Kocuria\_rhizophila\_DC2201
no rank

2.502.502.502.502.502.502.502.502.502.502.502.502.502.502.502.502.502.502.502.50
11111111111111111111
3.833212.945912.609442.791760101100000000000
177560101100000000000
1269
genus

2.502.502.502.502.502.502.502.502.502.502.502.502.502.502.502.502.502.502.502.50
11111111111111111111
3.833212.945912.609442.791760101100000000000
177560101100000000000
1270
species

2.502.502.502.502.502.502.502.502.502.502.502.502.502.502.502.502.502.502.502.50
11111111111111111111
3.833212.945912.609442.791760101100000000000
177560101100000000000
465515

Micrococcus\_luteus\_NCTC\_2665
Micrococcus\_luteus\_NCTC\_2665
Micrococcus\_luteus\_NCTC\_2665
Micrococcus\_luteus\_NCTC\_2665
Micrococcus\_luteus\_NCTC\_2665
Micrococcus\_luteus\_NCTC\_2665
Micrococcus\_luteus\_NCTC\_2665
Micrococcus\_luteus\_NCTC\_2665
Micrococcus\_luteus\_NCTC\_2665
Micrococcus\_luteus\_NCTC\_2665
Micrococcus\_luteus\_NCTC\_2665
Micrococcus\_luteus\_NCTC\_2665
Micrococcus\_luteus\_NCTC\_2665
Micrococcus\_luteus\_NCTC\_2665
Micrococcus\_luteus\_NCTC\_2665
Micrococcus\_luteus\_NCTC\_2665
Micrococcus\_luteus\_NCTC\_2665
Micrococcus\_luteus\_NCTC\_2665
Micrococcus\_luteus\_NCTC\_2665
Micrococcus\_luteus\_NCTC\_2665
no rank

28.8228.8228.8228.8228.8228.8228.8228.8228.8228.8228.8228.8228.8228.8228.8228.8228.8228.8228.8228.82
66666666666666666666
3.728793.540913.511673.456751.572591.057650.9431650.9431651.230440.9431650.7270530.6764440.3691870.3691870.3691870.3691870.297858000.17592
151312122111111111110000
1663
genus

4.984.984.984.984.984.984.984.984.984.984.984.984.984.984.984.984.984.984.984.98
11111111111111111111
2.609442.609442.609442.609441000100000000000
55551000100000000000
85085
species

4.984.984.984.984.984.984.984.984.984.984.984.984.984.984.984.984.984.984.984.98
11111111111111111111
2.609442.609442.609442.609441000100000000000
55551000100000000000
452863

Arthrobacter\_chlorophenolicus\_A6
Arthrobacter\_chlorophenolicus\_A6
Arthrobacter\_chlorophenolicus\_A6
Arthrobacter\_chlorophenolicus\_A6
Arthrobacter\_chlorophenolicus\_A6
Arthrobacter\_chlorophenolicus\_A6
Arthrobacter\_chlorophenolicus\_A6
Arthrobacter\_chlorophenolicus\_A6
Arthrobacter\_chlorophenolicus\_A6
Arthrobacter\_chlorophenolicus\_A6
Arthrobacter\_chlorophenolicus\_A6
Arthrobacter\_chlorophenolicus\_A6
Arthrobacter\_chlorophenolicus\_A6
Arthrobacter\_chlorophenolicus\_A6
Arthrobacter\_chlorophenolicus\_A6
Arthrobacter\_chlorophenolicus\_A6
Arthrobacter\_chlorophenolicus\_A6
Arthrobacter\_chlorophenolicus\_A6
Arthrobacter\_chlorophenolicus\_A6
Arthrobacter\_chlorophenolicus\_A6
no rank

3.923.923.923.923.923.923.923.923.923.923.923.923.923.923.923.923.923.923.923.92
11111111111111111111
4.713574.295844.178054.091041000000000000000
412724221000000000000000
256701
species

3.923.923.923.923.923.923.923.923.923.923.923.923.923.923.923.923.923.923.923.92
11111111111111111111
4.713574.295844.178054.091041000000000000000
412724221000000000000000
861360

Arthrobacter\_arilaitensis\_Re117
Arthrobacter\_arilaitensis\_Re117
Arthrobacter\_arilaitensis\_Re117
Arthrobacter\_arilaitensis\_Re117
Arthrobacter\_arilaitensis\_Re117
Arthrobacter\_arilaitensis\_Re117
Arthrobacter\_arilaitensis\_Re117
Arthrobacter\_arilaitensis\_Re117
Arthrobacter\_arilaitensis\_Re117
Arthrobacter\_arilaitensis\_Re117
Arthrobacter\_arilaitensis\_Re117
Arthrobacter\_arilaitensis\_Re117
Arthrobacter\_arilaitensis\_Re117
Arthrobacter\_arilaitensis\_Re117
Arthrobacter\_arilaitensis\_Re117
Arthrobacter\_arilaitensis\_Re117
Arthrobacter\_arilaitensis\_Re117
Arthrobacter\_arilaitensis\_Re117
Arthrobacter\_arilaitensis\_Re117
Arthrobacter\_arilaitensis\_Re117
no rank

5.235.235.235.235.235.235.235.235.235.235.235.235.235.235.235.235.235.235.235.23
11111111111111111111
3.772593.772593.639063.639061.693151.693151.693151.693151.693151.693151.693151.6931500000000
161614142222222200000000
43663
species

5.235.235.235.235.235.235.235.235.235.235.235.235.235.235.235.235.235.235.235.23
11111111111111111111
3.772593.772593.639063.639061.693151.693151.693151.693151.693151.693151.693151.6931500000000
161614142222222200000000
290340

Arthrobacter\_aurescens\_TC1
Arthrobacter\_aurescens\_TC1
Arthrobacter\_aurescens\_TC1
Arthrobacter\_aurescens\_TC1
Arthrobacter\_aurescens\_TC1
Arthrobacter\_aurescens\_TC1
Arthrobacter\_aurescens\_TC1
Arthrobacter\_aurescens\_TC1
Arthrobacter\_aurescens\_TC1
Arthrobacter\_aurescens\_TC1
Arthrobacter\_aurescens\_TC1
Arthrobacter\_aurescens\_TC1
Arthrobacter\_aurescens\_TC1
Arthrobacter\_aurescens\_TC1
Arthrobacter\_aurescens\_TC1
Arthrobacter\_aurescens\_TC1
Arthrobacter\_aurescens\_TC1
Arthrobacter\_aurescens\_TC1
Arthrobacter\_aurescens\_TC1
Arthrobacter\_aurescens\_TC1
no rank

5.075.075.075.075.075.075.075.075.075.075.075.075.075.075.075.075.075.075.075.07
11111111111111111111
3.639063.484913.39793.302592.098612.386292.098612.098612.386292.098612.386292.098612.098612.098612.098612.098611.69315001
141211103433434333332001
290399

Arthrobacter\_sp.\_FB24
Arthrobacter\_sp.\_FB24
Arthrobacter\_sp.\_FB24
Arthrobacter\_sp.\_FB24
Arthrobacter\_sp.\_FB24
Arthrobacter\_sp.\_FB24
Arthrobacter\_sp.\_FB24
Arthrobacter\_sp.\_FB24
Arthrobacter\_sp.\_FB24
Arthrobacter\_sp.\_FB24
Arthrobacter\_sp.\_FB24
Arthrobacter\_sp.\_FB24
Arthrobacter\_sp.\_FB24
Arthrobacter\_sp.\_FB24
Arthrobacter\_sp.\_FB24
Arthrobacter\_sp.\_FB24
Arthrobacter\_sp.\_FB24
Arthrobacter\_sp.\_FB24
Arthrobacter\_sp.\_FB24
Arthrobacter\_sp.\_FB24
species

4.544.544.544.544.544.544.544.544.544.544.544.544.544.544.544.544.544.544.544.54
11111111111111111111
4.526364.091044.25814.091042.609442.098611.693151.693152.098611.693150000000000
342226225322320000000000
361575
species

4.544.544.544.544.544.544.544.544.544.544.544.544.544.544.544.544.544.544.544.54
11111111111111111111
4.526364.091044.25814.091042.609442.098611.693151.693152.098611.693150000000000
342226225322320000000000
930171

Arthrobacter\_phenanthrenivorans\_Sphe3
Arthrobacter\_phenanthrenivorans\_Sphe3
Arthrobacter\_phenanthrenivorans\_Sphe3
Arthrobacter\_phenanthrenivorans\_Sphe3
Arthrobacter\_phenanthrenivorans\_Sphe3
Arthrobacter\_phenanthrenivorans\_Sphe3
Arthrobacter\_phenanthrenivorans\_Sphe3
Arthrobacter\_phenanthrenivorans\_Sphe3
Arthrobacter\_phenanthrenivorans\_Sphe3
Arthrobacter\_phenanthrenivorans\_Sphe3
Arthrobacter\_phenanthrenivorans\_Sphe3
Arthrobacter\_phenanthrenivorans\_Sphe3
Arthrobacter\_phenanthrenivorans\_Sphe3
Arthrobacter\_phenanthrenivorans\_Sphe3
Arthrobacter\_phenanthrenivorans\_Sphe3
Arthrobacter\_phenanthrenivorans\_Sphe3
Arthrobacter\_phenanthrenivorans\_Sphe3
Arthrobacter\_phenanthrenivorans\_Sphe3
Arthrobacter\_phenanthrenivorans\_Sphe3
Arthrobacter\_phenanthrenivorans\_Sphe3
no rank

5.085.085.085.085.085.085.085.085.085.085.085.085.085.085.085.085.085.085.085.08
11111111111111111111
3.39793.197223.197223.197221000000000000000
119991000000000000000
1118963

Arthrobacter\_sp.\_Rue61a
Arthrobacter\_sp.\_Rue61a
Arthrobacter\_sp.\_Rue61a
Arthrobacter\_sp.\_Rue61a
Arthrobacter\_sp.\_Rue61a
Arthrobacter\_sp.\_Rue61a
Arthrobacter\_sp.\_Rue61a
Arthrobacter\_sp.\_Rue61a
Arthrobacter\_sp.\_Rue61a
Arthrobacter\_sp.\_Rue61a
Arthrobacter\_sp.\_Rue61a
Arthrobacter\_sp.\_Rue61a
Arthrobacter\_sp.\_Rue61a
Arthrobacter\_sp.\_Rue61a
Arthrobacter\_sp.\_Rue61a
Arthrobacter\_sp.\_Rue61a
Arthrobacter\_sp.\_Rue61a
Arthrobacter\_sp.\_Rue61a
Arthrobacter\_sp.\_Rue61a
Arthrobacter\_sp.\_Rue61a
species

13.3613.3613.3613.3613.3613.3613.3613.3613.3613.3613.3613.3613.3613.3613.3613.3613.3613.3613.3613.36
44444444444444444444
2.893752.74222.699232.538240.2544910000.29790400000000000
76550000000000000000
85023
family

2.582.582.582.582.582.582.582.582.582.582.582.582.582.582.582.582.582.582.582.58
11111111111111111111
3.995734.091044.044523.708050000000000000000
202221150000000000000000
110932
genus

2.582.582.582.582.582.582.582.582.582.582.582.582.582.582.582.582.582.582.582.58
11111111111111111111
3.995734.091044.044523.708050000000000000000
202221150000000000000000
1575
species

2.582.582.582.582.582.582.582.582.582.582.582.582.582.582.582.582.582.582.582.58
11111111111111111111
3.995734.091044.044523.708050000000000000000
202221150000000000000000
59736
subspecies

2.582.582.582.582.582.582.582.582.582.582.582.582.582.582.582.582.582.582.582.58
11111111111111111111
3.995734.091044.044523.708050000000000000000
202221150000000000000000
281090

Leifsonia\_xyli\_subsp.\_xyli\_str.\_CTCB07
Leifsonia\_xyli\_subsp.\_xyli\_str.\_CTCB07
Leifsonia\_xyli\_subsp.\_xyli\_str.\_CTCB07
Leifsonia\_xyli\_subsp.\_xyli\_str.\_CTCB07
Leifsonia\_xyli\_subsp.\_xyli\_str.\_CTCB07
Leifsonia\_xyli\_subsp.\_xyli\_str.\_CTCB07
Leifsonia\_xyli\_subsp.\_xyli\_str.\_CTCB07
Leifsonia\_xyli\_subsp.\_xyli\_str.\_CTCB07
Leifsonia\_xyli\_subsp.\_xyli\_str.\_CTCB07
Leifsonia\_xyli\_subsp.\_xyli\_str.\_CTCB07
Leifsonia\_xyli\_subsp.\_xyli\_str.\_CTCB07
Leifsonia\_xyli\_subsp.\_xyli\_str.\_CTCB07
Leifsonia\_xyli\_subsp.\_xyli\_str.\_CTCB07
Leifsonia\_xyli\_subsp.\_xyli\_str.\_CTCB07
Leifsonia\_xyli\_subsp.\_xyli\_str.\_CTCB07
Leifsonia\_xyli\_subsp.\_xyli\_str.\_CTCB07
Leifsonia\_xyli\_subsp.\_xyli\_str.\_CTCB07
Leifsonia\_xyli\_subsp.\_xyli\_str.\_CTCB07
Leifsonia\_xyli\_subsp.\_xyli\_str.\_CTCB07
Leifsonia\_xyli\_subsp.\_xyli\_str.\_CTCB07
no rank

6.806.806.806.806.806.806.806.806.806.806.806.806.806.806.806.806.806.806.806.80
22222222222222222222
3.178352.844442.777672.589030.5000000000000000
96651000000000000000
1573
genus

6.806.806.806.806.806.806.806.806.806.806.806.806.806.806.806.806.806.806.806.80
22222222222222222222
3.178352.844442.777672.589030.5000000000000000
96651000000000000000
28447
species

3.403.403.403.403.403.403.403.403.403.403.403.403.403.403.403.403.403.403.403.40
11111111111111111111
2.791762.609442.609442.098611000000000000000
65531000000000000000
33013
subspecies

3.403.403.403.403.403.403.403.403.403.403.403.403.403.403.403.403.403.403.403.40
11111111111111111111
2.791762.609442.609442.098611000000000000000
65531000000000000000
443906

Clavibacter\_michiganensis\_subsp.\_michiganensis\_NCPPB\_382
Clavibacter\_michiganensis\_subsp.\_michiganensis\_NCPPB\_382
Clavibacter\_michiganensis\_subsp.\_michiganensis\_NCPPB\_382
Clavibacter\_michiganensis\_subsp.\_michiganensis\_NCPPB\_382
Clavibacter\_michiganensis\_subsp.\_michiganensis\_NCPPB\_382
Clavibacter\_michiganensis\_subsp.\_michiganensis\_NCPPB\_382
Clavibacter\_michiganensis\_subsp.\_michiganensis\_NCPPB\_382
Clavibacter\_michiganensis\_subsp.\_michiganensis\_NCPPB\_382
Clavibacter\_michiganensis\_subsp.\_michiganensis\_NCPPB\_382
Clavibacter\_michiganensis\_subsp.\_michiganensis\_NCPPB\_382
Clavibacter\_michiganensis\_subsp.\_michiganensis\_NCPPB\_382
Clavibacter\_michiganensis\_subsp.\_michiganensis\_NCPPB\_382
Clavibacter\_michiganensis\_subsp.\_michiganensis\_NCPPB\_382
Clavibacter\_michiganensis\_subsp.\_michiganensis\_NCPPB\_382
Clavibacter\_michiganensis\_subsp.\_michiganensis\_NCPPB\_382
Clavibacter\_michiganensis\_subsp.\_michiganensis\_NCPPB\_382
Clavibacter\_michiganensis\_subsp.\_michiganensis\_NCPPB\_382
Clavibacter\_michiganensis\_subsp.\_michiganensis\_NCPPB\_382
Clavibacter\_michiganensis\_subsp.\_michiganensis\_NCPPB\_382
Clavibacter\_michiganensis\_subsp.\_michiganensis\_NCPPB\_382
no rank

3.403.403.403.403.403.403.403.403.403.403.403.403.403.403.403.403.403.403.403.40
11111111111111111111
3.564953.079442.945913.079440000000000000000
138780000000000000000
31964

Clavibacter\_michiganensis\_subsp.\_sepedonicus
Clavibacter\_michiganensis\_subsp.\_sepedonicus
Clavibacter\_michiganensis\_subsp.\_sepedonicus
Clavibacter\_michiganensis\_subsp.\_sepedonicus
Clavibacter\_michiganensis\_subsp.\_sepedonicus
Clavibacter\_michiganensis\_subsp.\_sepedonicus
Clavibacter\_michiganensis\_subsp.\_sepedonicus
Clavibacter\_michiganensis\_subsp.\_sepedonicus
Clavibacter\_michiganensis\_subsp.\_sepedonicus
Clavibacter\_michiganensis\_subsp.\_sepedonicus
Clavibacter\_michiganensis\_subsp.\_sepedonicus
Clavibacter\_michiganensis\_subsp.\_sepedonicus
Clavibacter\_michiganensis\_subsp.\_sepedonicus
Clavibacter\_michiganensis\_subsp.\_sepedonicus
Clavibacter\_michiganensis\_subsp.\_sepedonicus
Clavibacter\_michiganensis\_subsp.\_sepedonicus
Clavibacter\_michiganensis\_subsp.\_sepedonicus
Clavibacter\_michiganensis\_subsp.\_sepedonicus
Clavibacter\_michiganensis\_subsp.\_sepedonicus
Clavibacter\_michiganensis\_subsp.\_sepedonicus
subspecies

3.983.983.983.983.983.983.983.983.983.983.983.983.983.983.983.983.983.983.983.98
11111111111111111111
1.693151.693151.693151.693150000100000000000
22220000100000000000
33882
genus

3.983.983.983.983.983.983.983.983.983.983.983.983.983.983.983.983.983.983.983.98
11111111111111111111
1.693151.693151.693151.693150000100000000000
22220000100000000000
2033
species

3.983.983.983.983.983.983.983.983.983.983.983.983.983.983.983.983.983.983.983.98
11111111111111111111
1.693151.693151.693151.693150000100000000000
22220000100000000000
979556

Microbacterium\_testaceum\_StLB037
Microbacterium\_testaceum\_StLB037
Microbacterium\_testaceum\_StLB037
Microbacterium\_testaceum\_StLB037
Microbacterium\_testaceum\_StLB037
Microbacterium\_testaceum\_StLB037
Microbacterium\_testaceum\_StLB037
Microbacterium\_testaceum\_StLB037
Microbacterium\_testaceum\_StLB037
Microbacterium\_testaceum\_StLB037
Microbacterium\_testaceum\_StLB037
Microbacterium\_testaceum\_StLB037
Microbacterium\_testaceum\_StLB037
Microbacterium\_testaceum\_StLB037
Microbacterium\_testaceum\_StLB037
Microbacterium\_testaceum\_StLB037
Microbacterium\_testaceum\_StLB037
Microbacterium\_testaceum\_StLB037
Microbacterium\_testaceum\_StLB037
Microbacterium\_testaceum\_StLB037
no rank

4.674.674.674.674.674.674.674.674.674.674.674.674.674.674.674.674.674.674.674.67
11111111111111111111
1.693151.693151.693151.693150000000000000000
22220000000000000000
125316
family

4.674.674.674.674.674.674.674.674.674.674.674.674.674.674.674.674.674.674.674.67
11111111111111111111
1.693151.693151.693151.693150000000000000000
22220000000000000000
84756
genus

4.674.674.674.674.674.674.674.674.674.674.674.674.674.674.674.674.674.674.674.67
11111111111111111111
1.693151.693151.693151.693150000000000000000
22220000000000000000
84757
species

4.674.674.674.674.674.674.674.674.674.674.674.674.674.674.674.674.674.674.674.67
11111111111111111111
1.693151.693151.693151.693150000000000000000
22220000000000000000
471853

Beutenbergia\_cavernae\_DSM\_12333
Beutenbergia\_cavernae\_DSM\_12333
Beutenbergia\_cavernae\_DSM\_12333
Beutenbergia\_cavernae\_DSM\_12333
Beutenbergia\_cavernae\_DSM\_12333
Beutenbergia\_cavernae\_DSM\_12333
Beutenbergia\_cavernae\_DSM\_12333
Beutenbergia\_cavernae\_DSM\_12333
Beutenbergia\_cavernae\_DSM\_12333
Beutenbergia\_cavernae\_DSM\_12333
Beutenbergia\_cavernae\_DSM\_12333
Beutenbergia\_cavernae\_DSM\_12333
Beutenbergia\_cavernae\_DSM\_12333
Beutenbergia\_cavernae\_DSM\_12333
Beutenbergia\_cavernae\_DSM\_12333
Beutenbergia\_cavernae\_DSM\_12333
Beutenbergia\_cavernae\_DSM\_12333
Beutenbergia\_cavernae\_DSM\_12333
Beutenbergia\_cavernae\_DSM\_12333
Beutenbergia\_cavernae\_DSM\_12333
no rank

7.147.147.147.147.147.147.147.147.147.147.147.147.147.147.147.147.147.147.147.14
22222222222222222222
3.065772.553122.758112.470430000000000000000
85640000000000000000
85017
family

3.833.833.833.833.833.833.833.833.833.833.833.833.833.833.833.833.833.833.833.83
11111111111111111111
3.302592.945913.079442.791760000000000000000
107860000000000000000
186188
genus

3.833.833.833.833.833.833.833.833.833.833.833.833.833.833.833.833.833.833.833.83
11111111111111111111
3.302592.945913.079442.791760000000000000000
107860000000000000000
186189
species

3.833.833.833.833.833.833.833.833.833.833.833.833.833.833.833.833.833.833.833.83
11111111111111111111
3.302592.945913.079442.791760000000000000000
107860000000000000000
446471

Xylanimonas\_cellulosilytica\_DSM\_15894
Xylanimonas\_cellulosilytica\_DSM\_15894
Xylanimonas\_cellulosilytica\_DSM\_15894
Xylanimonas\_cellulosilytica\_DSM\_15894
Xylanimonas\_cellulosilytica\_DSM\_15894
Xylanimonas\_cellulosilytica\_DSM\_15894
Xylanimonas\_cellulosilytica\_DSM\_15894
Xylanimonas\_cellulosilytica\_DSM\_15894
Xylanimonas\_cellulosilytica\_DSM\_15894
Xylanimonas\_cellulosilytica\_DSM\_15894
Xylanimonas\_cellulosilytica\_DSM\_15894
Xylanimonas\_cellulosilytica\_DSM\_15894
Xylanimonas\_cellulosilytica\_DSM\_15894
Xylanimonas\_cellulosilytica\_DSM\_15894
Xylanimonas\_cellulosilytica\_DSM\_15894
Xylanimonas\_cellulosilytica\_DSM\_15894
Xylanimonas\_cellulosilytica\_DSM\_15894
Xylanimonas\_cellulosilytica\_DSM\_15894
Xylanimonas\_cellulosilytica\_DSM\_15894
Xylanimonas\_cellulosilytica\_DSM\_15894
no rank

3.313.313.313.313.313.313.313.313.313.313.313.313.313.313.313.313.313.313.313.31
11111111111111111111
2.791762.098612.386292.098610000000000000000
63430000000000000000
254250
genus

3.313.313.313.313.313.313.313.313.313.313.313.313.313.313.313.313.313.313.313.31
11111111111111111111
2.791762.098612.386292.098610000000000000000
63430000000000000000
139208
species

3.313.313.313.313.313.313.313.313.313.313.313.313.313.313.313.313.313.313.313.31
11111111111111111111
2.791762.098612.386292.098610000000000000000
63430000000000000000
743718

Isoptericola\_variabilis\_225
Isoptericola\_variabilis\_225
Isoptericola\_variabilis\_225
Isoptericola\_variabilis\_225
Isoptericola\_variabilis\_225
Isoptericola\_variabilis\_225
Isoptericola\_variabilis\_225
Isoptericola\_variabilis\_225
Isoptericola\_variabilis\_225
Isoptericola\_variabilis\_225
Isoptericola\_variabilis\_225
Isoptericola\_variabilis\_225
Isoptericola\_variabilis\_225
Isoptericola\_variabilis\_225
Isoptericola\_variabilis\_225
Isoptericola\_variabilis\_225
Isoptericola\_variabilis\_225
Isoptericola\_variabilis\_225
Isoptericola\_variabilis\_225
Isoptericola\_variabilis\_225
no rank

2.792.792.792.792.792.792.792.792.792.792.792.792.792.792.792.792.792.792.792.79
11111111111111111111
3.890372.945912.386292.609440000000000000000
187450000000000000000
145357
family

2.792.792.792.792.792.792.792.792.792.792.792.792.792.792.792.792.792.792.792.79
11111111111111111111
3.890372.945912.386292.609440000000000000000
187450000000000000000
57499
genus

2.792.792.792.792.792.792.792.792.792.792.792.792.792.792.792.792.792.792.792.79
11111111111111111111
3.890372.945912.386292.609440000000000000000
187450000000000000000
1276
species

2.792.792.792.792.792.792.792.792.792.792.792.792.792.792.792.792.792.792.792.79
11111111111111111111
3.890372.945912.386292.609440000000000000000
187450000000000000000
478801

Kytococcus\_sedentarius\_DSM\_20547
Kytococcus\_sedentarius\_DSM\_20547
Kytococcus\_sedentarius\_DSM\_20547
Kytococcus\_sedentarius\_DSM\_20547
Kytococcus\_sedentarius\_DSM\_20547
Kytococcus\_sedentarius\_DSM\_20547
Kytococcus\_sedentarius\_DSM\_20547
Kytococcus\_sedentarius\_DSM\_20547
Kytococcus\_sedentarius\_DSM\_20547
Kytococcus\_sedentarius\_DSM\_20547
Kytococcus\_sedentarius\_DSM\_20547
Kytococcus\_sedentarius\_DSM\_20547
Kytococcus\_sedentarius\_DSM\_20547
Kytococcus\_sedentarius\_DSM\_20547
Kytococcus\_sedentarius\_DSM\_20547
Kytococcus\_sedentarius\_DSM\_20547
Kytococcus\_sedentarius\_DSM\_20547
Kytococcus\_sedentarius\_DSM\_20547
Kytococcus\_sedentarius\_DSM\_20547
Kytococcus\_sedentarius\_DSM\_20547
no rank

1.861.861.861.861.861.861.861.861.861.861.861.861.861.861.861.861.861.861.861.86
22222222222222222222
2.497872.386292.386292.497870.50.5000.84657400000000000
44441100100000000000
577468
no rank

1.861.861.861.861.861.861.861.861.861.861.861.861.861.861.861.861.861.861.861.86
22222222222222222222
2.497872.386292.386292.497870.50.5000.84657400000000000
44441100100000000000
2038
genus

1.861.861.861.861.861.861.861.861.861.861.861.861.861.861.861.861.861.861.861.86
22222222222222222222
2.497872.386292.386292.497870.50.5000.84657400000000000
44441100100000000000
2039
species

0.930.930.930.930.930.930.930.930.930.930.930.930.930.930.930.930.930.930.930.93
11111111111111111111
2.386292.386292.386292.386290000000000000000
44440000000000000000
218496

Tropheryma\_whipplei\_TW08\_27
Tropheryma\_whipplei\_TW08\_27
Tropheryma\_whipplei\_TW08\_27
Tropheryma\_whipplei\_TW08\_27
Tropheryma\_whipplei\_TW08\_27
Tropheryma\_whipplei\_TW08\_27
Tropheryma\_whipplei\_TW08\_27
Tropheryma\_whipplei\_TW08\_27
Tropheryma\_whipplei\_TW08\_27
Tropheryma\_whipplei\_TW08\_27
Tropheryma\_whipplei\_TW08\_27
Tropheryma\_whipplei\_TW08\_27
Tropheryma\_whipplei\_TW08\_27
Tropheryma\_whipplei\_TW08\_27
Tropheryma\_whipplei\_TW08\_27
Tropheryma\_whipplei\_TW08\_27
Tropheryma\_whipplei\_TW08\_27
Tropheryma\_whipplei\_TW08\_27
Tropheryma\_whipplei\_TW08\_27
Tropheryma\_whipplei\_TW08\_27
no rank

0.930.930.930.930.930.930.930.930.930.930.930.930.930.930.930.930.930.930.930.93
11111111111111111111
2.609442.386292.386292.6094411001.6931500000000000
54451100200000000000
203267

Tropheryma\_whipplei\_str.\_Twist
Tropheryma\_whipplei\_str.\_Twist
Tropheryma\_whipplei\_str.\_Twist
Tropheryma\_whipplei\_str.\_Twist
Tropheryma\_whipplei\_str.\_Twist
Tropheryma\_whipplei\_str.\_Twist
Tropheryma\_whipplei\_str.\_Twist
Tropheryma\_whipplei\_str.\_Twist
Tropheryma\_whipplei\_str.\_Twist
Tropheryma\_whipplei\_str.\_Twist
Tropheryma\_whipplei\_str.\_Twist
Tropheryma\_whipplei\_str.\_Twist
Tropheryma\_whipplei\_str.\_Twist
Tropheryma\_whipplei\_str.\_Twist
Tropheryma\_whipplei\_str.\_Twist
Tropheryma\_whipplei\_str.\_Twist
Tropheryma\_whipplei\_str.\_Twist
Tropheryma\_whipplei\_str.\_Twist
Tropheryma\_whipplei\_str.\_Twist
Tropheryma\_whipplei\_str.\_Twist
no rank

4.024.024.024.024.024.024.024.024.024.024.024.024.024.024.024.024.024.024.024.02
11111111111111111111
3.639063.302592.945912.945910000000000000000
1410770000000000000000
85021
family

4.024.024.024.024.024.024.024.024.024.024.024.024.024.024.024.024.024.024.024.02
11111111111111111111
3.639063.302592.945912.945910000000000000000
1410770000000000000000
53357
genus

4.024.024.024.024.024.024.024.024.024.024.024.024.024.024.024.024.024.024.024.02
11111111111111111111
3.639063.302592.945912.945910000000000000000
1410770000000000000000
53358
species

4.024.024.024.024.024.024.024.024.024.024.024.024.024.024.024.024.024.024.024.02
11111111111111111111
3.639063.302592.945912.945910000000000000000
1410770000000000000000
710696

Intrasporangium\_calvum\_DSM\_43043
Intrasporangium\_calvum\_DSM\_43043
Intrasporangium\_calvum\_DSM\_43043
Intrasporangium\_calvum\_DSM\_43043
Intrasporangium\_calvum\_DSM\_43043
Intrasporangium\_calvum\_DSM\_43043
Intrasporangium\_calvum\_DSM\_43043
Intrasporangium\_calvum\_DSM\_43043
Intrasporangium\_calvum\_DSM\_43043
Intrasporangium\_calvum\_DSM\_43043
Intrasporangium\_calvum\_DSM\_43043
Intrasporangium\_calvum\_DSM\_43043
Intrasporangium\_calvum\_DSM\_43043
Intrasporangium\_calvum\_DSM\_43043
Intrasporangium\_calvum\_DSM\_43043
Intrasporangium\_calvum\_DSM\_43043
Intrasporangium\_calvum\_DSM\_43043
Intrasporangium\_calvum\_DSM\_43043
Intrasporangium\_calvum\_DSM\_43043
Intrasporangium\_calvum\_DSM\_43043
no rank

11.9211.9211.9211.9211.9211.9211.9211.9211.9211.9211.9211.9211.9211.9211.9211.9211.9211.9211.9211.92
33333333333333333333
2.201672.081591.838391.693150.3582210000.35822100000000000
33221000100000000000
85016
family

11.9211.9211.9211.9211.9211.9211.9211.9211.9211.9211.9211.9211.9211.9211.9211.9211.9211.9211.9211.92
33333333333333333333
2.201672.081591.838391.693150.3582210000.35822100000000000
33221000100000000000
1707
genus

3.533.533.533.533.533.533.533.533.533.533.533.533.533.533.533.533.533.533.533.53
11111111111111111111
2.098611.693151.693151.693150000000000000000
32220000000000000000
11
species

3.533.533.533.533.533.533.533.533.533.533.533.533.533.533.533.533.533.533.533.53
11111111111111111111
2.098611.693151.693151.693150000000000000000
32220000000000000000
593907

[Cellvibrio]\_gilvus\_ATCC\_13127
[Cellvibrio]\_gilvus\_ATCC\_13127
[Cellvibrio]\_gilvus\_ATCC\_13127
[Cellvibrio]\_gilvus\_ATCC\_13127
[Cellvibrio]\_gilvus\_ATCC\_13127
[Cellvibrio]\_gilvus\_ATCC\_13127
[Cellvibrio]\_gilvus\_ATCC\_13127
[Cellvibrio]\_gilvus\_ATCC\_13127
[Cellvibrio]\_gilvus\_ATCC\_13127
[Cellvibrio]\_gilvus\_ATCC\_13127
[Cellvibrio]\_gilvus\_ATCC\_13127
[Cellvibrio]\_gilvus\_ATCC\_13127
[Cellvibrio]\_gilvus\_ATCC\_13127
[Cellvibrio]\_gilvus\_ATCC\_13127
[Cellvibrio]\_gilvus\_ATCC\_13127
[Cellvibrio]\_gilvus\_ATCC\_13127
[Cellvibrio]\_gilvus\_ATCC\_13127
[Cellvibrio]\_gilvus\_ATCC\_13127
[Cellvibrio]\_gilvus\_ATCC\_13127
[Cellvibrio]\_gilvus\_ATCC\_13127
no rank

4.124.124.124.124.124.124.124.124.124.124.124.124.124.124.124.124.124.124.124.12
11111111111111111111
2.098612.098611.693151.693150000000000000000
33220000000000000000
1711
species

4.124.124.124.124.124.124.124.124.124.124.124.124.124.124.124.124.124.124.124.12
11111111111111111111
2.098612.098611.693151.693150000000000000000
33220000000000000000
446466

Cellulomonas\_flavigena\_DSM\_20109
Cellulomonas\_flavigena\_DSM\_20109
Cellulomonas\_flavigena\_DSM\_20109
Cellulomonas\_flavigena\_DSM\_20109
Cellulomonas\_flavigena\_DSM\_20109
Cellulomonas\_flavigena\_DSM\_20109
Cellulomonas\_flavigena\_DSM\_20109
Cellulomonas\_flavigena\_DSM\_20109
Cellulomonas\_flavigena\_DSM\_20109
Cellulomonas\_flavigena\_DSM\_20109
Cellulomonas\_flavigena\_DSM\_20109
Cellulomonas\_flavigena\_DSM\_20109
Cellulomonas\_flavigena\_DSM\_20109
Cellulomonas\_flavigena\_DSM\_20109
Cellulomonas\_flavigena\_DSM\_20109
Cellulomonas\_flavigena\_DSM\_20109
Cellulomonas\_flavigena\_DSM\_20109
Cellulomonas\_flavigena\_DSM\_20109
Cellulomonas\_flavigena\_DSM\_20109
Cellulomonas\_flavigena\_DSM\_20109
no rank

4.274.274.274.274.274.274.274.274.274.274.274.274.274.274.274.274.274.274.274.27
11111111111111111111
2.386292.386292.098611.693151000100000000000
44321000100000000000
1708
species

4.274.274.274.274.274.274.274.274.274.274.274.274.274.274.274.274.274.274.274.27
11111111111111111111
2.386292.386292.098611.693151000100000000000
44321000100000000000
590998

Cellulomonas\_fimi\_ATCC\_484
Cellulomonas\_fimi\_ATCC\_484
Cellulomonas\_fimi\_ATCC\_484
Cellulomonas\_fimi\_ATCC\_484
Cellulomonas\_fimi\_ATCC\_484
Cellulomonas\_fimi\_ATCC\_484
Cellulomonas\_fimi\_ATCC\_484
Cellulomonas\_fimi\_ATCC\_484
Cellulomonas\_fimi\_ATCC\_484
Cellulomonas\_fimi\_ATCC\_484
Cellulomonas\_fimi\_ATCC\_484
Cellulomonas\_fimi\_ATCC\_484
Cellulomonas\_fimi\_ATCC\_484
Cellulomonas\_fimi\_ATCC\_484
Cellulomonas\_fimi\_ATCC\_484
Cellulomonas\_fimi\_ATCC\_484
Cellulomonas\_fimi\_ATCC\_484
Cellulomonas\_fimi\_ATCC\_484
Cellulomonas\_fimi\_ATCC\_484
Cellulomonas\_fimi\_ATCC\_484
no rank

4.254.254.254.254.254.254.254.254.254.254.254.254.254.254.254.254.254.254.254.25
11111111111111111111
2.386292.386292.386292.386290000100000000000
44440000100000000000
145360
family

4.254.254.254.254.254.254.254.254.254.254.254.254.254.254.254.254.254.254.254.25
11111111111111111111
2.386292.386292.386292.386290000100000000000
44440000100000000000
60919
genus

4.254.254.254.254.254.254.254.254.254.254.254.254.254.254.254.254.254.254.254.25
11111111111111111111
2.386292.386292.386292.386290000100000000000
44440000100000000000
60920
species

4.254.254.254.254.254.254.254.254.254.254.254.254.254.254.254.254.254.254.254.25
11111111111111111111
2.386292.386292.386292.386290000100000000000
44440000100000000000
446469

Sanguibacter\_keddieii\_DSM\_10542
Sanguibacter\_keddieii\_DSM\_10542
Sanguibacter\_keddieii\_DSM\_10542
Sanguibacter\_keddieii\_DSM\_10542
Sanguibacter\_keddieii\_DSM\_10542
Sanguibacter\_keddieii\_DSM\_10542
Sanguibacter\_keddieii\_DSM\_10542
Sanguibacter\_keddieii\_DSM\_10542
Sanguibacter\_keddieii\_DSM\_10542
Sanguibacter\_keddieii\_DSM\_10542
Sanguibacter\_keddieii\_DSM\_10542
Sanguibacter\_keddieii\_DSM\_10542
Sanguibacter\_keddieii\_DSM\_10542
Sanguibacter\_keddieii\_DSM\_10542
Sanguibacter\_keddieii\_DSM\_10542
Sanguibacter\_keddieii\_DSM\_10542
Sanguibacter\_keddieii\_DSM\_10542
Sanguibacter\_keddieii\_DSM\_10542
Sanguibacter\_keddieii\_DSM\_10542
Sanguibacter\_keddieii\_DSM\_10542
no rank

2.752.752.752.752.752.752.752.752.752.752.752.752.752.752.752.752.752.752.752.75
11111111111111111111
2.945912.945912.945912.945911.693151001.6931510000000000
77772100210000000000
85022
family

2.752.752.752.752.752.752.752.752.752.752.752.752.752.752.752.752.752.752.752.75
11111111111111111111
2.945912.945912.945912.945911.693151001.6931510000000000
77772100210000000000
43673
genus

2.752.752.752.752.752.752.752.752.752.752.752.752.752.752.752.752.752.752.752.75
11111111111111111111
2.945912.945912.945912.945911.693151001.6931510000000000
77772100210000000000
43674
species

2.752.752.752.752.752.752.752.752.752.752.752.752.752.752.752.752.752.752.752.75
11111111111111111111
2.945912.945912.945912.945911.693151001.6931510000000000
77772100210000000000
471856

Jonesia\_denitrificans\_DSM\_20603
Jonesia\_denitrificans\_DSM\_20603
Jonesia\_denitrificans\_DSM\_20603
Jonesia\_denitrificans\_DSM\_20603
Jonesia\_denitrificans\_DSM\_20603
Jonesia\_denitrificans\_DSM\_20603
Jonesia\_denitrificans\_DSM\_20603
Jonesia\_denitrificans\_DSM\_20603
Jonesia\_denitrificans\_DSM\_20603
Jonesia\_denitrificans\_DSM\_20603
Jonesia\_denitrificans\_DSM\_20603
Jonesia\_denitrificans\_DSM\_20603
Jonesia\_denitrificans\_DSM\_20603
Jonesia\_denitrificans\_DSM\_20603
Jonesia\_denitrificans\_DSM\_20603
Jonesia\_denitrificans\_DSM\_20603
Jonesia\_denitrificans\_DSM\_20603
Jonesia\_denitrificans\_DSM\_20603
Jonesia\_denitrificans\_DSM\_20603
Jonesia\_denitrificans\_DSM\_20603
no rank

4.964.964.964.964.964.964.964.964.964.964.964.964.964.964.964.964.964.964.964.96
11111111111111111111
2.945912.609442.386292.386291.69315000000000000000
75442000000000000000
622452
suborder

4.964.964.964.964.964.964.964.964.964.964.964.964.964.964.964.964.964.964.964.96
11111111111111111111
2.945912.609442.386292.386291.69315000000000000000
75442000000000000000
83778
family

4.964.964.964.964.964.964.964.964.964.964.964.964.964.964.964.964.964.964.964.96
11111111111111111111
2.945912.609442.386292.386291.69315000000000000000
75442000000000000000
33981
genus

4.964.964.964.964.964.964.964.964.964.964.964.964.964.964.964.964.964.964.964.96
11111111111111111111
2.945912.609442.386292.386291.69315000000000000000
75442000000000000000
131568
species

4.964.964.964.964.964.964.964.964.964.964.964.964.964.964.964.964.964.964.964.96
11111111111111111111
2.945912.609442.386292.386291.69315000000000000000
75442000000000000000
266940

Kineococcus\_radiotolerans\_SRS30216
Kineococcus\_radiotolerans\_SRS30216
Kineococcus\_radiotolerans\_SRS30216
Kineococcus\_radiotolerans\_SRS30216
Kineococcus\_radiotolerans\_SRS30216
Kineococcus\_radiotolerans\_SRS30216
Kineococcus\_radiotolerans\_SRS30216
Kineococcus\_radiotolerans\_SRS30216
Kineococcus\_radiotolerans\_SRS30216
Kineococcus\_radiotolerans\_SRS30216
Kineococcus\_radiotolerans\_SRS30216
Kineococcus\_radiotolerans\_SRS30216
Kineococcus\_radiotolerans\_SRS30216
Kineococcus\_radiotolerans\_SRS30216
Kineococcus\_radiotolerans\_SRS30216
Kineococcus\_radiotolerans\_SRS30216
Kineococcus\_radiotolerans\_SRS30216
Kineococcus\_radiotolerans\_SRS30216
Kineococcus\_radiotolerans\_SRS30216
Kineococcus\_radiotolerans\_SRS30216
no rank

6.846.846.846.846.846.846.846.846.846.846.846.846.846.846.846.846.846.846.846.84
11111111111111111111
1.693151.693151.693151.693151000000000000000
22221000000000000000
85014
suborder

6.846.846.846.846.846.846.846.846.846.846.846.846.846.846.846.846.846.846.846.84
11111111111111111111
1.693151.693151.693151.693151000000000000000
22221000000000000000
85034
family

6.846.846.846.846.846.846.846.846.846.846.846.846.846.846.846.846.846.846.846.84
11111111111111111111
1.693151.693151.693151.693151000000000000000
22221000000000000000
283810
genus

6.846.846.846.846.846.846.846.846.846.846.846.846.846.846.846.846.846.846.846.84
11111111111111111111
1.693151.693151.693151.693151000000000000000
22221000000000000000
283811
species

6.846.846.846.846.846.846.846.846.846.846.846.846.846.846.846.846.846.846.846.84
11111111111111111111
1.693151.693151.693151.693151000000000000000
22221000000000000000
446470

Stackebrandtia\_nassauensis\_DSM\_44728
Stackebrandtia\_nassauensis\_DSM\_44728
Stackebrandtia\_nassauensis\_DSM\_44728
Stackebrandtia\_nassauensis\_DSM\_44728
Stackebrandtia\_nassauensis\_DSM\_44728
Stackebrandtia\_nassauensis\_DSM\_44728
Stackebrandtia\_nassauensis\_DSM\_44728
Stackebrandtia\_nassauensis\_DSM\_44728
Stackebrandtia\_nassauensis\_DSM\_44728
Stackebrandtia\_nassauensis\_DSM\_44728
Stackebrandtia\_nassauensis\_DSM\_44728
Stackebrandtia\_nassauensis\_DSM\_44728
Stackebrandtia\_nassauensis\_DSM\_44728
Stackebrandtia\_nassauensis\_DSM\_44728
Stackebrandtia\_nassauensis\_DSM\_44728
Stackebrandtia\_nassauensis\_DSM\_44728
Stackebrandtia\_nassauensis\_DSM\_44728
Stackebrandtia\_nassauensis\_DSM\_44728
Stackebrandtia\_nassauensis\_DSM\_44728
Stackebrandtia\_nassauensis\_DSM\_44728
no rank

49.7049.7049.7049.7049.7049.7049.7049.7049.7049.7049.7049.7049.7049.7049.7049.7049.7049.7049.7049.70
77777777777777777777
3.350133.085942.971952.780980.7515260.406640.1164990.1164990.3831650.1164990.1164990.1164990.1164990.1164990.1164990.1164990.1164990.1164990.1164990.116499
108761100100000000000
85008
suborder

49.7049.7049.7049.7049.7049.7049.7049.7049.7049.7049.7049.7049.7049.7049.7049.7049.7049.7049.7049.70
77777777777777777777
3.350133.085942.971952.780980.7515260.406640.1164990.1164990.3831650.1164990.1164990.1164990.1164990.1164990.1164990.1164990.1164990.1164990.1164990.116499
108761100100000000000
28056
family

18.0118.0118.0118.0118.0118.0118.0118.0118.0118.0118.0118.0118.0118.0118.0118.0118.0118.0118.0118.01
22222222222222222222
3.440273.234513.079442.945910.8686660.513048000.51304800000000000
119871100100000000000
1865
genus

9.249.249.249.249.249.249.249.249.249.249.249.249.249.249.249.249.249.249.249.24
11111111111111111111
3.39793.079443.079442.945911.69315100100000000000
118872100100000000000
134676

Actinoplanes\_sp.\_SE50\_110
Actinoplanes\_sp.\_SE50\_110
Actinoplanes\_sp.\_SE50\_110
Actinoplanes\_sp.\_SE50\_110
Actinoplanes\_sp.\_SE50\_110
Actinoplanes\_sp.\_SE50\_110
Actinoplanes\_sp.\_SE50\_110
Actinoplanes\_sp.\_SE50\_110
Actinoplanes\_sp.\_SE50\_110
Actinoplanes\_sp.\_SE50\_110
Actinoplanes\_sp.\_SE50\_110
Actinoplanes\_sp.\_SE50\_110
Actinoplanes\_sp.\_SE50\_110
Actinoplanes\_sp.\_SE50\_110
Actinoplanes\_sp.\_SE50\_110
Actinoplanes\_sp.\_SE50\_110
Actinoplanes\_sp.\_SE50\_110
Actinoplanes\_sp.\_SE50\_110
Actinoplanes\_sp.\_SE50\_110
Actinoplanes\_sp.\_SE50\_110
species

8.778.778.778.778.778.778.778.778.778.778.778.778.778.778.778.778.778.778.778.77
11111111111111111111
3.484913.39793.079442.945910000000000000000
1211870000000000000000
1866
species

8.778.778.778.778.778.778.778.778.778.778.778.778.778.778.778.778.778.778.778.77
11111111111111111111
3.484913.39793.079442.945910000000000000000
1211870000000000000000
512565

Actinoplanes\_missouriensis\_431
Actinoplanes\_missouriensis\_431
Actinoplanes\_missouriensis\_431
Actinoplanes\_missouriensis\_431
Actinoplanes\_missouriensis\_431
Actinoplanes\_missouriensis\_431
Actinoplanes\_missouriensis\_431
Actinoplanes\_missouriensis\_431
Actinoplanes\_missouriensis\_431
Actinoplanes\_missouriensis\_431
Actinoplanes\_missouriensis\_431
Actinoplanes\_missouriensis\_431
Actinoplanes\_missouriensis\_431
Actinoplanes\_missouriensis\_431
Actinoplanes\_missouriensis\_431
Actinoplanes\_missouriensis\_431
Actinoplanes\_missouriensis\_431
Actinoplanes\_missouriensis\_431
Actinoplanes\_missouriensis\_431
Actinoplanes\_missouriensis\_431
no rank

6.736.736.736.736.736.736.736.736.736.736.736.736.736.736.736.736.736.736.736.73
11111111111111111111
2.609442.609442.609442.609440000000000000000
55550000000000000000
84593
genus

6.736.736.736.736.736.736.736.736.736.736.736.736.736.736.736.736.736.736.736.73
11111111111111111111
2.609442.609442.609442.609440000000000000000
55550000000000000000
1003110
species

6.736.736.736.736.736.736.736.736.736.736.736.736.736.736.736.736.736.736.736.73
11111111111111111111
2.609442.609442.609442.609440000000000000000
55550000000000000000
263358

Verrucosispora\_maris\_AB-18-032
Verrucosispora\_maris\_AB-18-032
Verrucosispora\_maris\_AB-18-032
Verrucosispora\_maris\_AB-18-032
Verrucosispora\_maris\_AB-18-032
Verrucosispora\_maris\_AB-18-032
Verrucosispora\_maris\_AB-18-032
Verrucosispora\_maris\_AB-18-032
Verrucosispora\_maris\_AB-18-032
Verrucosispora\_maris\_AB-18-032
Verrucosispora\_maris\_AB-18-032
Verrucosispora\_maris\_AB-18-032
Verrucosispora\_maris\_AB-18-032
Verrucosispora\_maris\_AB-18-032
Verrucosispora\_maris\_AB-18-032
Verrucosispora\_maris\_AB-18-032
Verrucosispora\_maris\_AB-18-032
Verrucosispora\_maris\_AB-18-032
Verrucosispora\_maris\_AB-18-032
Verrucosispora\_maris\_AB-18-032
no rank

13.9913.9913.9913.9913.9913.9913.9913.9913.9913.9913.9913.9913.9913.9913.9913.9913.9913.9913.9913.99
22222222222222222222
3.190462.843262.609442.241730.850809000000000000000
96531000000000000000
1873
genus

7.037.037.037.037.037.037.037.037.037.037.037.037.037.037.037.037.037.037.037.03
11111111111111111111
3.079442.609442.609442.098611.69315000000000000000
85532000000000000000
47850
species

7.037.037.037.037.037.037.037.037.037.037.037.037.037.037.037.037.037.037.037.03
11111111111111111111
3.079442.609442.609442.098611.69315000000000000000
85532000000000000000
644283

Micromonospora\_aurantiaca\_ATCC\_27029
Micromonospora\_aurantiaca\_ATCC\_27029
Micromonospora\_aurantiaca\_ATCC\_27029
Micromonospora\_aurantiaca\_ATCC\_27029
Micromonospora\_aurantiaca\_ATCC\_27029
Micromonospora\_aurantiaca\_ATCC\_27029
Micromonospora\_aurantiaca\_ATCC\_27029
Micromonospora\_aurantiaca\_ATCC\_27029
Micromonospora\_aurantiaca\_ATCC\_27029
Micromonospora\_aurantiaca\_ATCC\_27029
Micromonospora\_aurantiaca\_ATCC\_27029
Micromonospora\_aurantiaca\_ATCC\_27029
Micromonospora\_aurantiaca\_ATCC\_27029
Micromonospora\_aurantiaca\_ATCC\_27029
Micromonospora\_aurantiaca\_ATCC\_27029
Micromonospora\_aurantiaca\_ATCC\_27029
Micromonospora\_aurantiaca\_ATCC\_27029
Micromonospora\_aurantiaca\_ATCC\_27029
Micromonospora\_aurantiaca\_ATCC\_27029
Micromonospora\_aurantiaca\_ATCC\_27029
no rank

6.966.966.966.966.966.966.966.966.966.966.966.966.966.966.966.966.966.966.966.96
11111111111111111111
3.302593.079442.609442.386290000000000000000
108540000000000000000
648999

Micromonospora\_sp.\_L5
Micromonospora\_sp.\_L5
Micromonospora\_sp.\_L5
Micromonospora\_sp.\_L5
Micromonospora\_sp.\_L5
Micromonospora\_sp.\_L5
Micromonospora\_sp.\_L5
Micromonospora\_sp.\_L5
Micromonospora\_sp.\_L5
Micromonospora\_sp.\_L5
Micromonospora\_sp.\_L5
Micromonospora\_sp.\_L5
Micromonospora\_sp.\_L5
Micromonospora\_sp.\_L5
Micromonospora\_sp.\_L5
Micromonospora\_sp.\_L5
Micromonospora\_sp.\_L5
Micromonospora\_sp.\_L5
Micromonospora\_sp.\_L5
Micromonospora\_sp.\_L5
species

10.9710.9710.9710.9710.9710.9710.9710.9710.9710.9710.9710.9710.9710.9710.9710.9710.9710.9710.9710.97
22222222222222222222
3.86023.443823.480183.303140.89364810.5278030.5278030.8936480.5278030.5278030.5278030.5278030.5278030.5278030.5278030.5278030.5278030.5278030.527803
171212101111111111111111
168694
genus

5.795.795.795.795.795.795.795.795.795.795.795.795.795.795.795.795.795.795.795.79
11111111111111111111
3.833213.484913.639063.39791.693151111.6931511111111111
171214112111211111111111
168697
species

5.795.795.795.795.795.795.795.795.795.795.795.795.795.795.795.795.795.795.795.79
11111111111111111111
3.833213.484913.639063.39791.693151111.6931511111111111
171214112111211111111111
391037

Salinispora\_arenicola\_CNS-205
Salinispora\_arenicola\_CNS-205
Salinispora\_arenicola\_CNS-205
Salinispora\_arenicola\_CNS-205
Salinispora\_arenicola\_CNS-205
Salinispora\_arenicola\_CNS-205
Salinispora\_arenicola\_CNS-205
Salinispora\_arenicola\_CNS-205
Salinispora\_arenicola\_CNS-205
Salinispora\_arenicola\_CNS-205
Salinispora\_arenicola\_CNS-205
Salinispora\_arenicola\_CNS-205
Salinispora\_arenicola\_CNS-205
Salinispora\_arenicola\_CNS-205
Salinispora\_arenicola\_CNS-205
Salinispora\_arenicola\_CNS-205
Salinispora\_arenicola\_CNS-205
Salinispora\_arenicola\_CNS-205
Salinispora\_arenicola\_CNS-205
Salinispora\_arenicola\_CNS-205
no rank

5.185.185.185.185.185.185.185.185.185.185.185.185.185.185.185.185.185.185.185.18
11111111111111111111
3.890373.39793.302593.197220100000000000000
18111090100000000000000
168695
species

5.185.185.185.185.185.185.185.185.185.185.185.185.185.185.185.185.185.185.185.18
11111111111111111111
3.890373.39793.302593.197220100000000000000
18111090100000000000000
369723

Salinispora\_tropica\_CNB-440
Salinispora\_tropica\_CNB-440
Salinispora\_tropica\_CNB-440
Salinispora\_tropica\_CNB-440
Salinispora\_tropica\_CNB-440
Salinispora\_tropica\_CNB-440
Salinispora\_tropica\_CNB-440
Salinispora\_tropica\_CNB-440
Salinispora\_tropica\_CNB-440
Salinispora\_tropica\_CNB-440
Salinispora\_tropica\_CNB-440
Salinispora\_tropica\_CNB-440
Salinispora\_tropica\_CNB-440
Salinispora\_tropica\_CNB-440
Salinispora\_tropica\_CNB-440
Salinispora\_tropica\_CNB-440
Salinispora\_tropica\_CNB-440
Salinispora\_tropica\_CNB-440
Salinispora\_tropica\_CNB-440
Salinispora\_tropica\_CNB-440
no rank

50.9150.9150.9150.9150.9150.9150.9150.9150.9150.9150.9150.9150.9150.9150.9150.9150.9150.9150.9150.91
[truncated: 2,238,898 more chars]
